# 2-Amino-3′-dialkylaminobiphenyl-based fluorescent intracellular probes for nitric oxide surrogate N_2_O_3_†

**DOI:** 10.1039/c9sc04304g

**Published:** 2020-01-02

**Authors:** P. Rogelio Escamilla, Yanming Shen, Quanjuan Zhang, Derek S. Hernandez, Cecil J. Howard, Xuhong Qian, Daria Y. Filonov, Alexander V. Kinev, Jason B. Shear, Eric V. Anslyn, Youjun Yang

**Affiliations:** State Key Laboratory of Bioreactor Engineering, East China University of Science and Technology Meilong Road 130 Shanghai 200237 China youjunyang@ecust.edu.cn; Shanghai Key Laboratory of Chemical Biology, School of Pharmacy, East China University of Science and Technology Meilong Road 130 Shanghai 200237 China; Department of Chemistry, University of Texas at Austin Austin Texas USA anslyn@austin.utexas.edu jshear@cm.utexas.edu; Creative Scientist, Inc. Durham NC USA

## Abstract

Fluorescent probes for nitric oxide (NO), or more frequently for its oxidized surrogate dinitrogen trioxide (N_2_O_3_), have enabled scientists to study the contributions of this signaling molecule to many physiological processes. Seeking to improve upon limitations of other probes, we have developed a family of fluorescent probes based on a 2-amino-3′-dialkylaminobiphenyl core. This core condenses with N_2_O_3_ to form benzo[*c*]cinnoline structures, incorporating the analyte into the newly formed fluorophore, which results in product fluorescence with virtually no background contribution from the initial probe. We varied the substituents in the core in order to optimize both the reactivity of the probes with N_2_O_3_ and their cinnoline products' fluorescence wavelengths and brightness. The top candidates were then applied to cultured cells to verify that they could respond to NO within cellular milieus, and the top performer, NO_530_, was compared with a “gold standard” commercial probe, DAF-FM, in a macrophage-derived cell line, RAW 264.7, stimulated to produce NO. NO_530_ demonstrated similar or better sensitivity and higher selectivity for NO than DAF, making it an attractive potential alternative for NO tracking in various applications.

## Abstract 2

A thorough structure-property relationship study has produced much-improved fluorescence probes for nitric oxide surrogate, *e.g.* N_2_O_3_, compared to the original NO_550_.

## Graphical abstract

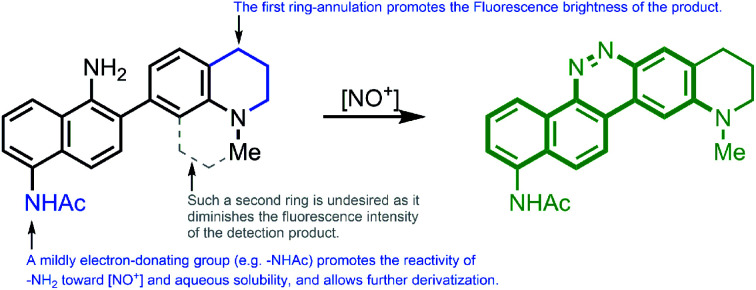

## Introduction

Nitric oxide (NO) is a blood vessel relaxation factor with multiple roles in physiological signaling.^1–5^ Considering how NO is integrally involved in cardiovascular, nervous, immune, and other human body systems, it is no surprise that abnormal NO levels are implicated in numerous pathological conditions, including cardiovascular diseases, circulatory shock, local inflammation, asthma, cancer, stroke, ischemic reperfusion injury, neurodegenerative disorders, depression and diabetes.^6–25^ In order to establish NO levels and activity, scientists have employed NO-specific electrodes^26–34^ and chemical probes that, upon reaction with NO or its surrogates, produce a change in electron paramagnetic resonance (EPR),^35–39^ UV-vis absorbance,^40,41^ chemiluminescence,^42–45^ and/or fluorescence.^46–59^ For monitoring NO in cells and tissues, fluorescence imaging provides a sensitive and spatially defined signal that can be followed over time, often with minimal interference to cell or tissue processes.

**Scheme 1:**
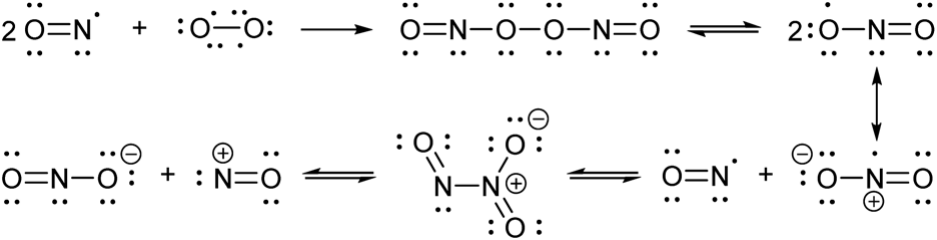
Reactions involving NO and N_2_O_3_.

Nitric oxide synthases (NOSs) produce the free radical NO when they convert l-arginine to l-citrulline.^60^ This neutral and slightly polar dissolved gas diffuses through both aqueous and non-polar (lipid membrane) environments, where it has several fates.^61–63^ Transition metals such as those bound to heme proteins, as well as other radicals, such as superoxide, scavenge NO and therefore contribute to its short lifetime.^24^ NO binds readily (often under diffusion control) to paramagnetic metals. In addition, high oxidation-state metals can be reduced by NO, with concomitant or swiftly ensuing nitrosation of a proximal nucleophile, such as a thiol.^64–66^ If present, superoxide will react with NO in a diffusion-controlled rate. It produces the peroxynitrite anion, a potent oxidizer responsible for DNA mutations.^67^ When triplet oxygen reacts with four equivalents of NO, it forms two equivalents of the potent nitrosating dinitrogen trioxide (N_2_O_3_, Scheme 1). The diradical molecular oxygen first reacts with two NO radicals to form the dinitroso peroxide which homolyzes to the nitrogen dioxide (NO_2_) radical. Reaction with another NO radical produces the asymmetric N_2_O_3_, rather than the symmetric nitrous anhydride.

**Scheme 2:**
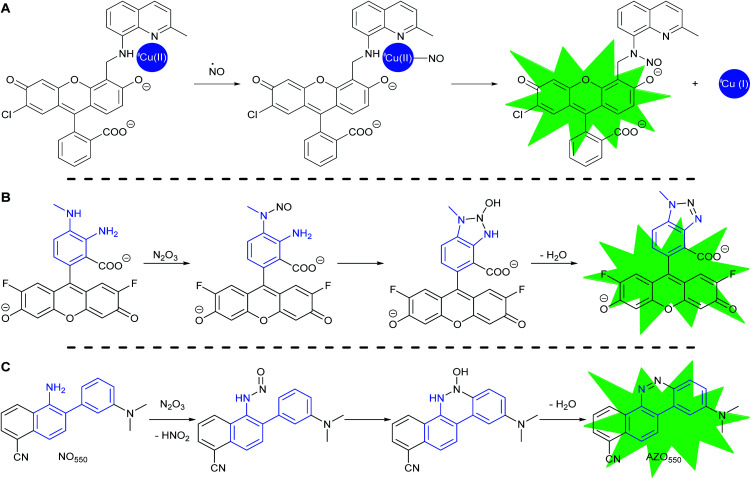
Common strategies to detect NO: (A) CuFL-1 fluorescence turns on with the release of copper after the complex binds NO with subsequent *copper-reductive N*-nitrosation. (B) PET-quenching *ortho*-diaminobenzene (in blue) reacts with N_2_O_3_, the oxidation product of NO, to make non-PET-quenching benzotriazole, with a turn-on of fluorescence, as exemplified by DAF-FM. (C) The reaction of 2-aminobiphenyl scaffold (in blue) with N_2_O_3_ to produce benzo[*c*]cinnoline.

Most fluorescent probes for NO fall into three categories. First are probes inspired by high oxidation state transition metal NO scavengers, in which a fluorescent ligand binds a fluorescence-quenching metal, such as copper(ii), as exemplified by Lippard's Cu(FL) probes.^68–74^ After NO complexation to Cu(ii), nucleophilic attack by the ligand amine reduces the metal to Cu(i). Metal reduction and *N*-nitrosation lower the affinity of the ligand for the metal, to the extent that the metal dissociates and no longer quenches ligand fluorescence (Scheme 2A).

The second category consists of the *ortho*-diaminobenzene family, which detects N_2_O_3_ (Scheme 2B).^75–79^ The diaminobenzene moiety is attached to a fluorophore to produce photoinduced electron-transfer (PET) quenching of fluorescence. Nitrosation of either of the amines, followed by condensation of the other amine upon the *N*-nitrosamine, produces a triazole that can no longer efficiently PET quench fluorescence. In this category lies DAF-FM, one of the most cited probes for NO imaging. It has also been suggested that the nitrosation occurs *via* peroxynitrite, which is formed due to a reaction between superoxide and NO,^80^ however the probes cannot differentiate the source of nitrosation.

Both sensing strategies produce an enhancement, not a wavelength-shift, of emission as the signal of reaction with NO or its oxidized surrogate. Incomplete quenching of fluorescence in the unreacted probes can result in significant background signal. With no change in wavelength, this background degrades the detection limits of the reaction with NO. Transition metal-based probes are most affected by this incomplete quenching, with relatively small increases (3–40×) in fluorescence due to NO addition when compared to the non-metal-based probes (40–1500×). In addition, the performance of *ortho*-diaminobenzene-based probes is limited both by chelation to alkali-earth metals and reaction with other bioavailable dielectrophiles, such as dehydroascorbic acid (DHA) and methyl glyoxal, resulting in the attenuation of PET quenching and thus a non-NO-selective signal.^81–84^

In 2010 Shear, Anslyn, and Yang presented a new fluorescence detection mechanism for NO embodied in the probe NO_550_ (probe **9**, Table 1).^85^ This probe comprises a 2-amino-3′-dimethylaminobiphenyl core that condenses with N_2_O_3_ to produce a benzo[*c*]cinnoline *via* nitrosation of the amine, followed by nucleophilic aryl attack on the nitrosamine, with subsequent loss of water – an intramolecular diazotization, creating AZO_550_ (Scheme 2C).

**Table 1:** The structures of the nitric oxide fluorescent probes for nitric oxide and their photo-physical properties

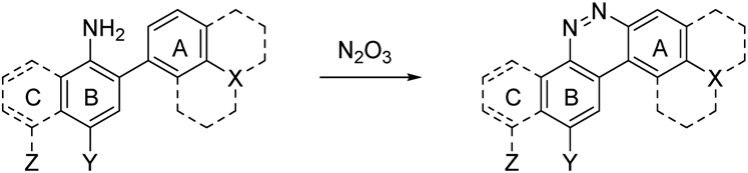											
Unreacted probe					Pr. no	Cinnoline product					
*λ*_abs_ a (nm)	*λ*_em_ b (nm)	*ε* c × 10^3^ M^−1^ cm^−1^	*ϕ* d	Structure		X or Z	*λ*_abs_ a (nm)	*λ*_em_ b (nm)	*ε* c × 10^3^ M^−1^ cm^−1^	*ϕ* d	Relative^e^^,^^f^ brightness
300	420	10.0	0.54	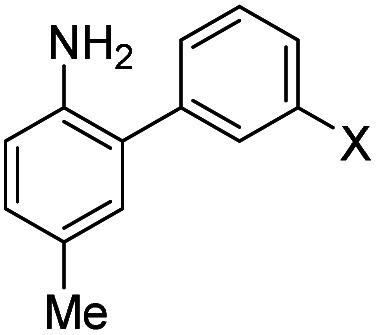	1	–OMe	395	488	14.0	0.09	3.0
300	420	11.0	0.47		2	–OH	395	490	13.2	0.67	20.0
310	415	9.0	0.27		3	–NMe_2_	415	510	14.3	0.02	0.7
300	390	14.4	0.003	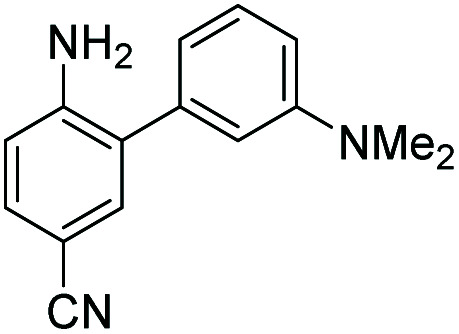	4	—	450	550	16.2	0.003	0.1
310	415	9.9	0.24	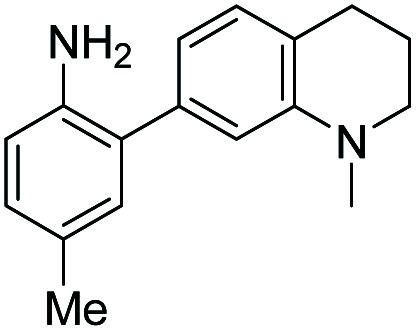	5	—	425	508	20.8	0.62	29.3
300	415	9.5	0.1	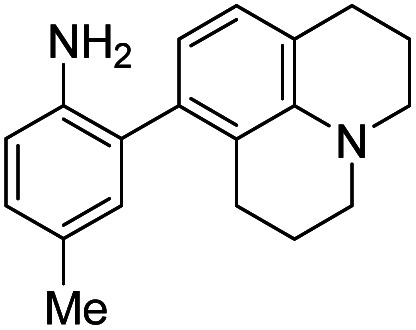	6	—	447	535	11.3	0.36	9.3
350	450	9.9	0.02	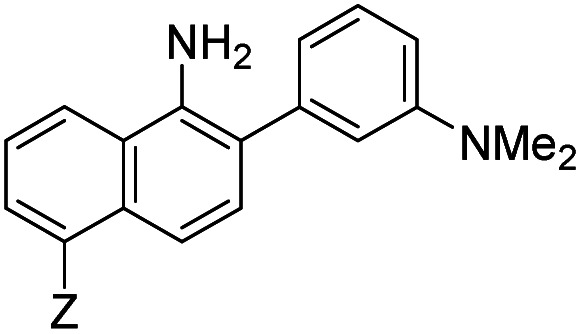	7	–NHAc	433	535	13.0	0.08	2.3
330	445	8.0	0.04		8	–H	430	530	13.9	0.06	1.9
350	n.d.	3.5	n.d.^g^		9	–CN (NO_550_)	450	550	4.0	0.11	1.0
370	455	9.4	0.07	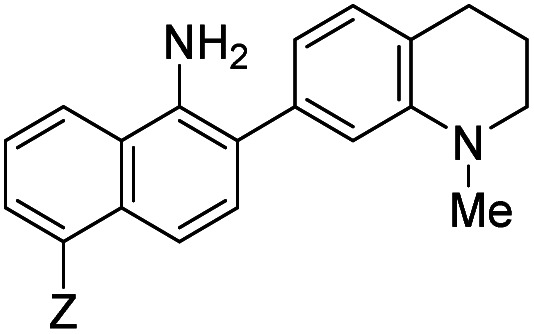	10	–NHAc (NO_530_)	445	530	20.1	0.5	23.0
355	450	10.3	0.02		11	–H	440	525	24.1	0.5	27.5
345	510	11.6	0.03		12	–CN	450	545	7.3	0.13	2.2
420	465	12.2	0.01	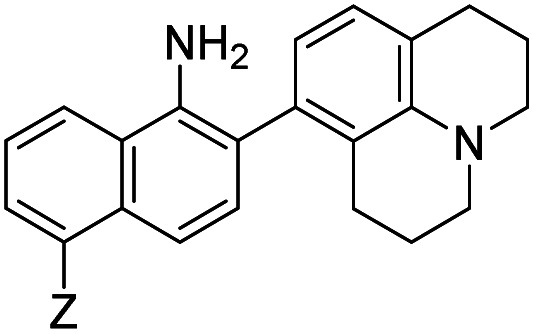	13	–NHAc (NO_562_)	470	562	15.5	0.35	12.3
350	425	11.3	0.01		14	H	466	555	16.9	0.24	9.3
390	515	12.4	0.02		15	CN	480	580	9.0	0.11	2.3

a Wavelength of maximum absorbance for the most bathochromic peak in the UV-vis spectrum.

b Wavelength of maximum emission for the largest peak in the emission spectrum when excited at the most bathochromic absorbance maximum.

c Absorptivity coefficient/1000.

d Quantum efficiency.

e Brightness relative to the cinnoline product of NO_550_ (**9**), previously reported as AZO_550_.

f Cinnoline brightness is defined as the product of the absorptivity coefficient and the quantum efficiency.

g NO_550_ does not fluoresce in 1 : 4 DMSO/50 mM PBS at pH 7.4. The blue values highlight the best performers' fluorescence properties in 1 : 4 DMSO/50 mM PBS at pH 7.4, of the probes and their cinnoline, or azo, products (hereafter denoted as “AZO-Probe Number”), produced from reaction with nitrite in acid.

The excitation and emission wavelengths of AZO_550_ are sufficiently bathochromically-shifted that excitation of AZO_550_ produces virtually no excitation of NO_550_. Such elimination of background signal deriving from NO_550_ results in a 1500-fold enhancement of emission upon full conversion to AZO_550_. Moreover, NO_550_ demonstrates high selectivity for N_2_O_3_ in its fluorescence response, showing no response to 100-fold excess of DHA and 1000-fold excess of other biologically relevant analytes. It either fails to react with the competing analytes, or their reactions produce non-fluorescent products. Unfortunately, AZO_550_'s brightness (defined here as the product of molar absorptivity and fluorescence quantum yield: 440 M^−1^ cm^−1^) is significantly lower than that for fluorescein-based DAF-FM triazole (from reaction of DAF-FM with N_2_O_3_, 59 000 M^−1^ cm^−1^ brightness), due both to AZO_550_'s lower molar absorptivity (4000 M^−1^ cm^−1^*versus* 73 000 M^−1^ cm^−1^) and fluorescence quantum yield (0.11 *versus* 0.81).^76^ Ghebremariam and co-workers found that not even at 75 μM loading concentration did NO_550_ match the intensity of only 1 μM DAF-FM in the same endothelial cells at the same time point.^86^ Loading with higher concentrations of NO_550_ was also hampered by poor water solubility and cytotoxicity. Herein, we describe the systematic substitution of the 2-aminobiphenyl scaffold, the effect of this substitution on reactivity and fluorescence, and the response of the best candidates to exogenous and endogenous NO in cells, and compare these results to those obtained for DAF-FM.

## Results and discussion

### Spectroscopic properties of probes and their cinnoline products

Despite NO_550_'s limitations, the results were sufficiently promising to study the 2-aminobiphenyl core more thoroughly, with the goals of increasing brightness, quantum yield, and red-shifting the fluorescence, all while maintaining the selectivity for NO and the spectral resolution of probe fluorescence *versus* that of the corresponding cinnoline product. Table 1 shows the permutations synthesized and studied. As N_2_O_3_ is dielectrophilic, these probes are dinucleophilic, and positions X and Y on rings A and B, respectively, have the greatest influence on the probes' nucleophilicity. We incorporated ring C to impart bathochromic shifts. Substituent Z on ring C is not only commercially and synthetically convenient but also distant from (and less inductively and sterically interfering with) the N_2_O_3_ reaction sites. It is therefore an attractive attachment point for hydrophobic, hydrophilic, cell-compartment directing, or tethering functionalities. We first evaluated the brightness and fluorescence properties (Table 1), in 1 : 4 DMSO/50 mM PBS at pH 7.4, of the probes and their cinnoline (*i.e.* azo) products (hereafter denoted as AZO-Probe Number, black column) accessed from the reaction of nitrite with acid (ESI page S9†).

**Fig. 1:**
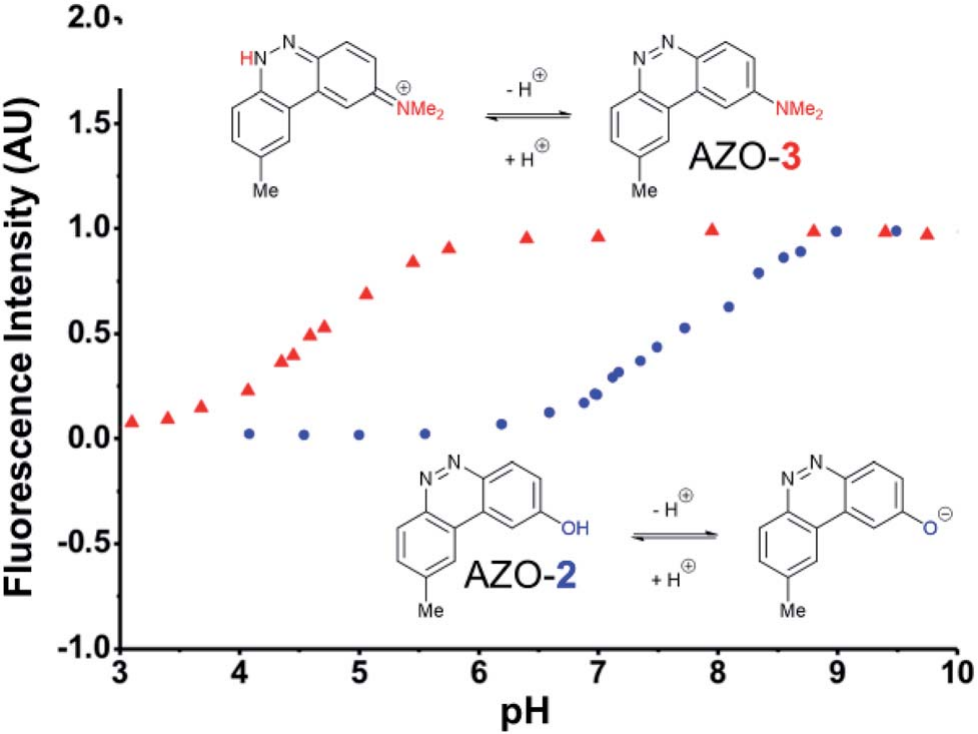
Fluorescence emission pH titration of 5 μM solutions of the cinnoline products of probes **2** (blue) and **3** (red) in 2.5% aqueous DMSO with excitation set at the probes' respective absorbance maxima (Table 1).

The first set studied consisted of simple variants with rings A and B only (no C annulation). The Y substituent was fixed as a methyl group while varying the X substituent on ring A. Since ring A attacks the trapped *N*-nitroso group, to X we assigned electron-donating methoxy (OMe, **1**), hydroxy (OH, **2**), and *N*,*N*-dimethylamino (NMe_2_, **3**) groups. By far, the hydroxyl variant surpassed the others in brightness due to AZO-**2**'s much higher quantum yield of 67%. Unfortunately, this brightness showed a dependence upon the protonation state of the phenol around physiological pH. For fluorescein-based probes, the p*K*_a_ of the phenol in the excited state normally lies around 6.7,^87^ meaning that these probes may non-selectively detect (by quenching of fluorescence) pH in cells. We performed fluorescence pH titrations on AZO-**2** and AZO-**3**, establishing their p*K*_a_'s as 7.8 and 4.5, respectively (Fig. 1). Protonation quenches the fluorescence of both cinnolines, but AZO-**3** provides a pH-independent signal above pH 6. AZO-**1**'s signal is also pH-independent at physiological pH, but we did not pursue it or any variants with X = OMe because of this probe poor reactivity, even with nitrite in acid.

We next evaluated the influence of position Y by changing it from methyl to cyano, postulating that the greater conjugation from NMe_2_ to CN would result in a bathochromic shift of fluorescence wavelengths. The X = NMe_2_ and Y = CN cinnoline variant (AZO-**4**) had excitation and emission maxima 35–40 nm longer than AZO-**3**; however, the quantum yield of AZO-**4** was determined to be less than 1%, even lower than AZO-**3**'s quantum yield (2%).

**Fig. 2:**
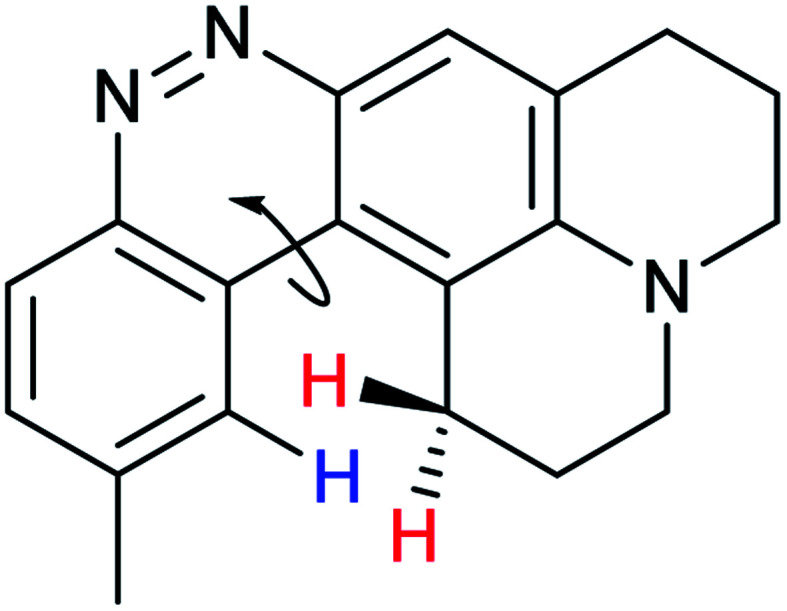
Disruption of planarity in AZO-**6** due to A^1,3^ strain.

When considering AZO-**3** and AZO_550_'s low quantum yield, we speculated that the excitation energy was lost to non-radiative relaxation through rotation about the ring A aryl-NMe_2_ bond.^88,89^ This bond rotation decreases significantly when the *N*-alkyl groups are fused to the aryl ring, so we synthesized *N*-methyl-tetrahydroquinoline (**5**) and julolidine (**6**) variants on ring A, with methyl at Y on ring B. The ring fusion in both variants resulted in a remarkable increase in quantum yield, from 0.02 and 0.11 for non-fused AZO-**3** and AZO_550_ to 0.36 for AZO-**6** and 0.62 for AZO-**5**. In addition, the molar absorptivity for AZO-**5** also increased, making it the brightest of the biphenyl series – nearly twenty times brighter than AZO_550_, albeit at shorter wavelengths. The julolidine variant did produce the longest fluorescence wavelengths of the group, confirming the expected greater planarity of the dialkyl-*N* to aryl bond. However, a steric clash between the aromatic proton and the julolidine benzylic protons, as depicted in Fig. 2, disrupts the planarity of the entire fluorophore, possibly explaining AZO-**6**'s lower quantum yield and lower molar absorptivity than AZO-**5**.

Probe **5** proved to be the brightest candidate, but AZO-**5**'s excitation and emission maxima (425 and 508 nm) fall short of the ideal wavelengths for the universal FITC filters. We decided to add ring C with the hypothesis that benzannulation would extend fluorescence wavelengths to those more suitable for the FITC filter set. For a thorough comparison, we studied the *N*,*N*-dimethylamino, *N*-methyltetrahydroquinoline, and julolidine variants at position X on ring A. For each of these variants, H occupied position Y and we derivatized position Z with H, *N*-acetamide (NHAc), and CN – a total of nine variants.

Focusing on brightness first, the results listed in Table 1 (probes **7–15**) show that both NMe_2_ (at X) and CN (at Z) substitutions are detrimental to cinnoline brightness. For the same Z substituent, the NMe_2_ series members had lower molar absorptivities and quantum yields than their corresponding *N*-methyl tetrahydroquinoline or julolidine congeners, *i.e.*, cinnolines **7***versus***10** and **13**, **8***versus***11** and **14**, and **9***versus***12** and **15**. This trend is readily explained by the greater non-radiative relaxation rate caused by rotation about the *N*-aryl bond for NMe_2_ variants *versus* the singly fused *N*-methyl tetrahydroquinoline and doubly fused julolidine variants. Likewise, the Z = CN cinnoline series exhibited lower molar absorptivities than the corresponding Z = NHAc or H series (for the same X substituent). We speculate that the Franck–Condon excited state has significant quinoid character in the Z = CN series, with less quinoid character in the ground state, such that the poor orbital overlap between the two states results in lower probability of photon absorption.

Within the Z = CN series, for the two alicyclic amine variants at X (AZO-**12** and AZO-**15**), the ground state incorporates greater donation of the aligned nitrogen lone pair into the polyaromatic system than from the more freely-rotating X = NMe_2_ variant (AZO_550_), leading to better orbital overlap of the ground state with the excited state, as demonstrated by their higher absorptivities. For the Z = H and NHAc variants, both substituents are less electron withdrawing than the quinoid-inducing CN, meaning that their ground and excited states are more similar and their absorptivities higher.^90,91^ The Z = CN series also produced lower quantum yields. Here we posit that the excited state relaxes through internal charge transfer to the nitrile π* orbital, with subsequent non-radiative relaxation through the increased hydrogen-bonding of this negatively-charged complex with buffer. *N*-methyl tetrahydroquinoline variants proved brighter than their A^1,3^-strained julolidine counterparts, as discussed for AZO-**6**. Interestingly, for AZO-**13** the greater electron-withdrawing nature of the NHAc Z-substituent (acceptor) acting upon the dialkyl amine (donor) lone pair electrons countered the A^1,3^ strain to promote greater planarity and therefore a higher quantum yield, as compared to Z = H for AZO-**14**.

As anticipated, adding ring C to the biphenyl probes bathochromically shifted fluorescence wavelengths. While benzannulation of fluorones to seminaphthofluorones shifts wavelengths by more than 100 nm, we found only 15–30 nm shift for the cinnolines.^92–94^ Substituting CN at Z (**9**, **12**, **15**) induced the greatest shift, but this substitution prohibitively decreased brightness. Variants substituted with NHAc at Z (**7**, **10**, **13**) showed only slight shifts (3–7 nm) to longer wavelengths compared to their corresponding H (**8**, **11**, **14**) substituted ones. Varying X in the benzannulated series from NMe_2_ (**7**, **8**, **9**) to *N*-methyltetrahydroquinoline (**10**, **11**, **12**) to julolidine (**13**, **14**, **15**) continued the aforementioned pattern in the biphenyl series of red-shifting fluorescence wavelengths with greater ring fusion. Benzannulated julolidine variants therefore produced the longest wavelength probes; unfortunately, julolidine substitution also decreased brightness, as proposed above, due to A^1,3^ strain.

The spectroscopic data reveal probes **5**, **10**, **11**, and **13** as the brightest in the group. Importantly, as with first generation NO_550_ and AZO_550_, the maximum excitation and emission spectra are well resolved from each other for both the probes and their cinnoline products, obviating homo-Förster resonance energy transfer (FRET) that occurs for high intracellular concentrations of *ortho*-diaminophenyl-type probes.^82^ Furthermore, in the case of the four brightest probes, the absorption spectra for the probes minimally overlap with the absorption spectra of their corresponding cinnolines (Fig. S1†). Consequently, as described below, in cells the probes can be excited independently from their N_2_O_3_ reaction products to compare intracellular distribution or compartmentalization of probe and cinnoline product, as well as to maintain a nearly zero background for detecting product (as previously reported for NO_550_).

### Fluorimetric NO titrations of probes

**Fig. 3:**
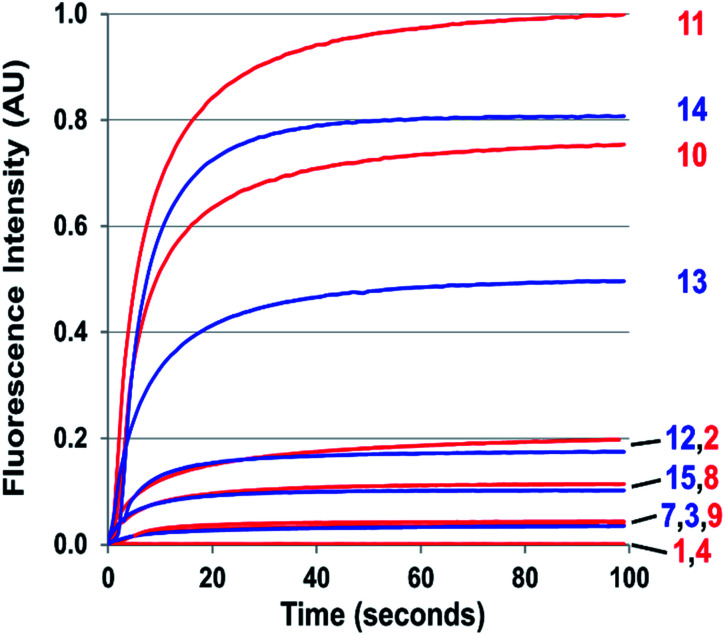
Kinetics of cinnoline emission emanating from exposure to one equivalent of NO of 50 μM solutions of **1–4** and **7–15** in 1 : 4 DMSO/phosphate buffer at pH 7.4 under aerobic conditions. Respective excitation wavelengths were set to the absorbance maximum of each probe's cinnoline product (AZO **1–4** and AZO **7–15**).

We next looked at fluorescence titrations with NO of the probes in aerobic buffer. Incremental aliquots of a saturated NO solution (1.9 mM),^15,95,96^ up to five equivalents of NO, were added to an air-equilibrated 50 μM solution of each probe variant in 4 : 1 50 mM PBS/DMSO at pH 7.4 (ESI Fig. S2†). Two equivalents of NO react with a half-equivalent of molecular oxygen to form one equivalent of N_2_O_3_ (Scheme 1), a portion of which is degraded to nitrite *via* hydrolysis.^23^ To maximize signal, probes should exhibit sufficient nucleophilicity to successfully compete with an excess of water. We started at the biphenyl system with no ring C, with Y as Me, and with X varied as OMe (**1**), OH (**2**), and NMe_2_ (**3**), their Hammett *σ*^+^ substituent constants of −0.78, −0.92, and −1.7, respectively.^97^ Variant **1** did not produce sufficient cinnoline product to be detected by fluorescence (Fig. 3), whereas **2** and **3** provided ample signal, confirming that X must be sufficiently electron-donating (*σ*^+^ < −0.78) to readily promote nucleophilic attack by ring A. Subsequently, we set X as NMe_2_ and changed the weakly donating methyl (*σ*^+^ = −0.311) at Y to a strongly-withdrawing cyano group, (*σ*^+^ = 0.659).^98^ With no cinnoline fluorescence detected, we concluded that the nitrile group inhibits the nucleophilicity of the *p*-amine to such an extent that it could not *N*-nitrosate.

The probes that did form cinnolines from N_2_O_3_ (**2–3**, **5–15**) for the most part required less than five minutes to nearly fully react. The high level of fluorescence from species **10**, **11**, **13**, and **14** makes these species the most promising of the group we examined (Fig. 3 and ESI Fig. S2†). More than two equivalents of NO, typically four in most cases, are necessary to reach saturation, however. We postulate that at the starting concentration of probe (50 μM, compared to 55.5 M for water), hydrolysis of N_2_O_3_ competes sufficiently as a side-reaction to warrant the excess NO.^99^ Despite requiring at least twice the theoretical amount of NO for full conversion, these probes surpass DAF-2, the predecessor of DAF-FM in reactivity. The fluorescence intensity of a solution of **3** exposed to two equivalents of NO, when calibrated to varying concentration standards of AZO-**3**, showed 36% cinnoline formation. In comparison, 27% of DAF-2 converted to the triazole when exposed to 2 equivalents of NO under similar conditions.^79^

With the high reactivity of these probes and with the creation of signal with virtually no background, excellent detection limits were obtained. We defined the lower detection limit (LDL) as the concentration of NO that produces signal equal to three times the experimental setup noise. The LDL of **9** (NO_550_) was previously determined to be 30 nM;^85^ for the brighter **10**, the detection limit is 2 nM, comparable to DAF-FM's detection limit of 3 nM.^76^

**Fig. 4:**
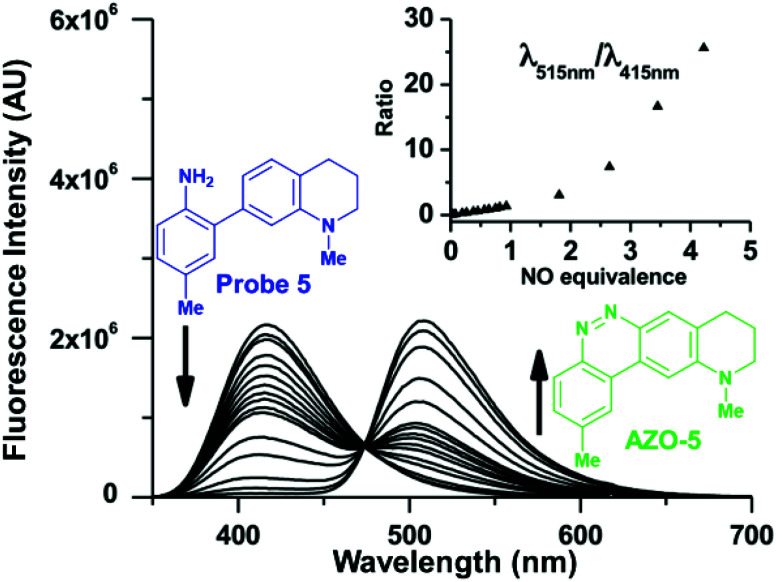
Fluorescence emission spectra depicting the ratiometric response to NO of a 50 μM probe **5** solution in 1 : 4 DMSO/50 mM phosphate buffer at pH 7.4 with excitation at 310 nm. The inset plots the ratio of emission intensity of AZO-**5** (515 nm) to that of probe **5** (415 nm) *versus* equivalents of NO.

The resolution of the probe and cinnoline emission spectra enables ratiometry when the probes and their cinnoline products share an excitation band (Fig. 4). Ratiometry corrects for several experimental factors, including variations in intracellular dye concentrations, in optical path due to cell thickness, and in instrumental noise.^100^ However, with our probes two factors moderate this benefit of ratiometry. First, the probe excitation band is in a shorter, cell-damaging near-UV range than that for the cinnoline products. Second, the shorter excitation results in little cinnoline emission, instead primarily unreacted probe background fluorescence. The later phenomenon however does not affect one of the most significant attributes of our 2-aminobiphenyl system: *i.e.* that exciting at the cinnoline absorbance maximum results in virtually no background contribution from the probe.

**Fig. 5:**
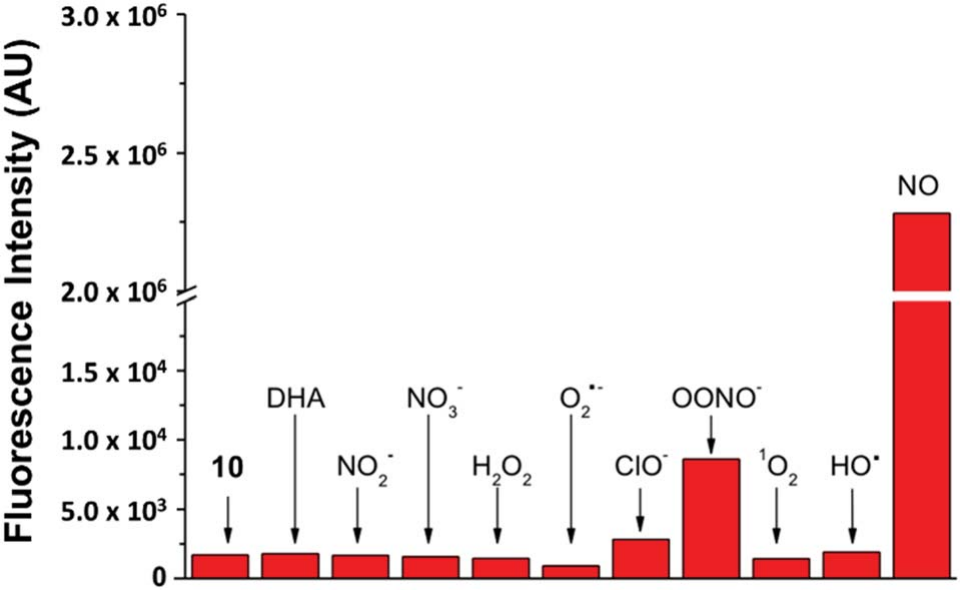
Probe **10**'s selective response to various physiological analytes in 100-fold excess, as compared to its response to one equivalent of NO (last column). The first column quantifies emission from **10** alone. Note the break/change in the vertical axis.

By keeping the 2-aminobiphenyl core, we expected to maintain in our newer probes the selectivity of NO_550_ for NO over other common biological substrates.^85^ Indeed this anticipation proved correct; as one example, Fig. 5 demonstrates fluorescence emission for probe **10** in the presence of NO *versus* that in the presence of various oxidants. Even at one hundred equivalents of peroxynitrite to probe, at most a five-fold increase of fluorescence was observed, as compared with a greater than 1300-fold increase for merely one equivalent of NO. It is important to note that we used purified peroxynitrite for this study. When this is generated *in cellulo* it involves both super oxide and NO which could potentially activate the fluorescent probe through a free radical mechanism.

### Probe performance in cells

Taking into consideration the probes' spectral properties and response to NO, we decided to focus on probes **10** (henceforth named NO_530_), and **13** (NO_562_) and to compare them to the first-generation probe **9** (NO_550_) and commercial probe DAF-FM. In the current studies, we examined the response of these probes to NO in an NIH-3T3 murine fibroblast cell line and a RAW 264.7 murine macrophage cell line because, while NO_530_ and NO_562_ readily reacted with NO in solution, most important is their performance in live cells. We first pre-incubated cells with the probes and then exposed cells to either exogenous NO-donor *S*-nitrosopenicillamine (SNAP) or to a NO solution. Next, we compared the specificity of NO_530_, NO_562_, and DAF-FM probes for the detection of endogenous NO produced in RAW 264.7 cells. Finally, we evaluated the cytotoxicity of NO_530_.

**Fig. 6:**
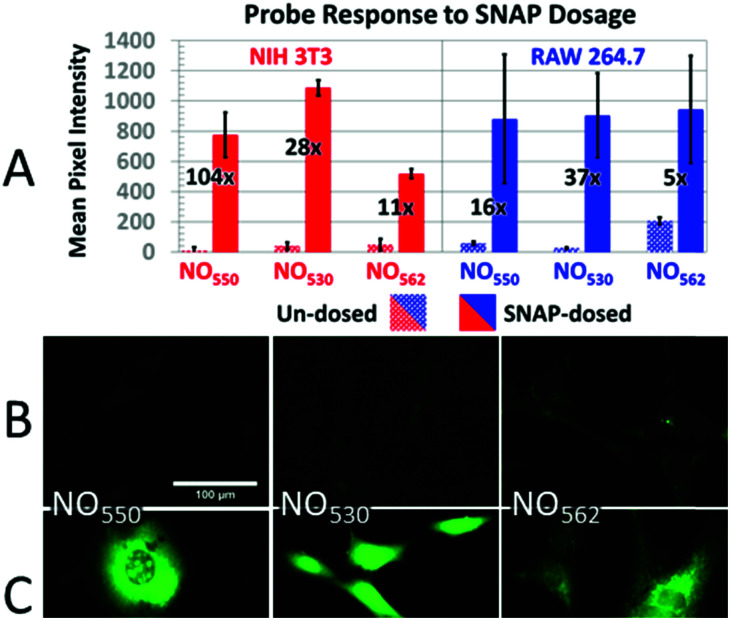
Response of probes to SNAP. (A) Response of control (dotted bars) *versus* SNAP-dosed (solid bars) NO_550_, NO_530_, and NO_562_ in NIH-3T3 (with 1 mM SNAP) and RAW 264.7 (with 200 μM SNAP) cells. The relative increase in intensity for cells dosed with SNAP *versus* un-dosed cells is provided as a fold increase. (B and C) 40× magnification, pseudo-colored FITC-filtered images of NIH-3T3 cells with no SNAP added (B), and with 1 mM SNAP added (C) for NO_550_, NO_530_, and NO_562_.

NIH-3T3 and RAW 264.7 cells were pre-loaded with 10 μM solutions of NO_550_, NO_530_, and NO_562_, and exposed to 1 mM and 200 μM SNAP solutions, respectively (Fig. 6). Microscopic observations revealed cell-associated blue fluorescence, characteristic of the unreacted probes (ESI Fig. S3†). In both cell types, the unreacted NO_550_, NO_530_, and NO_562_ produced homogenous cytoplasmic staining visible in the blue (DAPI) channel (ESI Fig. S4†). When exposed to SNAP, NO_550_, NO_530_, and NO_562_ converted to their respective cinnolines as demonstrated by increased green fluorescence signal in the FITC channel corresponding to cinnoline emission spectra (ESI Fig. S3†). In SNAP-dosed cells compared to the untreated cells, NO_562_ produced the smallest signal increase among the three tested probes (*F*_dosed_/*F*_un-dosed_, Fig. 6A). NO_562_ also produced higher background than the two other probes and generated lower signal in SNAP-treated NIH-3T3 cells. The observed increased background fluorescence of NO_562_ in untreated cells could be explained by the use of FITC filter set, which has shorter than necessary window for excitation/emission and thus may result in a bleed-through of the signal from the unreacted probe (Table 1 and ESI Fig. S3†). However, we also observed high background of NO_562_ reaction with NO endogenously produced in RAW264.7 cells using an IN Cell Analyzer imaging system (INCA 2200, GE Healthcare) with appropriate Cy3 filters (data not shown). In contrast, NO_530_ generated somewhat more SNAP-dependent signal than NO_550_ in NIH-3T3 cells, although it provided a several-fold lower increase relative to unstimulated cells (28-fold *vs.* 104-fold). In RAW 264.7 cells, NO_530_ and NO_550_ stimulated cell fluorescence was not significantly different, although NO_530_ provided somewhat higher fractional increase in fluorescence as a result of lower background (Fig. 6A blue). It is worth noting here, hat the production of N_2_O_3_ is dependent on O_2_ concentration in cells, and that higher nitrosation levels will occur when cells are treated with higher than normal O_2_ levels.

**Fig. 7:**
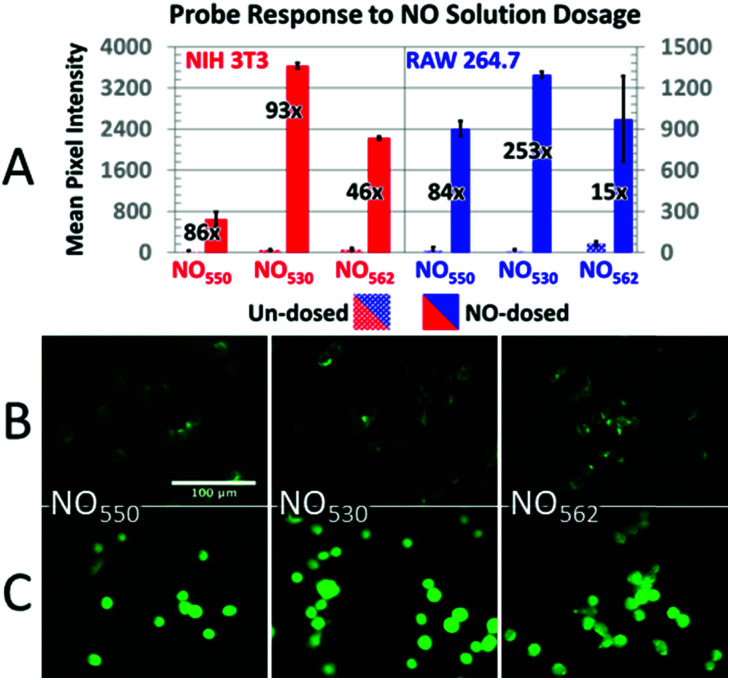
Response of probes to NO dosing. (A) Response of control (dotted bars) *versus* NO-dosed cells (solid bars) using NO_550_, NO_530_, and NO_562_ in both NIH-3T3 (with 200 μM NO) and RAW 264.7 (with 320 μM NO) cells. The relative increase in intensity for cells dosed with NO *versus* un-dosed cells is provided as a fold increase. (B and C) 40× magnification, pseudo-colored FITC-filtered images of NIH-3T3 cells without NO dosing (B), and with 320 μM NO added (C) for NO_550_, NO_530_, and NO_562_.

In addition, cells loaded with each probe were exposed to 200 μM NO (NIH-3T3 cells) or 320 μM NO (RAW 264.7 cells) by diluting 1.9 mM saturated NO solution into the cell medium (Fig. 7). In NIH-3T3 cells, NO_530_ produced the highest signal, while NO_550_ yielded the smallest change (although a similar fractional change in signal to NO_530_). All three probes yielded similar signal in RAW 264.7 cells; NO_530_ provided the best fractional change in signal because of substantially lower background before addition of NO. Taken together, these findings suggest that NO_530_ was the most promising probe to investigate endogenous production of NO in RAW 264.7 cells.

**Fig. 8:**
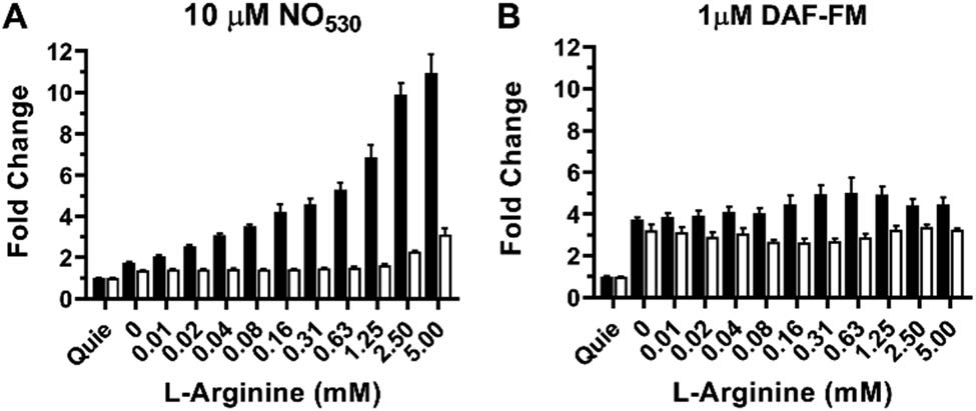
Detection of NO production in stimulated RAW 264.7 cells as a function of arginine concentration (open bars) using (A) NO_530_, and (B) DAF-FM. Black bars show results for cells inhibited with 2.5 mM NMMA; errors bars represent mean ± SD (*n* = 3). For all studies, cells were incubated with LPS and IFN-γ overnight. NMMA or control solutions were added to cells for 1 h and NO_530_ or DAF-FM were added during the final 15 min. l-Arginine was added for 30 min. Images were acquired using the INCA 2200 at 20× magnification and quantified using manufacturer's software. Quie = quiescent.

The RAW 264.7 cell line is known to express inducible NOS (iNOS), an enzyme that can be stimulated by a combination of lipopolysaccharide (LPS) and interferon-γ (IFN-γ) to produce NO in the presence of NO-synthase substrate l-arginine. We tested NO_530_ response in both quiescent and LPS/IFN-activated RAW 264.7 cells, and compared results to those obtained using DAF-FM DA. Data were obtained using cell imaging instrument INCA 2200. Cells were exposed to l-arginine in the presence of NO probes as described in the ESI.† In the presence of NO_530_, quiescent RAW 264.7 cells produced low fluorescence signal that did not measurably change after treatment with arginine. In the absence of exogenous arginine, LPS/IFN-activated RAW 264.7 cells produced approximately two-fold higher fluorescence signal than un-activated cells; cellular fluorescence further increased in response to arginine in a concentration-dependent manner (Fig. 8A, black bars). Pre-incubation of RAW 264.7 cells with pan-NOS inhibitor L-NG-monomethyl arginine (L-NMMA) blocked most of the signal in cells loaded with NO_530_, indicating the high NO specificity of the signal (Fig. 8A, white bars). In contrast, DAF-FM strongly reacted with activated RAW 264.7 cells, but both cells and media produced high fluorescence signal, requiring two iterative PBS washes to remove media fluorescence. Nevertheless, no arginine-concentration-dependence of the cell-associated signal could be observed and the pre-treatment with L-NMMA had little effect on DAF-FM-mediated fluorescence (Fig. 8B). The current observation confirms previously reported observations that 4,5-diaminofluorescein-based probes are prone to non-specific interactions with reactive species present in cells and cell culture media, exhibiting low selectivity towards cellular NO at physiologically relevant conditions.^101,102^

Finally, we did not observe signs of cytotoxicity when using our family of probes, even after 18 hour of incubation. Since NO_530_ was the best performer in terms of fluorescence ratio in NO-dosing studies, we evaluated its cytotoxicity in RAW 264.7 cells at various concentrations with a Calcein Blue/propidium iodide viability assay. For 1.25, 2.5, 5, 10, 20, and 40 μM loading concentrations of NO_530_, greater than 93% of the cells stained a viable blue (ESI Fig. S5†), similar to 95% viability in controls where either only DMSO or no additional solution was added.

## Conclusions

The 2-aminobiphenyl core selectively creates a longer-wavelength fluorophore when it reacts with oxidized NO. We found that attaching electron-donating groups, primarily dialkyl amines, on the nucleophilic aryl group, and avoiding the conjugation of electron withdrawing groups to the 2-amino group, renders the probes sufficiently nucleophilic to readily scavenge NO in acellular media. Furthermore, fusion of the 4′-aminoalkyl groups to the nucleophilic aryl group increased both fluorescence wavelengths and the fluorescence quantum yield of the cinnoline product. Benzannulation of the 2-amino aryl group also provoked a bathochromic shift in fluorescence and opened a functionalizable position, removed from the non-alkylated amine, for attachment of groups with desired properties *via* an amide linkage. When considering the propensity of the probe to react with NO, the brightness of the cinnoline product, and its low cytoxocity, NO_530_ (**10**) stands as a promising candidate for live cell imaging. Due to its greater response to stimuli, higher selectivity for NO, and the ability to image both reacted and unreacted probe, NO_530_ provides a attractive option as a nitric oxide probe and a potentially valuable alternative to DAF-FM in various applications.

## Conflicts of interest

DYF and AVK are employees of Creative Scientist, Inc.

## Supplementary Material

SC-011-C9SC04304G-s001
