# Supplementary material for: 2-Amino-3′-dialkylaminobiphenyl-based fluorescent intracellular probes for nitric oxide surrogate N2O3
Source: Chem Sci. 2020 Jan 2;11(5):1394–403. doi: 10.1039/c9sc04304g (PMC8148321; doi:10.1039/c9sc04304g)
Supplement: SC-011-C9SC04304G-s001 [file SC-011-C9SC04304G-s001.pdf]

Electronic Supplementary Material (ESI) for Chemical Science.
This journal is © The Royal Society of Chemistry 2019

SUPPORTING INFORMATION

2-Amino-3'-dialkylaminobiphenyl-Based Fluorescent Intracellular Probes for Nitric Oxide Surrogate N_2O_3

Yanming Shen[‡||], P. Rogelio Escamilla[§||], Quanjuan Zhang[‡], Derek S. Hernandez[§], Cecil J. Howard[§], Xuhong Qian[†‡], Daria Y. Filonov[#], Alexander V. Kinev[#], Jason Shear[§*], Eric V. Anslyn[§*], and Youjun Yang[†‡*]

[†]State Key Laboratory of Bioreactor Engineering, [‡]Shanghai Key Laboratory of Chemical Biology, School of Pharmacy, East China University of Science and Technology, Meilong Road 130, Shanghai 200237, China

[§]Department of Chemistry, University of Texas at Austin, Austin, Texas

[#]Creative Scientist, Inc., Durham, NC 27709

* anslyn@austin.utexas.edu, youjunyang@ecust.edu.cn, and jshear@mail.utexas.edu.

Contents

S2 *Materials and Equipment*

S3 *General Methods*

S4-S11 *Synthetic Procedures and Characterizations*

S12 *Absorbance and Fluorescence Spectra of Probes 5, 10, 11, and 13 (Figure S1)*

S13 *Fluorescence Titrations (Figure S2)*

S14 *DAPI and FITC Filter Transmission Spectra (Figure S3)*

S15-S19 *Cellular Experiments*

S15 *Cell culturing, passaging, and plating*

S16 *NO probe loading from DAPI-filtered fluorescence images of NIH 3T3 and RAW 264.7 cells (Figure S4)*

S16-17 *SNAP applied to NIH 3T3 and RAW 264.7 cells*

S17 *Aq. NO solution applied to NIH 3T3 and RAW 264.7 cells*

S17-18 *LPS variants screen*

S18 *LPS applied to RAW 264.7 cells*

S18-S19 *Calcein Blue and Propidium Iodide viability assay on RAW 264.7 cells loaded with NO_{530} (**10**) (Figure S5)*

S20-S21 *Cell image processing with FIJI (Figures S6a and S6b)*

S22 *References*

S23-S78 *^1H, ^{13}C, and HRMS spectra of NO probes (Figures S7 – S117)*

Materials

Reagents purchased from Energy Chemical Ltd., Fisher Scientific, VWR, and Sigma-Aldrich were used without further purification. Solvents for extraction or chromatographic purification purposes, including: CH_2Cl_2, EtOAc, MeOH and petroleum ether were purchased from Titan Chemical and Fisher Scientific and used without further purification. Silica gel (300 mesh) was purchased from Jiangyou Silica Gel production Ltd and from Fisher Scientific. HyClone DMEM + 4.00 mM L-glutamine, +4500 mg/L glucose, + sodium pyruvate; L-15 Leibovitz Medium + 2.05 mM L-glutamine; and DPBS + 1 g/L D-glucose and 36 mg/L sodium pyruvate were supplied by Thermo Scientific. HBSS modified with 10 mM HEPES came from STEMCELL Technologies and Kreb's Ringer Bicarbonate Buffer with 1.8 g/L D-glucose from Zen-Bio. Fetal bovine serum (FBS) was purchased from Gibco via Life Technologies and bovine calf serum (BCS) from ATCC. 0.05% Trypsin-EDTA was supplied by Gibco via Life Technologies. SNAP was supplied by Molecular Probes via Life Technologies. Lipopolysaccharides from Escherichia coli 0111:B4, γ-irradiated, came from Sigma. Interferon-gamma (IFN-γ) was obtained from Peprotech (Cat # 200-02). 384-well black clear-bottom plates were from Greiner Bio-One (Cat # 781091). L-N^G-monomethyl Arginine acetate (L-NMMA) was from Cayman Chemicals (Cat # 10005031).

Equipment

All ^{1}H-NMR and ^{13}C-NMR spectra were acquired in either $CDCl_3$ or DMSO-d_6 on a Bruker AV-400 spectrometer or a Varian 400. Chemicals shifts are referenced to the residual solvent peaks and given in ppm. HRMS were acquired on a Micromass GCT spectrometer or Agilent. Absorption spectra were acquired on a Cary 100-Bio UV-vis spectrophotometer. Fluorescence excitation and emission spectra were acquired on a PTI-QM4 steady-state fluorimeter with a 75 W Xenon arc-lamp and a R928 photo multiplier tube (PMT). The excitation and emission slits were both set to 2 nm. All emission spectra are corrected with respect to the PMT sensitivity. Cell images were acquired on an inverted Zeiss Axiovert 135 fluorescence microscope equipped with an ORCA-Flash 2.8 scientific-grade complementary metal oxide semiconductor camera from Hamamatsu, which was controlled with HCImage Live software. DAPI, FITC, and TRITC filter sets were Zeiss 02, 09, and 43, respectively. A 10x objective (NeoFluar, 0.5 NA) and a 40x (0.95 NA) objective were used for imaging. IN Cell Analyzer 2200 (GE Healthcare) was used to obtain images of RAW264.7 cells producing endogenous NO (20x objective).

General Methods

Solutions of DHA, NaNO2, NaNO3, H2O2, O^{2-}, ClO^-, $ONOO^-$, $HO\cdot$, and $^1O2$ for interference studies were prepared according to literature procedures.[1]

NO-saturated DPBS: Nitric oxide is quite toxic and has a chlorine-like smell; it was produced and manipulated in a well-ventilated hood. A three-neck 100 mL round-bottom flask was charged with 3.45 g $NaNO_2$ (50 mmol) in 20 mL distilled water. A pressure-equalizing funnel was placed on the central neck and filled with 30 mL 6 M H_2SO_4 (180 mmol). A side neck was connected via needle and plastic tubing to the inlet of a bubbler filled with 10 M aqueous NaOH, and the bubbler outlet was connected with needle and tubing to a 10 mL round-bottom flask containing 7 mL of DPBS. Nitrogen gas, passed through layers of NaOH and DryRite, was bubbled into both sulfuric acid and sodium nitrite solutions for at least twenty minutes to sparge the whole system free of dissolved O_2 that can oxidize NO. The sulfuric acid solution was added dropwise to the sodium nitrite solution, resulting in a light aqua reaction mixture with a brown vapor directly above. Once the rate of gas evolution became strong, the nitrogen sparging inlet line was removed from the system. The dropwise rate of addition was controlled to produce approximately one bubble per second through the bubbler. Before the sodium nitrite solution had fully reacted (before the bubbling rate decreased significantly), the clear NO-saturated DPBS solution was removed from the system and the septum was wrapped with parafilm. It was stored at -20 °C, for a maximum of three days prior to use. The reaction and NaOH solutions, as well as tubing, needles, septa, glassware, and solutions were left open to the air in the hood overnight to allow all NO to dissipate.

NO Concentration Measurements: A saturated solution of NO in H2O (1.9 mM) was prepared by bubbling pure NO gas into oxygen-free water for 15 min. The solubility of NO in pure H2O is 1.9 mM, which is widely cited data and no reference is further provided herein. An aliquot of this NO stock solution was transferred with an air-tight microsyringe (Milipore product) into the probe solution in a septum capped fluorescence cuvette (Starna product). The final concentration of the NO in the solution is calculated with the following equation:

$$[NO]final = ([NO]stock \times Vstock)/(VProbe\ solution + Vstock)$$

Synthetic Procedures and characterizations

*General Procedures for Suzuki-Miyaura coupling reactions to synthesize probes **1-6**, **8-9**, **11-12**, **14-15**.*

Aryl bromide (1eq), boronic acid or boronate ester (1.1 eq), and Na_2CO_3 (1.1 eq) were dissolved in H_2O, EtOH, and benzene (v/v 3:3:10). The resulting mixture was deoxygenated by sparging with argon for 15 min before addition of $Pd(PPh_3)_4$ (0.01 eq). The reaction was heated to 80°C with constant stirring under argon for 8 h. After the mixture cooled to room temperature, it was diluted with CH_2Cl_2 and washed with water. The organic layer was collected, dried over $MgSO_4$, and concentrated under reduced pressure. The residue was purified via flash chromatography with a mixture of petroleum ether:EtOAc (typically 20:1 v/v).

***3'-Methoxy-5-methyl-[1,1'-biphenyl]-2-amine** (**1**) Commercially available 2-bromo-4-methylaniline (2.00 g, 10.87 mmol), 3-methoxyphenylboronic acid (1.82 g, 11.97 mmol), and Na_2CO_3 (3.46 g, 32.61 mmol) gave **1** as a white solid (1.37 g, 59%). ^{1}H-NMR (400 MHz, $CDCl_3$) δ 7.53 (t, 1H, J = 8.0 Hz), 7.27-7.25 (m, 2H), 7.20 (s, 1H), 7.17 (d, 1H, J = 8.4 Hz), 7.09 (d, 1H, J = 8.0 Hz), 6.83 (d, 1H, J = 8.0 Hz), 3.98 (s, 3H), 3.84 (s, 2H), 2.51 (s, 3H). ^{13}C-NMR (100 MHz, $CDCl_3$) δ 159.7, 141.1, 141.0, 130.6, 129.6, 128.9, 127.3, 127.2, 121.2, 115.7, 114.4, 112.6, 54.9, 20.2. ESI-MS calculated for $C_{14}H_{15}NO$ $[M]^+$ 213.1, found 213.1.*

***3'-Hydroxy-5-methyl-[1,1'-biphenyl]-2-amine** (**2**) 2-bromo-4-methylaniline (870 mg, 4.67 mmol), 3-hydroxyphenylboronic acid (700 mg, 5.15 mmol) and Na_2CO_3 (540 mg, 5.15 mmol) gave **2** as a white solid (670 mg, 73%). Probe **2** may also be prepared alternatively via demethylation of probe **1** with BBr_3. ^{1}H-NMR (400 MHz, DMSO-d_6) δ 9.41 (s, 1H), 7.22 (t, 1H, J = 8.0 Hz), 6.84 (d, 1H, J = 8.0 Hz), 6.80-6.78 (m, 3H), 6.71 (d, 1H, J = 8.0 Hz), 6.64 (d, 1H, J = 8.0 Hz), 4.50 (s, 2H), 2.17 (s, 3H). ^{13}C-NMR (100 MHz, DMSO-d_6) δ 157.5, 142.2, 141.0, 130.1, 129.6, 128.5, 125.9, 124.9, 119.2, 115.4, 115.3, 113.7, 20.0. ESI-MS calculated for $C_{13}H_{13}NO$ $[M]^+$ 199.1, found 199.1.*

***$N^{3'}$,$N^{3'}$,5-trimethyl-[1,1'-biphenyl]-2,3'-diamine** (**3**) 2-bromo-4-methylaniline (1.00 g, 5.43 mmol), 3-(N,N-dimethylamino)phenylboronic acid (DMAPBA, 0.98 g, 5.94 mmol), and Na_2CO_3 (1.73 g, 16.30 mmol) gave **3** as a white solid (1.02 g, 82%). ^{1}H-NMR (400 MHz, $CDCl_3$) δ 7.42 (t, 1H, J = 8.0 Hz), 7.13 (s, 1H), 7.08 (d, 1H, J = 8.0Hz), 6.93-6.91 (m, 2H), 6.84 (d, 1H, J = 8.0 Hz), 6.77 (d, 1H, J = 8.0 Hz), 3.77 (s, 2H), 3.08 (s, 6H), 2.41 (s, 3H). ^{13}C-NMR (100 MHz, $CDCl_3$) δ 150.9, 141.1, 140.5, 130.8, 129.4, 128.8, 128.6, 127.5, 117.3, 115.7, 113.3, 111.3, 40.6, 20.5. ESI-HRMS calculated for $C_{15}H_{19}N_2$ $[M+H]^+$ 227.1543, found 227.1546.*

***6-Amino-3'-(dimethylamino)-[1,1'-biphenyl]-3-carbonitrile** (**4**) 4-amino-3-bromobenzonitrile (2.05 mmol), 3-(N,N-dimethylamino)phenylboronic acid (DMAPBA, 2.25 mmol), and Na_2CO_3 (652 mg, 6.15 mmol) gave **4** as a white solid (382 mg, 78%). ^{1}H-NMR (400 MHz, $CDCl_3$) δ 7.41-7.38 (m, 2H), 7.32 (t, 1H, J = 8.0 Hz), 6.75 (d, 1H, J = 8.4 Hz), 6.72-6.70 (m, 2H), 6.67 (s, 1H), 4.31 (s, 2H), 2.99 (s, 6H). ^{13}C-NMR (100 MHz, $CDCl_3$) δ 151.2, 147.9, 138.1, 134.4, 132.5, 130.0, 128.5, 120.4, 116.6, 115.0, 112.7, 112.2, 100.3, 40.6. ESI-MS calculated for $C_{15}H_{15}N_3$ $[M]^+$ 237.1, found 237.1.*

***4-Methyl-2-(1-methyl-1,2,3,4-tetrahydroquinolin-7-yl)aniline** (**5**) 2-bromo-4-methylaniline (1.00 mmol), compound **23** (0.66 mmol), and Na_2CO_3 (276 mg, 2.60 mmol) gave **5** as a white solid (145 mg, 86%). ^{1}H-NMR (400 MHz, $CDCl_3$) δ 7.06-7.03 (m,*

2H), 6.98 (d, 1H, J = 8.0 Hz), 6.72 (s, 1H), 6.69 (d, 2H, J = 6.4 Hz), 3.75 (s, 2H), 3.29 (t, 2H, J = 5.6 Hz), 2.93 (s, 3H), 2.84 (t, 2H, J = 6.4 Hz), 2.32 (s, 3H), 2.09-2.02 (m, 2H). ^{13}C-NMR (100 MHz, $CDCl_3$) δ 147.0, 141.2, 138.5, 130.9, 129.2, 128.8, 128.7, 127.6, 121.8, 116.8, 115.7, 111.7, 51.4, 39.3, 27.7, 22.5, 20.6. ESI-HRMS calculated for $C_{17}H_{21}N_2$ $[M+H]^+$ 253.1699, found 253.1705.

***2-(1,2,3,5,6,7-Hexahydropyrido[3,2,1-ij]quinolin-8-yl)-4-methylaniline** (**6**) 2-bromo-4-methylaniline (500 mg, 2.70 mmol), compound **24** (899 mg, 3.00 mmol), and Na_2CO_3 (859mg, 8.10 mmol) gave **6** as a white solid (270 mg, 73%). ^{1}H-NMR (400 MHz, $CDCl_3$) δ 6.95 (dd, 1H, J = 8.0, 1.6 Hz), 6.86-6.84 (m, 2H), 6.66 (d, 1H, J = 8.0 Hz), 6.41 (d, 1H, J = 8.0 Hz), 3.44 (s, 2H), 3.18 (t, 2H, J = 5.6 Hz), 3.13 (t, 2H, J = 5.6 Hz), 2.83-2.79 (m, 2H), 2.49 (t, 2H, J = 6.8 Hz), 2.25 (s, 3H), 2.05-1.98 (m, 2H), 1.92-1.86 (m, 2H). ^{13}C-NMR (100 MHz, DMSO-d_6) δ 143.3, 141.2, 136.6, 130.6, 128.4, 128.3, 127.2, 127.0, 120.9, 120.4, 117.5, 115.0, 50.4, 50.0, 27.9, 25.3, 22.2, 22.1, 20.5. ESI-HRMS calculated for $C_{19}H_{23}N_2$ $[M+H]^+$ 279.1856, found 279.1858.*

***2-(3-(Dimethylamino)phenyl)naphthalen-1-amine** (**8**) 2-bromonaphthylamine (220 mg, 0.99 mmol), 3-(N,N-dimethylamino)phenylboronic acid (DMAPBA, 220 mg, 1.09 mmol), and Na_2CO_3 (316 mg, 2.99 mmol) gave **8** as a white solid (260 mg, 85%). ^{1}H-NMR (400 MHz, $CDCl_3$) δ 7.92-7.86 (m, 2H), 7.53-7.51 (m, 2H), 7.44-7.40 (m, 3H), 6.94-6.92 (m, 2H), 6.81 (d, 1H, J = 9.2 Hz), 4.42 (s, 2H), 3.04 (s, 6H). ^{13}C-NMR (100 MHz, $CDCl_3$) δ 151.1, 141.0, 138.6, 133.7, 129.7, 128.7, 128.6, 125.7, 125.3, 123.7, 123.2, 121.3, 118.4, 117.8, 113.7, 111.4, 40.7. ESI-HRMS calculated for $C_{18}H_{19}N_2$ $[M+H]^+$ 263.1543, found 263.1548.*

***2-(1-Methyl-1,2,3,4-tetrahydroquinolin-7-yl)naphthalen-1-amine** (**11**) 2-bromonaphthylamine (359 mg, 1.62 mmol), compound **23** (300 mg, 1.01 mmol), and Na_2CO_3 (424 mg, 4.00 mmol) gave **11** as a white solid (299 mg, 64%). ^{1}H-NMR (400 MHz, $CDCl_3$) δ 7.89 (d, 1H, J = 7.2 Hz), 7.83 (d, 1H, J = 6.4Hz), 7.49-7.47 (m, 2H), 7.37 (s, 2H), 7.09 (d, 1H, J = 7.2 Hz), 6.77 (d, 1H, J = 7.2 Hz), 6.74 (s, 1H), 4.41 (s, 2H), 3.30 (t, 2H, J = 5.6 Hz), 2.93 (s, 3H), 2.86 (t, 2H, J = 6.4 Hz), 2.09-2.03 (m, 2H). ^{13}C-NMR (100 MHz, $CDCl_3$) δ 147.1, 138.9, 138.6, 133.6, 129.4, 128.8, 128.6, 125.7, 125.2, 123.8, 123.3, 121.9, 121.3, 118.4, 117.4, 112.1, 51.4, 39.3, 27.2, 22.6. EI-HRMS calculated for $C_{20}H_{20}N_2$ $[M]^+$ 288.1626, found 288.1627.*

***5-Amino-6-(1-methyl-1,2,3,4-tetrahydroquinolin-7-yl)-1-naphthonitrile** (**12**) 2-bromo-5-amino-1-naphthonitrile (100 mg, 0.41 mmol), compound **23** (122 mg, 0.45 mmol), and Na_2CO_3 (129 mg, 1.22 mmol) gave **12** as a white solid (70 mg, 55%). ^{1}H-NMR (400 MHz, $CDCl_3$) δ 8.12 (d, 1H, J = 8.4 Hz), 7.87 (d, 1H, J = 6.8 Hz), 7.72 (d, 1H, J = 8.4 Hz), 7.54 (d, 1H, J = 8.4 Hz), 7.48 (t, 1H, J = 8.0 Hz), 7.07 (d, 1H, J = 7.2 Hz), 6.71 (d, 1H, J = 7.2 Hz), 6.67 (s, 1H), 4.50 (s, 2H), 3.29 (t, 2H, J = 3.6 Hz), 2.92 (s, 3H), 2.84 (t, 2H, J = 6.0 Hz), 2.06-2.03 (m, 2H). ^{13}C-NMR (100 MHz, $CDCl_3$) δ 147.3, 139.6, 137.9, 132.7, 132.3, 131.6, 129.5, 126.8, 125.0, 123.9, 123.4, 122.4, 118.4, 117.0, 115.2, 111.7, 110.5, 51.4, 39.3, 27.8, 22.5. ESI-HRMS: calculated for $C_{21}H_{20}N_3$ $[M+H]^+$ 314.1652, found 314.1657.*

***2-(1,2,3,5,6,7-Hexahydropyrido[3,2,1-ij]quinolin-8-yl)naphthalen-1-amine** (**14**) 2-bromonaphthylamine (204 mg, 0.92 mmol), compound **24** (330 mg, 1.10 mmol), and Na_2CO_3 (293 mg, 2.76 mmol) gave **14** as a white solid (217 mg, 75%). ^{1}H-NMR (400 MHz, $CDCl_3$) δ 7.81-7.76 (m, 2H), 7.42-7.40 (m, 2H), 7.29 (d, 1H, J = 8.0 Hz), 7.17 (d, 1H, J = 8.0 Hz), 6.87 (d, 1H, J = 7.6 Hz), 6.47 (d, 1H, J = 7.6 Hz), 4.06 (s, 1H), 3.16 (t, 2H, J = 5.6 Hz), 3.10 (t, 2H, J = 5.6 Hz), 2.81-2.79 (m, 2H), 2.45 (t, 2H, J = 5.6 Hz), 2.03-*

1.97 (m, 2H), 1.86-1.80 (m, 2H). ^{13}C-NMR (100 MHz, $CDCl_3$) δ 143.5, 138.6, 136.8, 133.6, 128.6, 128.5, 127.2, 125.4, 124.9, 123.5, 122.6, 121.2, 121.0, 120.8, 118.0, 117.7, 50.3, 50.0, 27.9, 25.4, 22.2, 22.1. EI-HRMS calculated for $C_{22}H_{22}N_2$ $[M]^+$ 314.1783, found 314.1789.

5-Amino-6-(1,2,3,5,6,7-hexahydropyrido[3,2,1-ij]quinolin-8-yl)-1-naphthonitrile *(**15**) 2-bromo-5-amino-1-naphthonitrile (270 mg, 1.10 mmol), compound **24** (361 mg, 1.21 mmol), and Na_2CO_3 (350 mg, 3.30 mmol) gave **15** as a white solid (298 mg, 80%). ^{1}H-NMR (400 MHz, $CDCl_3$) δ 8.10 (d, 1H, J = 8.4 Hz), 7.87 (d, 1H, J = 7.6 Hz), 7.71 (d, 1H, J = 8.4 Hz), 7.47 (t, 1H, J = 8.4, 7.6 Hz), 7.40 (d, 1H, J = 8.4 Hz), 6.91 (d, 1H, J = 7.6 Hz), 6.46 (d, 1H, J = 7.6 Hz), 4.22 (s, 2H), 3.22 (t, 2H, J = 5.6 Hz), 3.16 (t, 2H, 5.6 Hz), 2.86-2.82 (m, 2H), 2.46-2.42 (m, 2H), 2.07-2.01 (m, 2H), 1.91-1.85 (m, 2H). ^{13}C-NMR (100 MHz, $CDCl_3$) δ 143.7, 139.7, 135.8, 132.8, 132.3, 131.5, 127.5, 126.7, 124.4, 123.7, 123.2, 121.5, 120.5, 118.4, 117.4, 115.0, 110.6, 50.4, 50.1, 28.1, 25.5, 22.2, 22.1. ESI-HRMS calculated for $C_{23}H_{22}N_3$ $[M+H]^+$ 340.1808, found 340.1811.*

*General Procedure for preparations of probes **7**, **10** and **13**.*

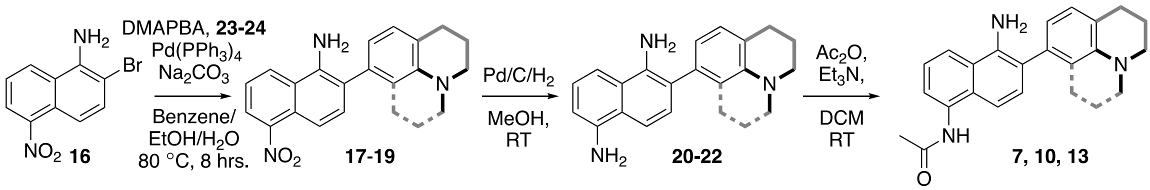

2-Bromo-5-nitronaphthalen-1-amine *(**16**) To a solution of 5-nitronaphthalen-1-amine[43] (3.5 g , 18.86 mmol) in 20 mL CH_2Cl_2 was added N-bromosuccinimide (3.6 g , 20.22 mmol) slowly at room temperature. The reaction mixture was stirred for 3 hours before removing solvent under reduced pressure. Crude product was purified by a silica gel chromatography as a white solid (4.5 g, 90%). ^{1}HNMR (400 MHz, $CDCl_3$) δ 8.11 (d, 1H, J = 8.0 Hz), 8.04 (d, 1H, J = 8.0 Hz), 7.73 (d, 1H, J = 9.2 Hz), 7.64 (d, 1H, J = 9.2 Hz), 7.47 (t, 1H, J = 9.2 Hz), 4.74 (s, 2H). ^{13}CNMR (100 MHz, $CDCl_3$) δ 147.5, 140.3, 133.3, 127.1, 125.1, 124.8, 123.9, 123.8, 113.8, 106.0. MS (EI) calculated for $C_{10}H_7BrN_2O_2$ $[M]^+$ 266.0, found 266.0.*

2-(3-(Dimethylamino)phenyl)-5-nitronaphthalen-1-amine *(**17**) Compound **16** (320 mg, 1.20 mmol) and 3-(N,N-dimethylamino)phenylboronic acid (DMAPBA, 200 mg, 1.00 mmol) were reacted according to the general Suzuki-Miyaura coupling procedure to give **17** as a white solid (270 mg, 88%). ^{1}HNMR (400 MHz, $CDCl_3$) δ 8.16 (d, 1H, J = 8.4 Hz), 8.11 (d, 1H, J = 7.6 Hz), 7.95 (d, 1H, J = 9.2 Hz), 7.55 (d, 1H, J = 9.2 Hz), 7.46 (t, 1H, J = 8.4, 7.6 Hz), 7.38 (t, 1H, J = 8.4, 7.6 Hz), 6.85-6.79 (m, 3H), 4.51 (s, 2H), 3.01 (s, 6H). ^{13}CNMR (100 MHz, $CDCl_3$) δ 151.1, 147.3, 139.7, 139.5, 132.2, 129.9, 127.6, 125.3, 124.9, 124.7, 123.4, 123.0, 117.3, 113.2, 112.7, 111.8, 40.6. EI-HRMS: calculated for $C_{18}H_{17}N_3O_2$ $[M]^+$ 307.1321, found 307.1320.*

2-(1-Methyl-1,2,3,4-tetrahydroquinolin-7-yl)-5-nitronaphthalen-1-amine *(**18**) Compound **16** (100 mg, 0.38 mmol) and compound **23** (103 mg, 0.38 mmol) were reacted according to the general Suzuki-Miyaura coupling procedure to give **18** as a white solid (80 mg, 64%). ^{1}HNMR (400 MHz, $CDCl_3$) δ 8.18 (d, 1H, J = 8.4 Hz), 8.13 (d, 1H, J = 7.6 Hz), 7.94 (d, 1H, J = 8.8 Hz), 7.55 (d, 1H, J = 8.8 Hz), 7.50 (t, 1H, J = 8.4, 7.6 Hz), 7.08 (d, 1H, J = 7.6 Hz), 6.71 (dd, 1H, J = 7.6, 1.2 Hz), 6.67 (d, 1H, J = 1.2 Hz), 4.50 (s,*

2H), 3.30 (t, 2H, J = 6.0 Hz), 2.92 (s, 3H), 2.84 (t, 2H, J = 6.8 Hz), 2.08-2.02 (m, 2H). ^{13}CNMR (100 MHz, $CDCl_3$) δ 147.5, 147.3, 139.4, 137.7, 132.3, 129.6, 127.6, 125.4, 125.1, 125.0, 123.3, 123.0, 122.5, 116.9, 112.9, 111.7, 51.4, 39.3, 27.8, 22.5. EI-HRMS: calculated for $C_{20}H_{19}N_3O_2$ $[M]^+$ 333.1477, found 333.1479.

***2-(1,2,3,5,6,7-Hexahydropyrido[3,2,1-ij]quinolin-8-yl)-5-nitronaphthalen-1-amine** (**19**) Compound **16** (2.0 g, 6.69 mmol) and compound **24** (1.5 g, 5.64 mmol) were reacted according to the general Suzuki-Miyaura coupling procedure to give **19** as a white solid (1.1 g 54%). ^{1}HNMR (400 MHz, $CDCl_3$) δ 8.16 (d, 1H, J = 8.4 Hz), 8.12 (d, 1H, J = 7.6 Hz), 7.93 (d, 1H, J = 8.8 Hz), 7.47 (t, 1H, J = 8.0 Hz), 7.42 (d, 1H, J = 8.8 Hz), 6.91 (d, 1H, J = 7.6 Hz), 6.46 (d, 1H, J = 7.6 Hz), 4.24 (s, 2H), 3.22 (t, 2H, J = 6.0 Hz), 3.16 (t, 2H, J = 5.6 Hz), 2.91-2.78 (m, 2H), 2.51-2.39 (m, 2H), 2.07-2.01 (m, 2H), 1.91-1.85 (m, 2H). ^{13}CNMR (100 MHz, $CDCl_3$) δ 147.4, 143.7,139.6, 135.6, 132.2, 127.5, 127.4, 125.3, 124.8, 124.3, 123.2, 122.8, 121.5, 120.4, 117.2, 112.4, 50.4, 50.0, 28.0, 25.4, 22.1, 22.0. ESI-MS: calculated for $C_{22}H_{21}N_3O_2$ $[M]^+$ 359.2, found 359.2.*

***2-(3-(Dimethylamino)phenyl)naphthalene-1,5-diamine** (**20**). A solution of compound **17** in 20mL MeOH:DCM (v/v 1:1) was stirred under hydrogen atmosphere (60 psi) with catalytic amount of 10% Pd/C for two hours. The reaction mixture was filtered through a celite pad, and solvents were removed under reduced pressure. Pure product was obtained in near quantitative yield after purification with silica gel flash chromatography. ^{1}HNMR (400 MHz, DMSO-d_6) δ 7.38 (d, 1H, J = 8.4 Hz), 7.32-7.28 (m, 2H), 7.14 (t, 1H, J = 8.0 Hz), 7.08 (d, 1H, J = 8.4 Hz), 6.76-6.72 (m, 3H), 6.65 (d, 1H, J = 7.2 Hz), 5.51 (s, 2H), 5.04 (s, 2H), 2.94 (s, 6H). ^{13}CNMR (100 MHz, DMSO-d_6) δ 151.3, 145.1, 141.5, 140.1, 129.8, 126.6, 125.6, 124.5, 123.2, 121.0, 117.4, 113.5, 111.4, 111.3, 110.7, 108.0, 40.6. ESI-HRMS: calculated for $C_{18}H_{19}N_3$ $[M]^+$ 277.1579, found 277.1577.*

***2-(1-Methyl-1,2,3,4-tetrahydroquinolin-7-yl)naphthalene-1,5-diamine** (**21**). Prepared analogously to compound **20** from **18**. ^{1}HNMR (400 MHz, DMSO-d_6) δ 7.35 (d, 1H, J = 8.8 Hz), 7.27 (d, 1H, J = 8.4 Hz), 7.12 (t, 1H, J = 8.4, 7.6 Hz), 7.05 (d, 1H, J = 8.8 Hz), 6.99 (d, 1H, J = 7.6 Hz), 6.62 (m, 3H), 5.48 (s, 2H), 5.00 (s, 2H), 3.23 (t, 2H, J = 5.6 Hz), 2.85 (s, 3H), 2.74 (t, 2H, J = 6.4 Hz), 1.96-1.90 (m, 2H). ^{13}CNMR (100 MHz, DMSO-d_6) δ 147.3, 145.0, 140.1, 139.4, 129.4, 126.6, 125.6, 124.5, 123.2, 121.3, 121.2, 117.0, 111.9, 111.3, 110.7, 108.0, 51.1, 39.2, 27.5, 22.4. ESI-HRMS: calculated for $C_{20}H_{21}N_3$ $[M]^+$ 303.1735, found 303.1737.*

***2-(1,2,3,5,6,7-Hexahydropyrido[3,2,1-ij]quinolin-8-yl)naphthalene-1,5-diamine** (**22**). Prepared analogously to compound **20** from **19**. ^{1}HNMR (400 MHz, DMSO-d_6) δ 7.31 (d, 1H, J = 8.0 Hz), 7.24 (d, 1H, J = 8.8 Hz), 7.10 (t, 1H, J = 8.0, 7.6 Hz), 6.86 (d, 1H, J = 8.8 Hz), 6.80 (d, 1H, J = 7.6 Hz), 6.62 (d, 1H, J = 8.0 Hz), 6.30 (d, 1H, J = 8.0 Hz), 5.46 (s, 2H), 4.64 (s, 2H), 3.15 (t, 2H, J = 5.6 Hz), 3.09 (t, 2H, J = 5.6 Hz), 2.73 (t, 2H, J = 6.4 Hz), 2.40-2.31 (m, 2H), 1.96-1.89 (m, 2H), 1.80-1.74 (m, 2H). ^{13}CNMR (100 MHz, DMSO-d_6) δ 144.6, 143.1, 139.6, 137.0, 126.7, 125.9, 124.9, 123.8, 122.7, 120.3, 120.0, 119.9, 117.1, 110.5, 110.1, 107.3, 49.6, 49.2, 27.3, 24.8, 21.6, 21.5. ESI-MS: calculated for $C_{22}H_{23}N_3$ $[M]^+$ 329.2, found 329.2.*

***N-(5-Amino-6-(3-(dimethylamino)phenyl)naphthalen-1-yl)acetamide** (**7**). To a solution of compound **20** (124 mg, 0.45 mmol) in 10 mL CH_2Cl_2 was added excess acetic anhydride (3 equiv) dropwise. The mixture was stirred at room temperature for 2 h before evaporating to dryness under reduced pressure. Pure product (270 mg, 55%) was obtained by a flash chromatography*

with silica gel. ^{1}H-NMR (400 MHz, DMSO-d_6) δ 9.77 (s, 1H), 7.99 (d, 1H, J = 8.4 Hz), 7.62 (d, 1H, J = 7.2 Hz), 7.40-7.37 (m, 2H), 7.31 (t, 1H, J = 8.0 Hz), 7.23 (d, 1H, J = 8.4 Hz), 6.76-6.74 (m, 3H), 5.31 (s, 2H), 2.94 (s, 6H), 2.17 (s, 3H). ^{13}C-NMR (100 MHz, DMSO-d_6) δ 169.2, 151.3, 141.0, 140.7, 134.1, 129.9, 128.7, 128.5, 124.2, 124.1, 121.9, 121.2, 120.4, 117.4, 113.4, 111.6, 111.4, 23.9. ESI-HRMS calculated for $C_{20}H_{21}N_3O$ $[M]^+$ 319.1685, found 319.1683.

***N-(5-amino-6-(1-methyl-1,2,3,4-tetrahydroquinolin-7-yl)naphthalen-1-yl)acetamide** (**10**). Prepared analogously to **7** from compound **21**. ^{1}H-NMR (400 MHz, DMSO-d_6) δ 9.77 (s, 1H), 7.97 (d, 1H, J = 8.4 Hz), 7.60 (d, 1H, J = 7.2 Hz), 7.40-7.36 (m, 2H), 7.21 (d, 1H, J = 8.8 Hz), 7.00 (d, 1H, J = 7.2 Hz), 6.62-6.60 (m, 2H), 5.28 (s, 2H), 3.23 (t, 2H, J = 5.6 Hz), 2.85 (s, 3H), 2.75 (t, 2H, J = 6.4 Hz), 2.16 (s, 3H), 1.96-1.90 (m, 2H). ^{13}C-NMR (100 MHz, DMSO-d_6) δ 169.3, 147.3, 140.6, 139.0, 134.0, 129.5, 128.8, 128.4, 124.2, 124.1, 121.8, 121.6, 121.4, 120.4, 117.0, 111.8, 111.4, 51.1, 39.2, 27.5, 23.9, 22.4. ESI-HRMS: calculated for $C_{22}H_{23}N_3O$ $[M]^+$ 345.1841, found 345.1843.*

***N-(5-amino-6-(1,2,3,5,6,7-hexahydropyrido[3,2,1-ij]quinolin-8-yl)naphthalen-1-yl)acetamide** (**13**). Prepared analogously to probe **7** from compound **22**. ^{1}H-NMR (400 MHz, DMSO-d_6) δ 9.74 (s, 1H), 7.94 (d, 1H, J = 8.4 Hz), 7.60 (d, 1H, J = 7.2 Hz), 7.38-7.34 (m, 2H), 7.04 (d, 1H, J = 8.8 Hz), 6.82 (d, 1H, J = 7.6 Hz), 6.31 (d, 1H, J = 7.6 Hz), 4.94 (s, 2H), 3.15 (t, 2H, J = 4.8 Hz), 3.12-3.05 (m, 2H), 2.74 (t, 2H, J = 6.4 Hz), 2.40-2.28 (m, 2H), 2.16 (s, 3H), 1.96-1.89 (m, 2H), 1.80-1.74 (m, 2H). ^{13}C-NMR (100 MHz, DMSO-d_6) δ 168.7, 143.1, 140.2, 136.5, 133.6, 128.1, 128.0, 126.8, 123.5, 123.4, 121.2, 120.5, 120.2, 119.8, 117.0, 110.5, 64.9, 49.6, 49.2, 27.4, 24.8, 23.4, 21.6, 21.5. ESI-HRMS: calculated for $C_{24}H_{26}N_3O$ $[M+H]^+$ 372.2070, found 372.2072.*

Synthesis of precursors for Suzuki couplings:

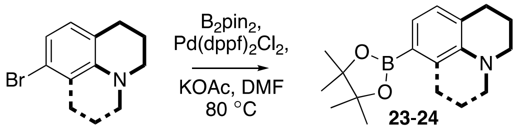

***1-Methyl-7-(4,4,5,5-tetramethyl-1,3,2-dioxaborolan-2-yl)-1,2,3,4-tetrahydroquinoline** (**23**). 7-Bromo-1-methyl-1,2,3,4–tetrahydroquinoline (710 mg，3.15 mmol), bis(pinacolato)diboron (962 mg，3.78 mmol) and KOAc (928 mg，9.47 mmol) were dissolved in anhydrous DMF (15 mL) and deoxygenated by sparging with argon for 15 min before adding $PdCl_2dppf$ (26 mg，0.03 mmol). The reaction mixture was heated at 80 °C under argon for 8 h. Upon cooling, the reaction mixture was diluted with 300 mL of H_2O and extracted with CH_2Cl_2. The organic layer was collected, dried over $MgSO_4$ and concentrated under reduced pressure to give a brown residue, which was purified by a flash chromatography to afford **23** as colorless oil (540 mg, 62%). ^{1}H-NMR (400 MHz, $CDCl_3$) δ 7.10 (d, 1H, J = 7.2 Hz), 7.05 (s, 1H), 6.98 (d, 1H, J = 7.2 Hz), 3.23 (t, 2H, J = 5.2 Hz), 2.95 (s, 3H), 2.79 (t, 2H, J = 6.4 Hz), 2.00-1.97 (m, 2H), 1.35 (s, 12H). ^{13}C-NMR (100 MHz, $CDCl_3$) δ 146.4, 128.4, 126.6, 123.2, 117.0, 83.5, 51.5, 39.4, 28.2, 24.9, 22.5. ESI-HRMS calculated for $C_{16}H_{25}BNO_2$ $[M+H]^+$ 274.1973, found 274.1971.*

***8-(4,4,5,5-Tetramethyl-1,3,2-dioxaborolan-2-yl)-1,2,3,5,6,7-hexahydropyrido[3,2,1-ij]quinolone** (**24**). Compound **24** was prepared analogously to compound **23**, with the aryl bromide as 8-bromo-1,2,3,5,6,7-hexahydropyrido[3,2,1-ij]quinolone. ^{1}H-*

NMR (400 MHz, $CDCl_3$) δ 7.28 (d, 1H, J = 7.2Hz), 7.01 (d, 1H, J = 7.2 Hz), 3.32-3.29 (m, 6H), 2.98 (t, 2H, J = 6.4 Hz), 2.21-2.16 (m, 4H), 1.54 (s, 12H). ^{13}C-NMR (100 MHz, $CDCl_3$) δ 142.8, 128.3, 126.1, 124.8, 123.9, 82.8, 50.4, 49.9, 28.1, 27.2, 24.7, 22.2, 22.0. ESI-HRMS calculated for $C_{18}H_{27}BNO_2$ $[M+H]^+$ 300.2129, found 300.2123.

General procedure for diazotization of probes to produce their cinnoline products:

*A solution of probe (**1-15**) in 1M HCl was cooled in an ice/brine bath to 0 °C. $NaNO_2$ (1.05 equivalents) in ice-cold H_2O was added dropwise while maintaining the reaction mixture below 0 °C. The mixture was stirred for 30 min before neutralizing with saturated sodium bicarbonate solution and extracting with CH_2Cl_2. The organic layer was dried over $MgSO_4$ and concentrated under reduced pressure to give crude product, which was purified by a flash chromatography with a mixture of CH_2Cl_2 and MeOH as the mobile phase.*

***2-Methoxy-9-methylbenzo[c]cinnoline** (from **1**) ^1H-NMR (400 MHz, DMSO-d_6) δ 8.72 (s, 1H), 8.53 (d, 1H, J = 9.2 Hz), 8.48 (d, 1H, J = 8.4 Hz), 8.16 (d, 1H, J = 2.4 Hz), 7.81 (d, 1H, J = 8.4 Hz), 7.56 (dd, 1H, J = 9.2, 2.4 Hz), 4.08 (s, 3H), 2.66 (s, 3H). ^{13}C-NMR (100 MHz, DMSO-d_6) δ 161.5, 143.5, 141.6, 141.4, 132.2, 131.5, 129.8, 122.5, 121.7, 120.7, 120.4, 101.5, 56.2, 21.7. ESI-MS calculated for $C_{14}H_{12}N_2O$ $[M]^+$ 224.1, found 224.1.*

***9-Methylbenzo[c]cinnolin-2-ol** (from **2**) ^1H-NMR (400 MHz, DMSO-d_6) δ 8.49-8.45 (m, 3H), 7.93 (s, 1H), 7.78 (d, 1H, J = 8.8 Hz), 7.47 (d, 1H, J = 8.8 Hz), 2.64 (s, 3H). ^{13}C-NMR (100 MHz, DMSO-d_6) δ 160.5, 143.2, 141.4, 141.0, 132.7, 131.4, 129.8, 122.7, 121.3, 120.7, 120.1, 103.8, 21.7. ESI-MS calculated for $C_{13}H_{10}N_2O$ $[M]^+$ 210.1, found 210.1.*

***N,N,9-trimethylbenzo[c]cinnolin-2-amine** (from **3**) ^1H-NMR (400 MHz, $CDCl_3$) δ 8.28 (d, 1H, J = 8.4 Hz), 8.20 (d, 1H, J = 9.2 Hz), 7.78 (s, 1H), 7.41 (d,1H, J = 8.4 Hz), 6.95 (d,1H, J = 9.2 Hz), 6.81 (s, 1H), 2.92 (s, 6H), 2.45 (s, 3H). ^{13}C-NMR (100 MHz, $CDCl_3$) δ 150.9, 143.7, 139.9, 139.9, 131.8, 130.3, 129.9, 122.8, 120.6, 120.3, 115.8, 97.3, 40.0, 22.0. ESI-HRMS calculated for $C_{15}H_{16}N_3$ $[M+H]^+$ 238.1339, found 238.1343.*

***9-(Dimethylamino)benzo[c]cinnoline-2-carbonitrile** (from **4**) ^1H-NMR (400 MHz, $CDCl_3$) δ 8.80 (s, 1H), 8.62 (d, 1H, J = 8.4 Hz), 8.51 (d, 1H, J = 9.2 Hz), 7.95 (d, 1H, J = 8.4 Hz), 7.39 (d, 1H, J = 9.2 Hz), 7.29 (s, 1H), 3.29 (s, 6H). ^{13}C-NMR (100 MHz, $CDCl_3$) δ 152.5, 145.2, 141.2, 133.3, 131.9, 130.1, 128.1, 122.4, 121.0, 118.8, 117.5, 112.8, 97.5, 40.7. ESI-MS calculated for $C_{15}H_{12}N_4$ $[M]^+$ 248.1, found 248.1.*

***2,11-Dimethyl-8,9,10,11-tetrahydrobenzo[c]pyrido[2,3-g]cinnoline** (from **5**) ^1H-NMR (400 MHz, $CDCl_3$) δ 8.41 (d, 1H, J = 8.4 Hz), 8.15 (s, 1H), 8.13 (s, 1H), 7.58 (d, 1H, J = 8.4 Hz), 7.18 (s, 1H), 3.50 (t, 2H, J = 5.6 Hz), 3.18 (s, 3H), 3.03 (t, 2H, J = 6.4 Hz), 2.64 (s, 3H), 2.10-2.04 (m, 2H). ^{13}C-NMR (100 MHz, $CDCl_3$) δ 148.9, 144.1, 140.8, 140.0, 130.4, 130.0, 127.8, 122.6, 121.0, 120.6, 96.0, 51.5, 39.4, 28.5, 22.4, 21.9. ESI-HRMS calculated for $C_{17}H_{18}N_3$ $[M+H]^+$ 264.1495, found 264.1499.*

***13-Methyl-1,2,3,5,6,7-hexahydrobenzo[c]quinolizino[1,9-fg]cinnoline** (from **6**) ^1H-NMR (400 MHz, $CDCl_3$) δ 8.41 (d, 1H, J = 8.4 Hz), 8.26 (s, 1H), 8.01 (s, 1H), 7.53 (d, 1H, J = 8.4 Hz), 3.40-3.37 (m, 6H), 2.98 (t, 2H, J = 6.4 Hz), 2.58 (s, 3H), 2.05-1.98 (m, 4H).*

^{13}C-NMR (100 MHz, $CDCl_3$) δ 145.7, 145.3, 140.7, 138.4, 130.4, 129.4, 129.3, 126.3, 125.8, 122.0, 120.8, 111.7, 51.1, 49.7, 29.4, 28.8, 22.7, 21.8, 21.3. ESI-HRMS calculated for $C_{19}H_{20}N_3$ [M+H]$^+$ 290.1652, found 290.1657.

***N-(9-(dimethylamino)dibenzo[c,h]cinnolin-1-yl)acetamide** (from **7**) ^{1}H-NMR (400 MHz, DMSO-d_6) δ 10.21 (s, 1H), 9.46 (d, 1H, J = 8.0 Hz), 8.76 (d, 1H, J = 9.2 Hz), 8.44 (d, 1H, J = 9.2 Hz), 8.37 (d, 1H, J = 9.2 Hz), 7.92 (d, 1H, J = 8.0 Hz), 7.85 (t, 1H, J = 8.0 Hz), 7.64-7.59 (m, 2H), 3.26 (s, 6H), 2.26 (s, 3H). ^{13}C-NMR (100 MHz, DMSO-d_6) δ 169.7, 152.2, 142.2, 141.3, 134.9, 132.0, 131.6, 128.1, 127.7, 126.2, 124.7, 123.7, 121.2, 120.1, 118.8, 118.5, 98.2, 49.1, 24.0. EI-HRMS calculated for $C_{20}H_{18}N_4O$ [M]$^+$ 330.1481, found 330.1479.*

***N,N-dimethyldibenzo[c,h]cinnolin-9-amine** (from **8**) ^{1}H-NMR (400 MHz, $CDCl_3$) δ 9.78 (d, 1H, J = 8.0 Hz), 8.53 (d, 1H, J = 9.2Hz), 8.35 (d, 1H, J = 8.8 Hz), 8.08 (d, 1H, J = 8.8 Hz), 7.98 (d, 1H, J = 8.0Hz), 7.85 (t, 1H, J = 8.0 Hz), 7.75 (t, 1H, J = 8.0 Hz), 7.39 (d, 1H, J = 9.2 Hz), 7.35 (s, 1H), 3.24 (s, 6H). ^{13}C-NMR (100 MHz, $CDCl_3$) δ 151.1, 142.2, 141.7, 133.0, 132.1, 131.2, 130.9, 128.0, 127.9, 127.9, 124.6, 123.9, 118.9, 118.7, 117.3, 97.2, 40.4. ESI-HRMS calculated for $C_{18}H_{16}N_3$ [M+H]$^+$ 274.1344, found 274.1346.*

***N-(11-methyl-8,9,10,11-tetrahydrobenzo[h]quinolino[6,7-c]cinnolin-1-yl)acetamide** (from **10**) ^{1}H-NMR (400 MHz, DMSO-d_6) δ 10.91 (s, 1H), 9.42 (d, 1H, J = 8.0 Hz), 8.70 (d, 1H, J = 9.2 Hz), 8.32 (d, 1H, J = 9.2 Hz), 8.12 (s, 1H), 7.89 (d, 1H, J = 8.0 Hz), 7.82 (t, 1H, J = 8.0 Hz), 7.47 (s, 1H), 3.52 (t, 2H, J = 6.0 Hz), 3.22 (s, 3H), 3.04 (t, 2H, J = 6.0 Hz), 2.24 (s, 3H), 2.00-1.98 (m, 2H). ^{13}C-NMR (100 MHz, DMSO-d_6) δ 169.6, 149.5, 142.6, 141.2, 134.8, 131.6, 130.0, 129.1, 128.0, 125.9, 124.6, 123.1, 121.1, 120.2, 118.2, 96.1, 51.1, 49.1, 28.3, 24.0, 21.4. EI-HRMS calculated for $C_{22}H_{20}N_4O$ [M]$^+$ 356.1637, found 356.1639.*

***11-Methyl-8,9,10,11-tetrahydrobenzo[h]quinolino[6,7-c]cinnoline** (from **11**). ^{1}H-NMR (400 MHz, $CDCl_3$) δ 9.74 (d,1H, J=8.0 Hz), 8.24 (d, 1H, J = 8.8 Hz), 8.13 (s, 1H), 8.00 (d, 1H, J = 8.8 Hz), 7.95 (d, 1H, J = 8.0 Hz), 7.82 (t, 1H, J = 7.6 Hz), 7.71 (t, 1H, J = 7.2 Hz), 7.09 (s, 1H), 3.45 (t, 2H, J = 5.6 Hz), 3.11 (s, 3H), 3.00 (t, 2H, J = 6.0 Hz), 2.07-2.01 (m, 2H). ^{13}C-NMR (100 MHz, $CDCl_3$) δ 148.8, 142.7, 141.7, 132.9, 131.4, 130.7, 129.6, 128.9, 127.9, 127.9, 124.6, 123.4, 119.1, 118.7, 95.4, 51.4, 39.3, 28.6, 21.8. ESI-HRMS calculated for $C_{20}H_{18}N_3$ [M + H]$^+$ 300.1495, found 300.1500.*

***11-Methyl-8,9,10,11-tetrahydrobenzo[h]quinolino[6,7-c]cinnoline-1-carbonitrile** (from **12**). ^{1}H-NMR (400 MHz, $CDCl_3$) δ 9.91 (d, 1H, J = 8.4 Hz), 8.27 (d, 1H, J = 8.8 Hz), 8.20 (d, 1H, J = 8.8 Hz), 8.08 (s, 1H), 8.02(d, 1H, J = 7.2 Hz), 7.80 (t, 1H, J = 8.4, 7.2 Hz), 6.95 (s, 1H), 3.48 (t, 2H, J = 5.2 Hz), 3.11 (s, 3H), 3.00 (t, 2H, J = 5.6 Hz), 2.06-2.04 (m, 2H). ^{13}C-NMR (100 MHz, $CDCl_3$) δ 149.1, 142.9, 140.6, 133.4, 132.3, 131.3, 129.8, 129.6, 129.6, 127.0, 126.3, 122.9, 122.2, 118.8, 117.9, 110.0, 95.2, 51.4, 39.4, 28.5, 21.5. ESI-HRMS calculated for $C_{21}H_{17}N_4$ [M+H]$^+$ 325.1448, found 325.1455.*

***N-(8,9,10,12,13,14-hexahydronaphtho[1,2-c]quinolizino[1,9-fg]cinnolin-1-yl)acetamide** (from **13**). ^{1}H-NMR (400 MHz, DMSO-d_6) δ 10.14 (s, 1H), 9.45 (d, 1H, J = 8.0 Hz), 8.60 (d, 1H, J = 9.6 Hz), 8.22 (d, 1H, J = 9.6 Hz), 8.02 (s, 1H), 7.88 (d, 1H, J = 7.6 Hz), 7.80 (t, 1H, J = 8.0, 7.6 Hz), 3.48-3.43 (m, 6H), 3.03 (t, 2H, J = 6.4 Hz), 2.23 (s, 3H), 1.98 (m, 4H). ^{13}C-NMR (100 MHz, DMSO-d_6) δ 169.1, 145.9, 141.9, 141.8, 134.0, 131.2, 128.2, 128.0, 127.2, 126.6, 123.6, 121.1, 121.0, 118.3, 109.8, 50.4, 49.1, 28.6, 28.2, 23.4, 21.0, 20.5. ESI-HRMS calculated for $C_{24}H_{23}N_4O$ [M+H]$^+$ 383.1866, found 383.1872.*

8,9,10,12,13,14-Hexahydronaphtho[1,2-c]quinolizino[1,9-fg]cinnoline *(from **14**).* *^{1}H-NMR (400 MHz, $CDCl_3$) δ 9.75 (d, 1H, J = 8.4 Hz), 8.36 (d, 1H, J = 9.6 Hz), 8.00 (s, 1H), 7.88 (d, 1H, J = 8.4 Hz), 7.84 (d, 1H, J = 9.6 Hz), 7.78 (t, 1H, J = 8.4 Hz), 7.68 (t, 1H, J = 8.4 Hz), 3.35-3.30 (m, 6H), 2.96 (t, 2H, J = 6.0 Hz), 1.99-1.96 (m, 4H). ^{13}C-NMR (100 MHz, $CDCl_3$) δ 145.6, 142.7, 142.5, 132.1, 131.2, 128.8, 128.4, 127.7, 127.4, 127.2, 124.9, 123.8, 122.0, 119.3, 110.1, 51.0, 49.7, 29.4, 28.9, 21.6, 21.1. ESI-HRMS calculated for $C_{22}H_{20}N_3$ $[M+H]^+$ 326.1652, found 326.1660.*

8,9,10,12,13,14-Hexahydronaphtho[1,2-c]quinolizino[1,9-fg]cinnoline-1-carbonitrile *(from **15**).* *^{1}H-NMR (400 MHz, $CDCl_3$) δ 9.96 (d, 1H, J = 8.4 Hz), 8.55 (d, 1H, J = 9.2 Hz), 8.16 (d, 1H, J = 9.2 Hz), 8.04-8.02 (m, 2H), 7.78 (t, 1H, J = 8.0 Hz), 3.44-3.34 (m, 6H), 3.01 (t, 2H, J = 6.0 Hz), 2.06-2.02 (m, 4H). ^{13}C-NMR (100 MHz, $CDCl_3$) δ 146.3, 142.8, 141.9, 133.5, 131.6, 131.3, 130.3, 129.1, 128.4, 126.9, 126.6, 124.4, 121.5, 119.7, 118.0, 110.1, 109.5, 51.2, 49.9, 29.4, 29.0, 21.5, 21.1. ESI-HRMS calculated for $C_{23}H_{19}N_4$ $[M+H]^+$ 351.1604, found 351.1608.*

Absorbance and Fluorescence Spectra of Probes 5, 10, 11, and 13

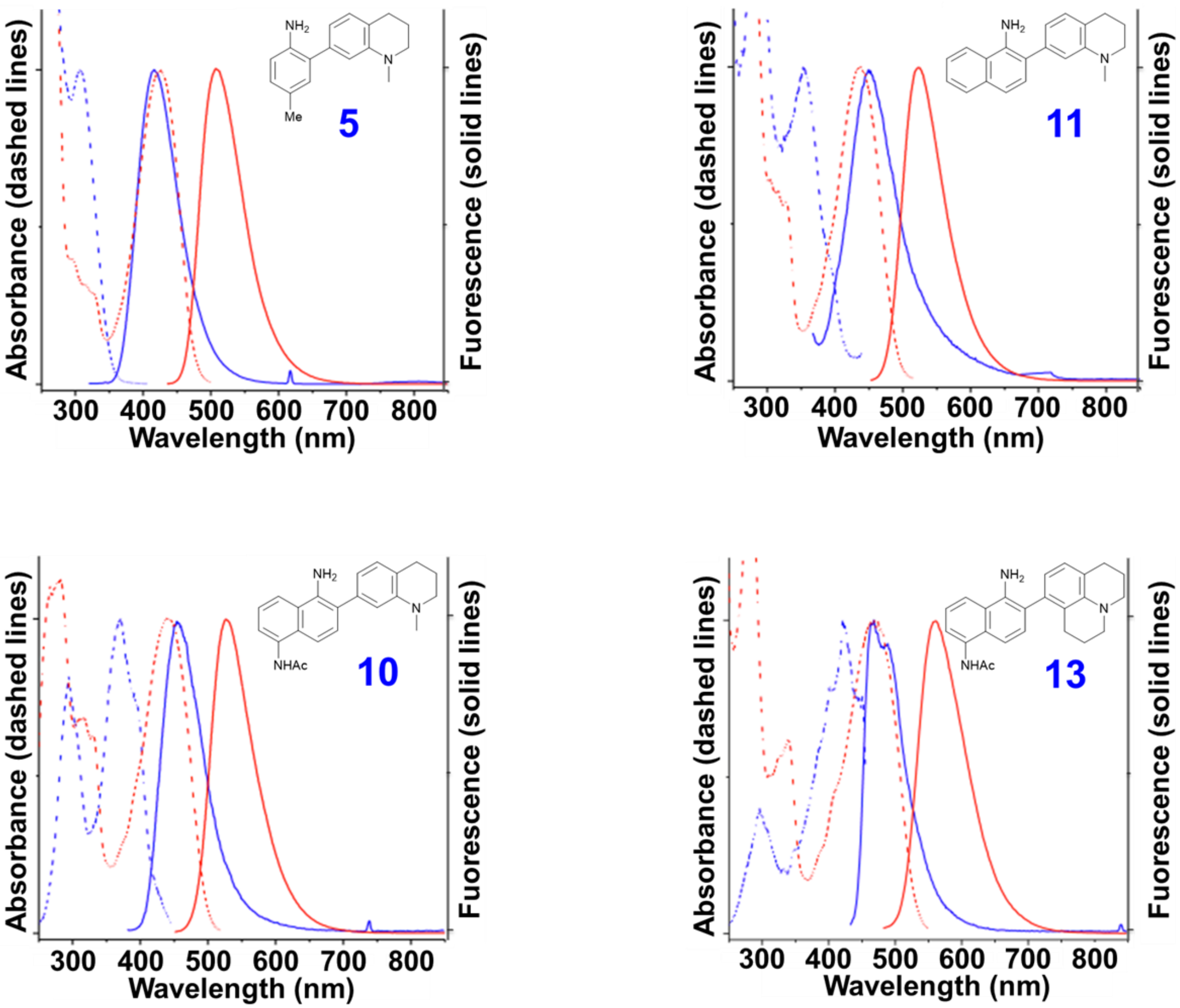

*Figure S1: Normalized absorbance (dotted lines) and fluorescence emission (solid lines) spectra of probes **5**, **10**, **11**, and **13** (blue) and of their cinnoline products (red). The spectra were acquired in 1:4 DMSO / 50 mM phosphate buffer at pH 7.4. If there is sufficient resolution of probe excitation (absorbance) from cinnoline excitation, with proper optical filters both probe and cinnoline can be imaged independently from one another. For **13**, probe and cinnoline excitation are not well resolved.*

Fluorescence Titrations

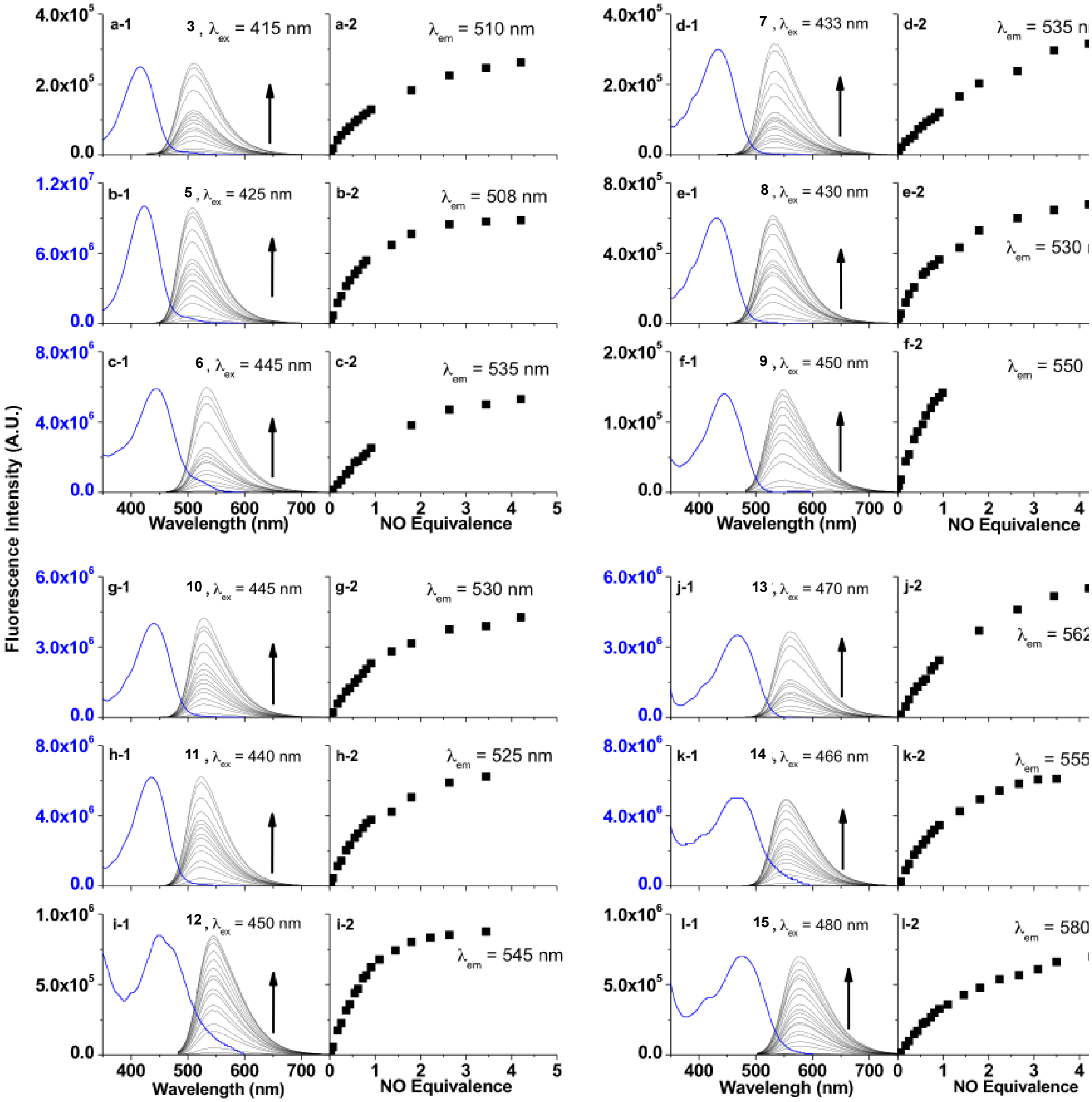

*Figure S2: Fluorescence titrations of probes **3**, **5-15** with NO. a1-l1) Fluorescence emission spectra (black) of titrations of 50 µM probe in 1:4 DMSO/50 mM phosphate buffer at pH 7.4 with NO at the specified excitation (λ_{ex}) and absorbance spectra (blue) of 50 µM solutions of their corresponding cinnoline. a2-l2) Plots of emission intensity of cinnoline, formed upon addition of NO, at the specified wavelength (λ_{em}) versus added equivalents of NO.*

DAPI and FITC Filter Transmission Spectra

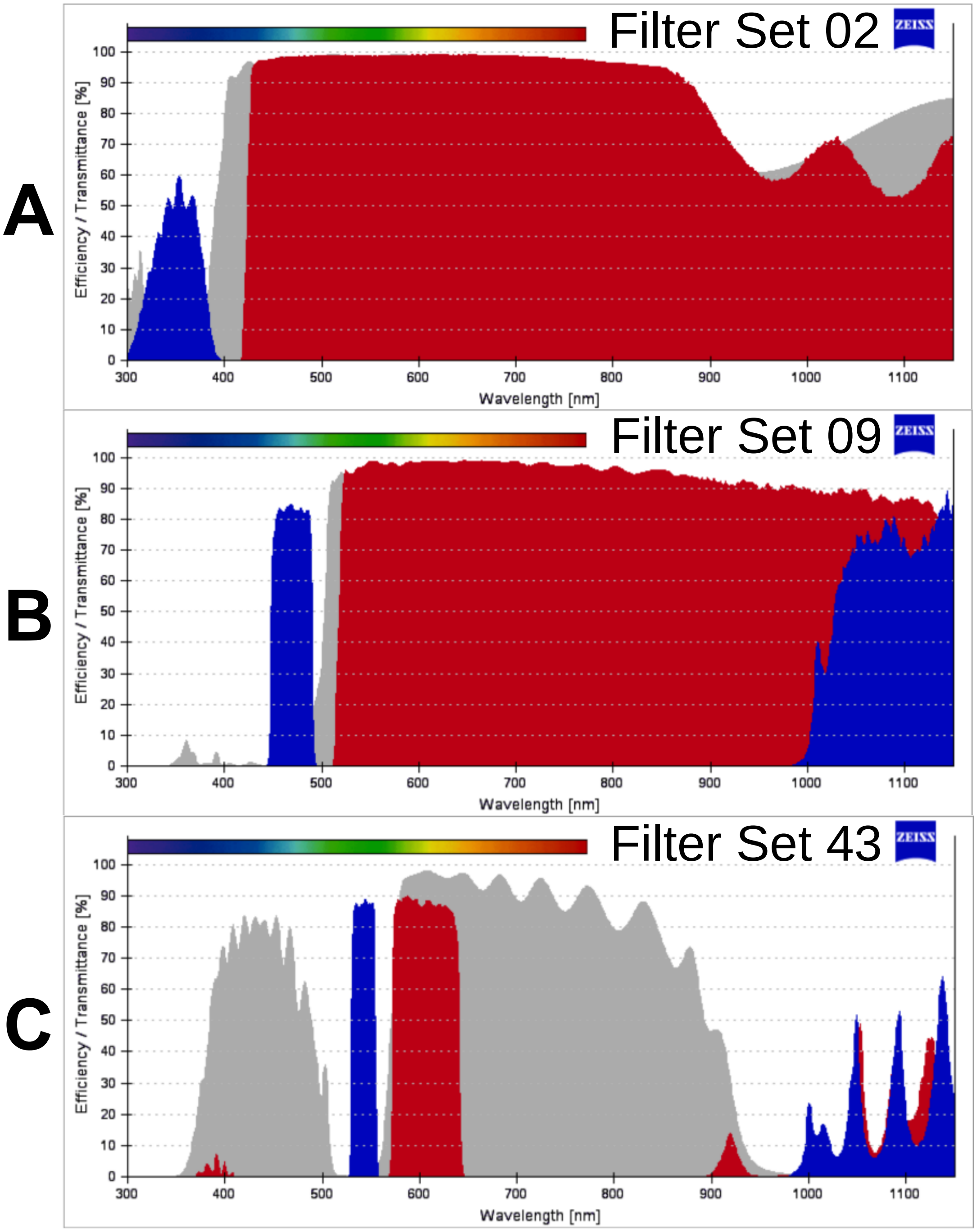

Figure S3: Percent transmittance versus wavelength graphs for DAPI (top), FITC (middle), and TRITC (bottom) filters.[2] *Excitation filter (blue), beam splitter (gray), and emission filter (red) parameters (listed in this order) for each set: DAPI (G 365, FT 395, LP 420), FITC (BP 450-490, FT 510, LP 515), and TRITC (545/25, FT 570, BP 605/70). The absorbance spectrum for probe 13 (Figure S1), poorly resolved from AZO-13, might fit better to the FITC filter than the DAPI filter. Purported cinnoline fluorescence from the FITC channel would therefore have a contribution from unreacted probe fluorescence.*

Cellular Experiments

Cell Cultures

NIH 3T3 and RAW 264.7 cells (ATCC) were cultured in high-glucose Dulbecco's Modified Eagle Medium (DMEM) or Leibovitz's L-15 Medium (L-15) with 10% BCS (NIH 3T3) or 10% FBS (RAW 264.7) added. For the bicarbonate-buffered DMEM medium, the cells were incubated in a humidified environment at 37 °C under 5% CO_2/air, whereas for the phosphate-buffered L-15 medium, no CO_2 was added. Cells were passaged at least three times prior to any imaging experiments. For fluorescence microscopy, cells were plated onto Lab-Tek II Chambered #1.5 German Coverglass sterile 8-well plates in DMEM or L-15 medium supplemented with 1% serum. For endogenous NO imaging, RAW264.7 cells were seeded in 384-well clear-bottom plates (Greiner Bio-One) in DMEM without L-Arginine supplemented with 1% FBS. NO production was quantified in high content imaging instrument (INCell 2200, GE Healthcare), as described below.

Passaging Cells

DMEM + 10% BCS medium was removed from 70-90% confluent NIH 3T3 cells in T25 culture flasks, and 0.05% trypsin-EDTA solution with phenol red was promptly added. The cells were incubated at 37 °C under 5% CO_2 for one minute so they would detach, and then the cell suspension was transferred to a 15 mL centrifuge tube. The culture flask was rinsed with 3 mL DPBS, and the cells were centrifuged to a pellet. The supernatant was discarded, and the cells were suspended in 1 mL DMEM + 10% BCS. 80-100 μL of this suspension was added to a new culture flask containing 4 mL DMEM + 10% BCS, and the cells were incubated at 37 °C under 5% CO_2 to complete the passage.

DMEM + 10% FBS was removed from 70-90% confluent RAW 264.7 cells, and 1 mL DPBS was added. The cells were detached with a cell scraper, and the cell suspension was transferred to a 15 mL centrifuge tube. The culture flask was rinsed with 3 mL DPBS, and the cell suspension was centrifuged. The cell pellet was suspended in 1 mL DMEM + 10% FBS, and an 80-100 μL aliquot of this suspension was added to 4 mL DMEM + 10% FBS in a new culture flask. The cells were incubated at 37 °C under 5% CO_2 to complete the passage.

Cell plating

Each well of a Nunc Lab-Tek II Chambered Coverglass 8-well plate was charged with 500 μL DMEM + 1% BCS (NIH 3T3) or FBS (RAW 264.7). The centrifuged cell pellet obtained as described in the section above was suspended in 1 mL DMEM + 1% serum. A 10-20 μL aliquot of this suspension was added to each well, and the cells were incubated at 37 °C under 5% CO_2.

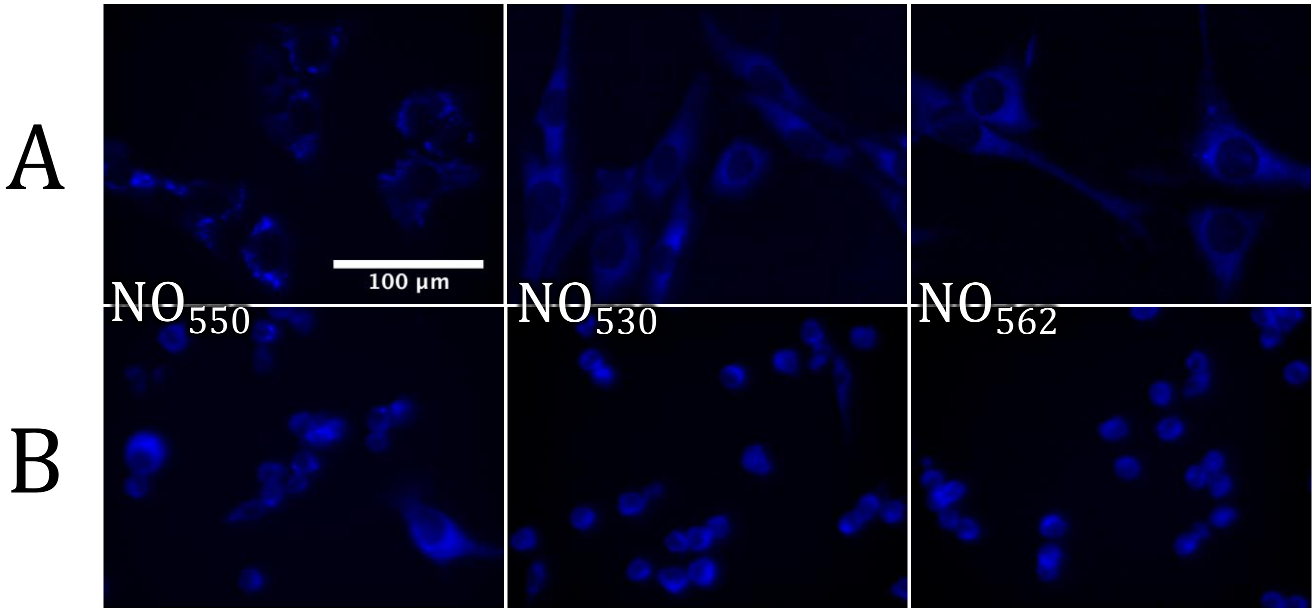

Probe loading evaluated by DAPI-filtered images

Figure S4: Pseudocolored DAPI-filtered images (40x magnification) of probes ***NO_{550}, NO_{530}***, *and* ***NO_{562}*** *in A) NIH 3T3 and B) RAW 264.7 cells. The DAPI filter (Figure S3 A) mostly captures unreacted probe emission. For the most part, probe loading is cytosolic and non-nuclear.*

SNAP stimulus experiments

*NIH 3T3 cells were plated into a Nunc 8-well plate in high-glucose DMEM + 1% BCS and incubated overnight at 37 °C under 5% CO_2/air. The DMEM was then replaced with Hepes Hanks Buffered Saline Solution (HHBSS) + 1% BCS, and the cells were incubated at 37°C for twenty minutes. 2 mM probe solutions of NO_{550} (**9**), NO_{530} (**10**), or NO_{562} (**13**) in DMSO were diluted in HHBSS + 1% BCS to give a 15 µM probe concentration, with 0.75% DMSO. The medium in a pair of wells was replaced with 15 µM probe loading solution – three probes, six wells total. To the no-probe pair of wells was added the same volume of DMSO without probe to give 0.75% DMSO. The cells were incubated in this loading solution for two hours at 37 °C. The loading solution was then replaced with HHBSS + 1% BCS. A 200 mM S-Nitroso N-acetyl D,L-penicillamine (SNAP) solution in DMSO was diluted into the medium of four wells (stimulated), containing the three different probes and no probe, to give 1 mM SNAP, 1.23% DMSO. The same volume of DMSO was diluted into the medium of the remaining wells (non-stimulated) to give 1.23% DMSO. The cells were incubated for four hours at 37 °C. Brightfield and DAPI and FITC-filtered fluorescence images were acquired through 10x and 40x objectives.*

RAW 264.7 cells were plated into a Nunc 8-well plate high-glucose DMEM + 1% FBS and incubated overnight at 37 °C under 5% CO_2/air. The DMEM was then replaced with Krebs-Ringer buffer (KRB) + 1% FBS. 2 mM probe solutions of ***9***, ***10***, *or* ***13*** *in DMSO were diluted in KRB + 1% FBS to a final concentration of 10 µM, 0.5% DMSO, and the medium over cells was replaced with this*

10 μM loading solution, two wells per probe, six wells total. A 25 mM SNAP solution in DMSO was diluted into the loading solution of three wells (stimulated), each containing a different probe, to give 200 μM SNAP, 1.3% DMSO. The three remaining wells (non-stimulated) were treated with the same volume of DMSO but without SNAP, to give 1.3% DMSO. The cells were incubated for four hours at 37 °C. The medium in all wells was replaced with KRB + 1% FBS. Brightfield and DAPI and FITC-filtered fluorescence images were acquired through 10x objective.

NO Solution Stimulus

*To the wells containing NIH 3T3 cells - loaded with probes **9**, **10**, or **13** or with no probe at all - that had not been subjected to 1 mM SNAP (above) was added 1.9 mM aqueous NO solution to a final concentration of 250 μM. The cells were incubated at 37 °C for 10 minutes, and then brightfield and DAPI and FITC-filtered fluorescence images of the four wells were acquired through 10x and 40x objectives.*

*RAW 264.7 cells were plated into a Nunc 8-well plate high-glucose DMEM + 1% FBS and incubated overnight at 37 °C under 5% CO_2/air. The DMEM was then replaced with DPBS + 1% FBS. 2 mM probe solutions of **9**, **10**, or **13** in DMSO were diluted in DPBS + 1% FBS to a final concentration of 10 μM, 0.5% DMSO, and the medium over cells was replaced with this 10 μM loading solution, two wells per probe, six wells total. Two wells were left without probe, but DMSO was added to the medium to 0.5%. The cells were incubated at 37 °C for 9 hours, and then the 10 μM loading solutions were replaced with DPBS + 1% FBS. A 1.8 mM aqueous NO solution was diluted into the medium in three wells (stimulated), each containing a different probe, and one no-probe well (also stimulated), to a final concentration of 300 μM NO. Nothing was added to the remaining four wells (non-stimulated). The cells were incubated for ten minutes at 37 °C, and then brightfield and DAPI and FITC-filtered fluorescence images of all wells were acquired through 10x and 40x objectives.*

LPS screen

Lipopolysaccharides from Escherichia coli: 026:B6 (L2654), 055:B5 (L6529), 0111:B4 (L4391) and purified by phenol extraction (L2630), and 0127:B8 (L4516) and from Salmonella enterica: serotype enteritidis (L7770), serotype Minnesota (L4641), and serotype typhimurium (L6143) were purchased from Sigma (catalog numbers in parentheses). 1 mg of each LPS variant was dissolved in 1 mL water sterilized by 0.2 μm filtration. To seven wells (stimulated) of an 8-well plate that had been plated with RAW 264.7 cells in DMEM + 1% FBS was added an appropriate aliquot of a different LPS variant so as to achieve a final concentration of 1 μg/mL LPS. No LPS was added to one well (non-stimulated). The cells were incubated for 90 minutes at 37 °C, 5% CO2. A 1 mM solution of DAF-FM diacetate in DMSO was diluted to 2 μM in DMEM + 1% FBS, and the medium in all wells was replaced with this 2 μM solution. After incubation at 37 °C, 5% CO2 for 30 minutes, the medium was replaced with KRB, and the cells were incubated for 5 minutes further at room temperature. They were then imaged through the FITC filter set. Cells

stimulated with variant Escherichia coli 011:B4 (L4391) produced the greatest increase in DAF-FM brightness over the non-stimulated cells, although the same variant purified by phenol extraction (L2630) also produced a greater increase than the remaining variants.

Cell exposure to LPS

*RAW 264.7 cells were plated into a Nunc 8-well plate in high-glucose DMEM + 1% FBS and incubated overnight at 37 °C under 5% CO_2/air. To four (stimulated) of the eight wells was added 1 mg/mL LPS in 0.2 µm filtered water to a final concentration of 1 µg/mL. Nothing was added to the remaining four wells (non-stimulated). The cells were incubated at 37 °C, 5% CO_2 for 15 hours. The medium in all wells was replaced with L-15 + 1% FBS, and 1 mg/mL LPS solution was diluted to 1 µg/mL in the four stimulated wells. The cells were incubated at 37 °C for another six hours. A 2 mM solution of probes **9**, **10**, and **13** in DMSO was first diluted to 150 µM, 7% DMSO in L-15 + 1% FBS in order to avoid high localized concentrations of DMSO over the cells. Each 150 µM probe solution was further diluted to 15 µM, 0.7% DMSO into two wells, stimulated and non-stimulated, for a total of six wells, and the cells were incubated at 37 °C for 2.5 hours. A 1 mM DAF-FM diacetate solution in DMSO was diluted to 10 µM in L-15 + 1% FBS and then diluted further to 1 µM in the two remaining, one stimulated and one non-stimulated, wells. After 25 minutes at room temperature, the DAF-FM diacetate loading medium was replaced with L-15 + 1% FBS. The cells were left for another ten minutes at room temperature for non-specific esterases to finish hydrolyzing the two acetate functionalities. (This extra hydrolysis time must be counterbalanced with the propensity of the hydrolyzed form of DAF-FM to leak or get pumped out of cells.) Brightfield and DAPI and FITC-filtered fluorescence images were acquired for all wells (starting with the two containing the leakage-prone DAF-FM) through a 10x objective.*

Analysis of endogenous NO production in RAW264.7 cells

RAW264.7 cells were seeded in DMEM without L-Arginine supplemented with 1% FBS in 384 well clear-bottom plates (Greiner Bio-One) at 10,000 cells per well. The cells were activated by overnight incubation with 100 µg/mL LPS and 200 EU/mL IFN-γ. On the next day, the media was replaced with HBSS with Ca^{++} and Mg^{++} (HBSS/Ca^{++}/Mg^{++}) and the cells were treated with increasing concentrations of L-Arginine with or without pan-NO synthase competitive inhibitor L-NMMA. NO probes 10 µM NO_{530} or 1 µM DAF-FM DA were added simultaneously with L-Arginine titers and the cells were incubated for 30 minutes at 37 °C. At te end of the incubation, cells were washed with HBSS/Ca^{++}/Mg^{++}. Hoechst 33342 (2 µg/mL) was used as a nuclear stain. Images of cells were taken using INCell 2200 high content analyzer equipped with 20x objective. Images are uploaded at and can be accessed via Creative Scientist's website (www.cscientist.com).

Calcein Blue/Propidium Iodide Cell Viability Assay

The membrane-permeable acetoxymethyl ester derivative of Calcein Blue is non-fluorescent until non-specific esterases in viable cells hydrolyze the esters to produce the membrane-impermeable blue fluorophore that remains in the cytosol of cells with intact membranes. Propidium iodide becomes red fluorescent when it intercalates into DNA. The membrane-impermeable propidium iodide cannot intercalate into the DNA of viable cells with intact membranes. Therefore, blue fluorescent cells are deemed viable, whereas red fluorescent are not.

The DMEM + 1% FBS medium over RAW 264.7 cells was replaced with L-15 + 1% FBS; 4% DMSO in L-15 + 1% FBS; or 1.25, 2.5, 5, 10, 20, or 40 μM ***10*** *(diluted from 2 mM probe* ***10*** *in DMSO) in L-15 + 1% FBS. Cells were incubated at 37 °C for two hours. A 2 mM Calcein Blue (AM) solution in DMSO was diluted to 50 μM in DPBS. A 1.5 mM propidium iodide solution in 0.2 μm-filtered water was diluted to 50 μM in the 50 μM Calcein Blue (AM) solution. The medium in all wells was replaced with DPBS, and then the 50 μM Calcein Blue (AM), 50 μM propidium iodide solution was diluted ten-fold into each well. Cells were incubated at 37 °C for 30 minutes prior to imaging. Brightfield and DAPI, FITC, and TRITC-filtered fluorescence images were acquired for all wells.*

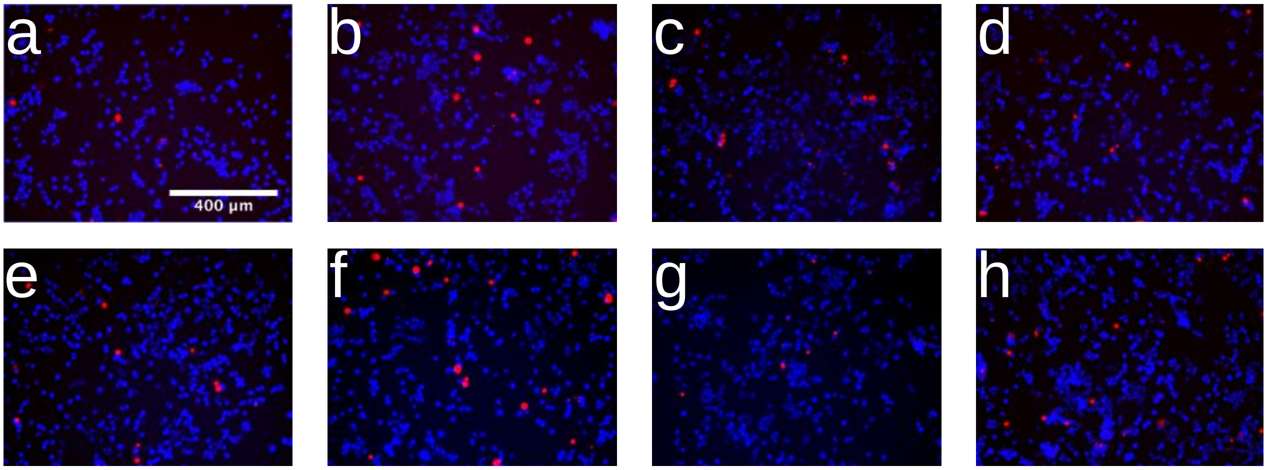

Figure S5: Pseudo-colored, 10x magnification images of Calcein Blue (DAPI-filtered, live cells) and Propidium Iodide (TRITC-filtered, red, dead cells) in RAW 264.7 cells incubated for two hours with 1.25 (c), 2.5 (d), 5 (e), 10 (f), 20 (g), and 40 (h) μM NO_{530}, as well as cells incubated with nothing (a) or with only 0.25% DMSO (b) for two hours.

Image processing

Images were saved as TIFFs. All images were processed with FIJI software, version 2.0.0-rc-65/1.52a.[3] *Brightfield images (e.g.; Figures S6a and S7a) were processed with the Subtract Background command, typically with a rolling ball radius of 10 pixels and with smoothing disabled, followed by further processing with a variance filter set at a radius of 2-5 pixels (Figure S7b). A threshold was applied to the background-subtracted and variance-filtered images to reduce them to 8-bit images from which a selection could be created (Figures S6e and S7d). This selection was converted to a region of interest and served as a mask for areas occupied by cells. The rolling ball radius values in the Subtract Background command and the radius values in the variance filter were adjusted to achieve the best fit of the region of interest to the cells in the image (Figure S6b, c, and d). Each brightfield image-derived mask was applied to the corresponding FITC and DAPI channel images to obtain the mean pixel intensity within the mask, which was treated as intracellular signal (Figures S6g,h and S7e,f). An inverse of the selection produced a mask of the region not occupied by cells (Figure S6f), and this mask was applied to the FITC and DAPI channel images to measure extracellular signal. An image 960 x 720 pixels in size contains 691,200 pixels. Processing Figure S7e and f gave a mean intensity value of 1316 for FITC and 705 for DAPI over 138,173 pixels for the intracellular signal. The inverse of the selection produced a mean of 636 for FITC and 604 for DAPI over 553,027 pixels for the extracellular signal. The intracellular and extracellular pixels totaled to 691,200 pixels.*

These signals were corrected by subtracting the mean intracellular signal from cells not loaded with probe from those loaded with probe for experiments with SNAP, NO solution, and LPS stimuli in NIH 3T3 and RAW 264.7 cells. For LPS stimuli experiments in cells, images of cells not loaded with probes were not collected, so the lowest extracellular mean signal, no matter which probe was loaded into cells, in a certain experiment was subtracted from the mean intracellular signals for all loaded probes. Furthermore, if at any point the intracellular signal from cells not loaded with probe was greater than that for cells loaded with probe, the lowest extracellular mean signal in the same experiment was subtracted from the mean intracellular signal for that probe.

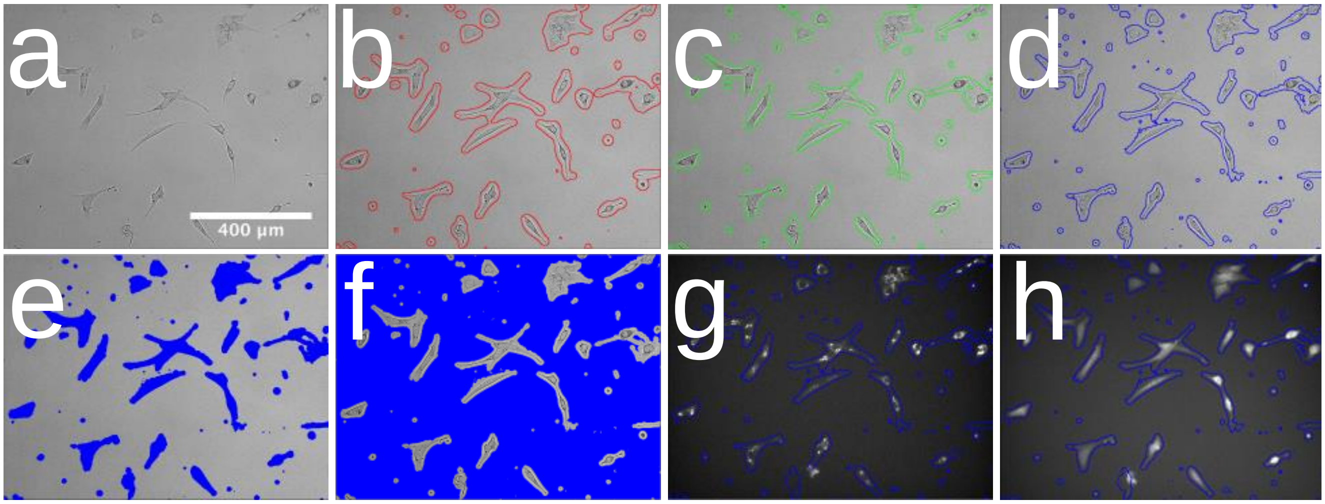

Figure S6a: Sample processing of NIH 3T3 cell images at 10x magnification. a) brightfield image, b) region of interest (ROI) after background and variance filter processing, c) improved ROI upon adjusting background subtraction and variance filter parameters, d) optimal ROI, e) threshold mask for cells, f) inverse mask for extracellular region, g) cellular mask applied to DAPI-filtered image, h) intracellular mask applied to FITC-filtered image

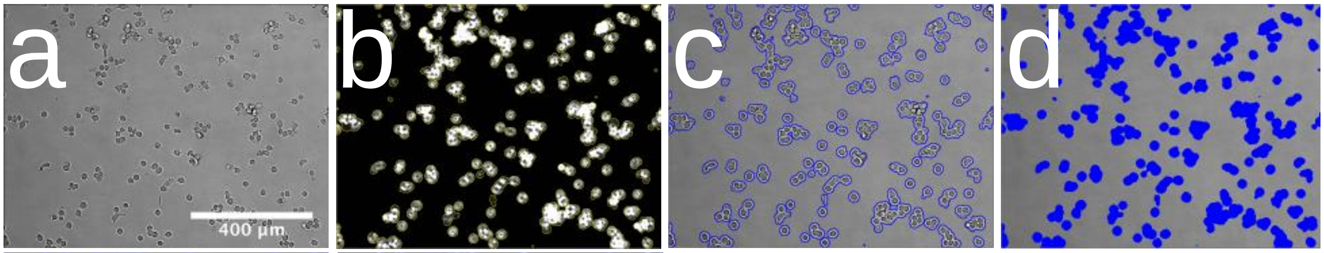

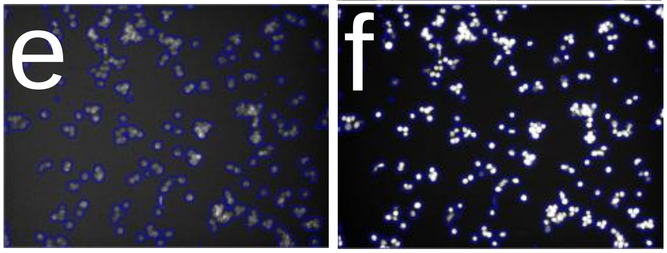

	**area**	**mean**
D_{intra}	138173	705.321
D_{extra}	553027	604.164
F_{intra}	138173	1316.318
F_{extra}	553027	636.323

Figure S6b: Sample processing of RAW 264.7 cell images at 10x magnification. a) brightfield image, b) image after background subtraction and variance filters, c) selection of region of interest (ROI), d) threshold mask for cells, e) cellular mask applied to DAPI-filtered image, f) cellular mask applied to FITC-filtered image. Results: Area of cellular ROI is 138,173 pixels and of extracellular ROI, 553,027 pixels. D and F are abbreviations for DAPI and FITC channels, respectively. The average intensity per pixel within the region of interest is listed as the mean.

References

1. Yang, Y.; Seidlits, S. K.; Adams, M. M.; Lynch, V. M.; Schmidt, C. E.; Anslyn, E. V.; Shear, J. B., A Highly Selective Low-Background Fluorescent Imaging Agent for Nitric Oxide. *J. Am. Chem. Soc.* **2010,** *132* (38), 13114-13116.

2. (a) Carl Zeiss Microscopy, L. Filter Set 02. https://www.micro-shop.zeiss.com/images/fat/chart.gif.php?l=en&a=v&ft=446431-0011-000&fa=000000-4242-879&fe=447741-8001-000; (b) Carl Zeiss Microscopy, L. Filter Set 09. https://www.micro-shop.zeiss.com/images/fat/chart.gif.php?l=en&a=v&ft=446431-0011-000&fa=000000-4242-879&fe=447741-8001-000; (c) Carl Zeiss Microscopy, L. Filter Set 43. https://www.micro-shop.zeiss.com/images/fat/chart.gif.php?l=en&a=v&ft=446431-0011-000&fa=000000-4242-879&fe=447741-8001-000.

3. (a) Schindelin, J.; Arganda-Carreras, I.; Frise, E.; Kaynig, V.; Longair, M.; Pietzsch, T.; Preibisch, S.; Rueden, C.; Saalfeld, S.; Schmid, B.; Tinevez, J.-Y.; White, D. J.; Hartenstein, V.; Eliceiri, K.; Tomancak, P.; Cardona, A., Fiji: an open-source platform for biological-image analysis. *Nature Methods* **2012,** *9*, 676; (b) Schneider, C. A.; Rasband, W. S.; Eliceiri, K. W., NIH Image to ImageJ: 25 years of image analysis. *Nature Methods* **2012,** *9*, 671.

^{1}H, ^{13}C, and HRMS spectra of NO probes

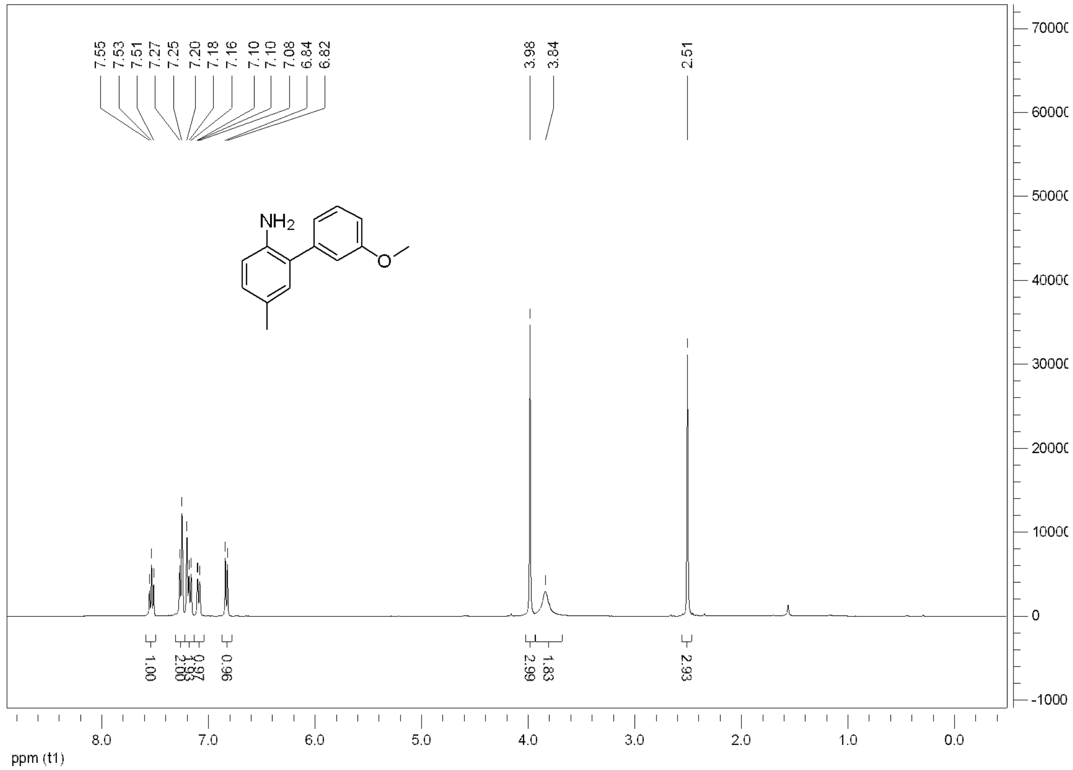

Figure S7: The ^1H-NMR of compound 1.

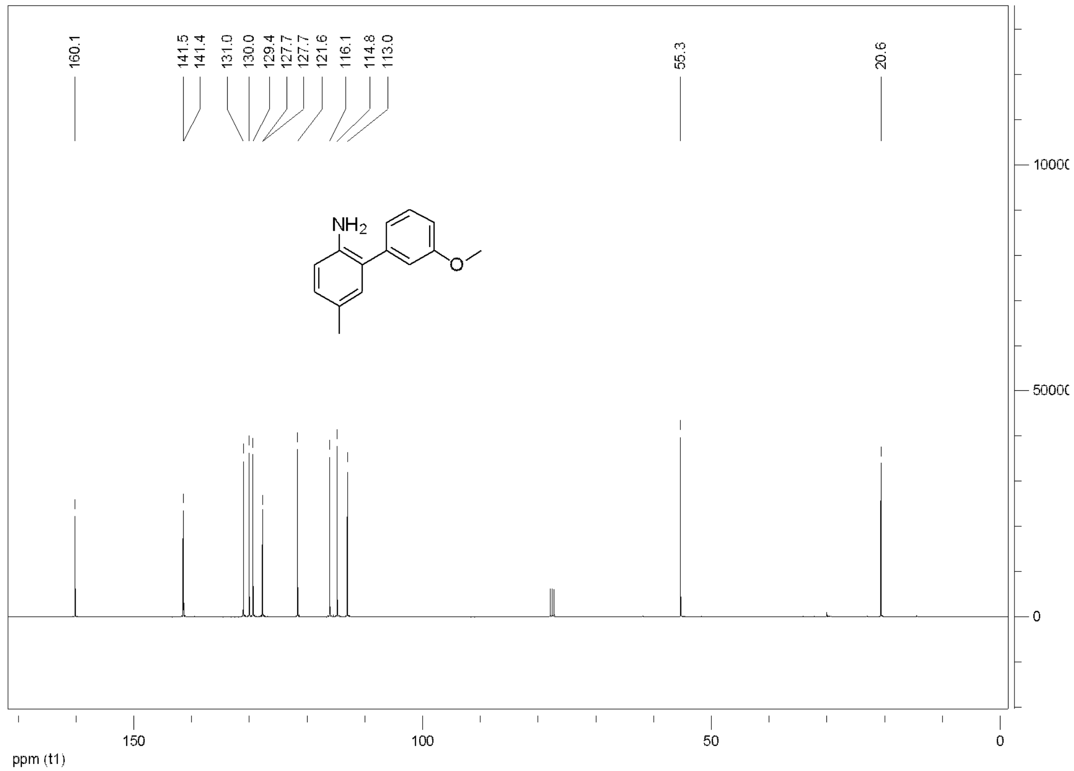

Figure S8: The ^{13}C-NMR spectrum of compound 1

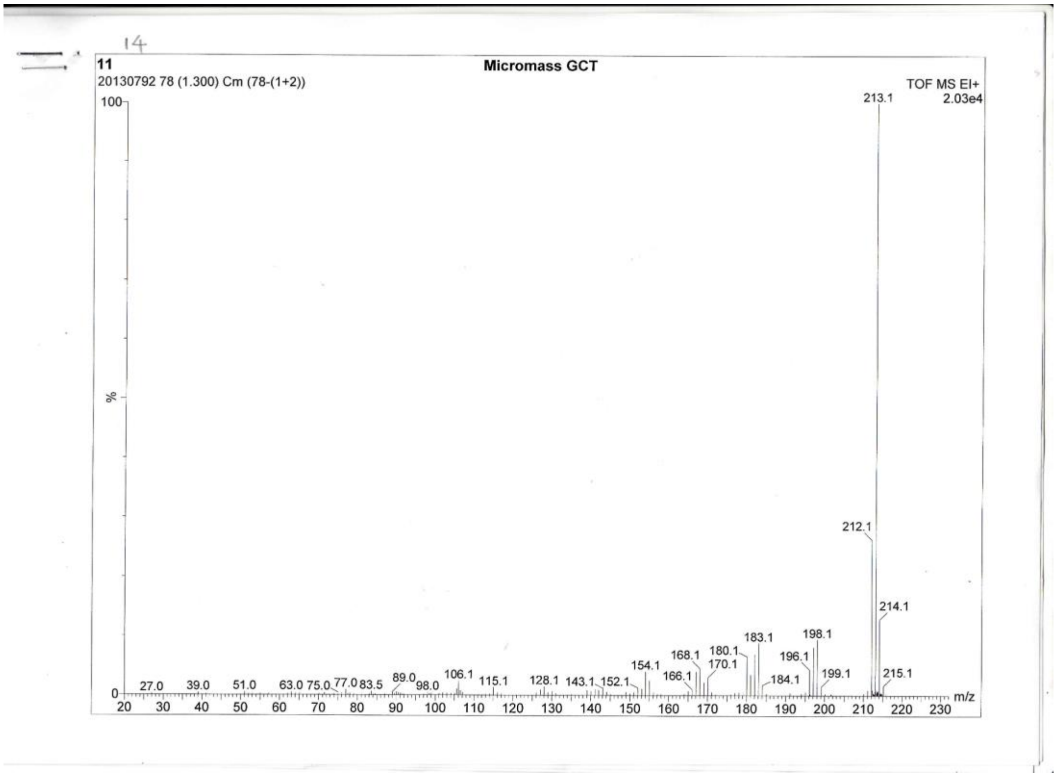

Figure S9: The MS spectrum of compound 1

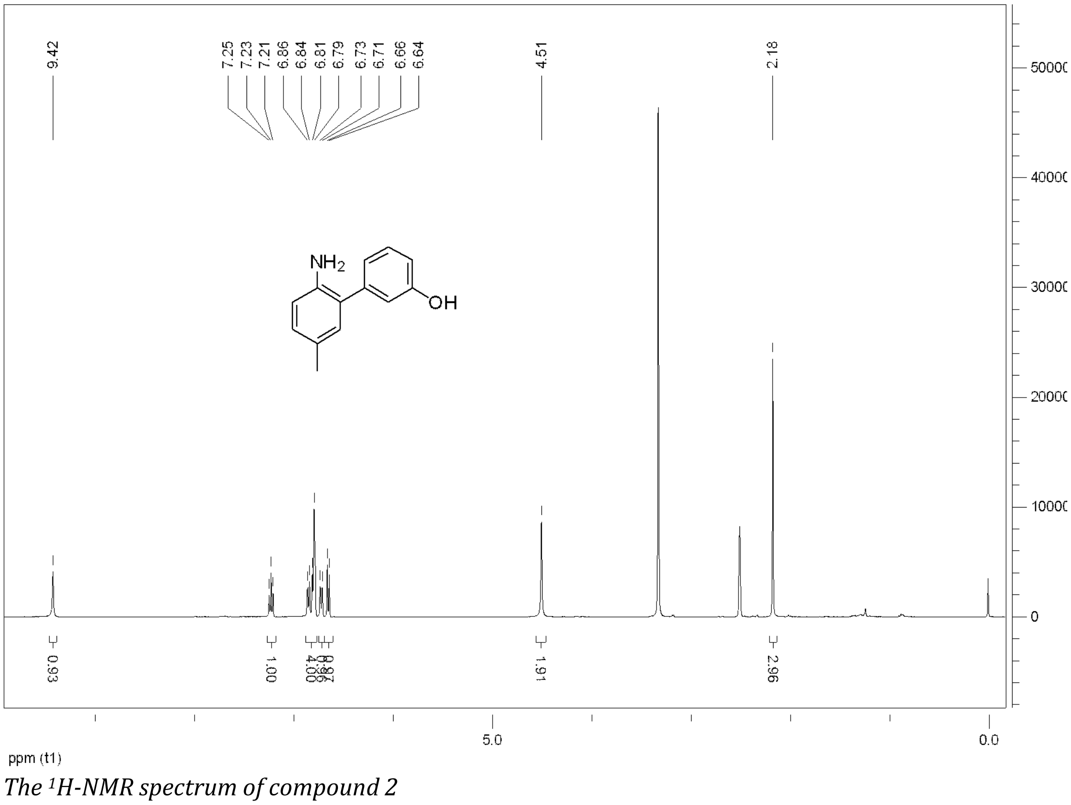

Figure S10: The ^{1}H-NMR spectrum of compound 2

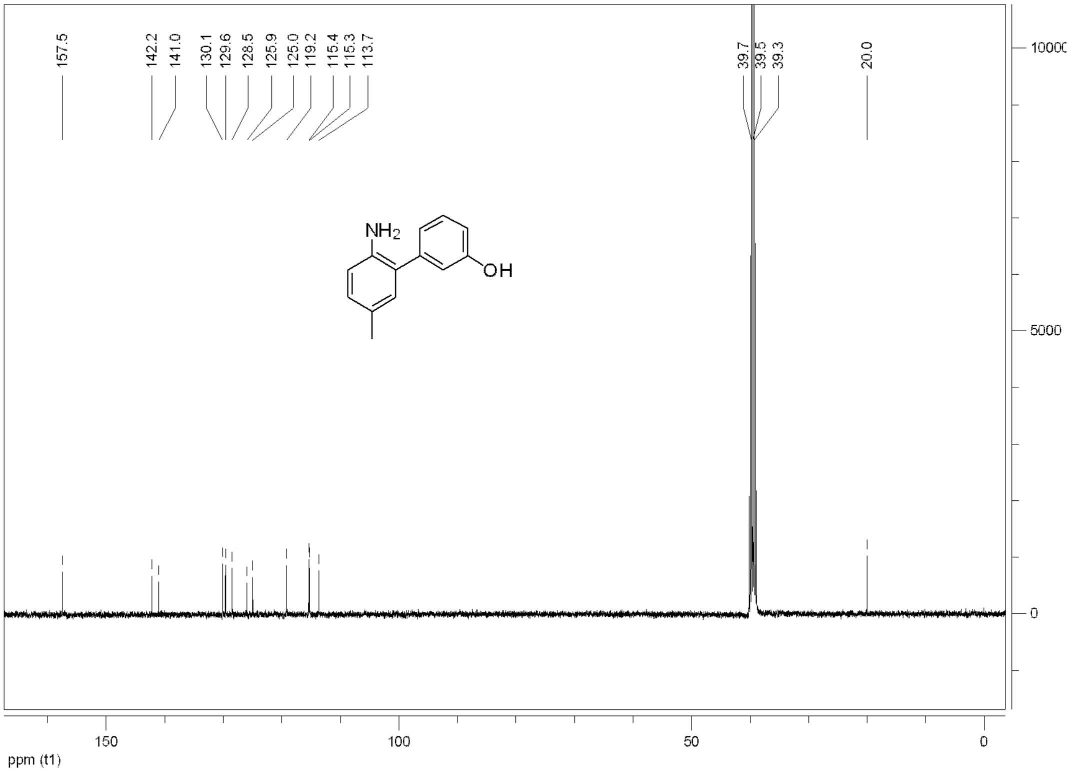

Figure S11: The ^{13}C-NMR spectrum of compound 2

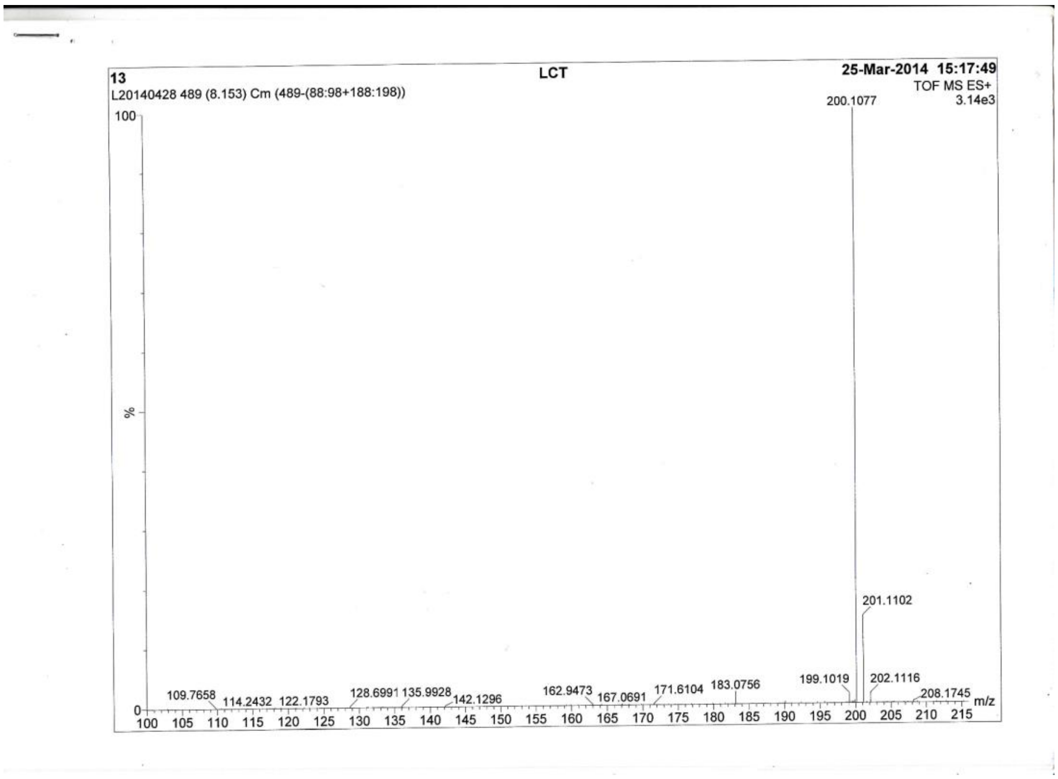

Figure S12: The HRMS spectrum of compound 2

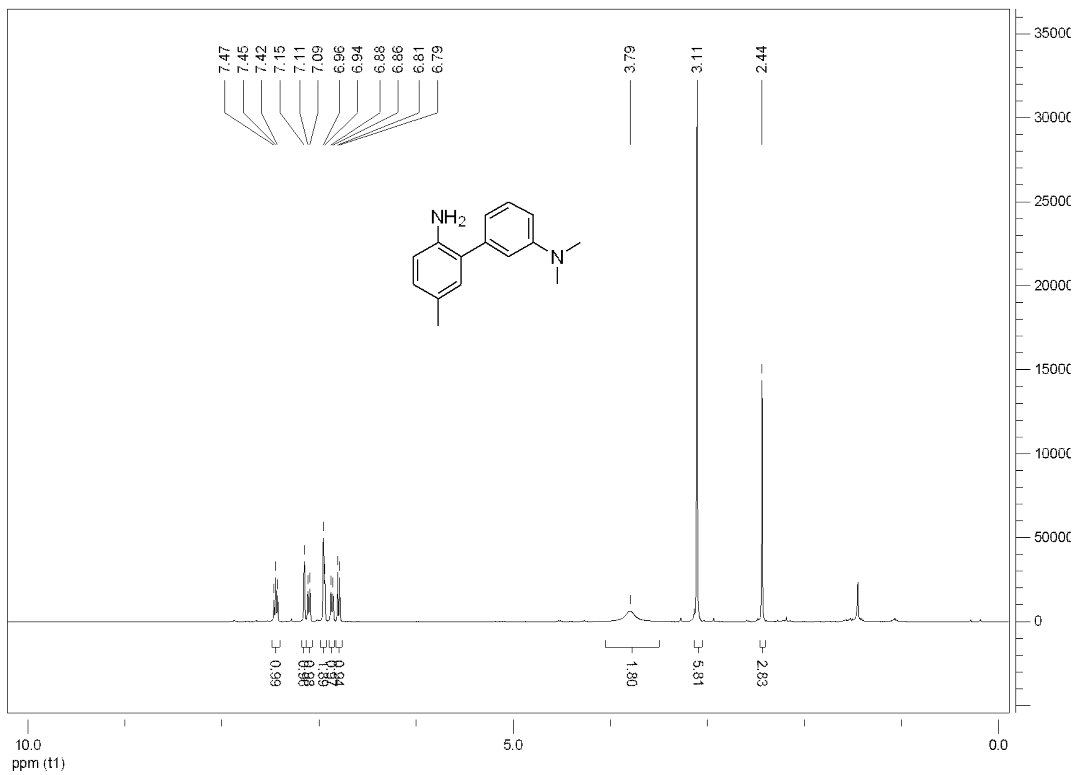

Figure S13: The ^{1}H-NMR spectrum of compound 3

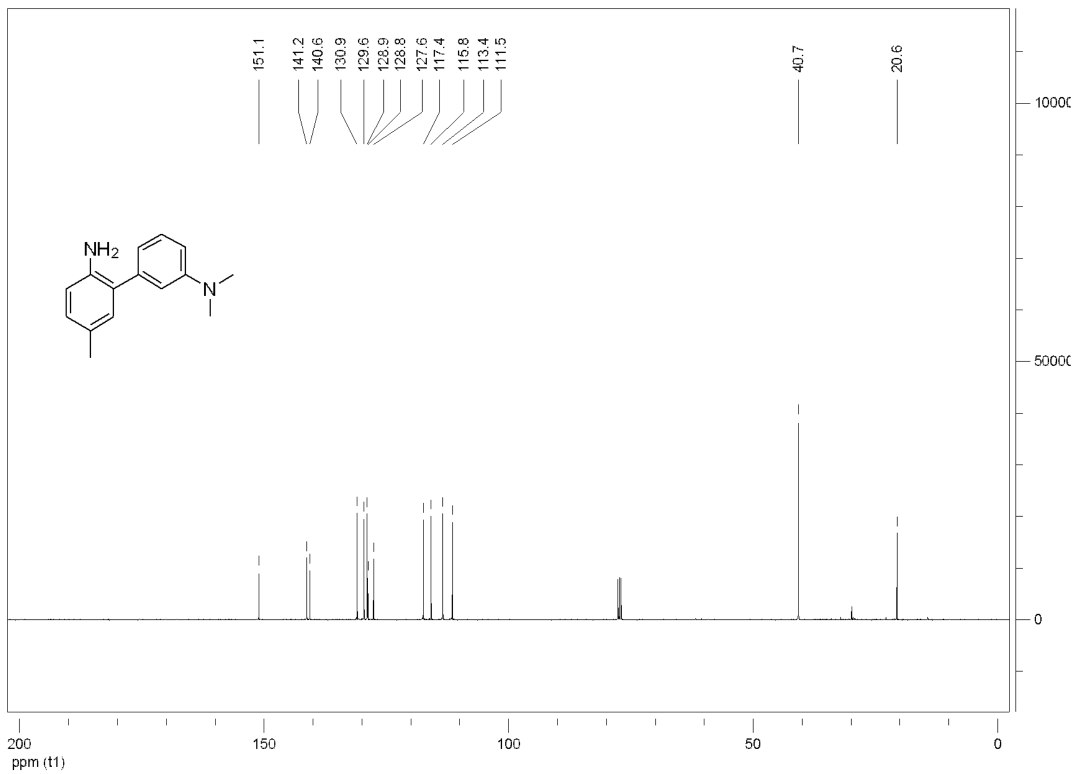

Figure S14: The ^{13}C-NMR spectrum of compound 3

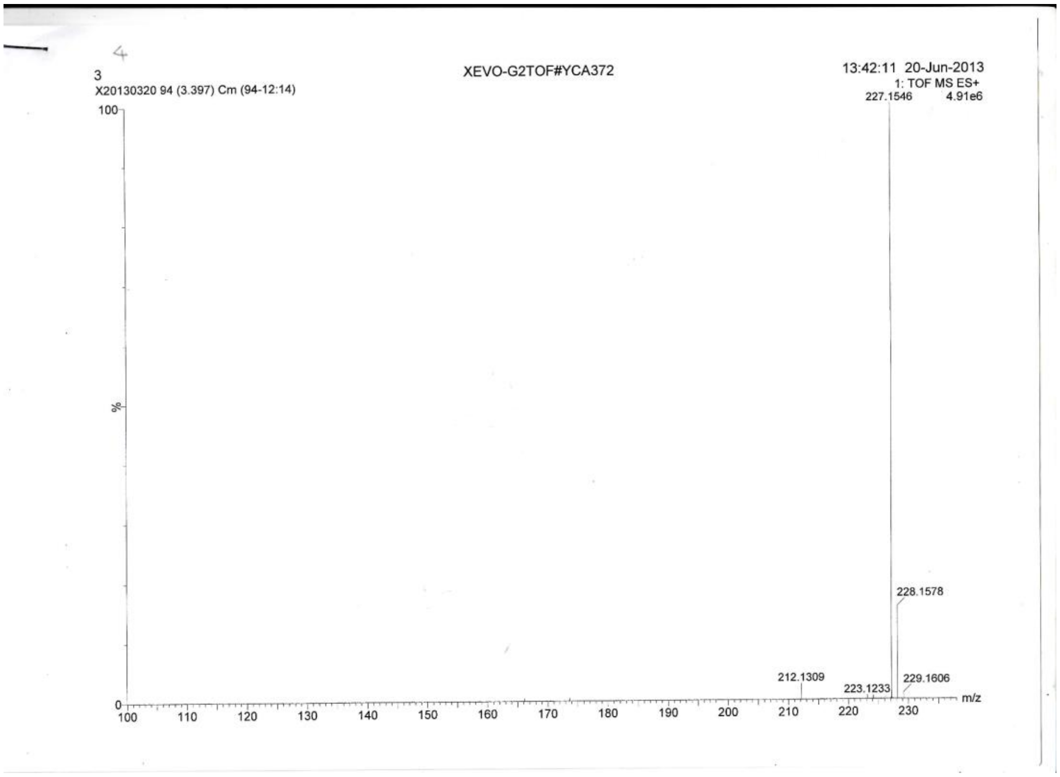

Figure S15: The HRMS spectrum of compound 3.

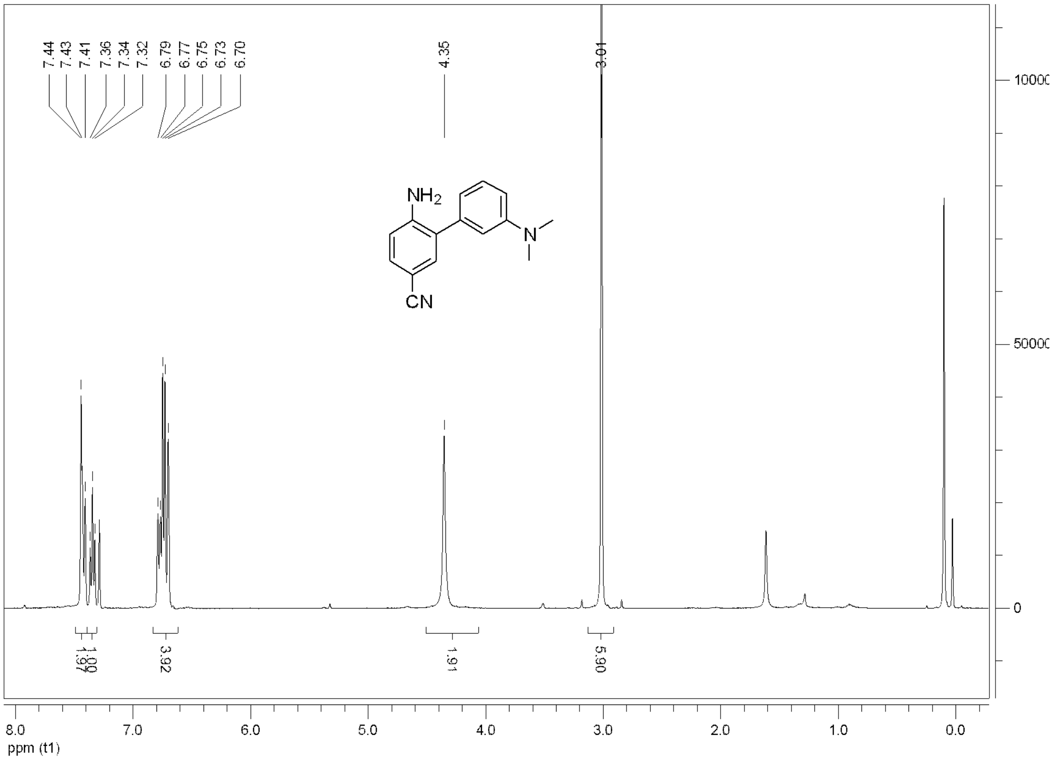

Figure S16: The ^{1}H-NMR spectrum of compound 4

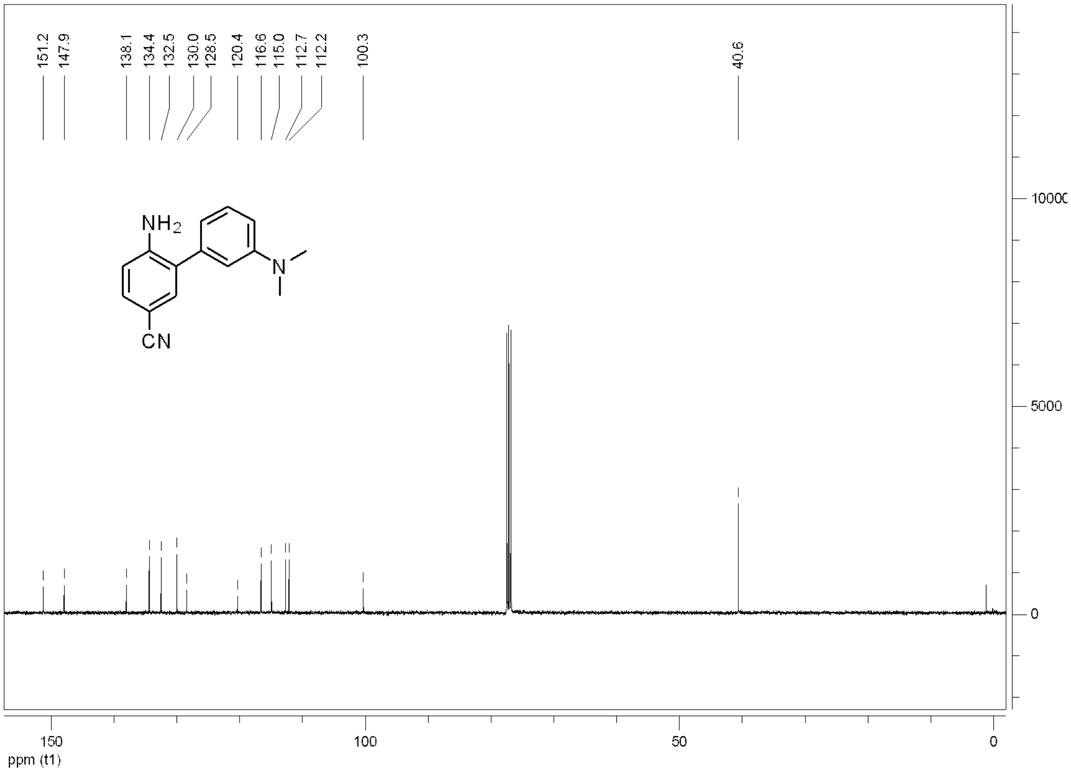

Figure S17: The ^{13}C-NMR spectrum of compound 4

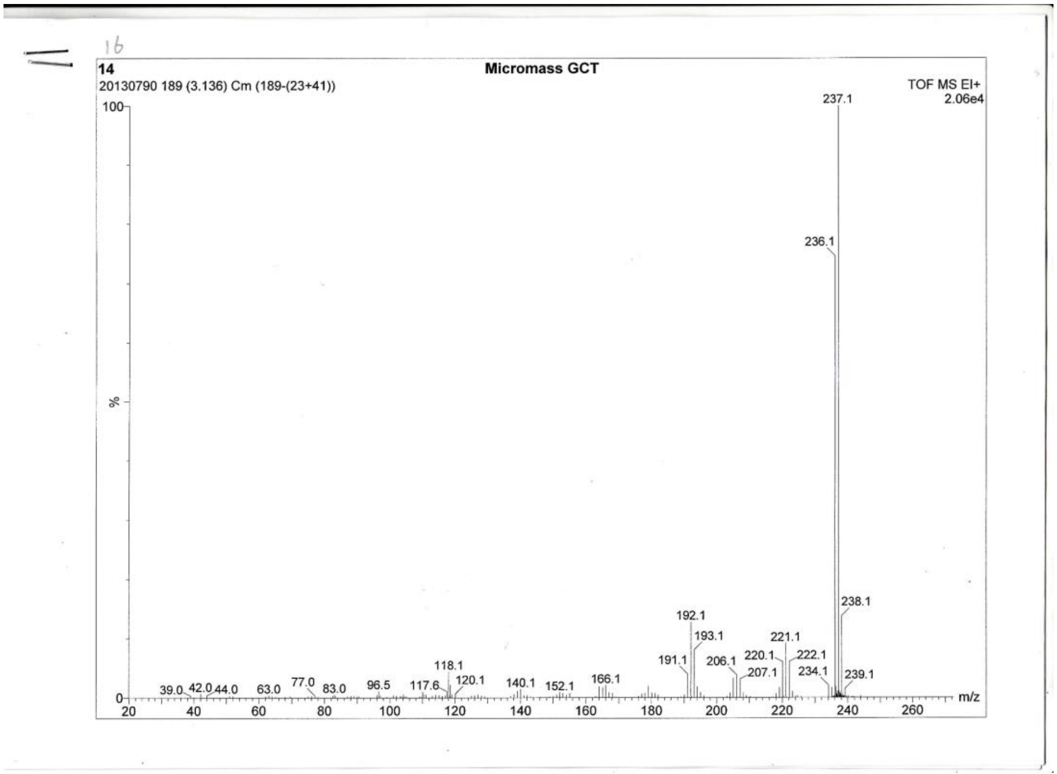

Figure S18: The MS spectrum of compound 4

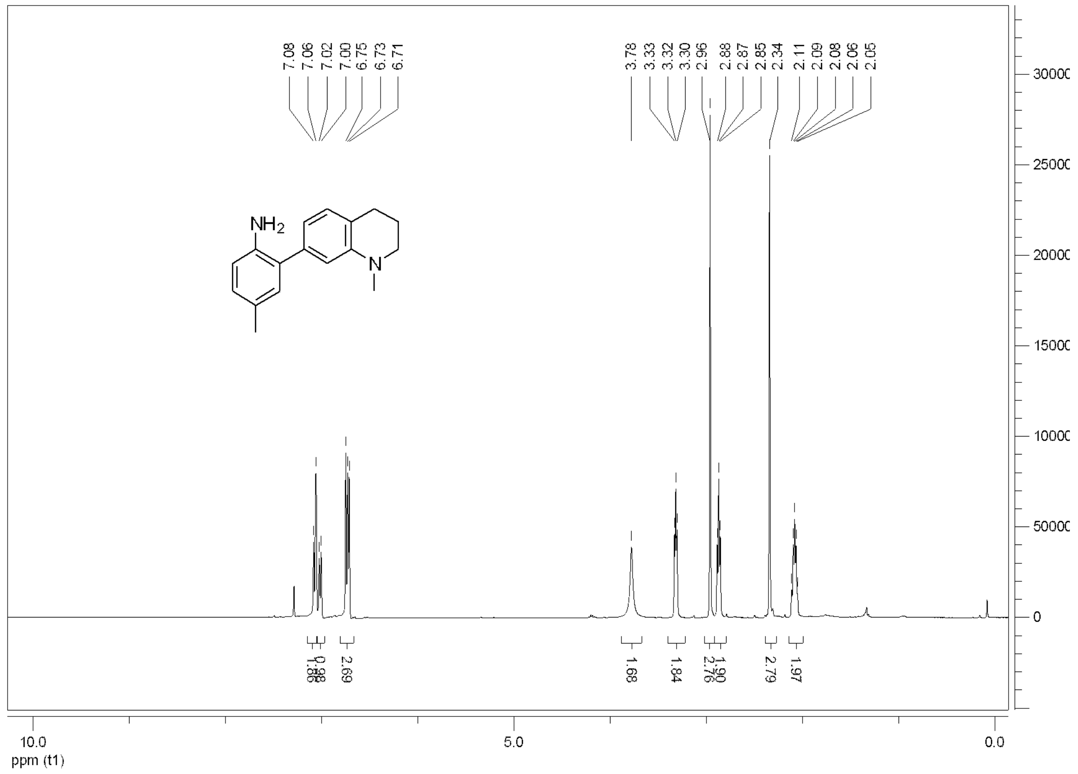

Figure S19: The ^{1}H-NMR spectrum of compound 5

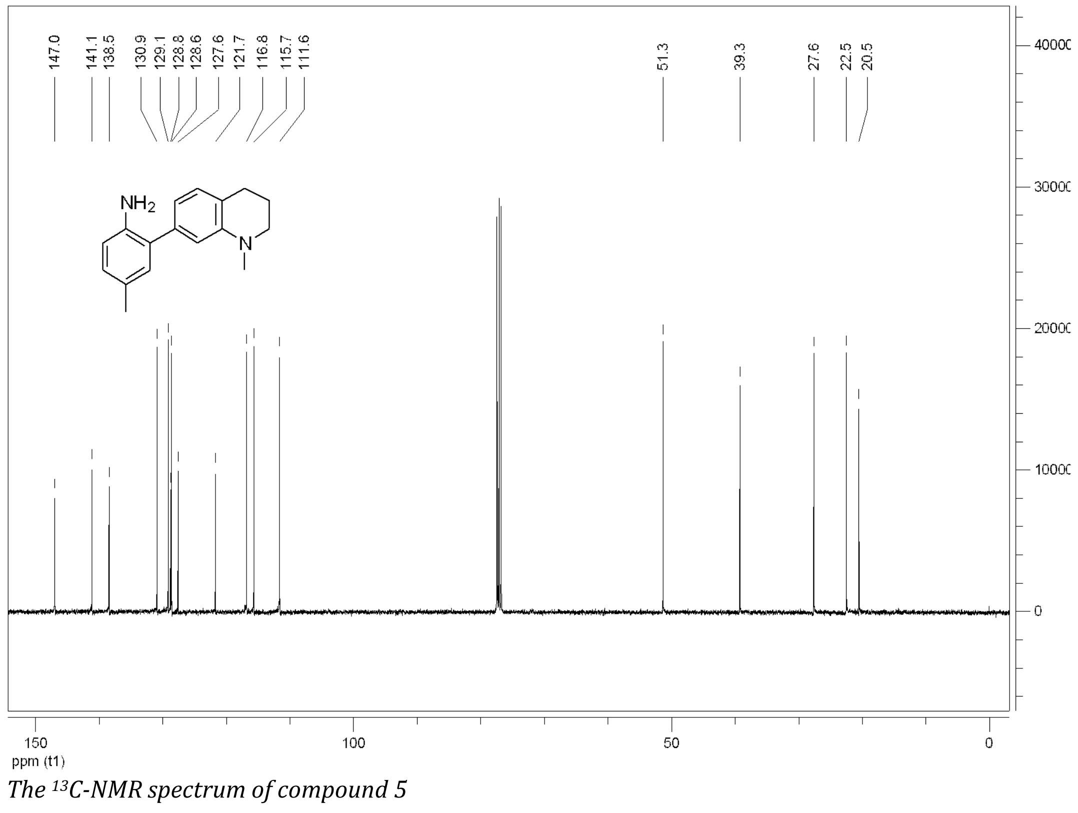

Figure S20: The ^{13}C-NMR spectrum of compound 5

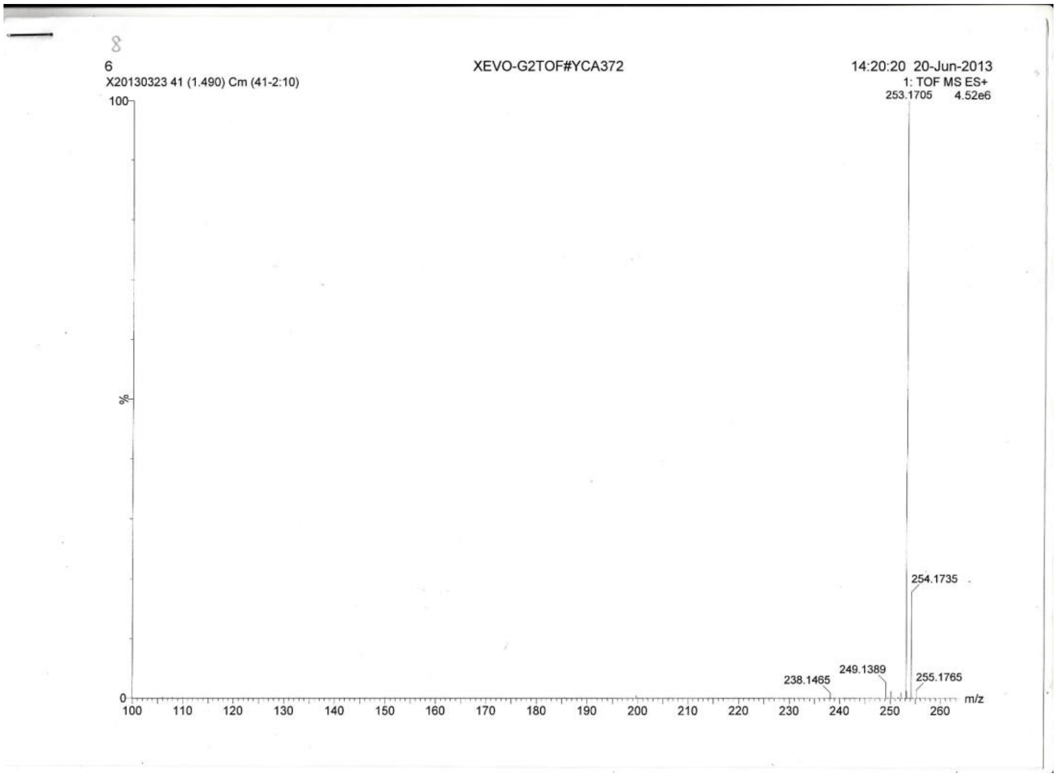

Figure S21: The HRMS spectrum of compound 5

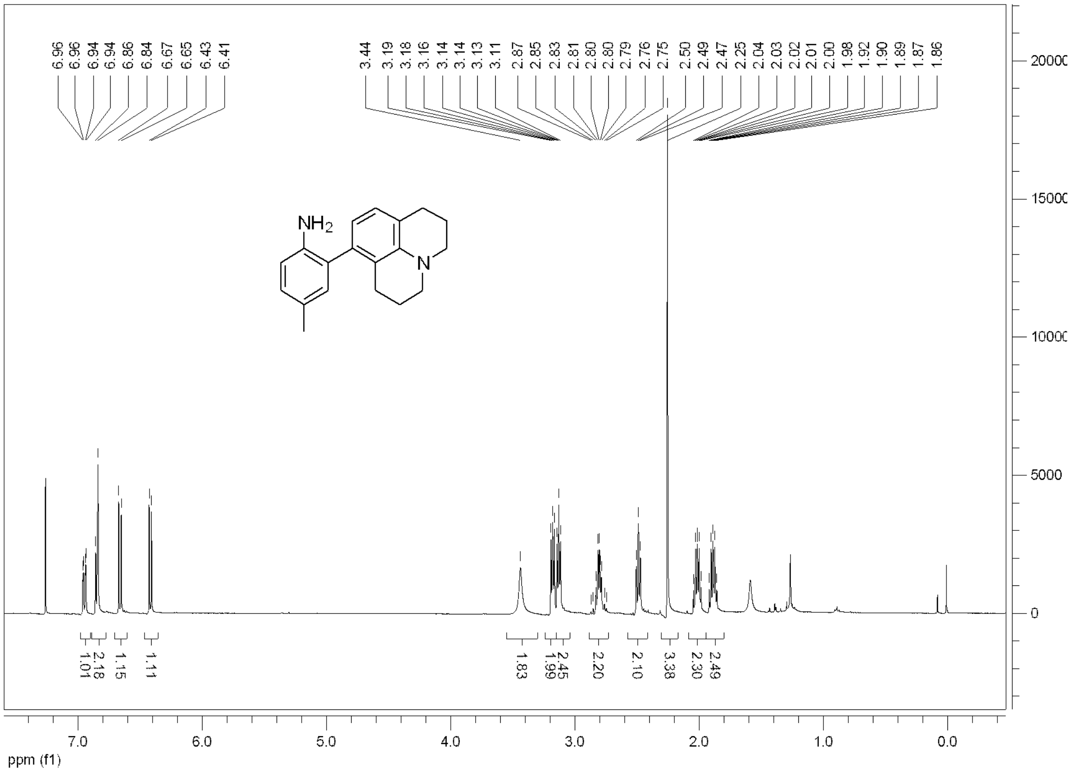

Figure S22: The ^{1}H-NMR spectrum of compound 6

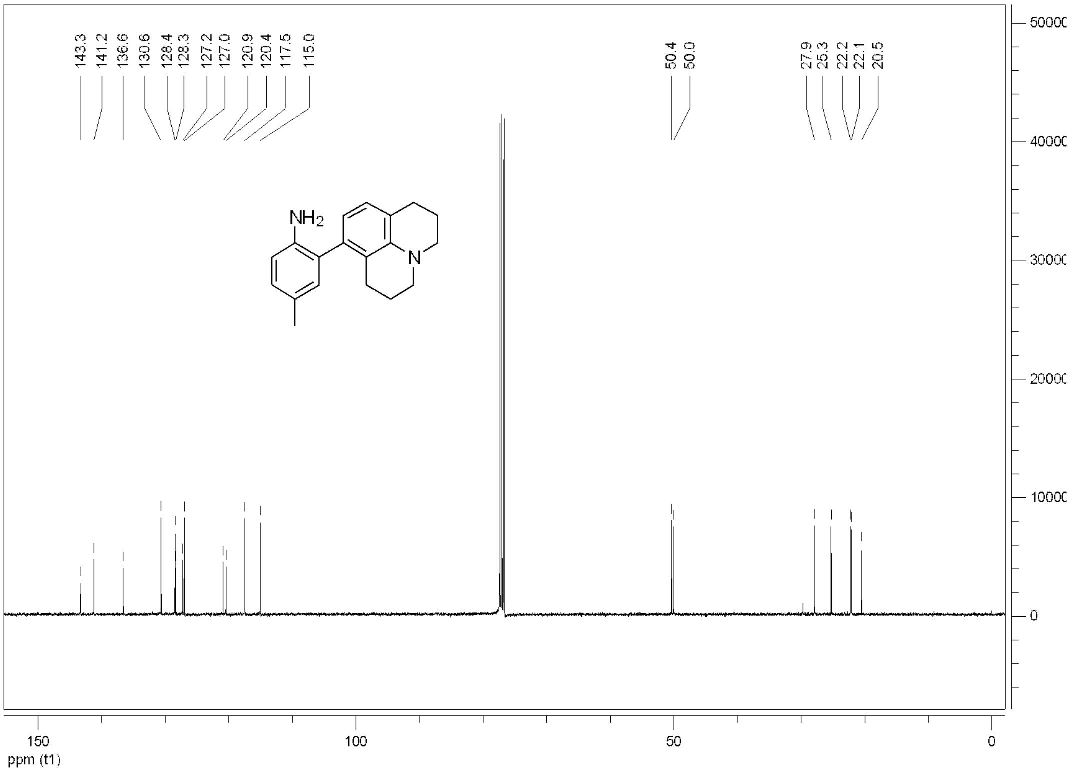

Figure S23: The ¹³C-NMR spectrum of compound 6

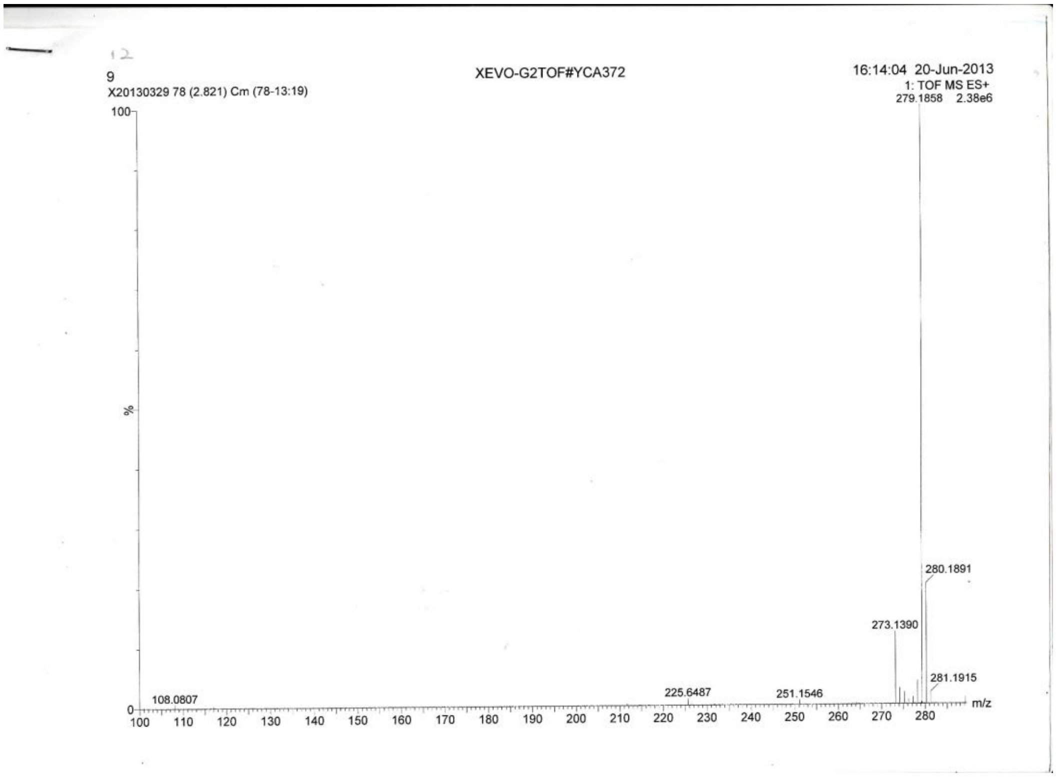

Figure S24: The HRMS spectrum of compound 6

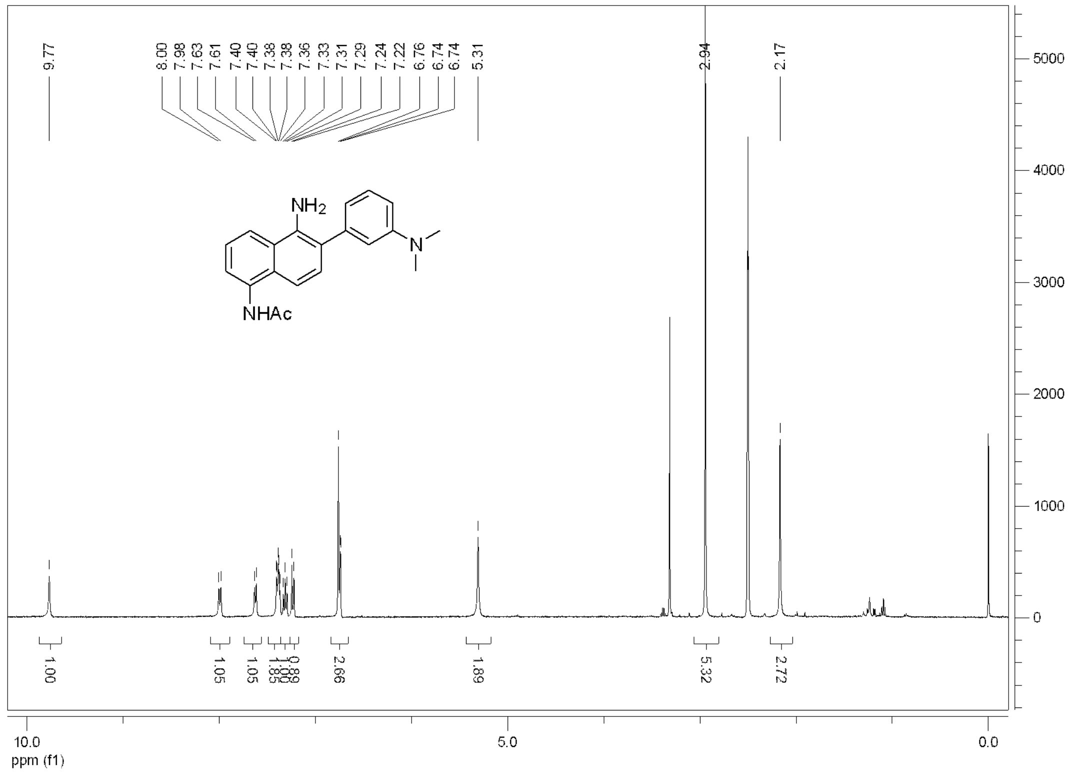

Figure S25: The ^{1}H-NMR spectrum of compound 7

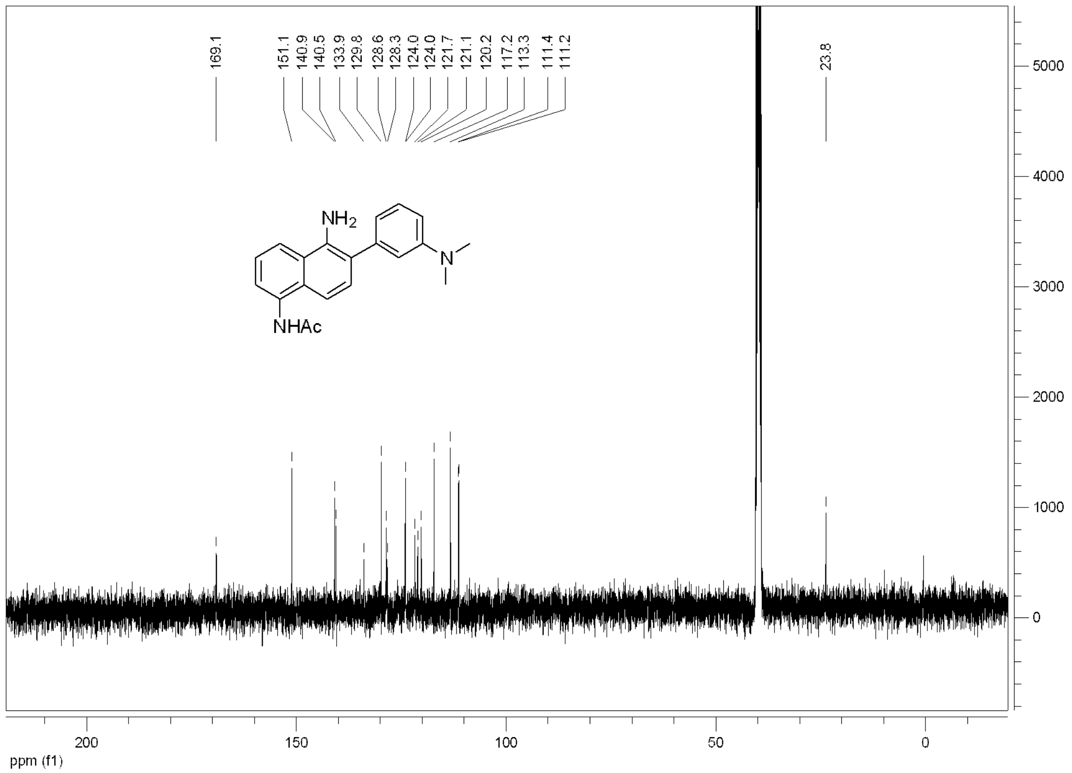

Figure S26: The ^{13}C-NMR spectrum of compound 7

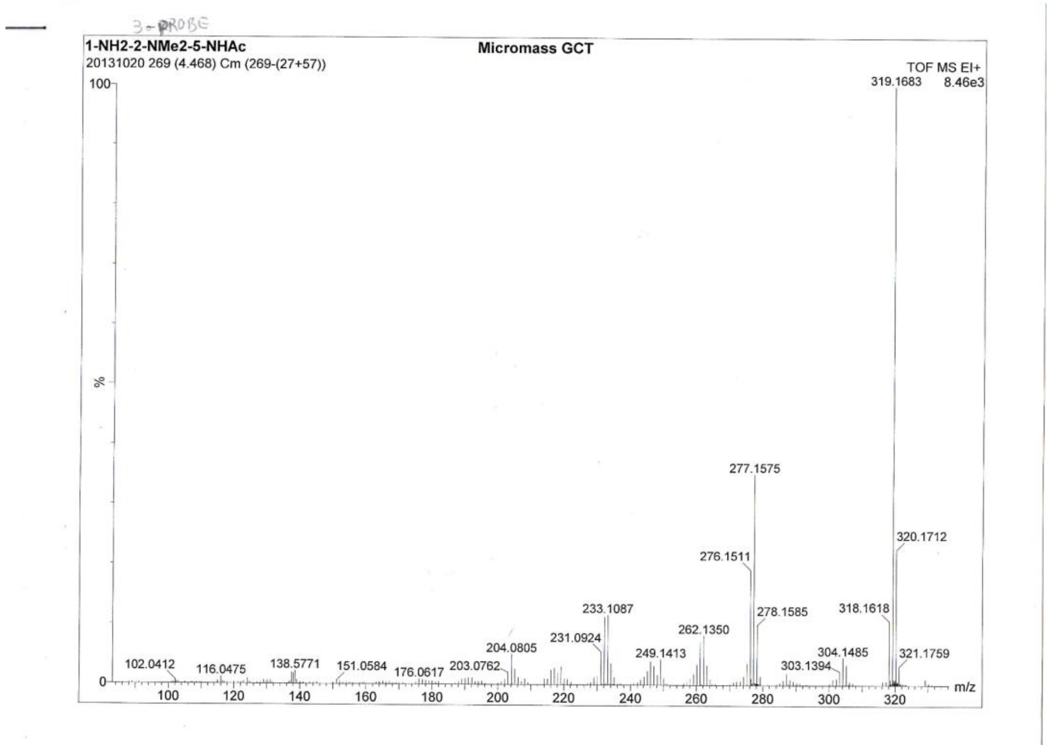

Figure S27: The HRMS spectrum of compound 7

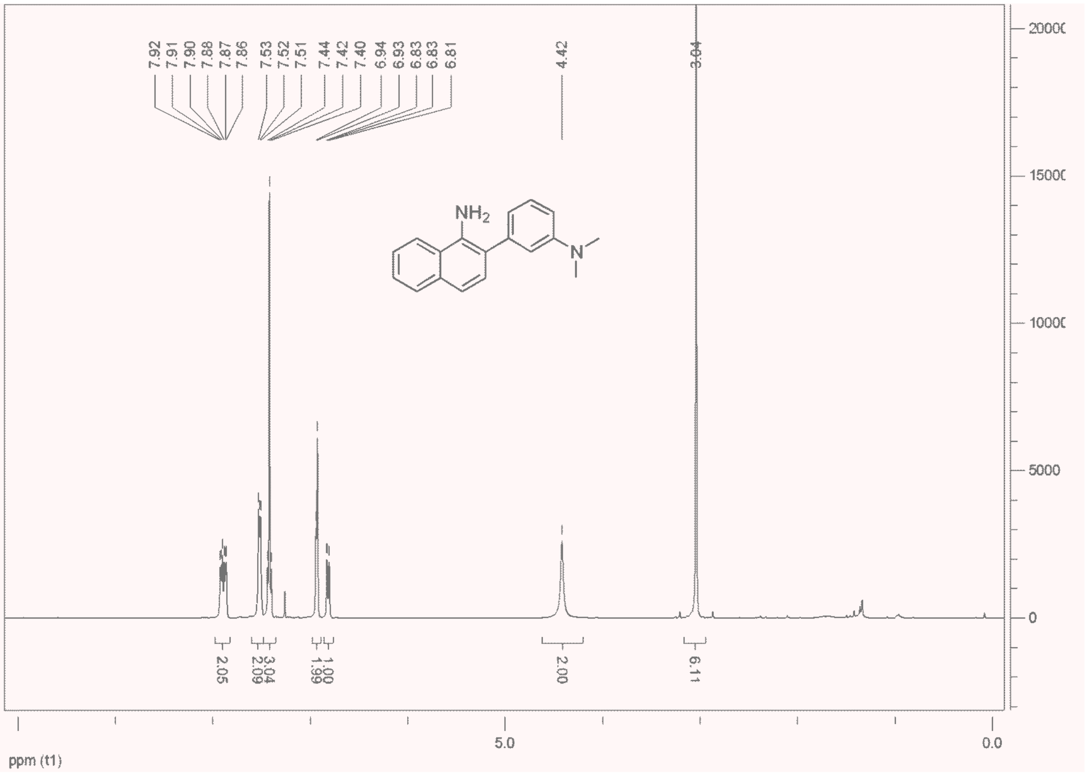

Figure S28: The ¹H-NMR spectrum of compound 8

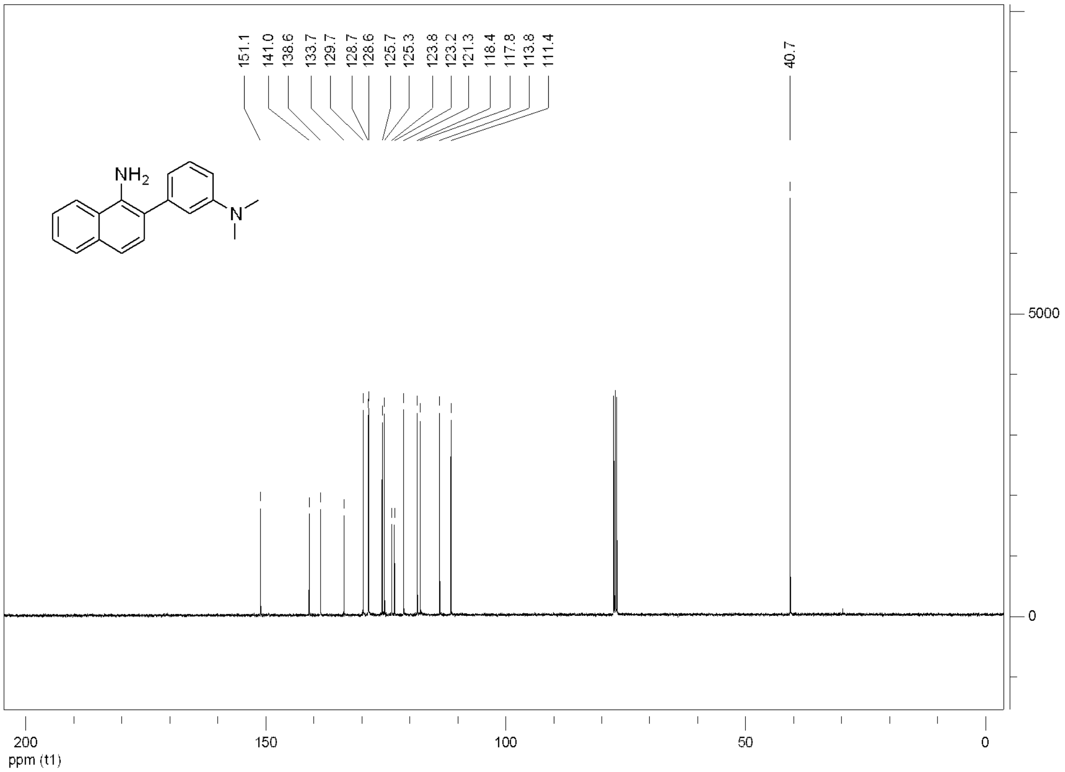

Figure S29: The ^{13}C-NMR spectrum of compound 8

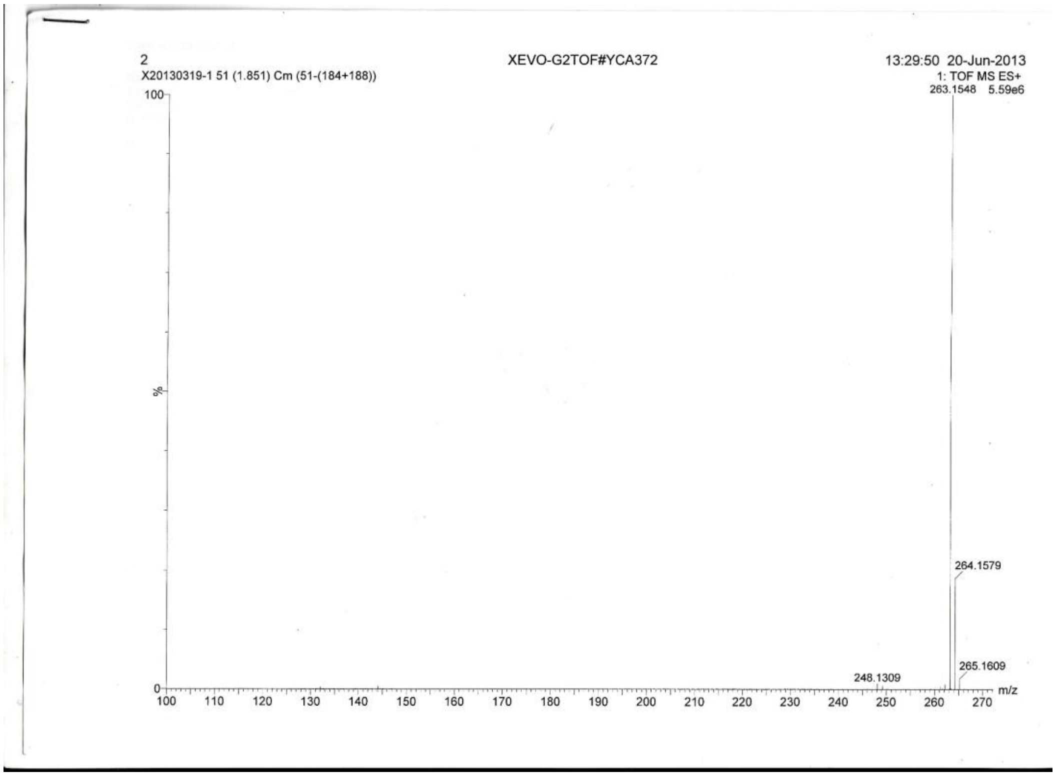

Figure S30: The HRMS spectrum of compound 8

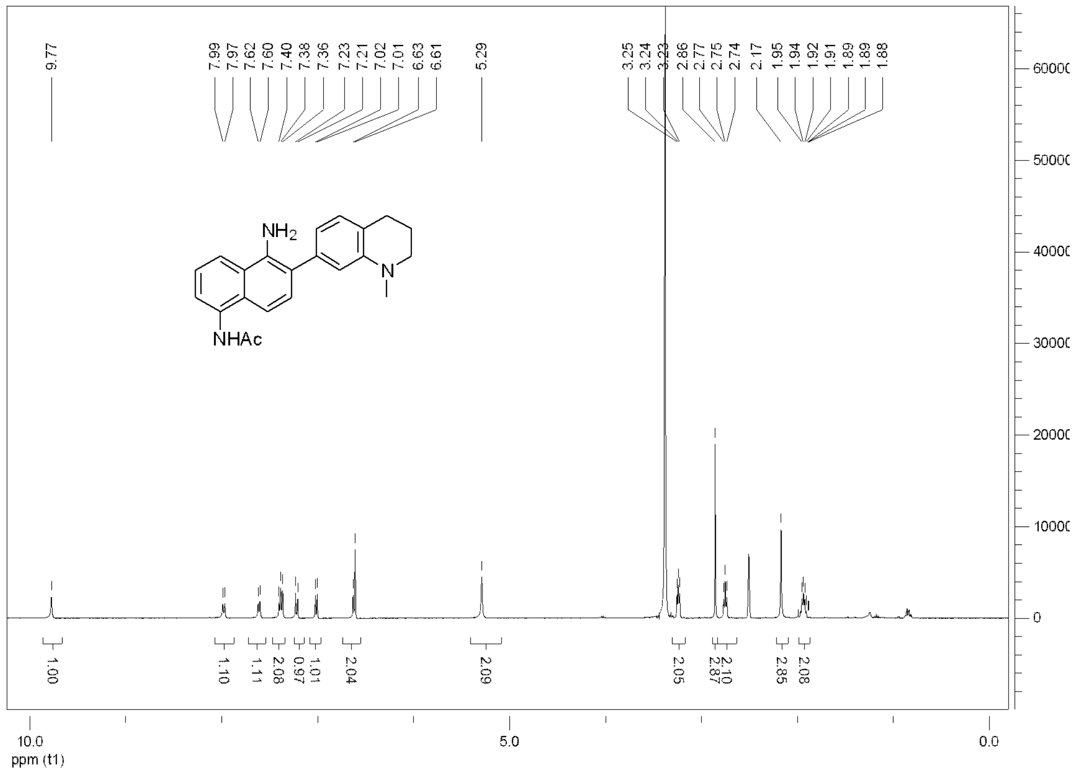

Figure S31: The ^{1}H-NMR spectrum of compound 10

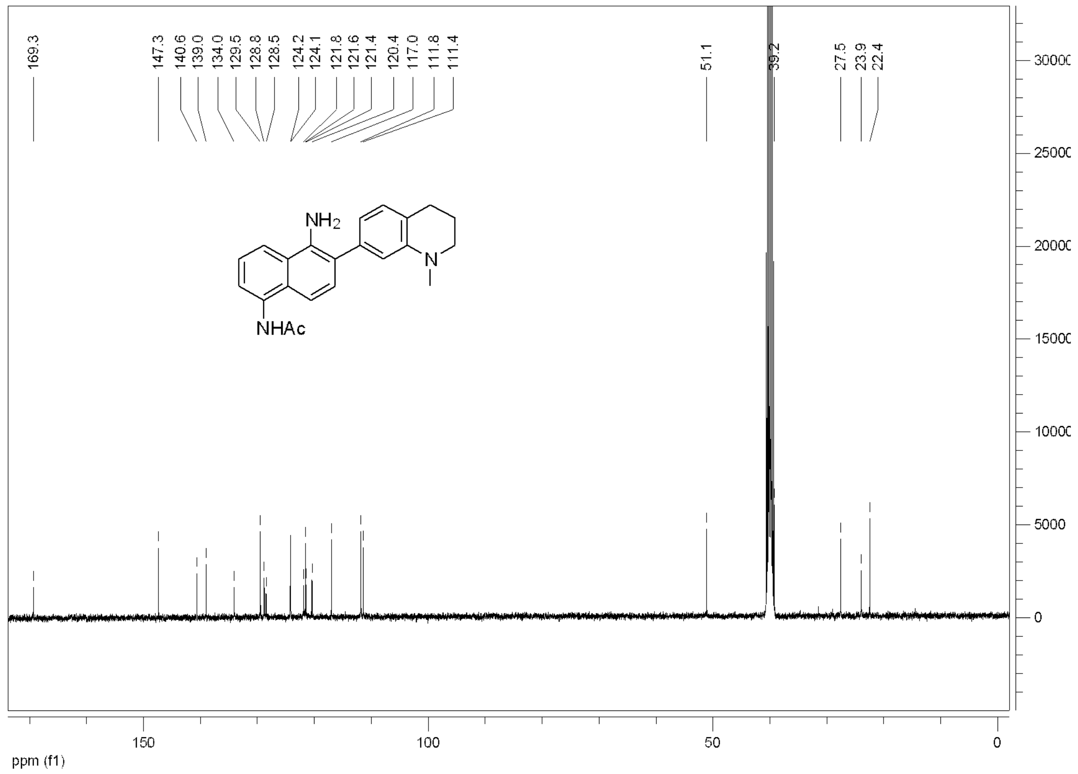

Figure S32: The ^{13}C-NMR spectrum of compound 10

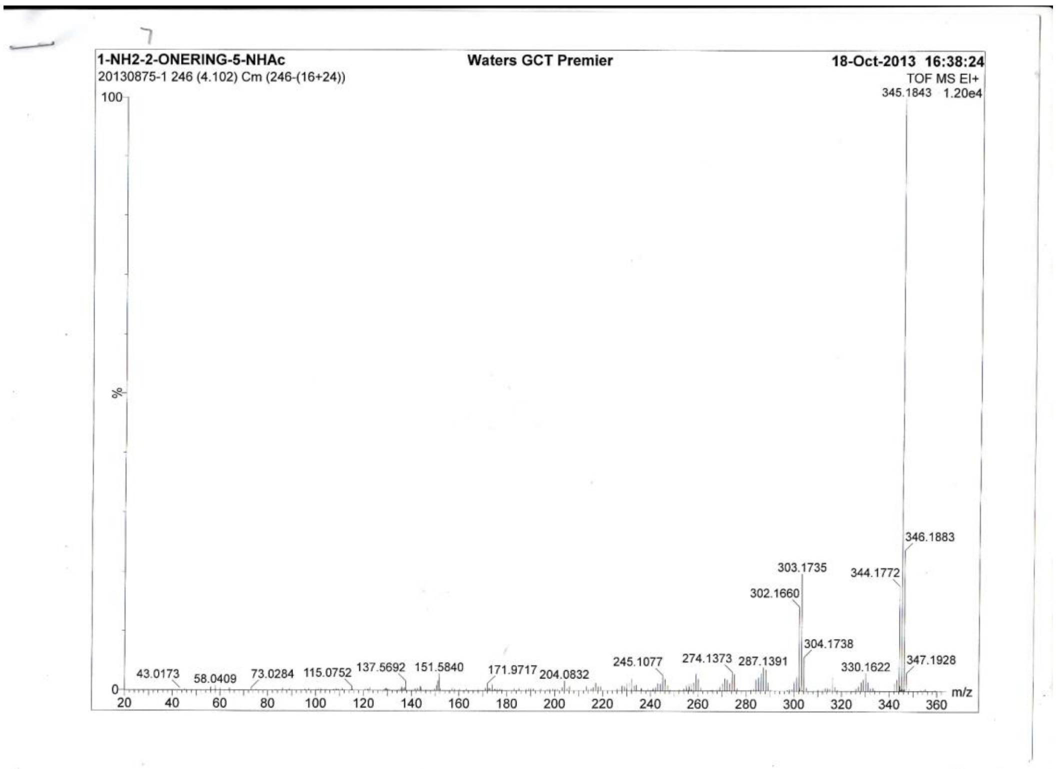

Figure S33: The HRMS spectrum of compound 10

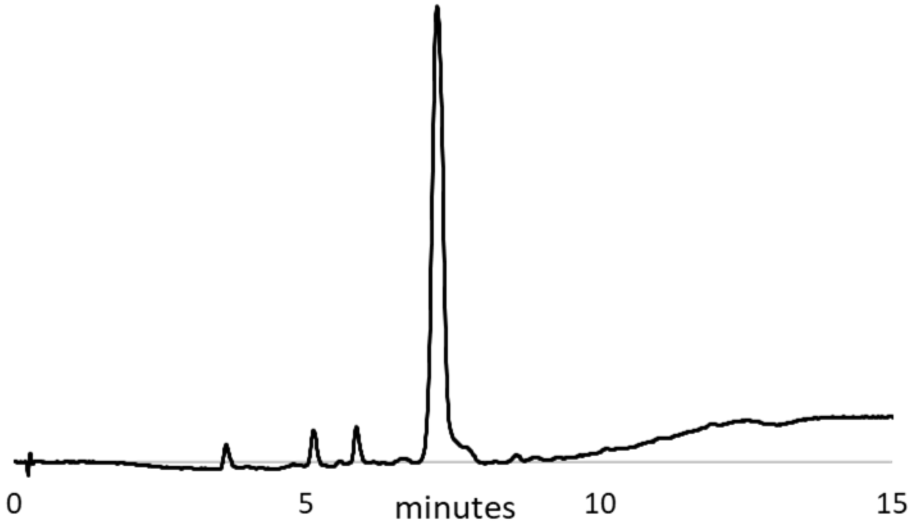

Figure S34: HPLC of compound 10 (280 nm)

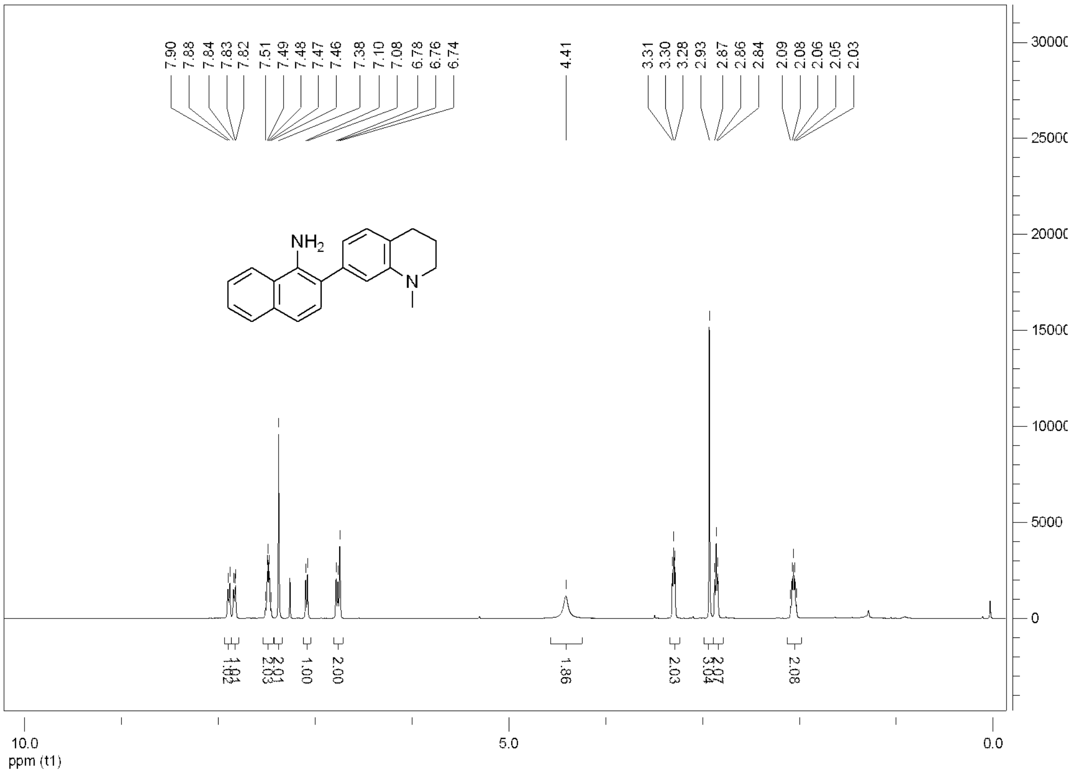

Figure S35: The ^{1}H-NMR spectrum of compound 11

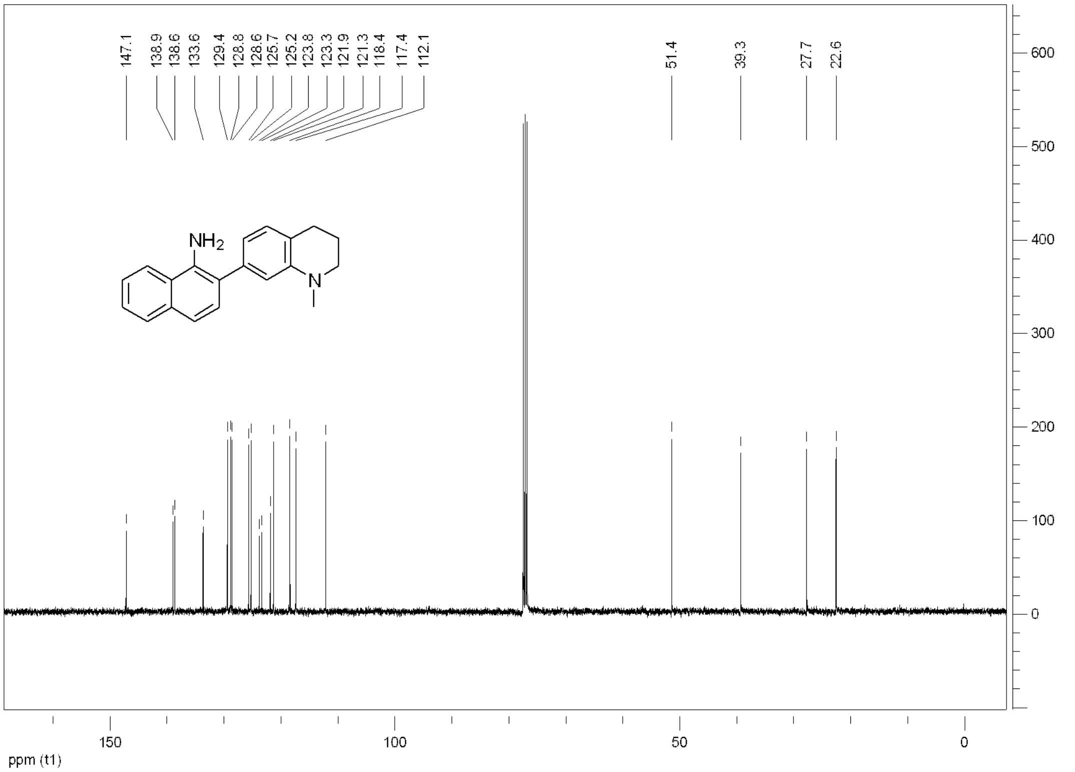

Figure S36: The ^{13}C-NMR spectrum of compound 11

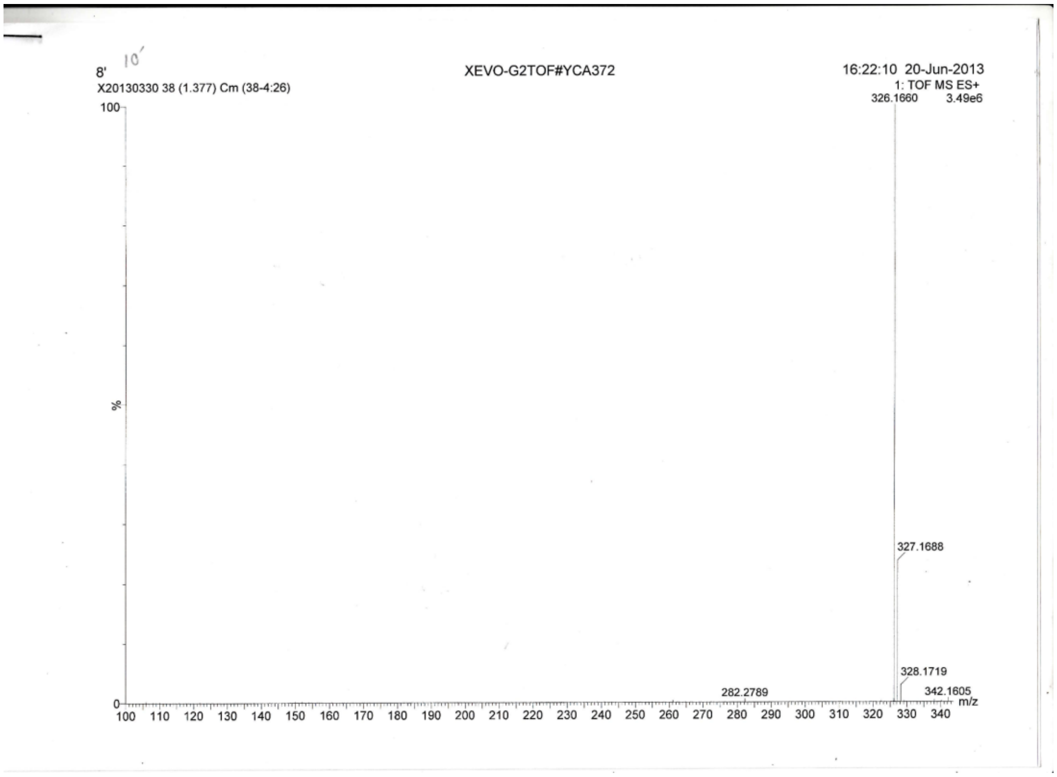

Figure S37: The HRMS spectrum of compound 11

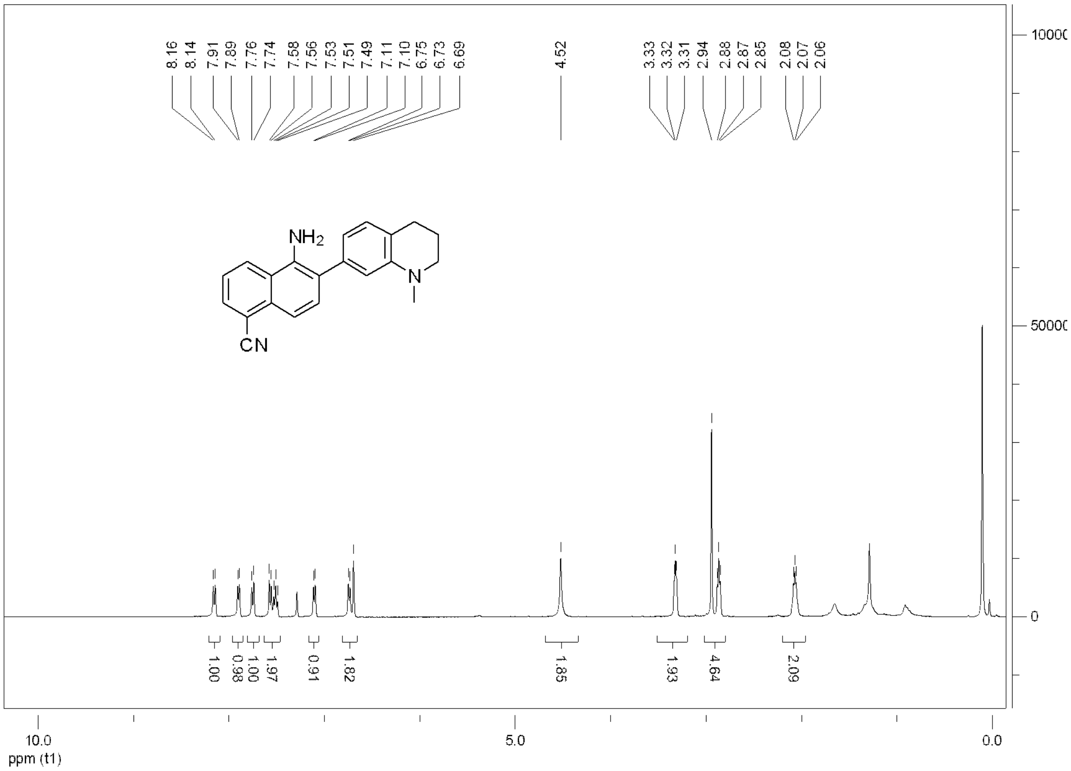

Figure S38: The 1H-NMR spectrum of compound 12

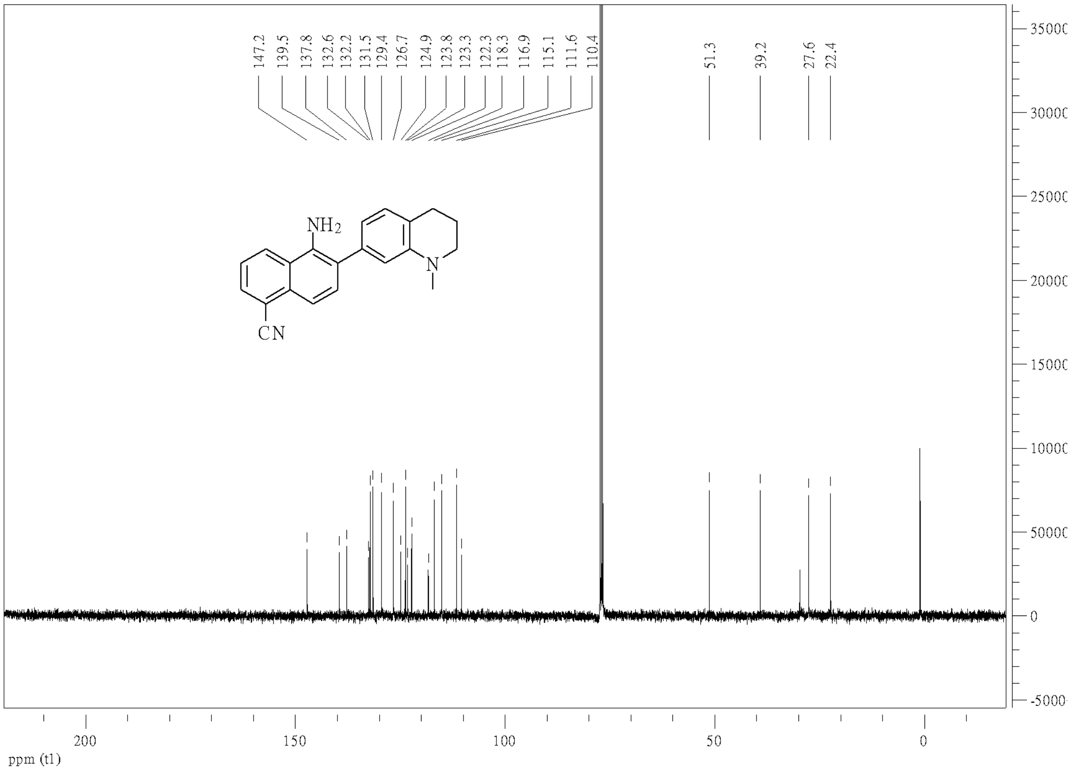

Figure S39: The ^{13}C-NMR spectrum of compound 12

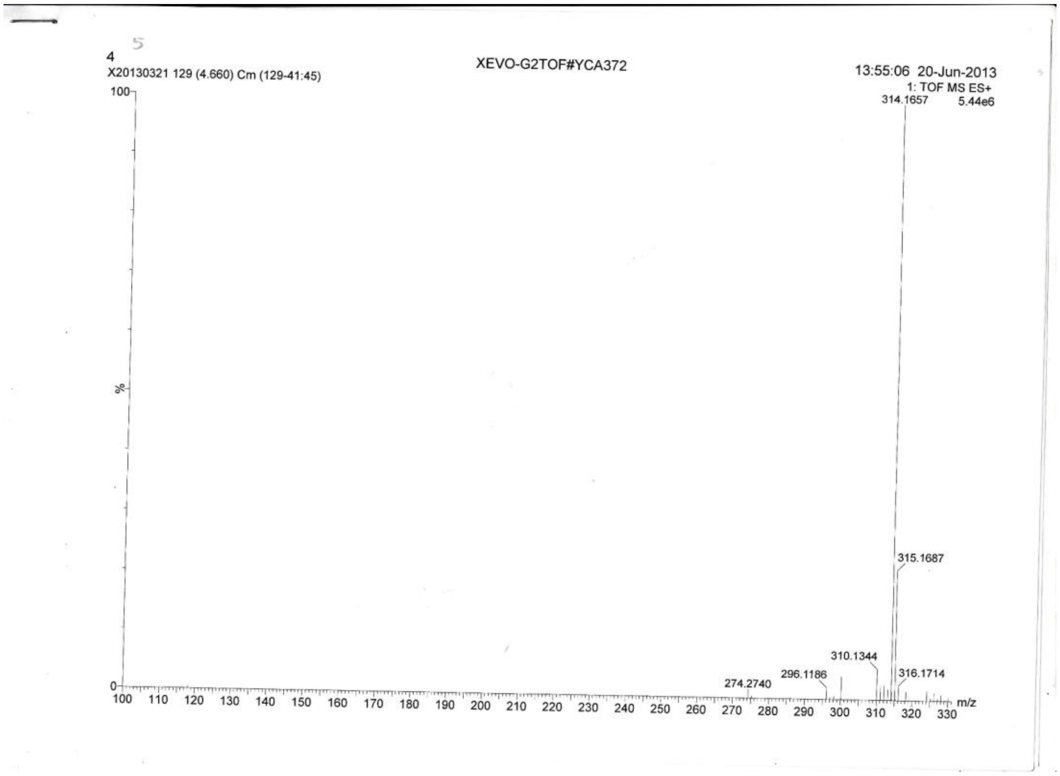

Figure S40: The HRMS spectrum of compound 12

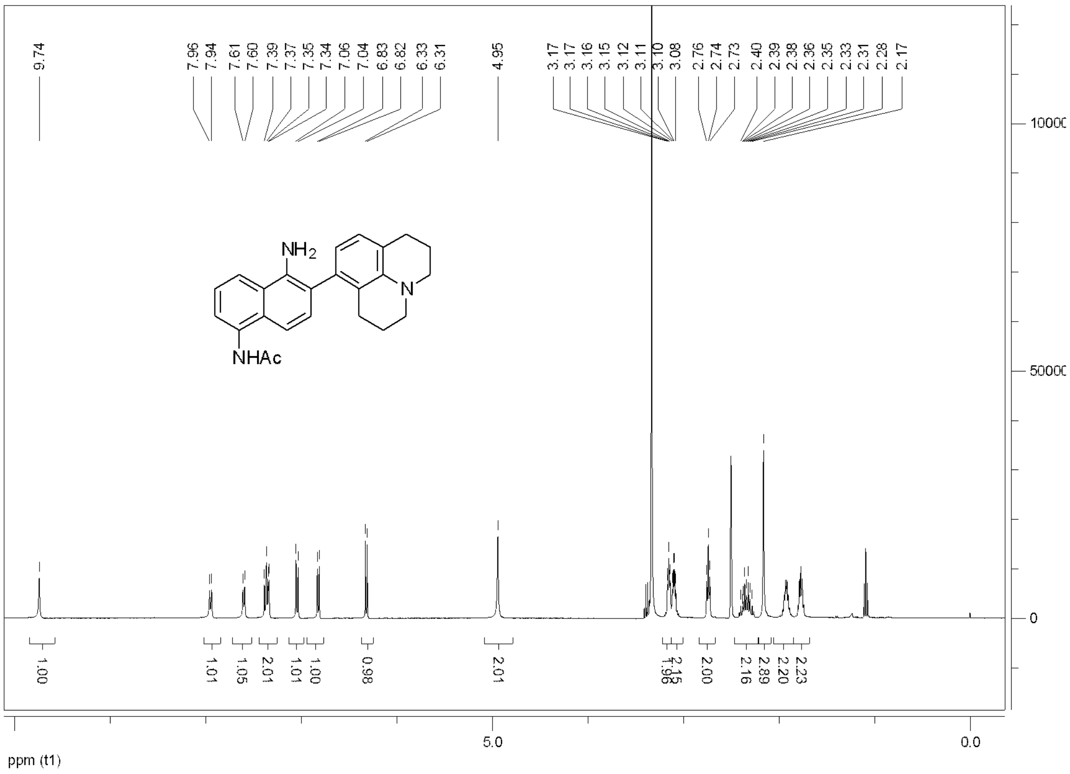

Figure S41: The ^{1}H-NMR spectrum of compound 13

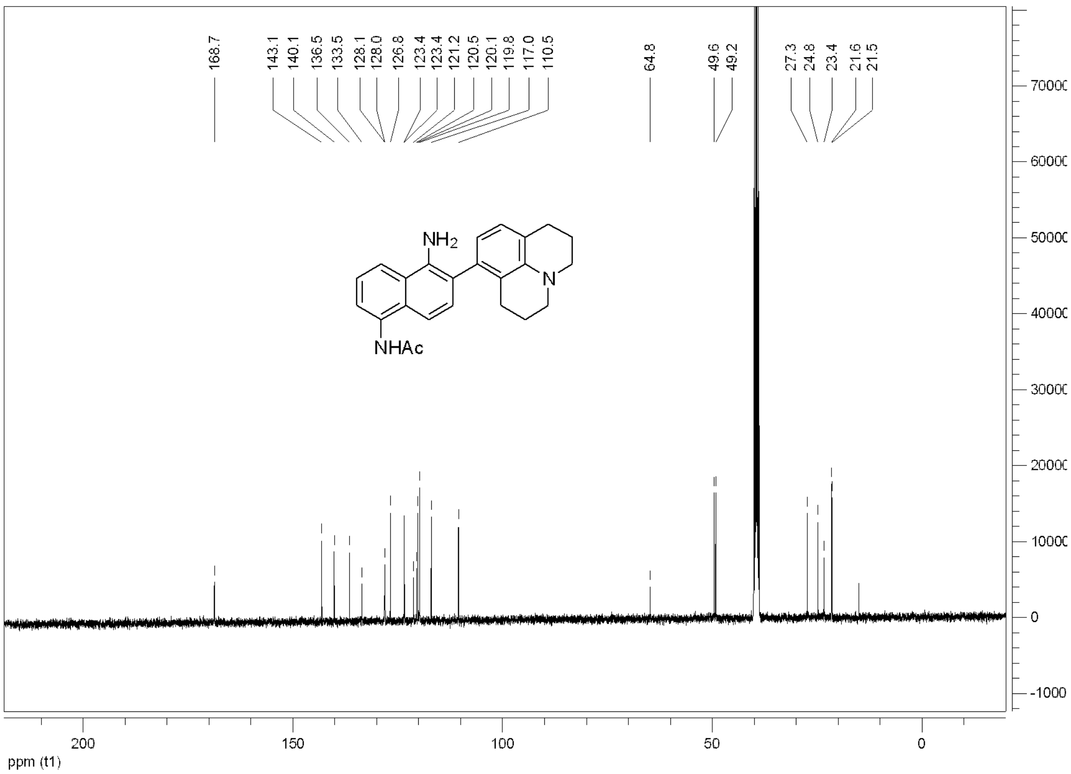

Figure S42: The ^{13}C-NMR spectrum of compound 13

Elemental Composition Report **Page 1**

Single Mass Analysis
Tolerance = 30.0 mDa / DBE: min = -1.5, max = 100.0
Element prediction: Off
Number of isotope peaks used for i-FIT = 2

Monoisotopic Mass, Even Electron Ions
130 formula(e) evaluated with 7 results within limits (up to 1 closest results for each mass)
Elements Used:
C: 0-50 H: 0-50 N: 0-4 O: 0-5

ZHANG-WB ECUST institute of Fine Chem 07-Mar-2014 21:09:59

ZWB-THY-11 45 (1.482) Cm (45:50) 1: TOF MS ES+ 2.40e+003

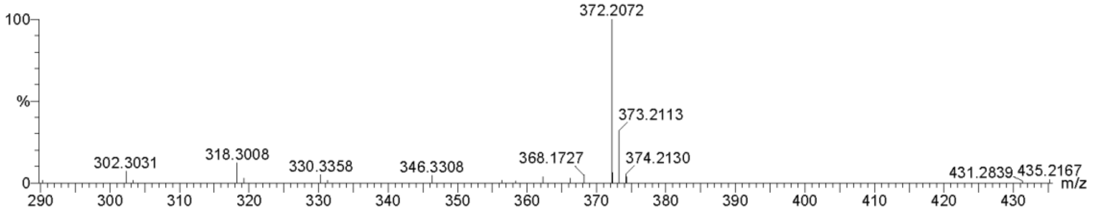

Minimum:				-1.5			
Maximum:		30.0	50.0	100.0			
Mass	Calc. Mass	mDa	PPM	DBE	i-FIT	i-FIT (Norm)	Formula
372.2072	372.2076	-0.4	-1.1	13.5	25.0	0.0	C24 H26 N3 O

Figure S43: The HRMS spectrum of compound 13

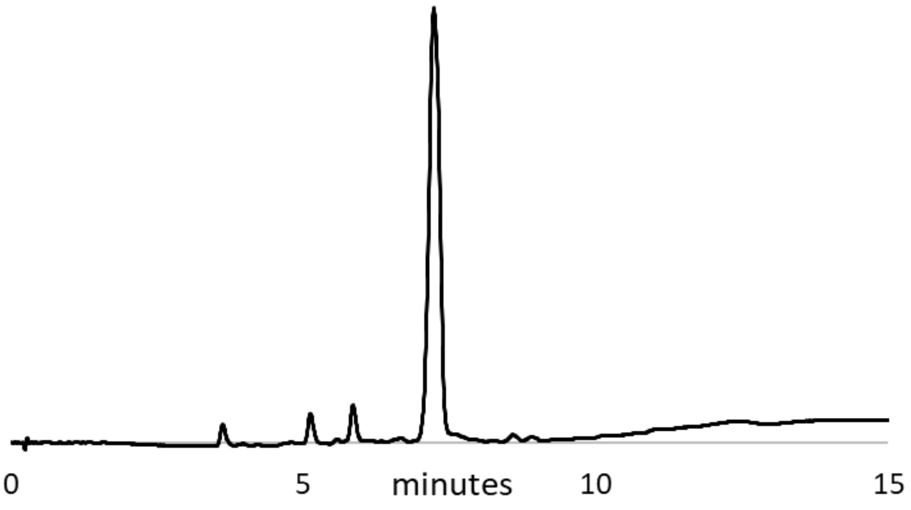

Figure S44: HPLC of Compound 13 (280 nm)

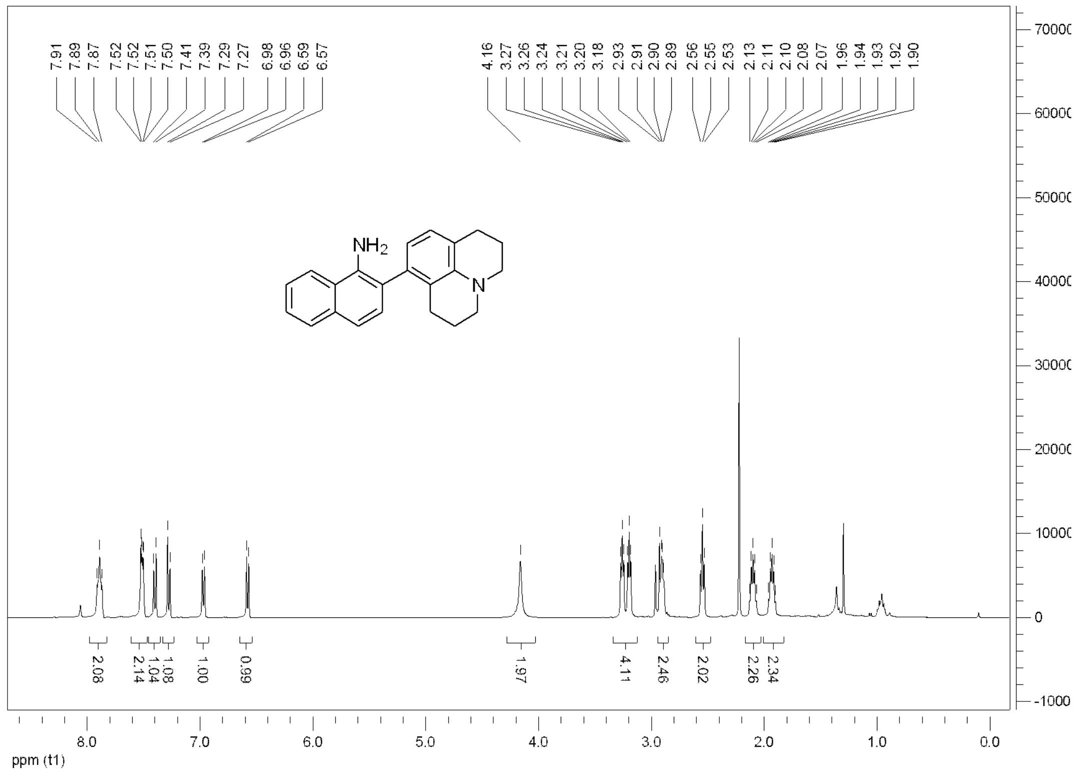

Figure S45: The ¹H-NMR spectrum of compound 14

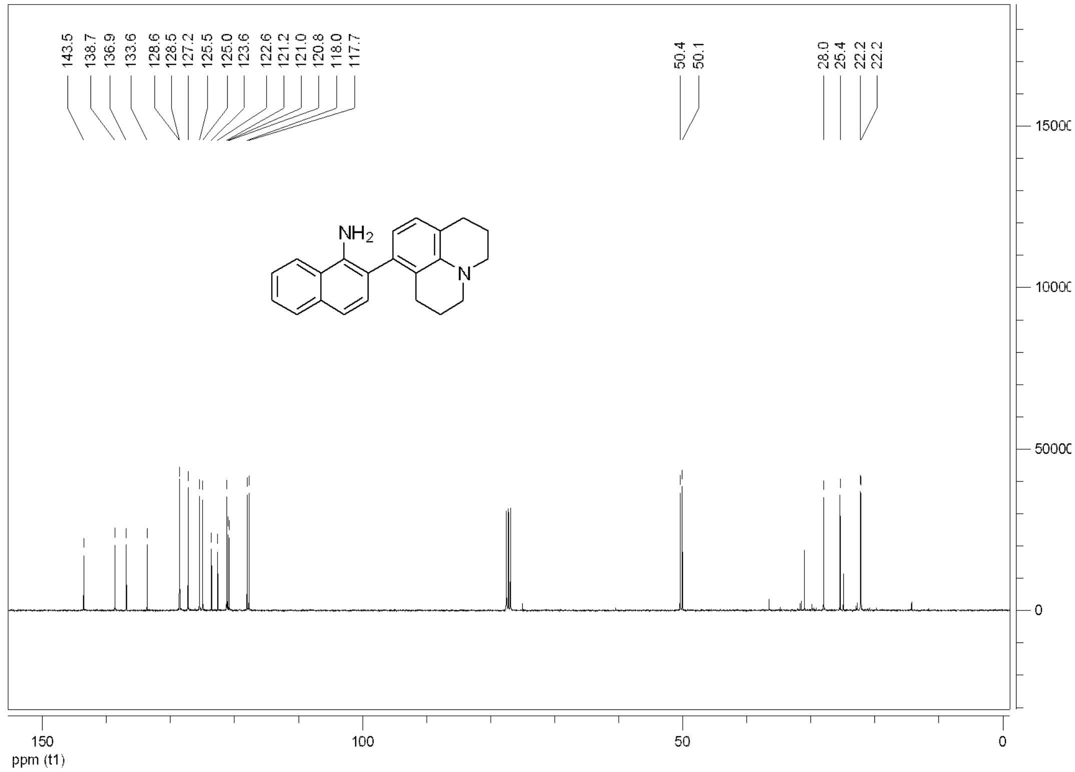

Figure S46: The ¹³C-NMR spectrum of compound 14

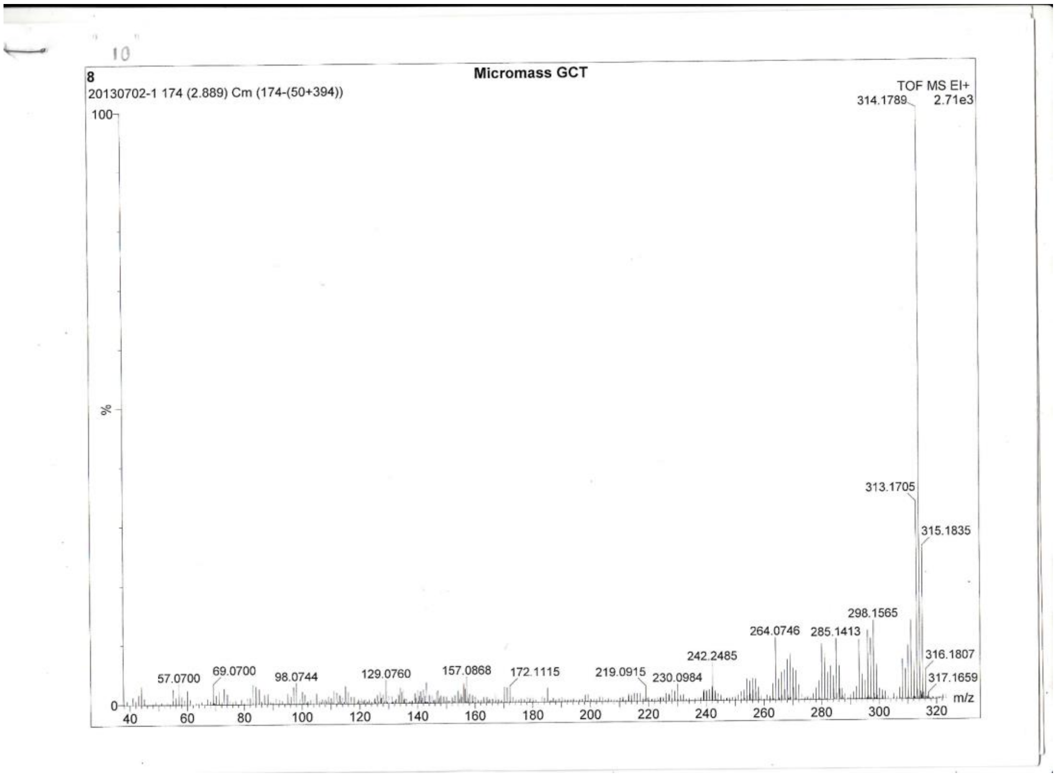

Figure S47: The HRMS spectrum of compound 14

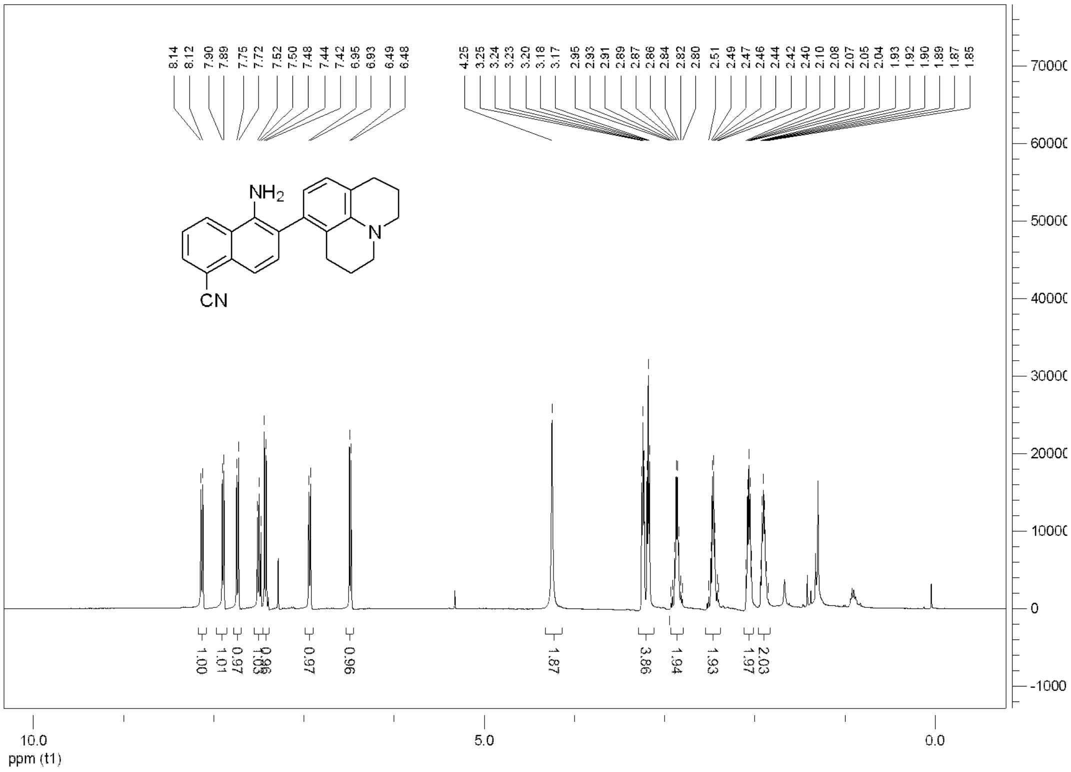

Figure S48: The ^{1}H-NMR spectrum of compound 15

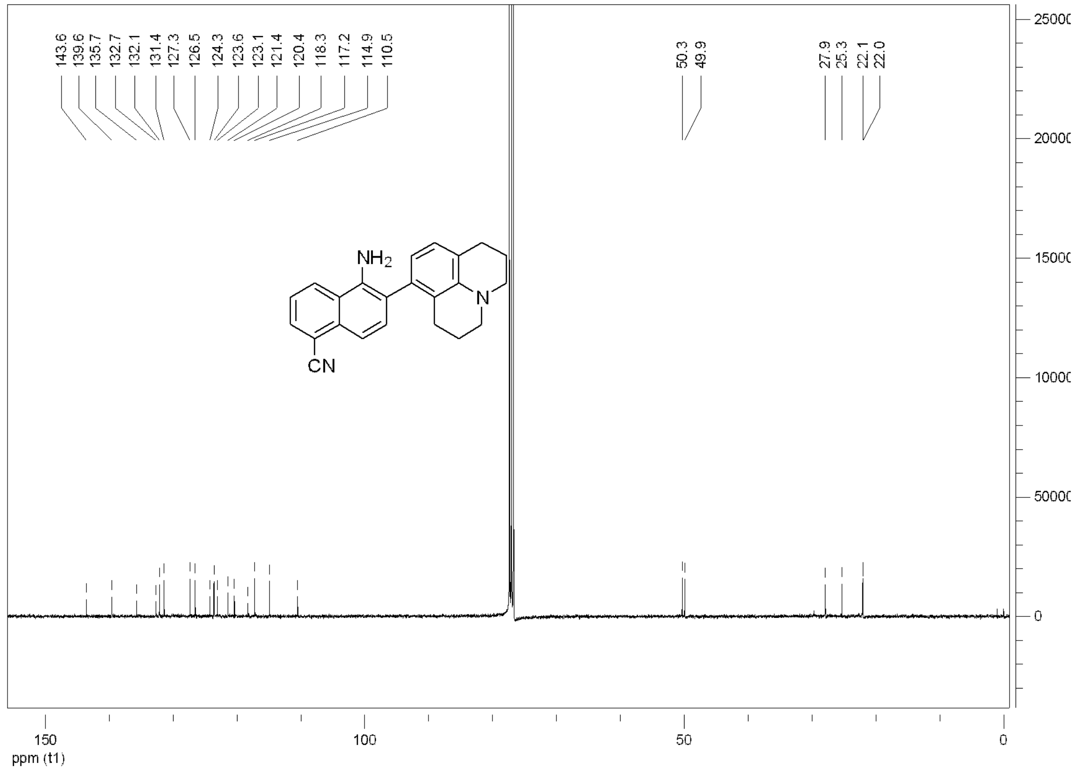

Figure S49: The ^{13}C-NMR spectrum of compound 15

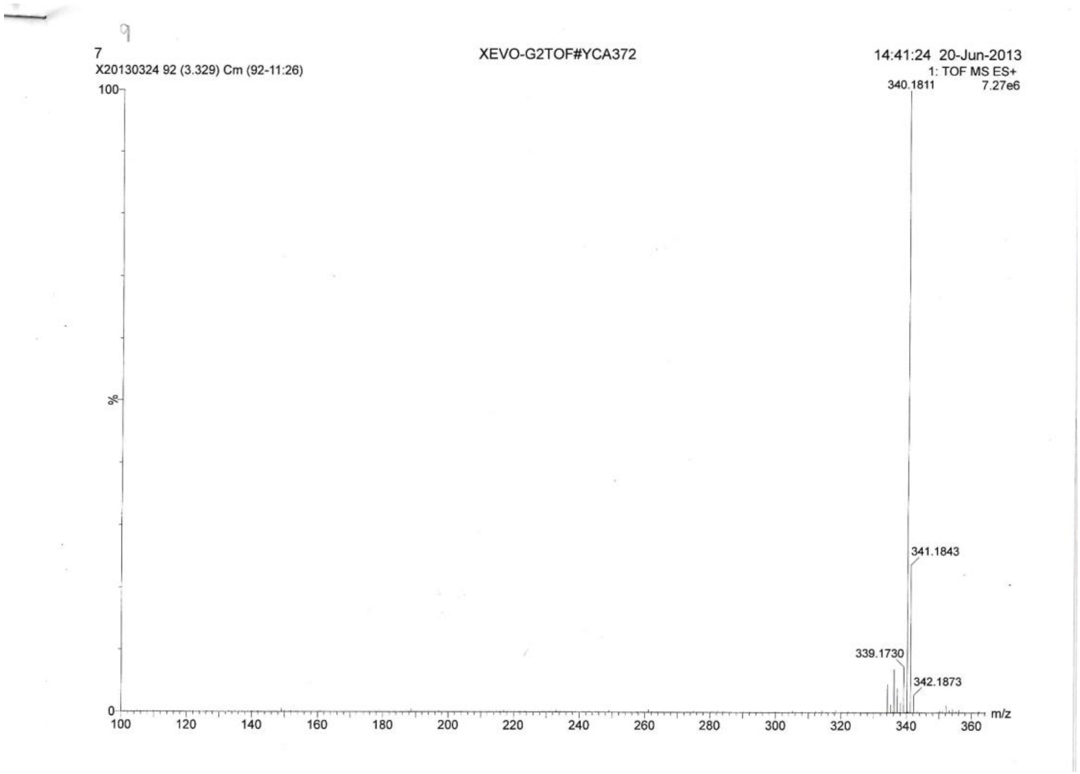

Figure S50: The HRMS spectrum of compound 15

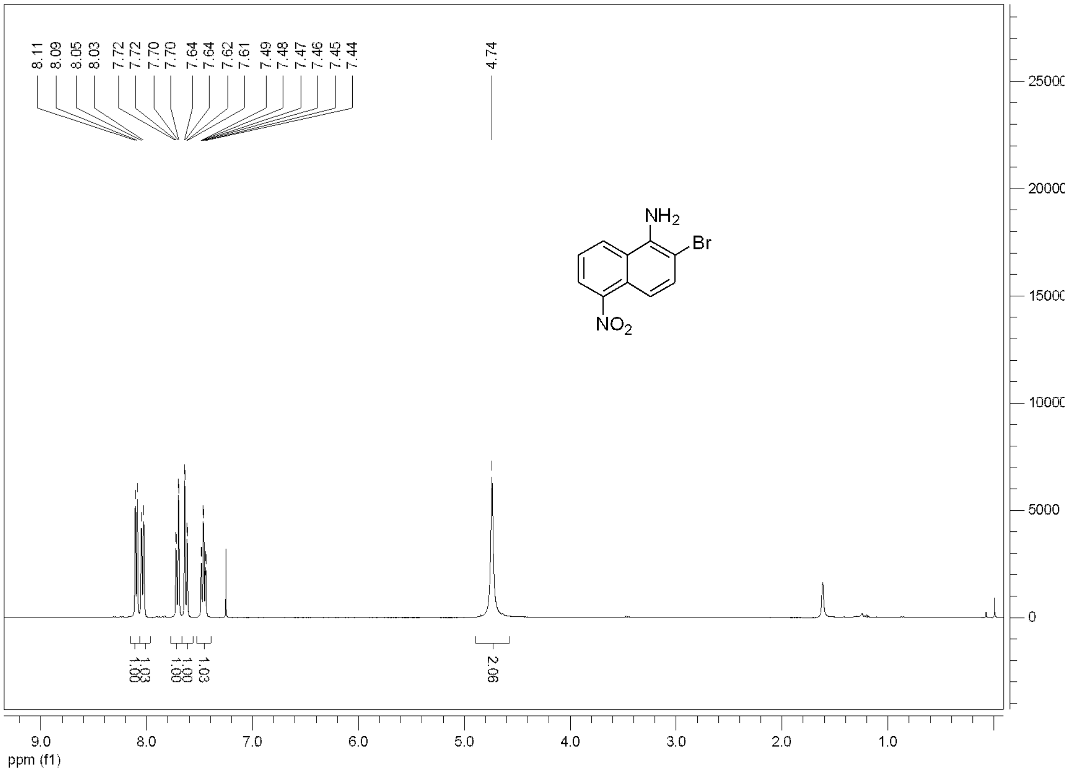

Figure S51: The ^{1}H-NMR spectrum of compound 16

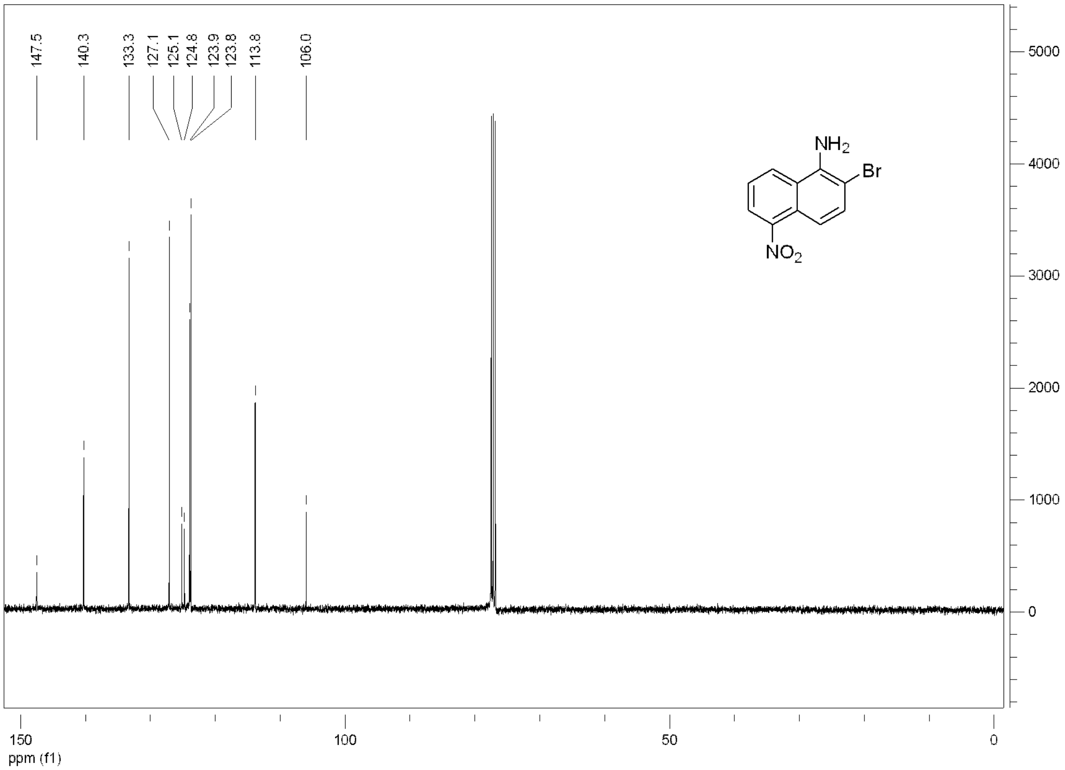

Figure S52: The ^{13}C-NMR spectrum of compound 16

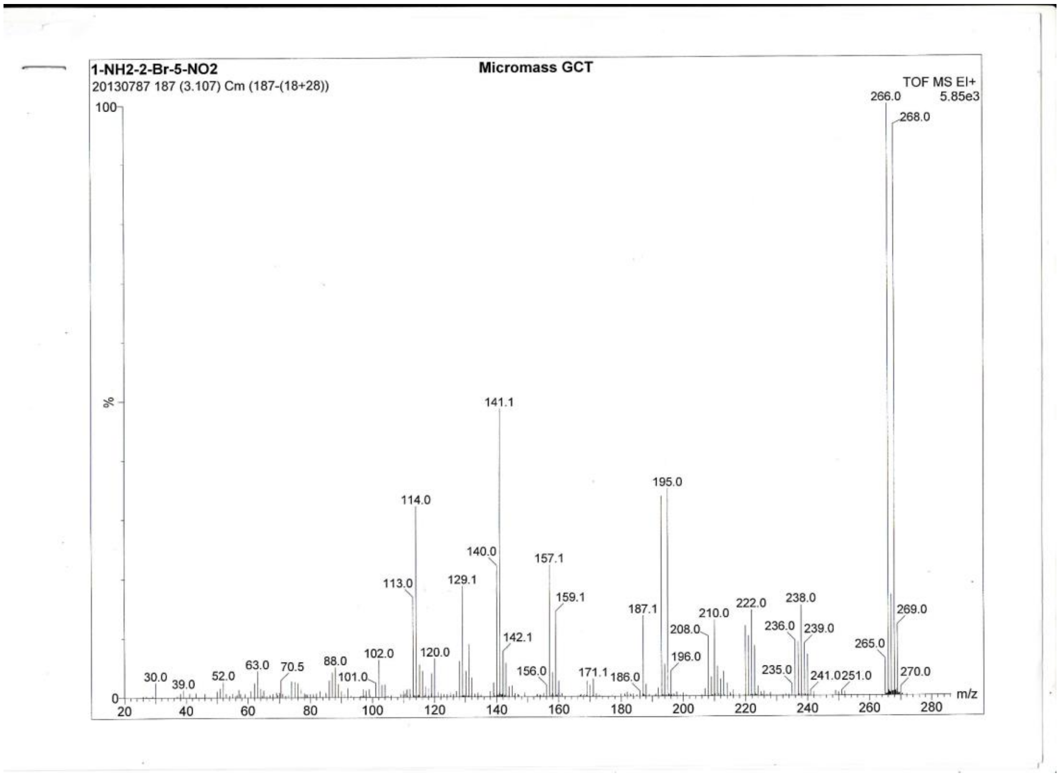

Figure S53: The MS spectrum of compound 16

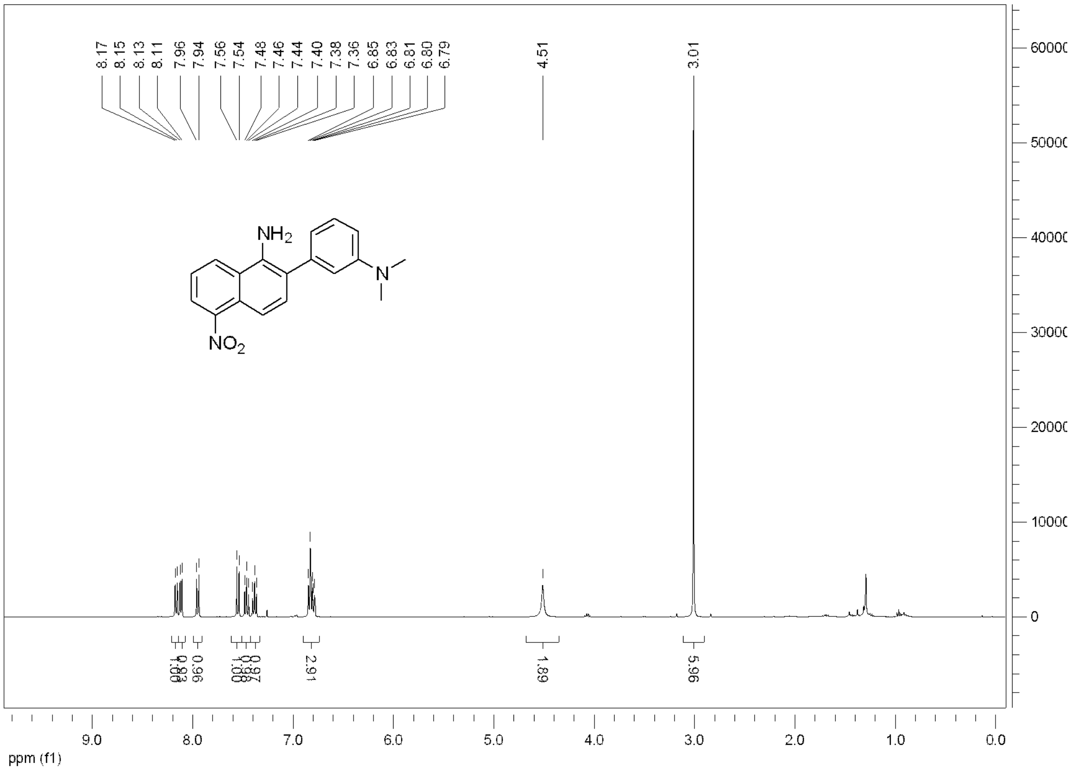

Figure S54: The ¹H-NMR spectrum of compound 17

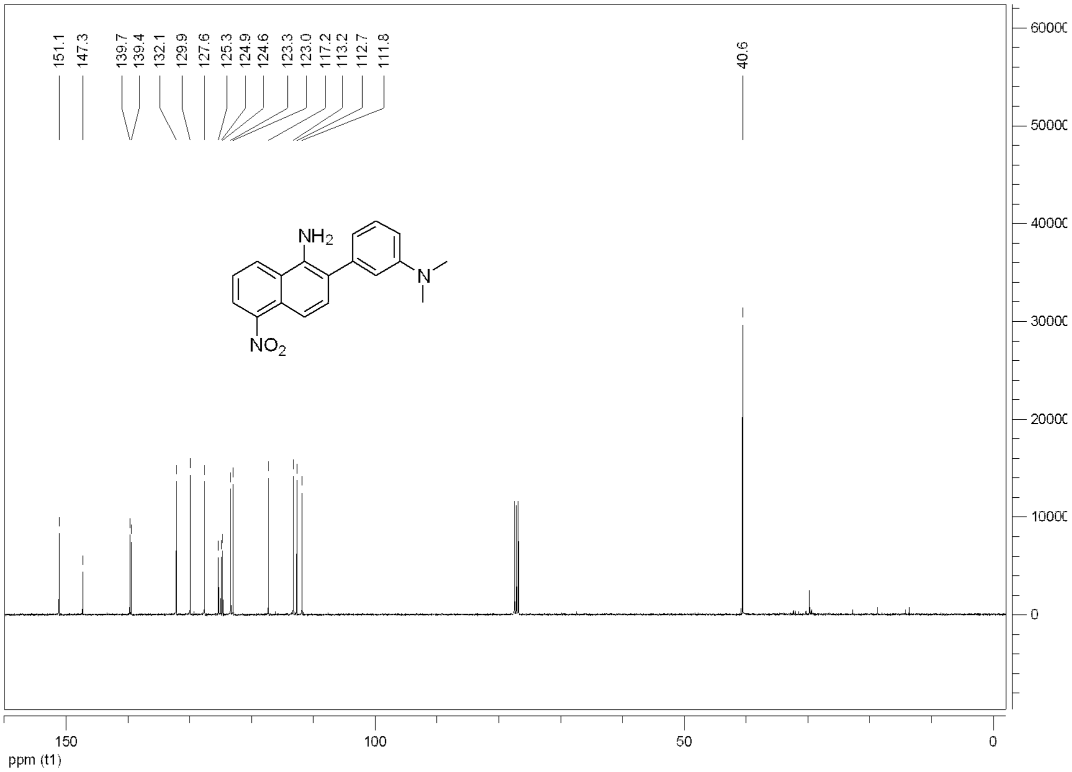

Figure S55: The ^{13}C-NMR spectrum of compound 17

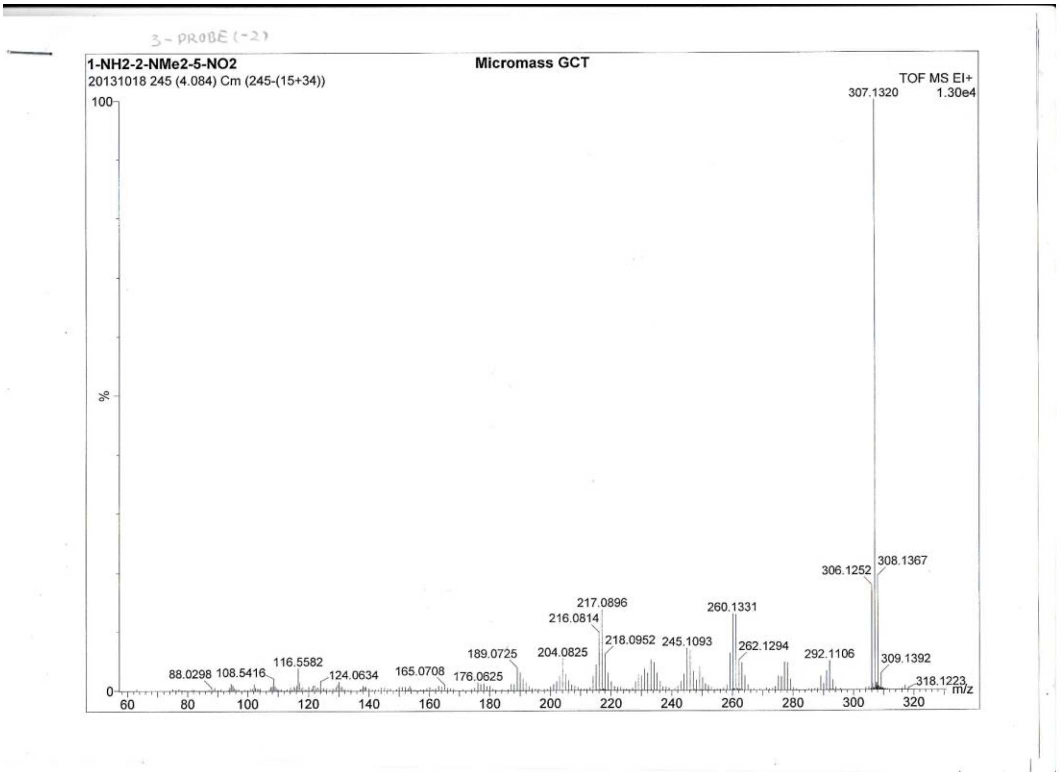

Figure S56: The HRMS spectrum of compound 17

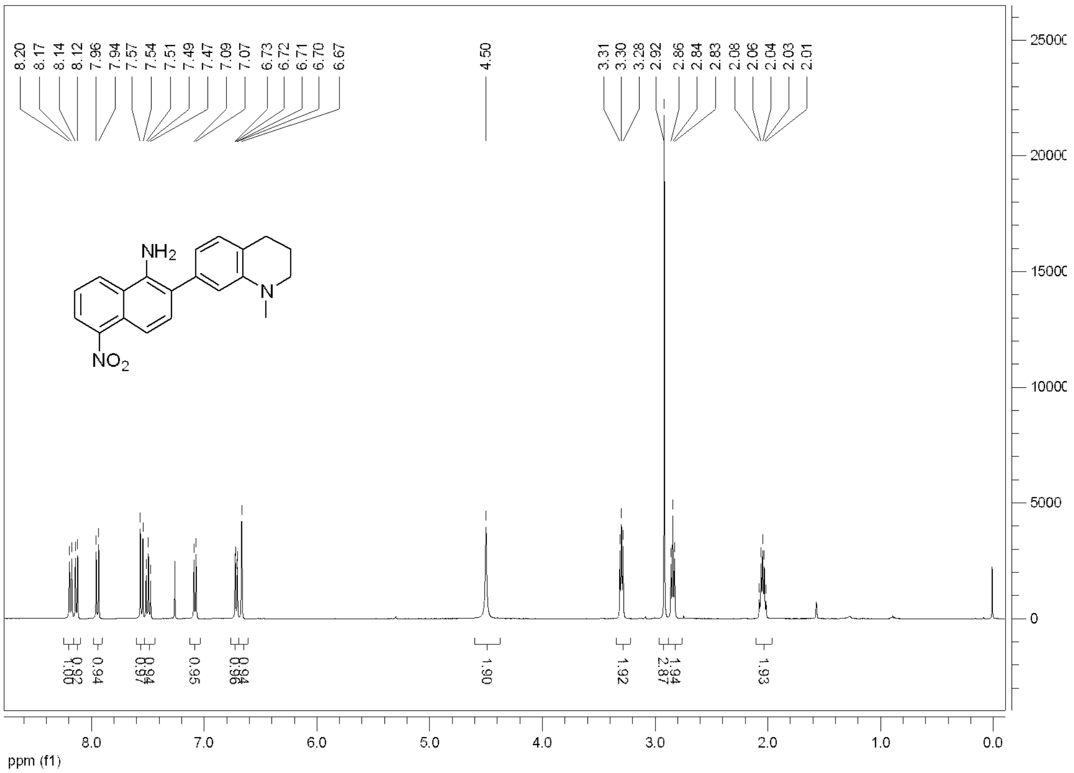

Figure S57: The ^{1}H-NMR spectrum of compound 18

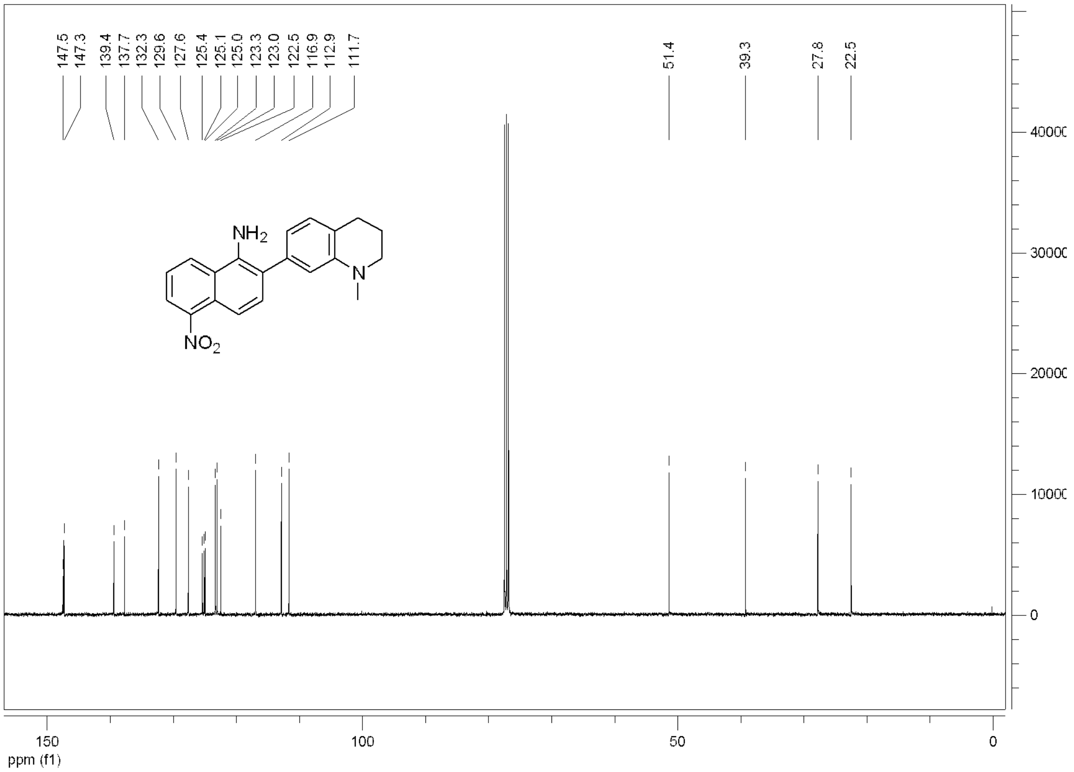

Figure S58: The ^{13}C-NMR spectrum of compound 18

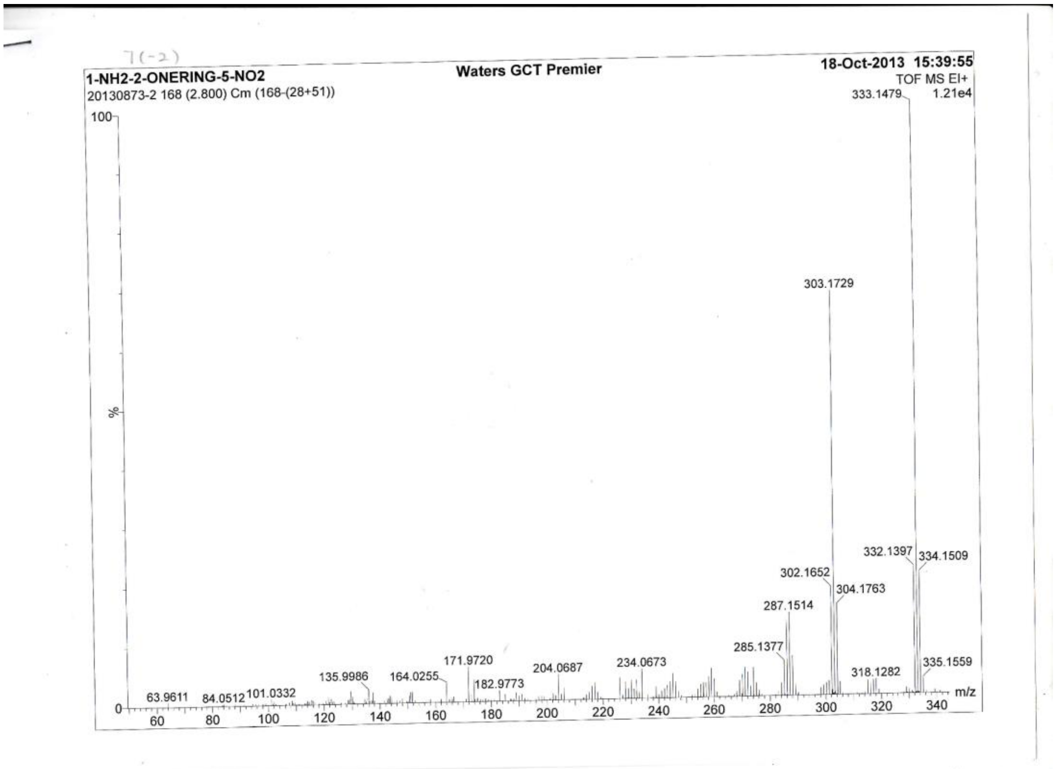

Figure S59: The HRMS spectrum of compound 18

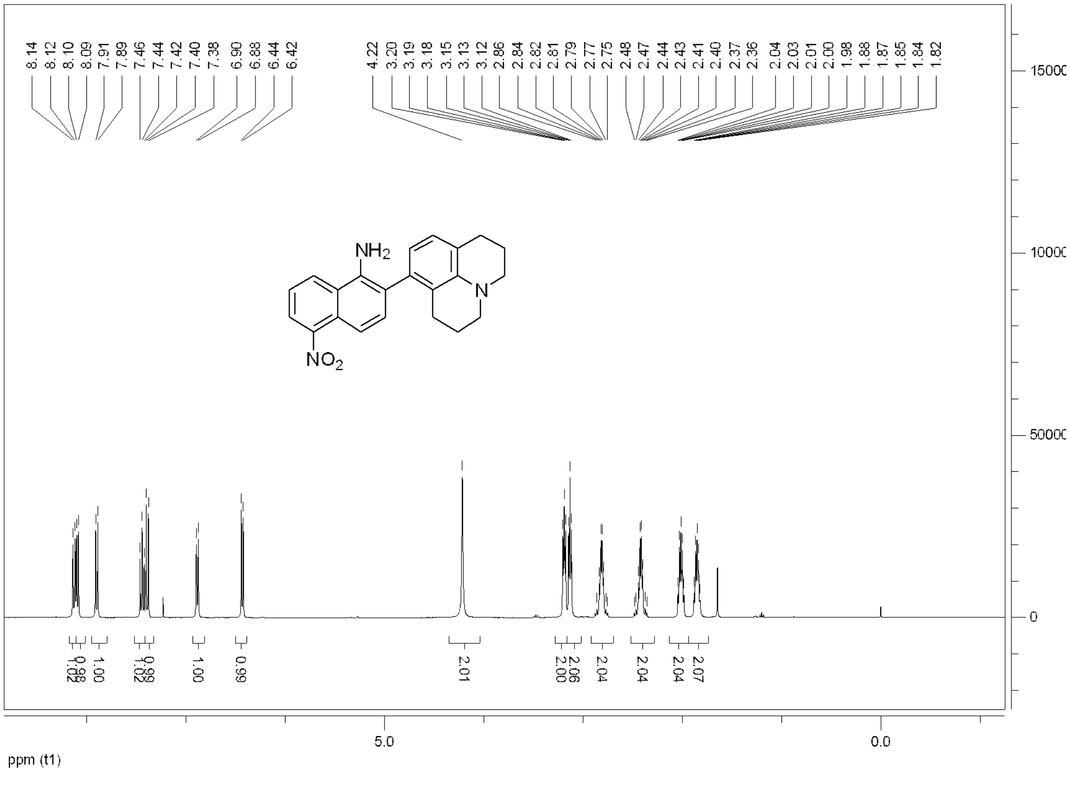

Figure S60: The ^{1}H-NMR spectrum of compound 19

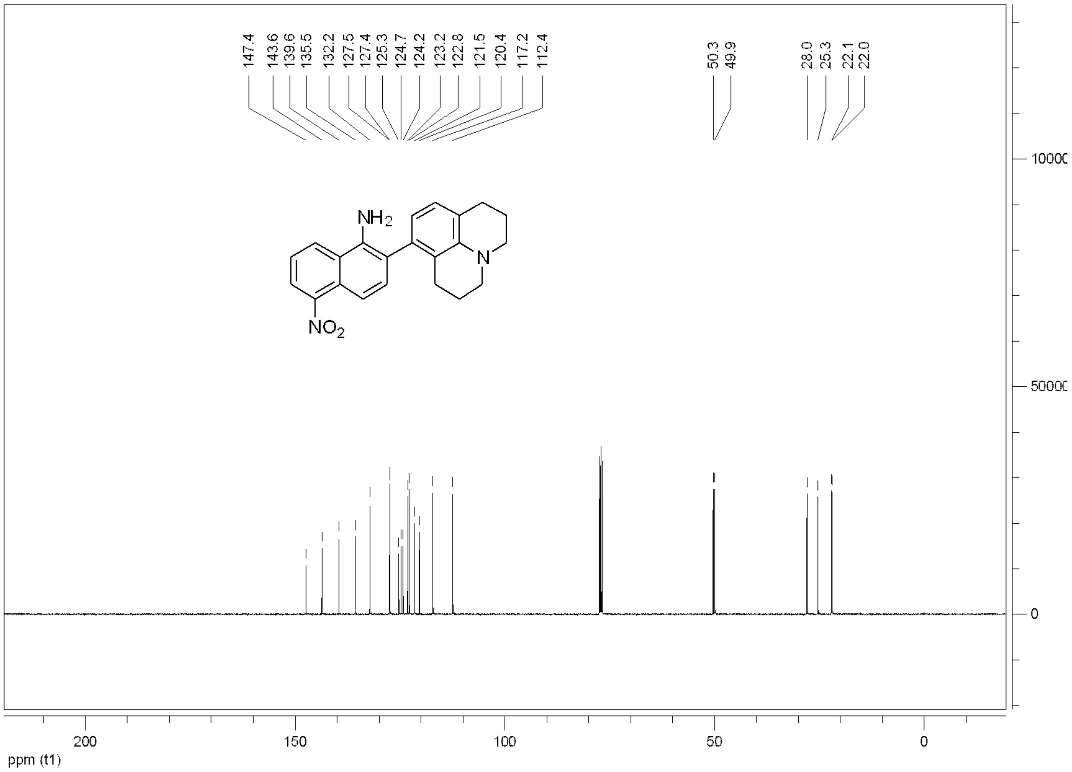

Figure S61: The ^{13}C-NMR spectrum of compound 19

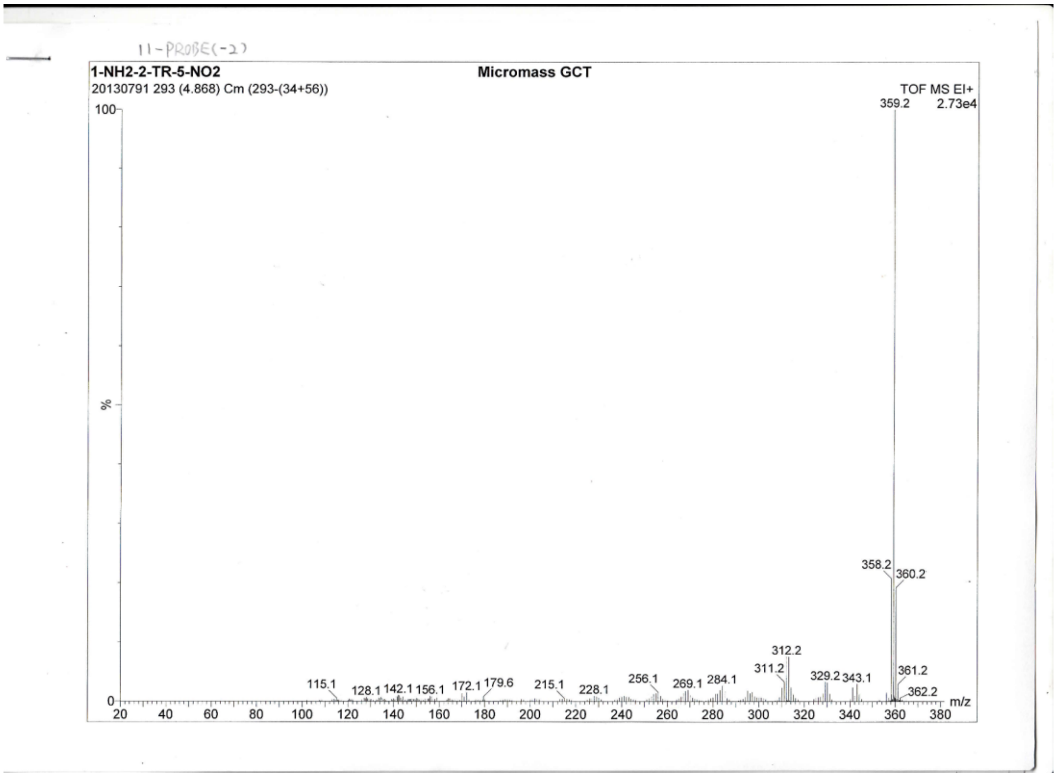

Figure S62: The MS spectrum of compound 19

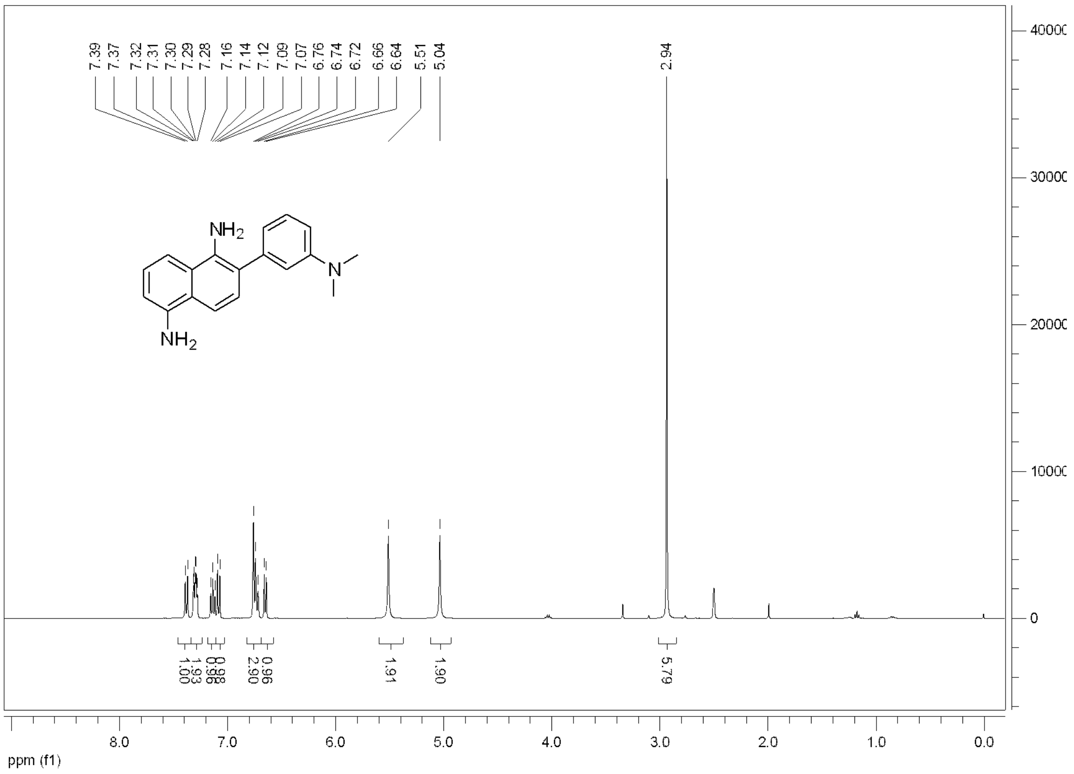

Figure S63: The ^{1}H-NMR spectrum of compound 20

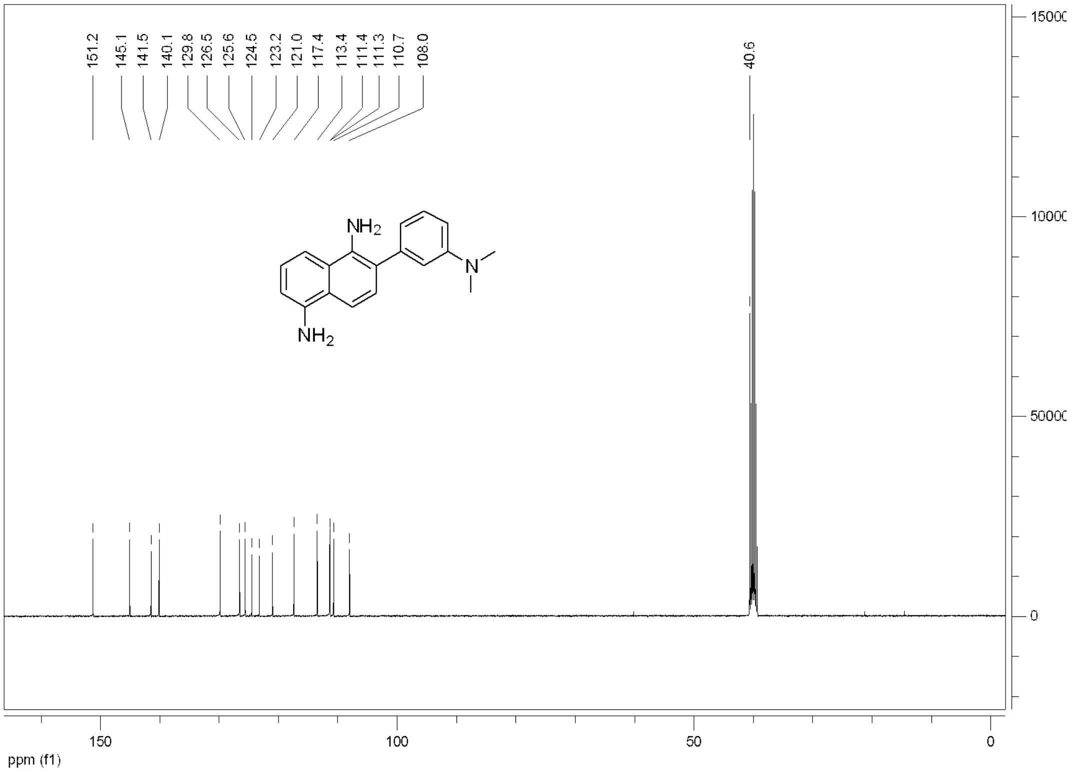

Figure S64: The ^{13}C-NMR spectrum of compound 20

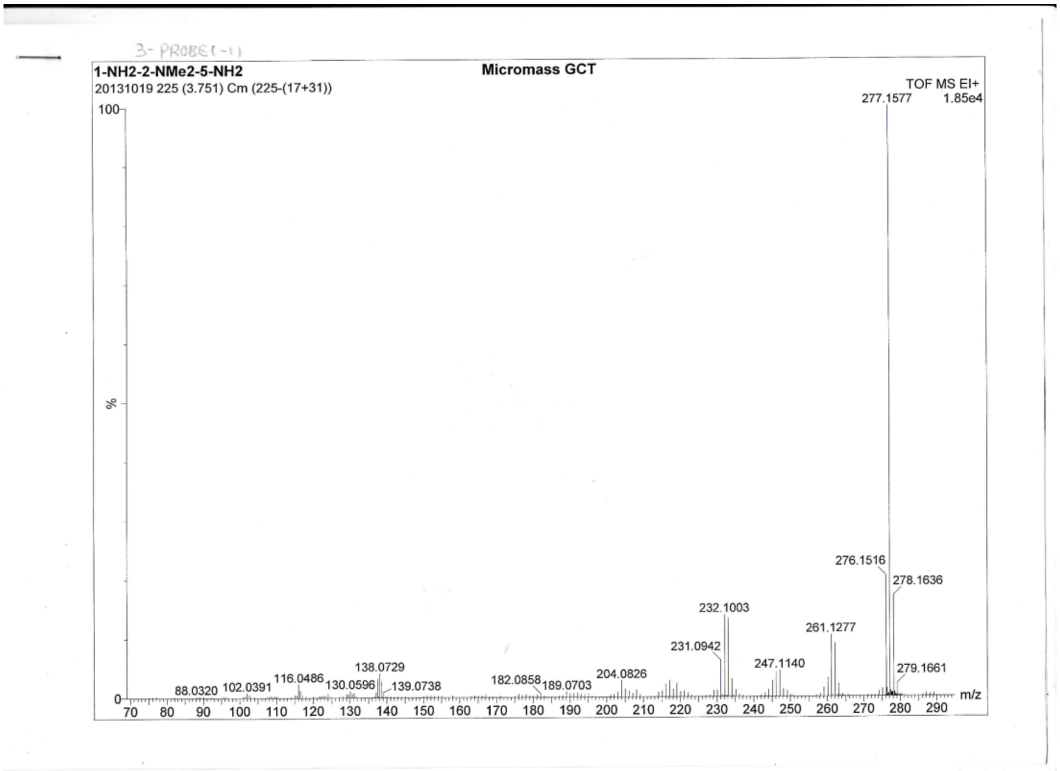

Figure S65: The HRMS spectrum of compound 20

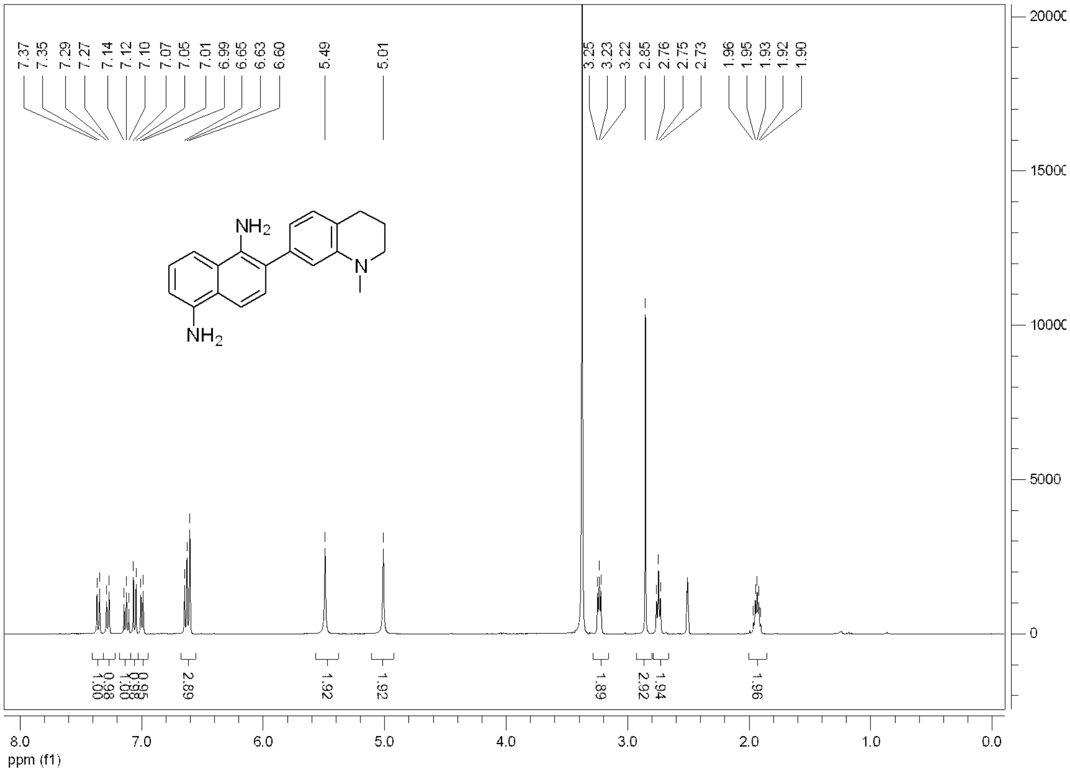

Figure S66: The ^{1}H-NMR spectrum of compound 21

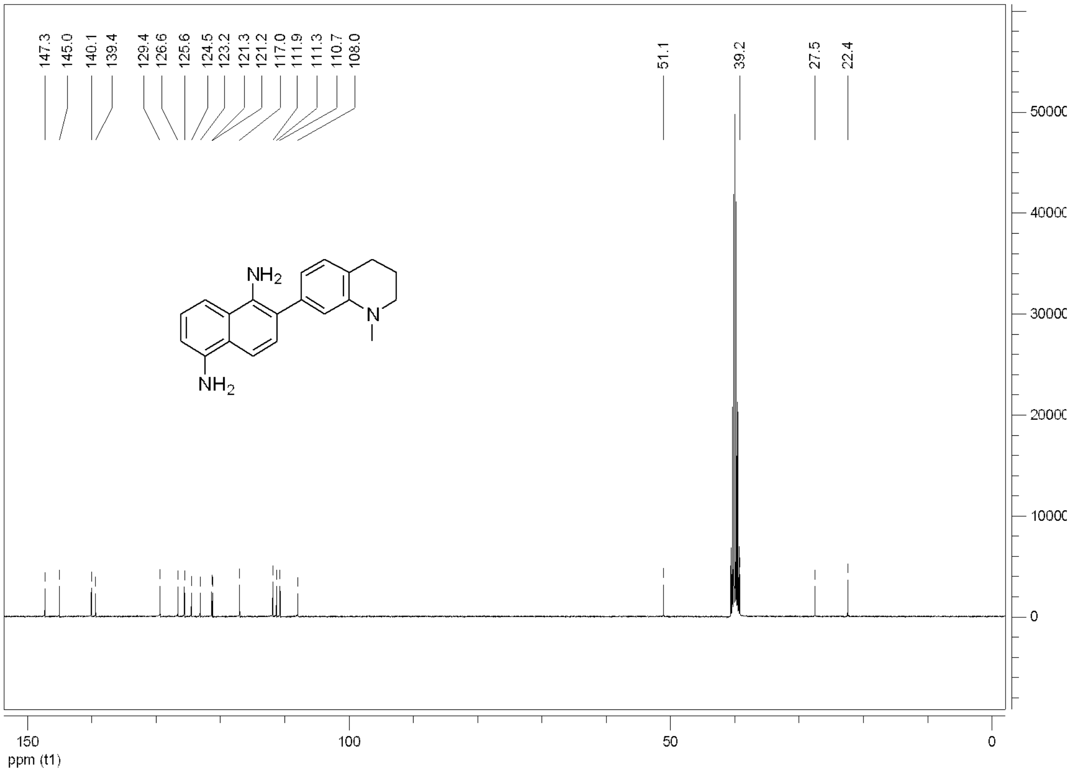

Figure S67: The ^{13}C-NMR spectrum of compound 21

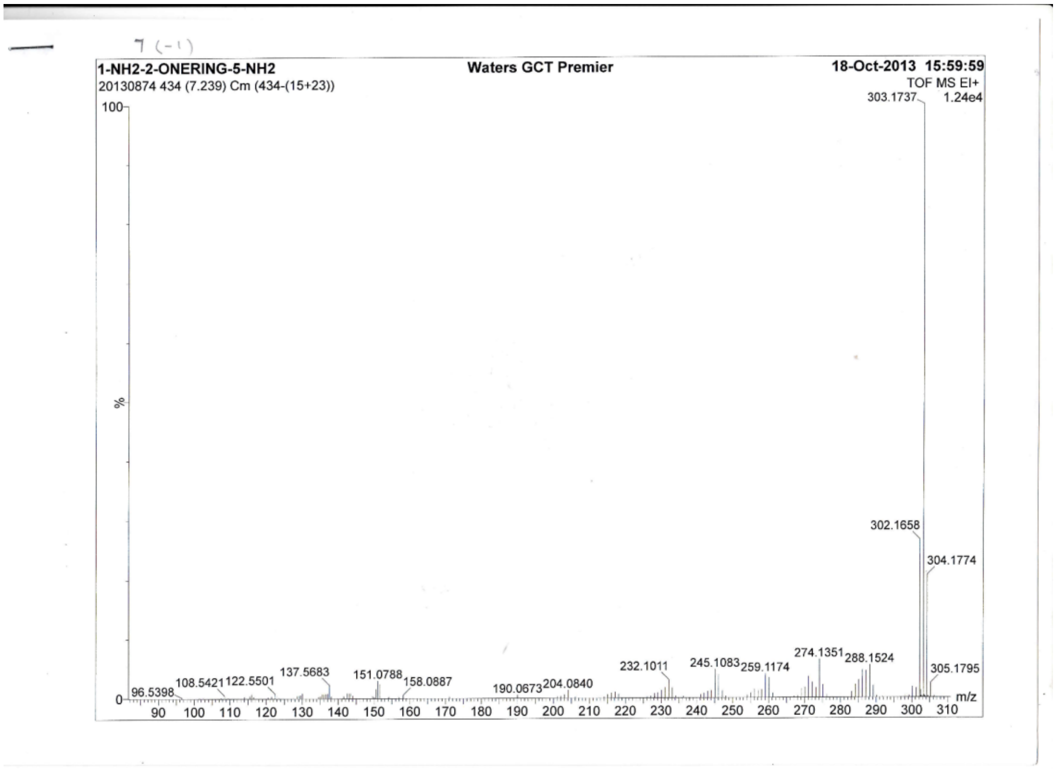

Figure S68: The HRMS spectrum of compound 21

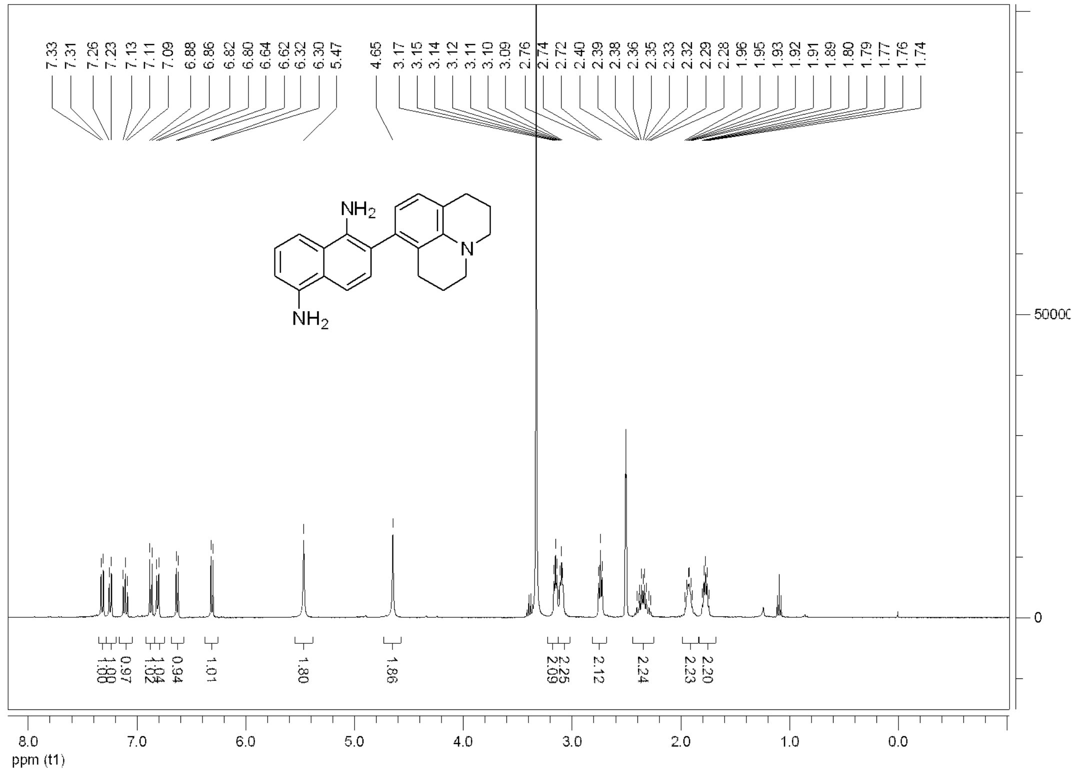

Figure S69: The ^{1}H-NMR spectrum of compound 22

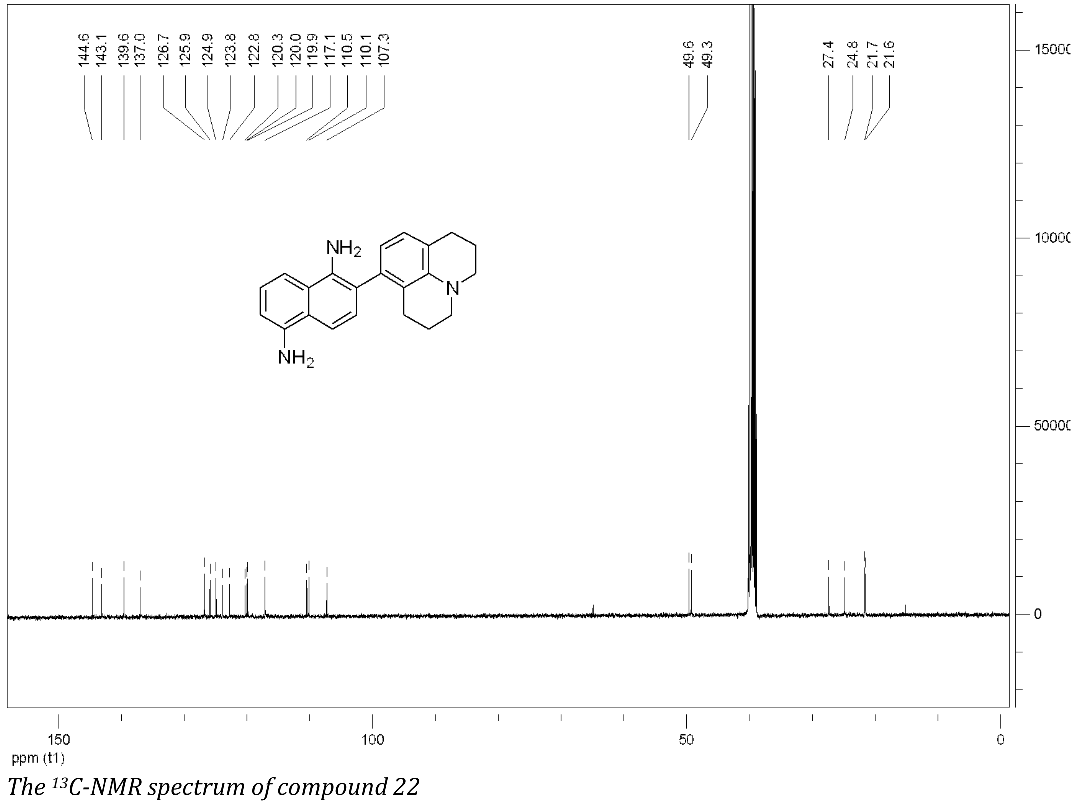

Figure S70: The ^{13}C-NMR spectrum of compound 22

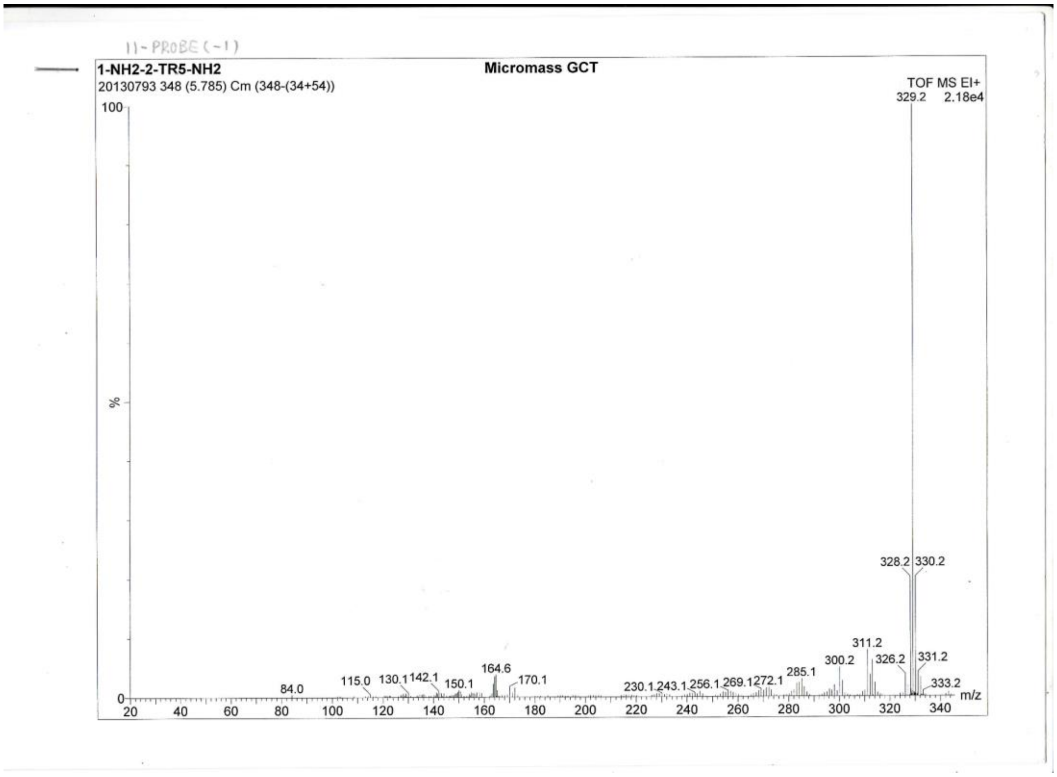

Figure S71: The MS spectrum of compound 22

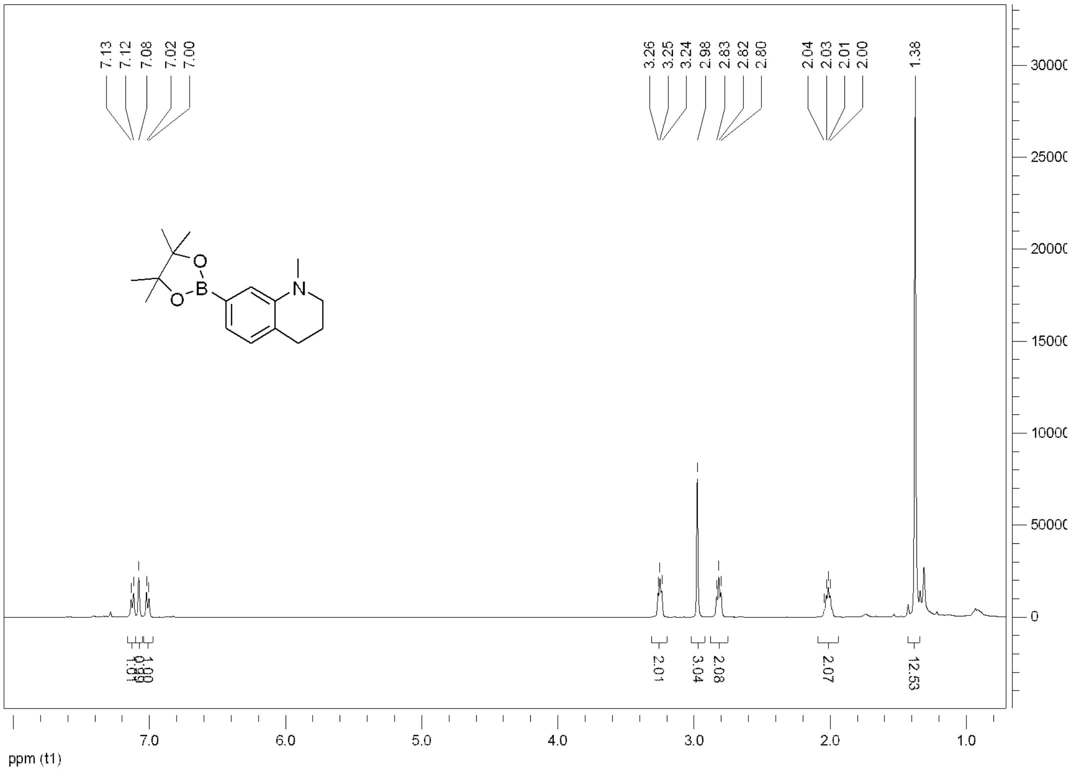

Figure S72: The ^{1}H-NMR spectrum of compound 23

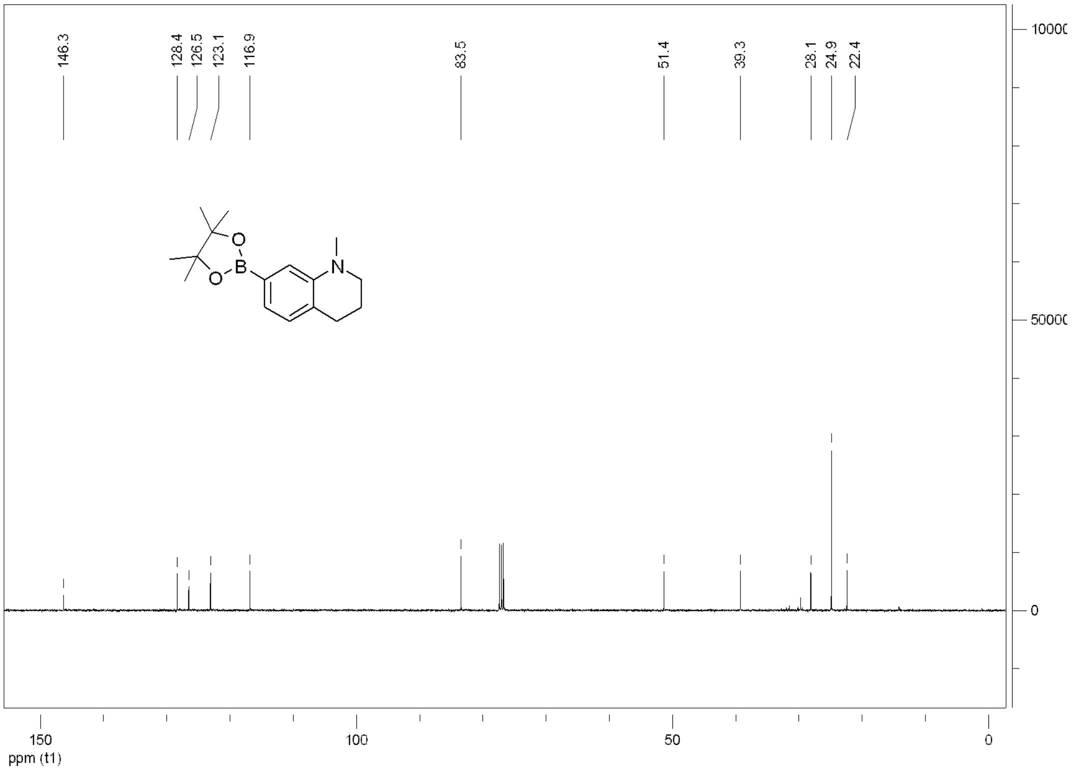

Figure S73: The ^{13}C-NMR spectrum of compound 23

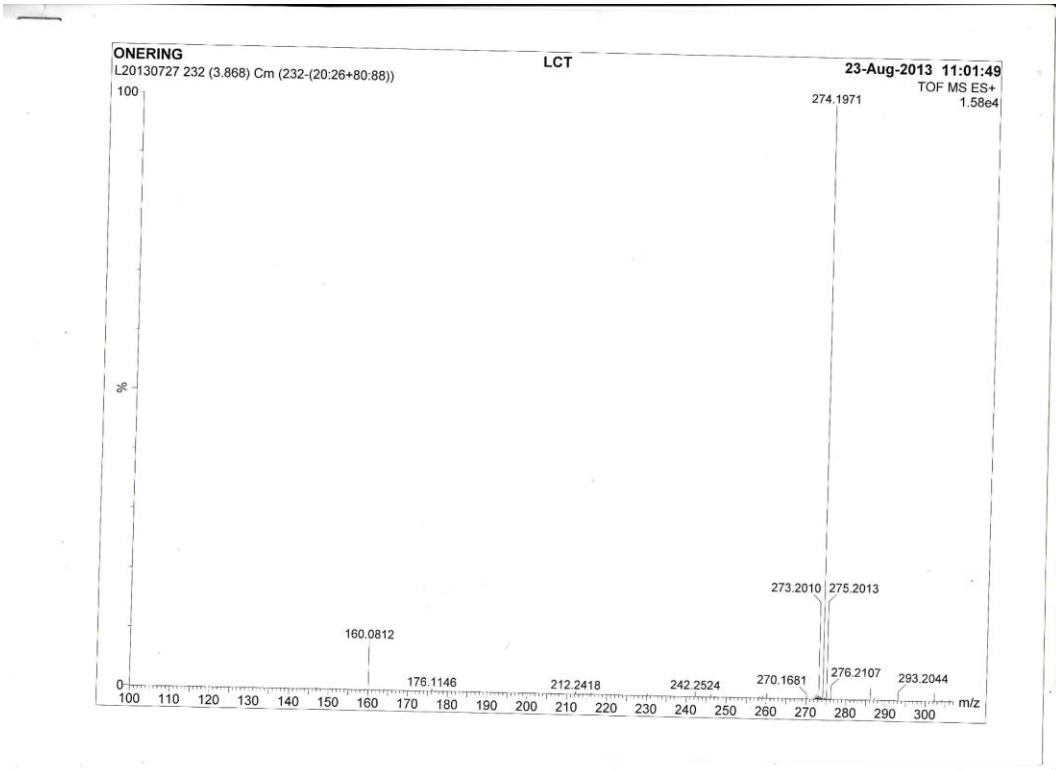

Figure S74: The HRMS spectrum of compound 23

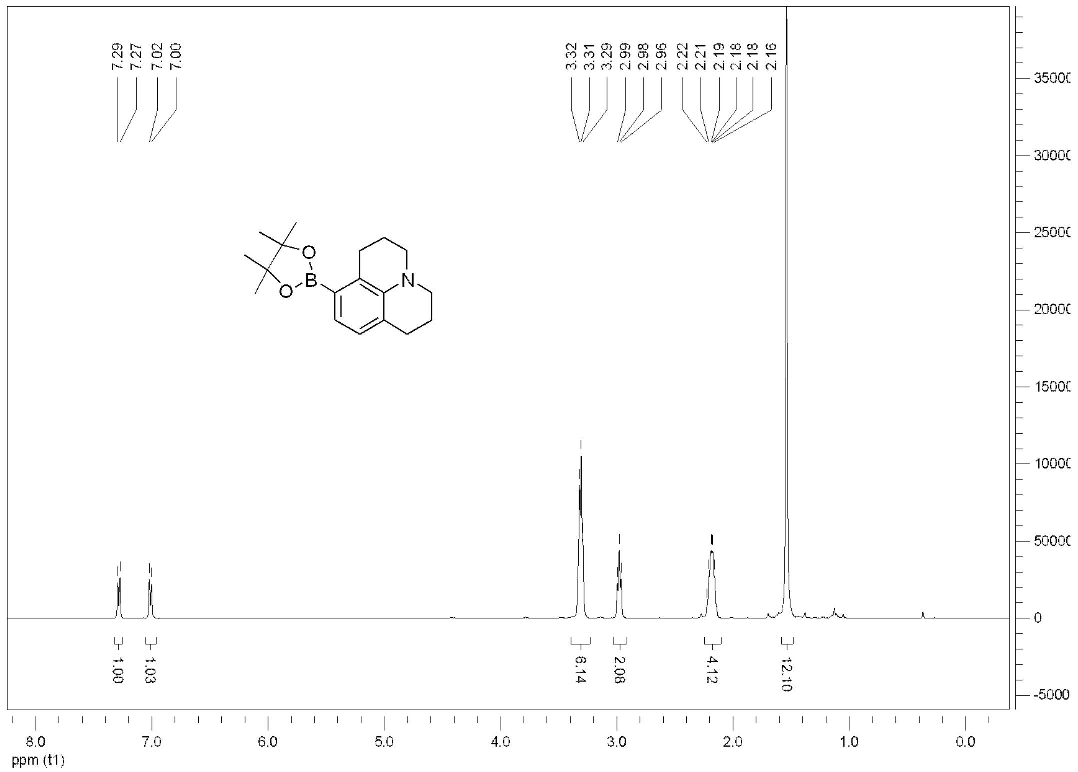

Figure S75: The ^{1}H-NMR spectrum of compound 24

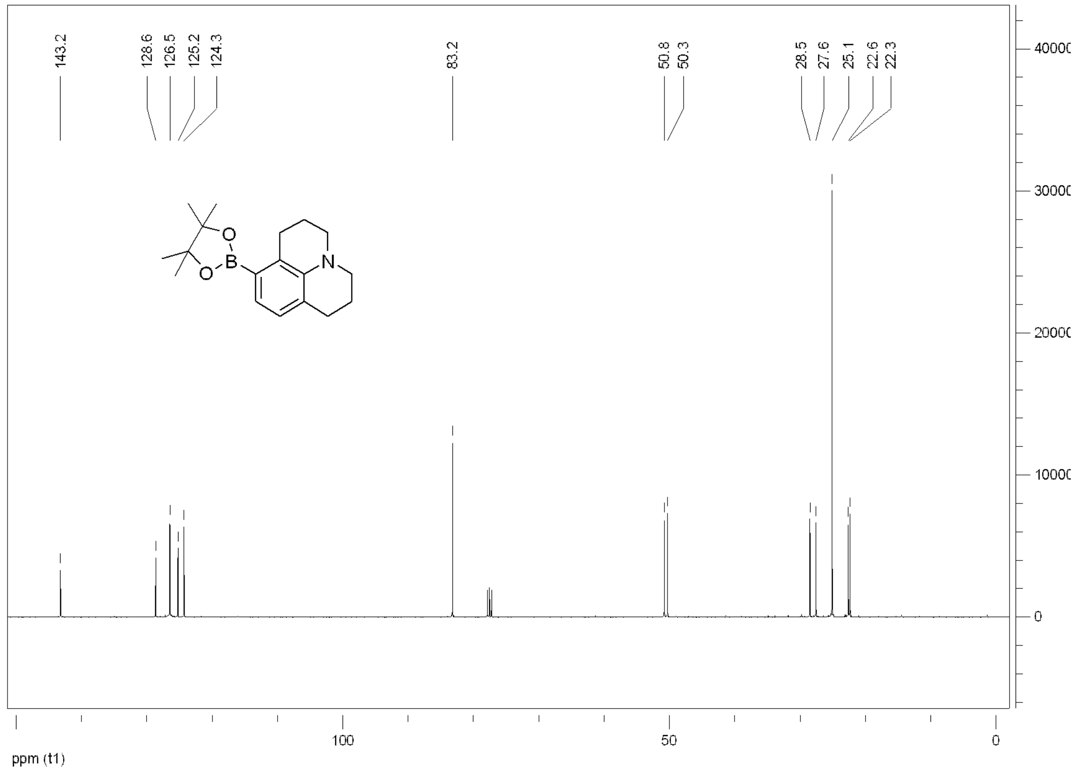

Figure S76: The ^{13}C-NMR spectrum of compound 24

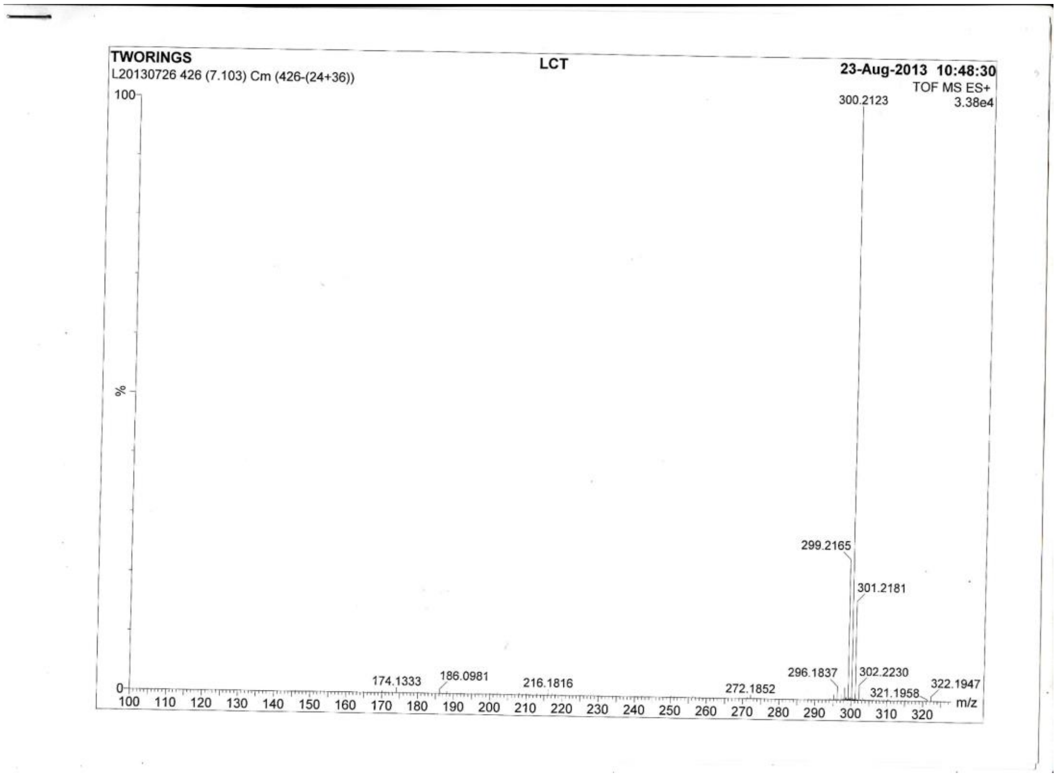

Figure S77: The HRMS spectrum of compound 24

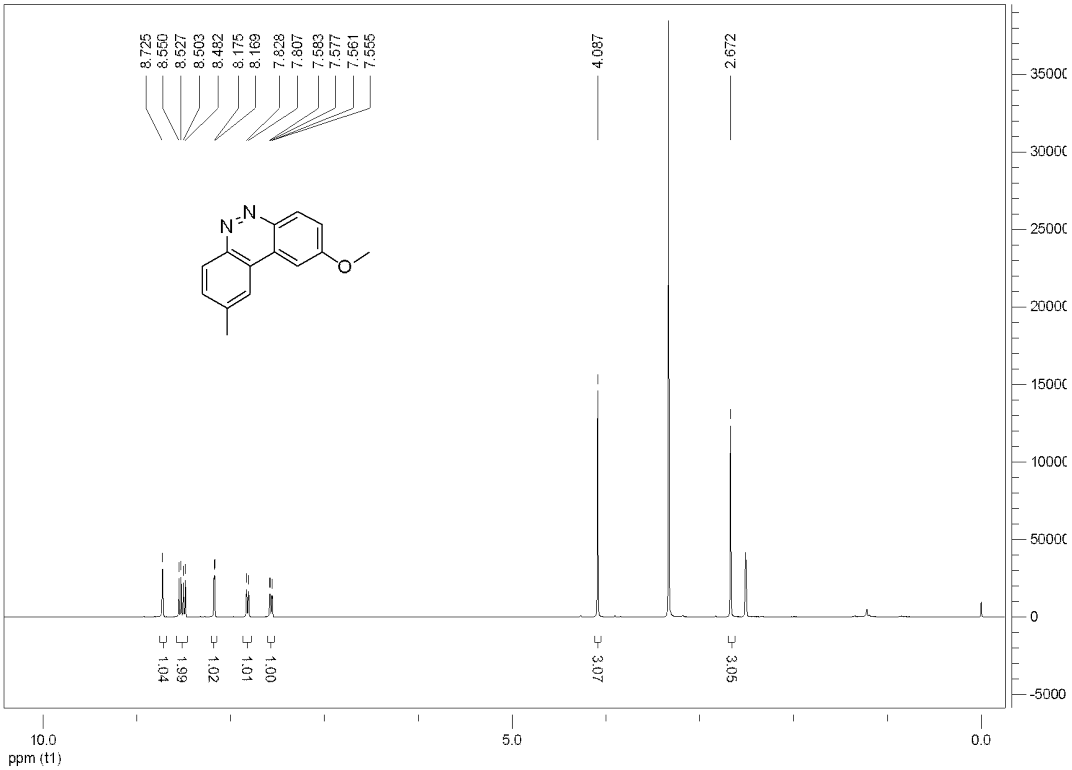

Figure S78: The ^{1}H-NMR spectrum of the cinnoline product from 1

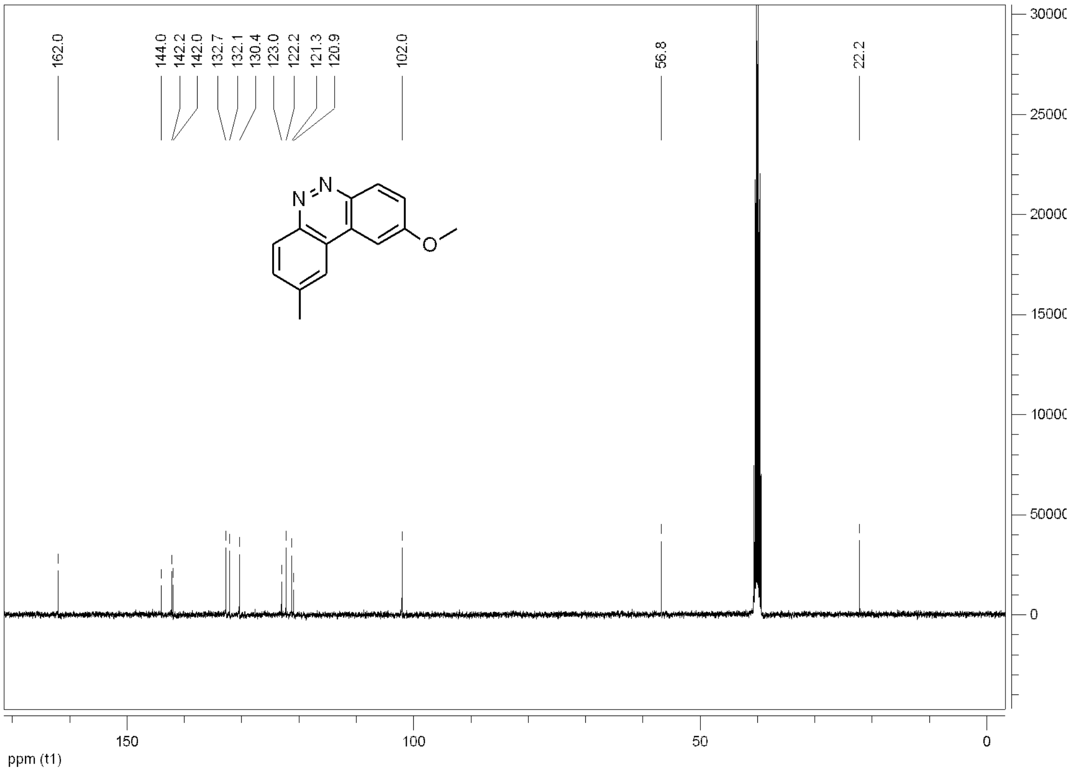

Figure S79: The ^{13}C-NMR spectrum of the cinnoline product from 1

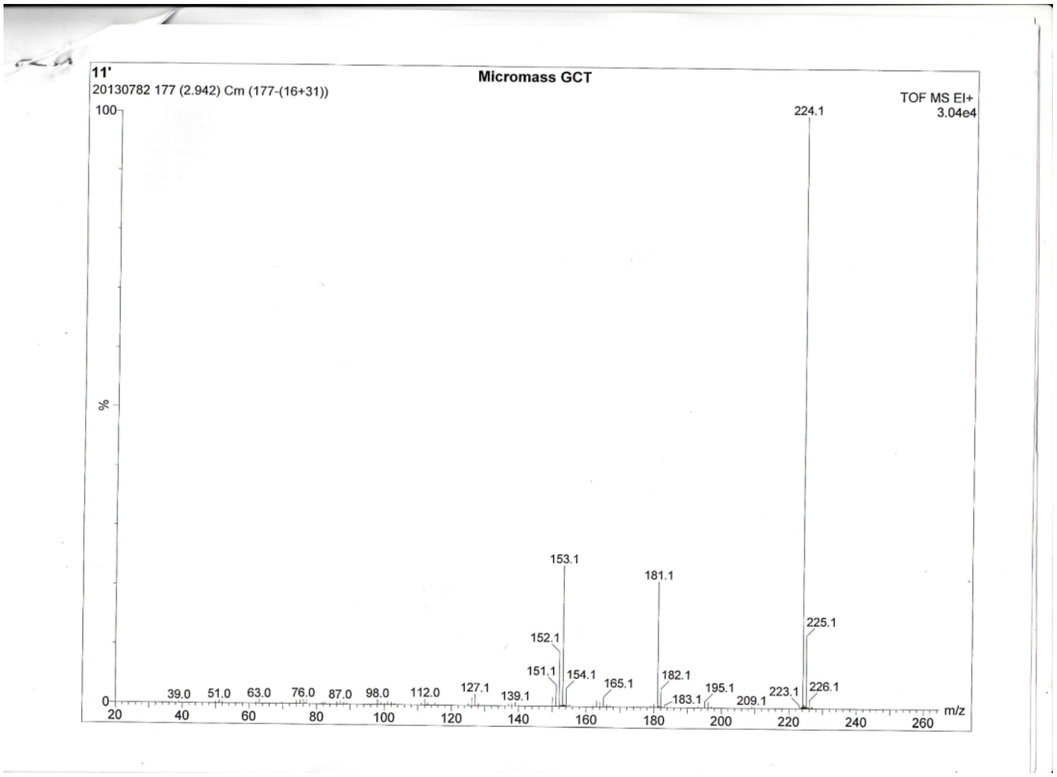

Figure S80: The MS spectrum of the cinnoline product from 1

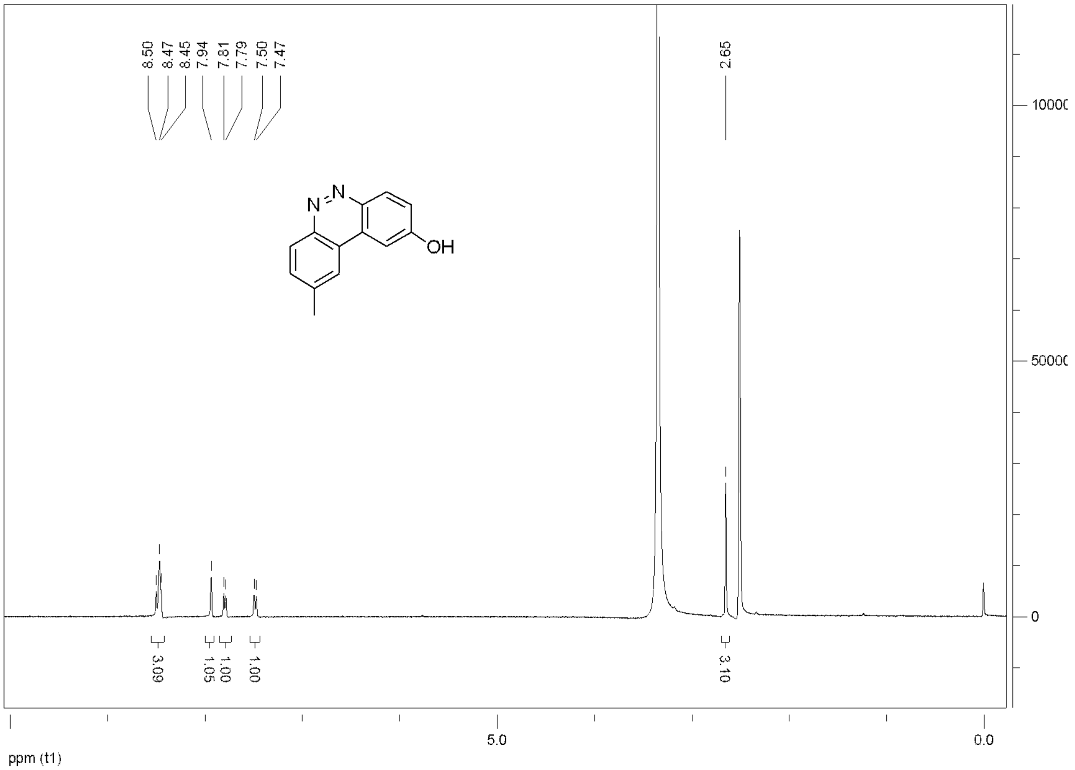

Figure S81: The ^{1}H-NMR spectrum of the cinnoline product from 2

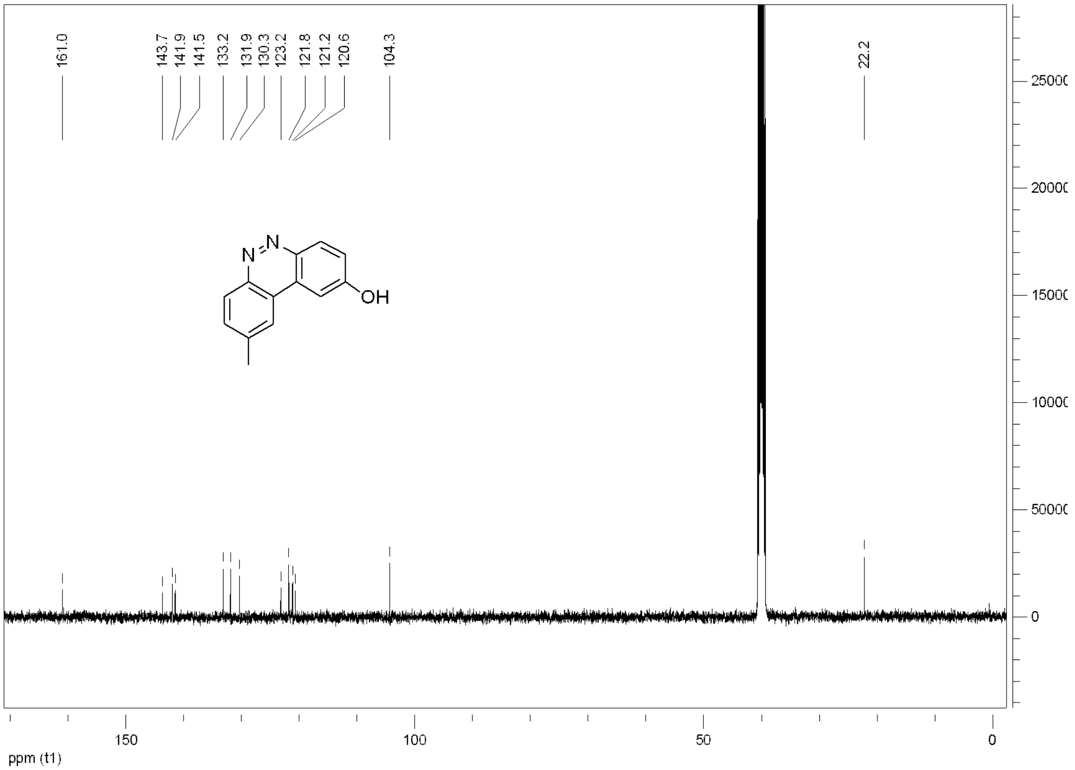

Figure S82: The ^{13}C-NMR spectrum of the cinnoline product from 2

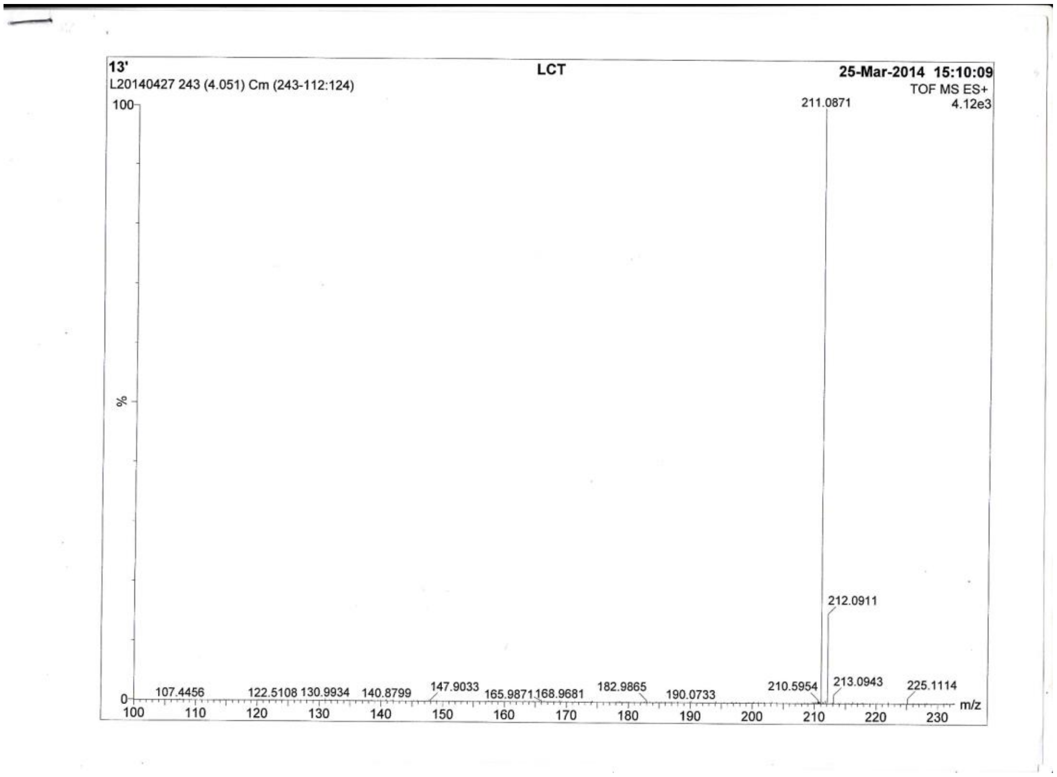

Figure S83: The HRMS spectrum of the cinnoline product from 2

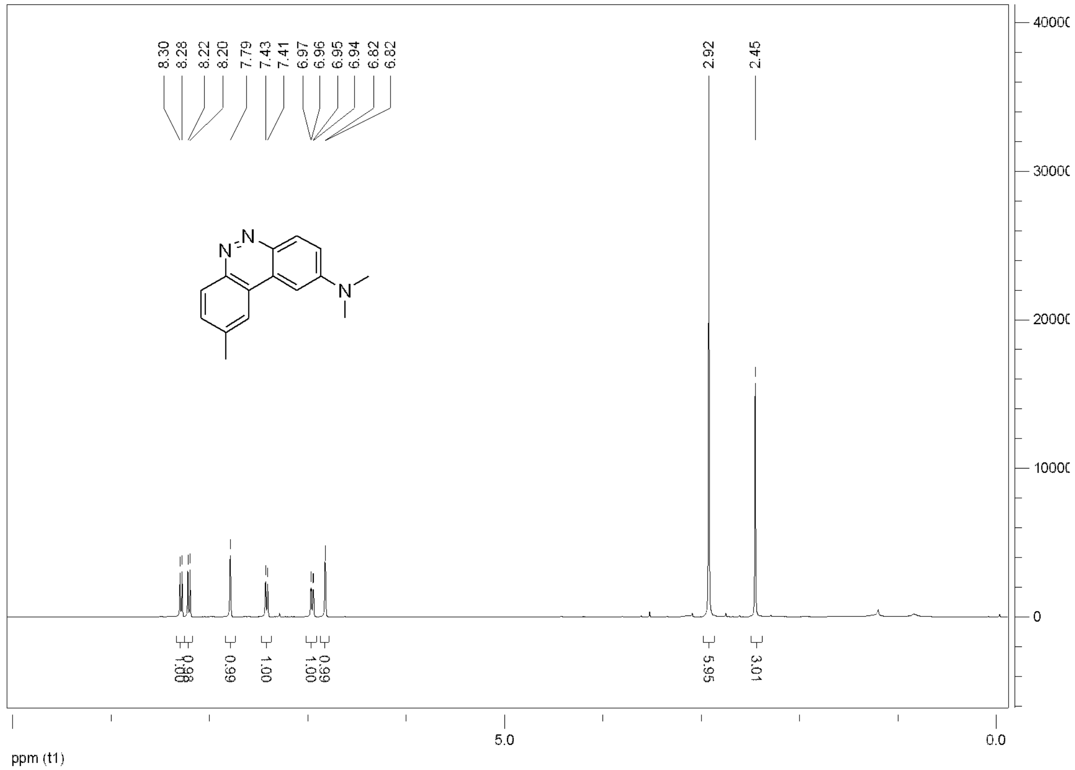

Figure S84: The ^{1}H-NMR spectrum of the cinnoline product from 3

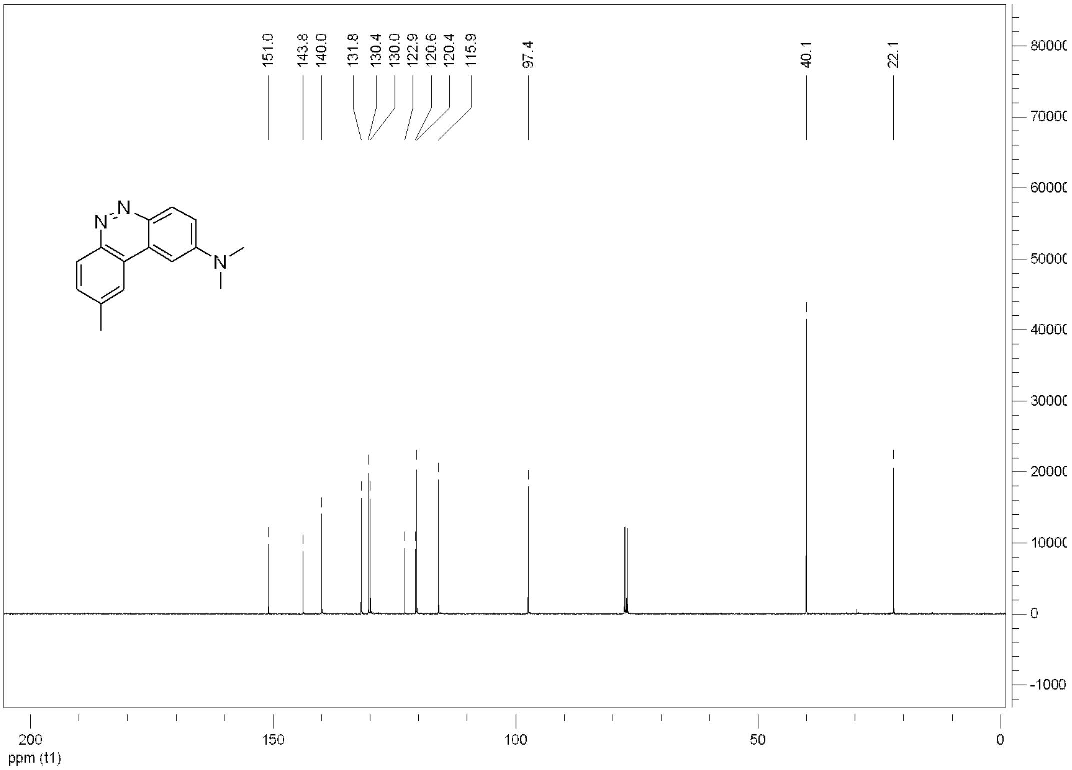

Figure S85: The ^{13}C-NMR spectrum of the cinnoline product from 3

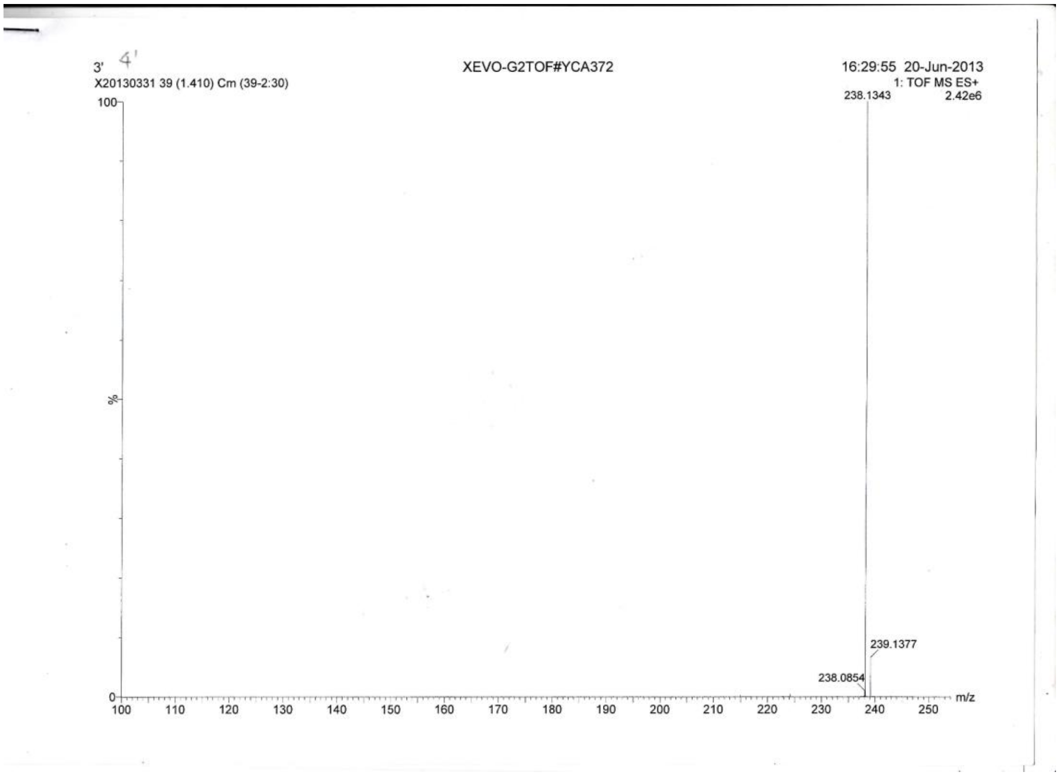

Figure S86: The HRMS spectrum of the cinnoline product from 3

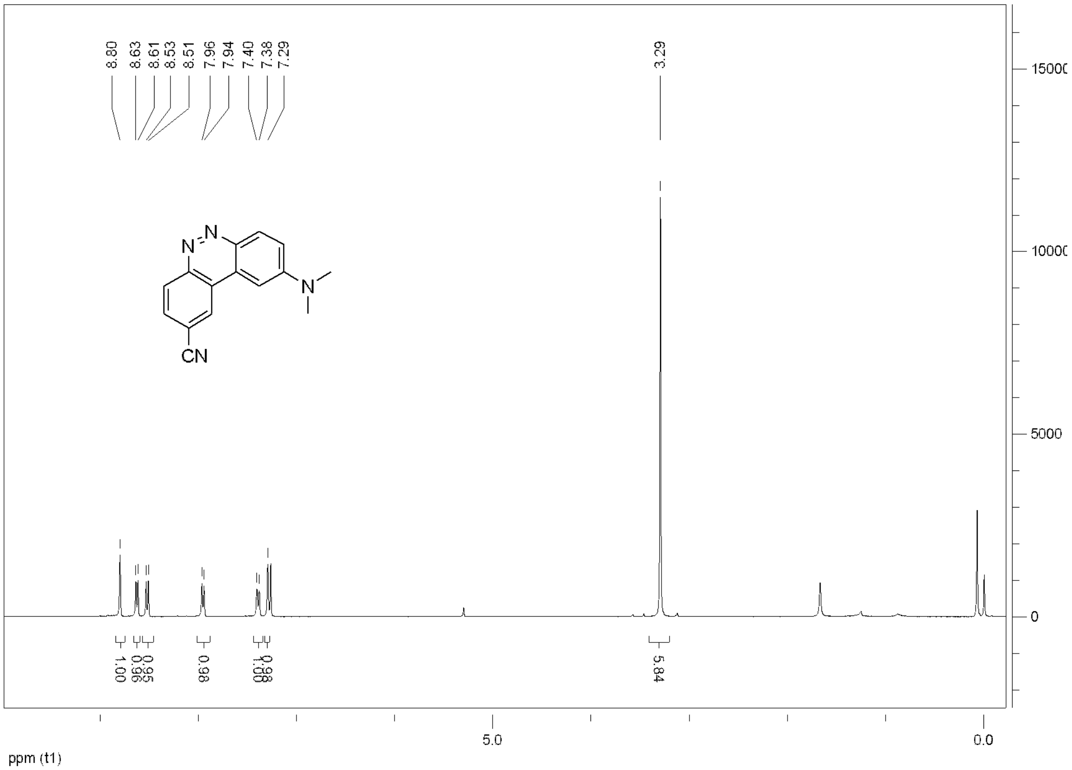

Figure S87: The ^{1}H-NMR spectrum of the cinnoline product from 4

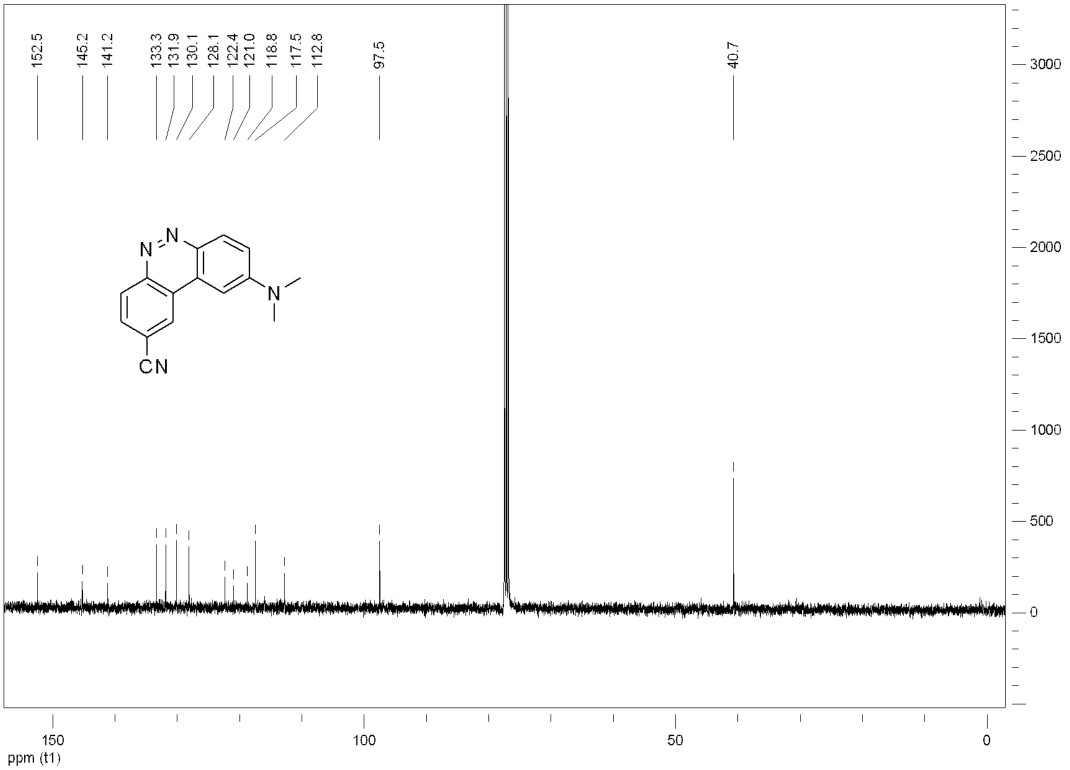

Figure S88: The ^{13}C-NMR spectrum of the cinnoline product from 4

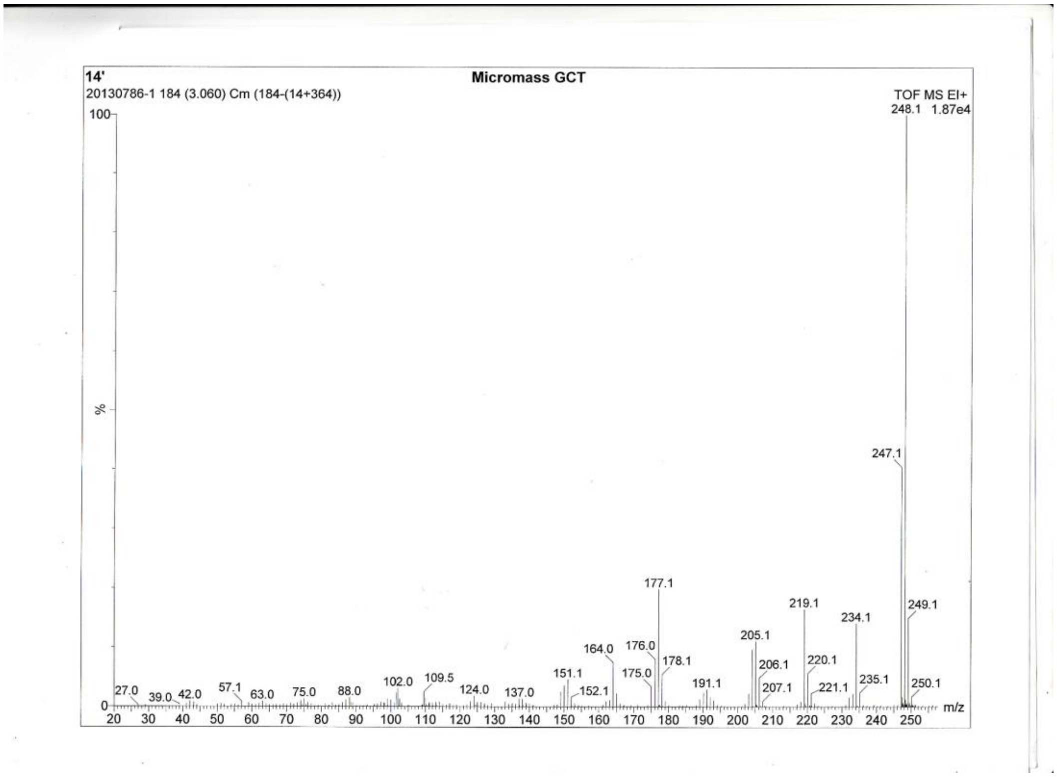

Figure S89: The MS spectrum of the cinnoline product from 4

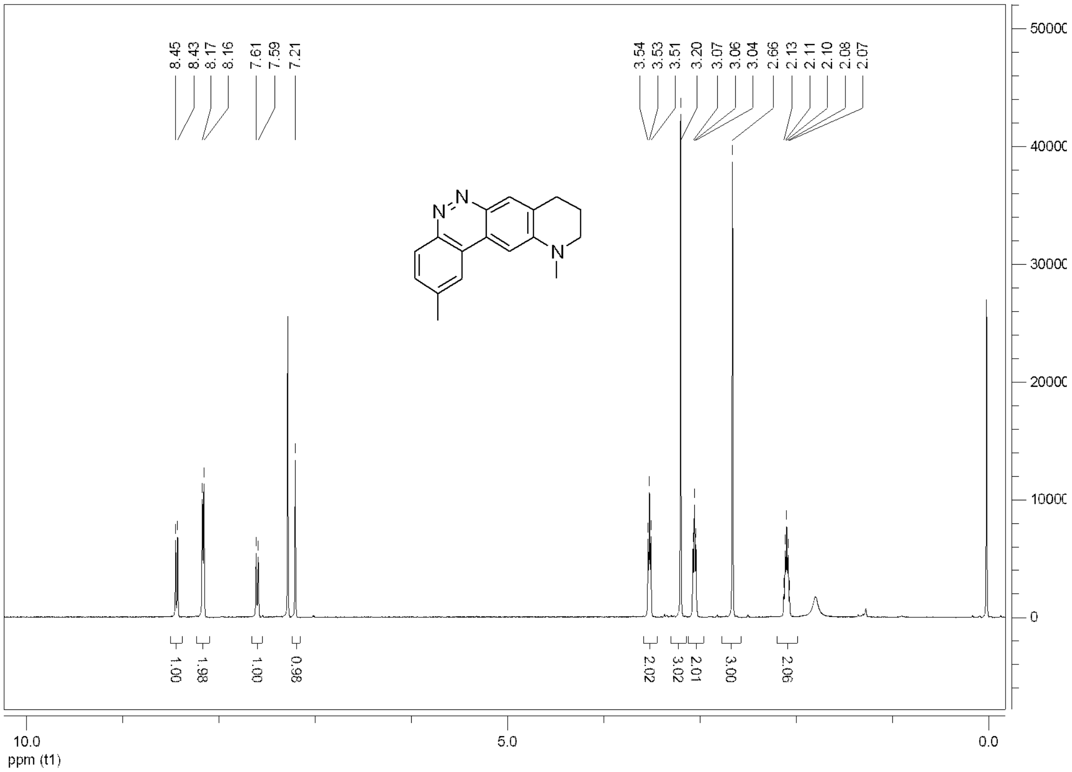

Figure S90: The ^{1}H-NMR spectrum of the cinnoline product from 5

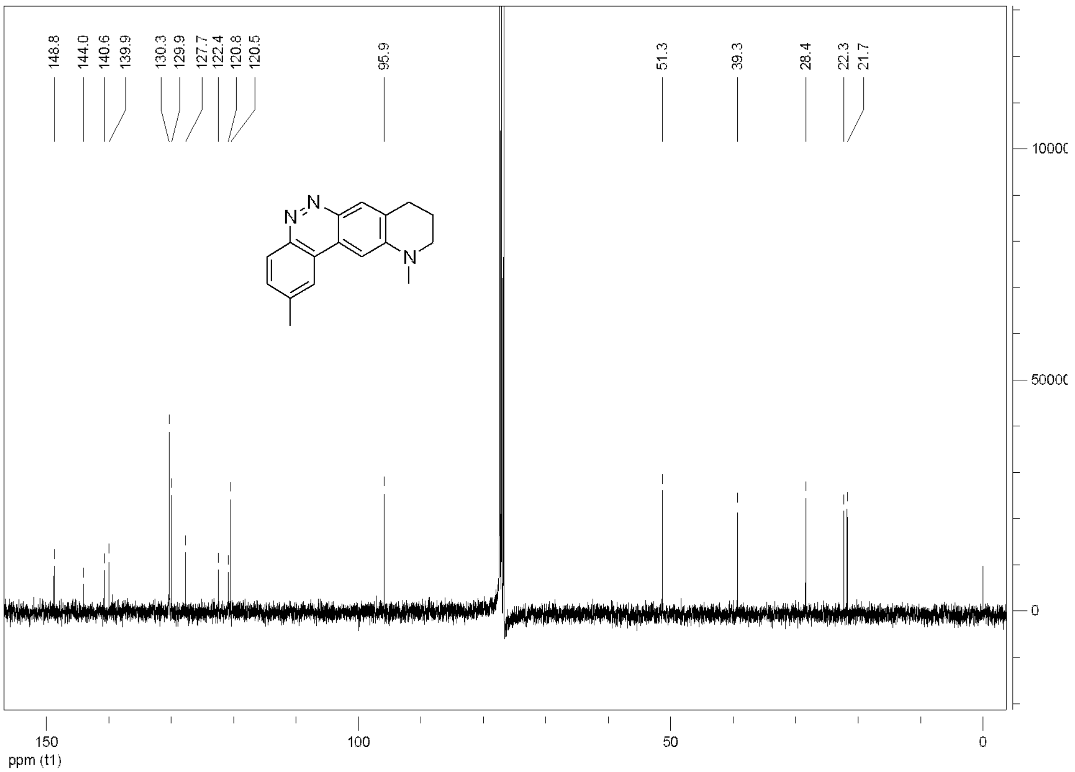

Figure S91: The ^{13}C-NMR spectrum of the cinnoline product from 5

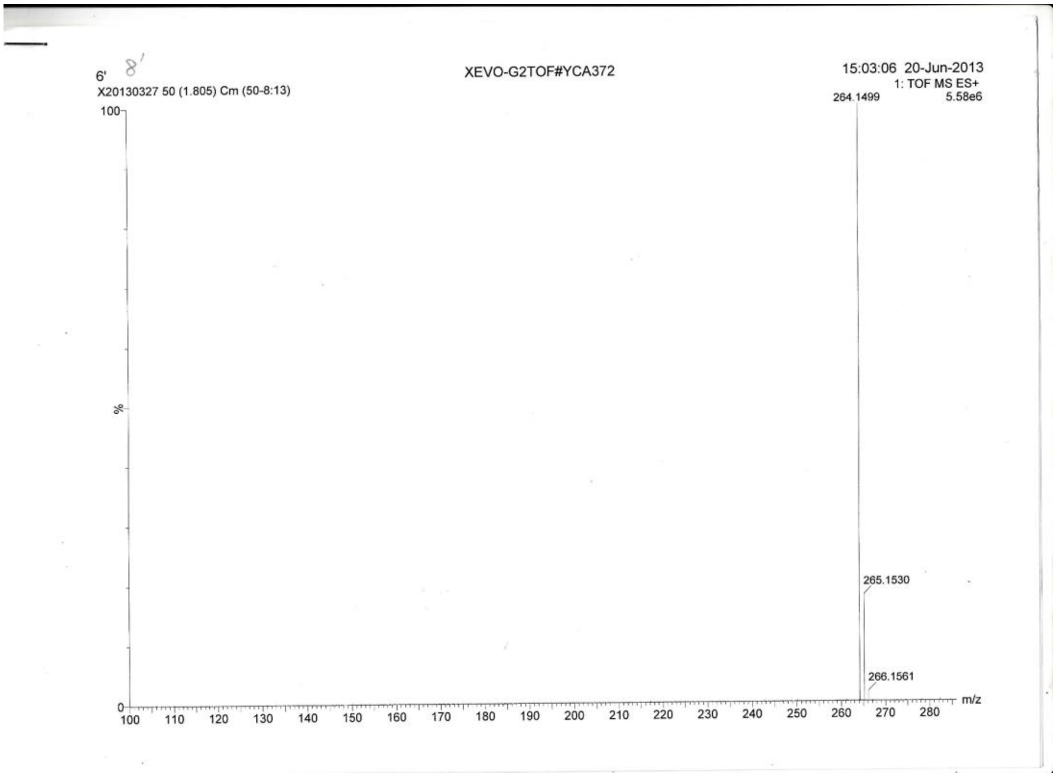

Figure S92: The HRMS spectrum of the cinnoline product from 5

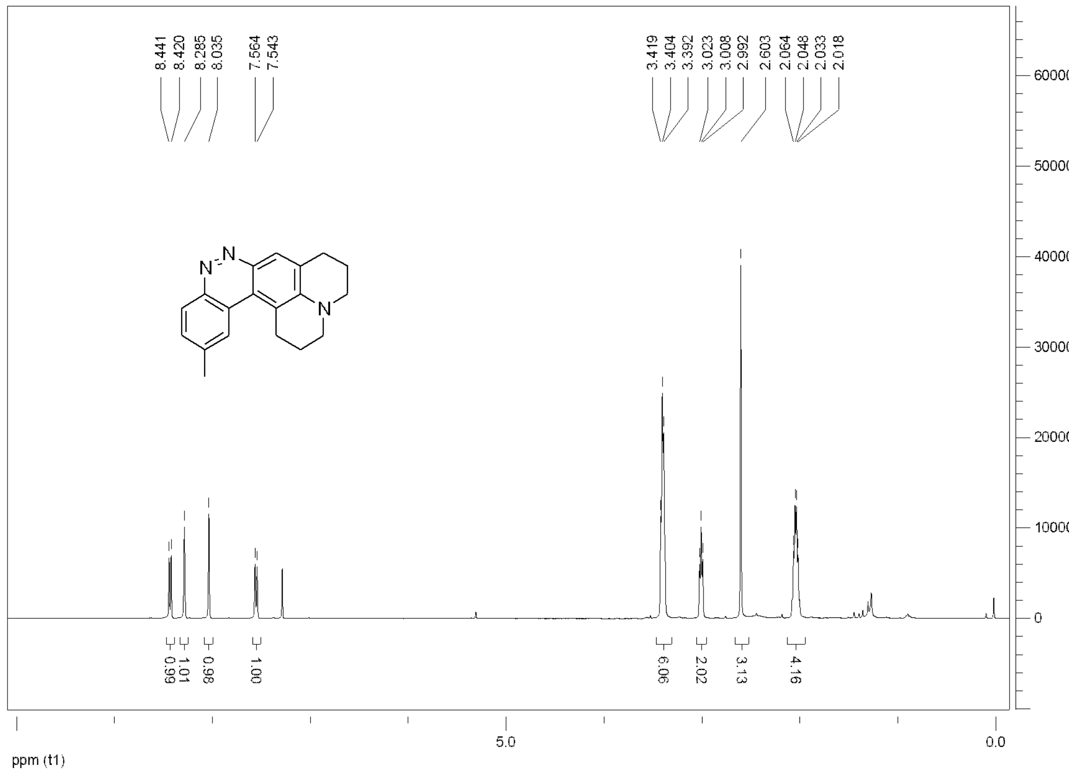

Figure S93: The ¹H-NMR spectrum of the cinnoline product from 6

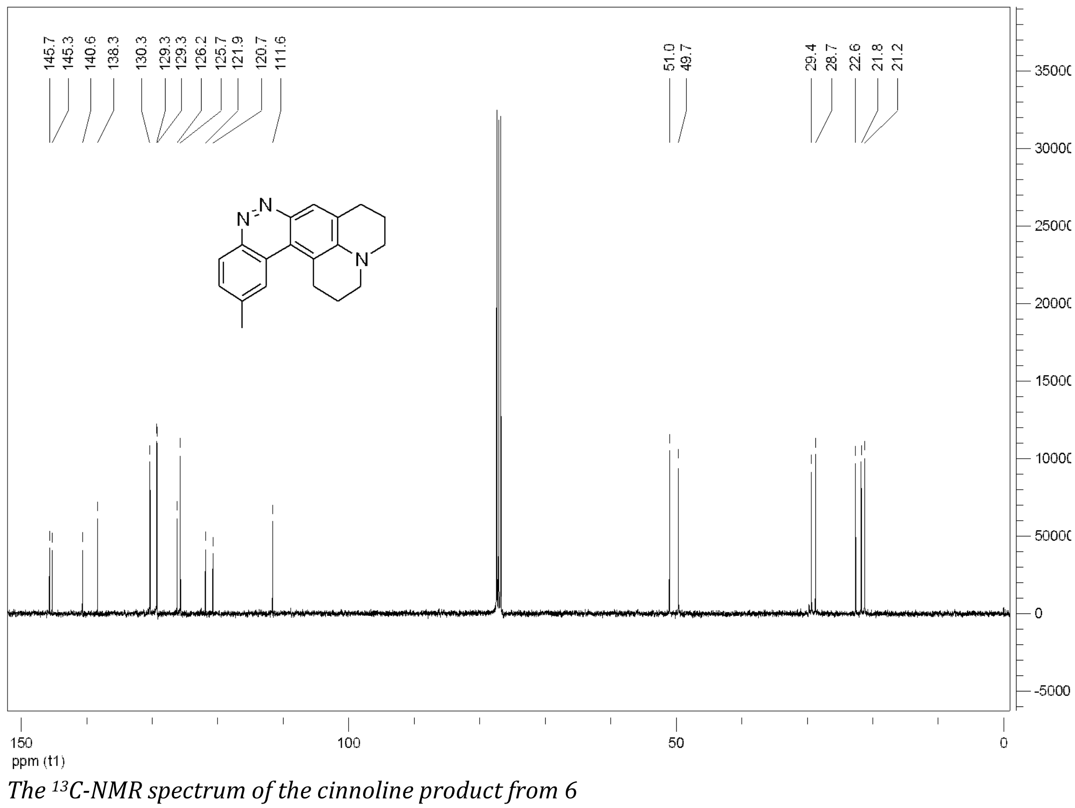

Figure S94: The ¹³C-NMR spectrum of the cinnoline product from 6

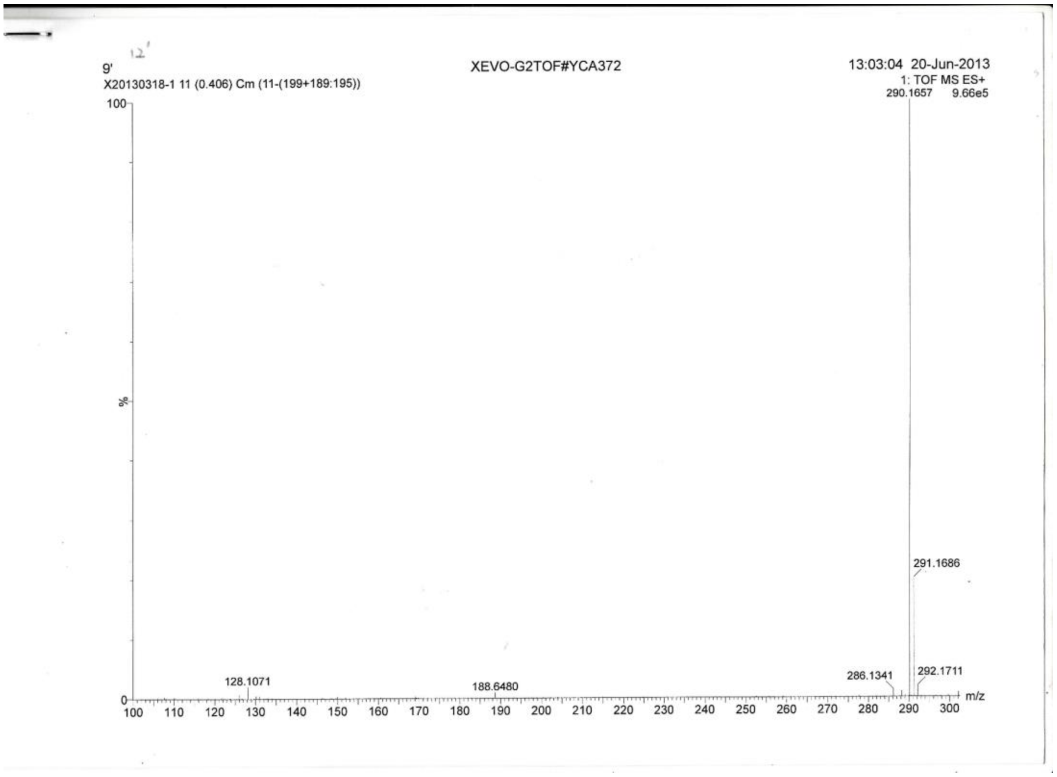

Figure S95: The HRMS spectrum of the cinnoline product from 6

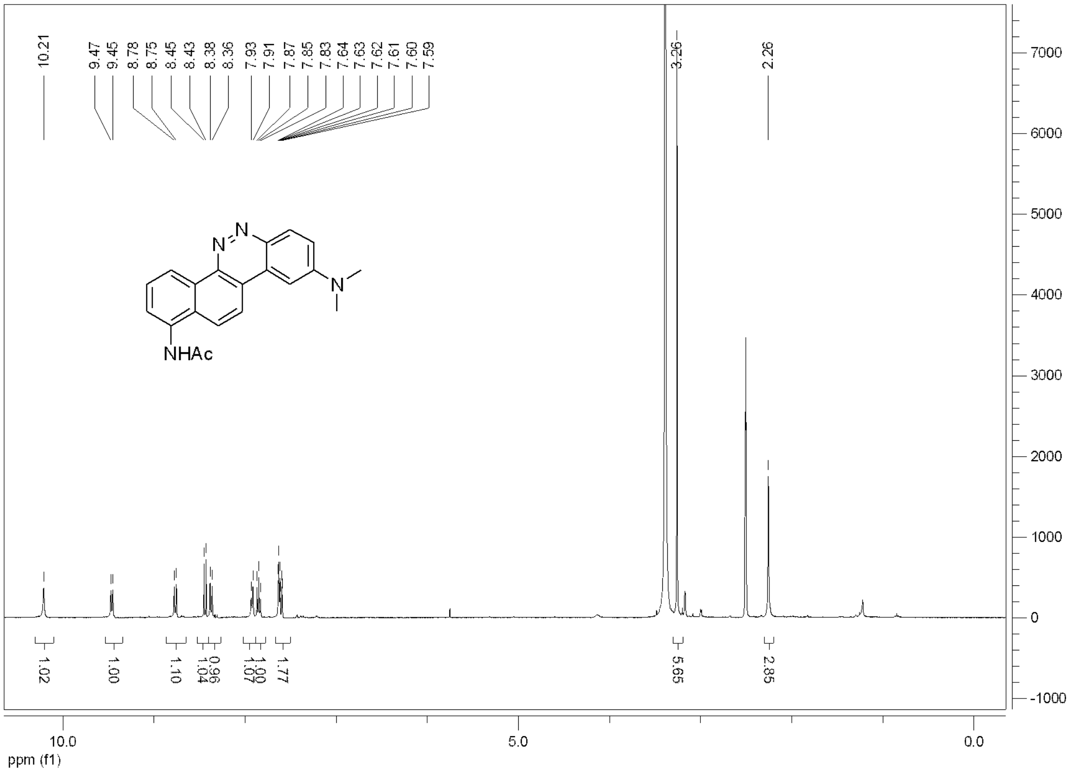

Figure S96: The ^{1}H-NMR spectrum of the cinnoline product from 7

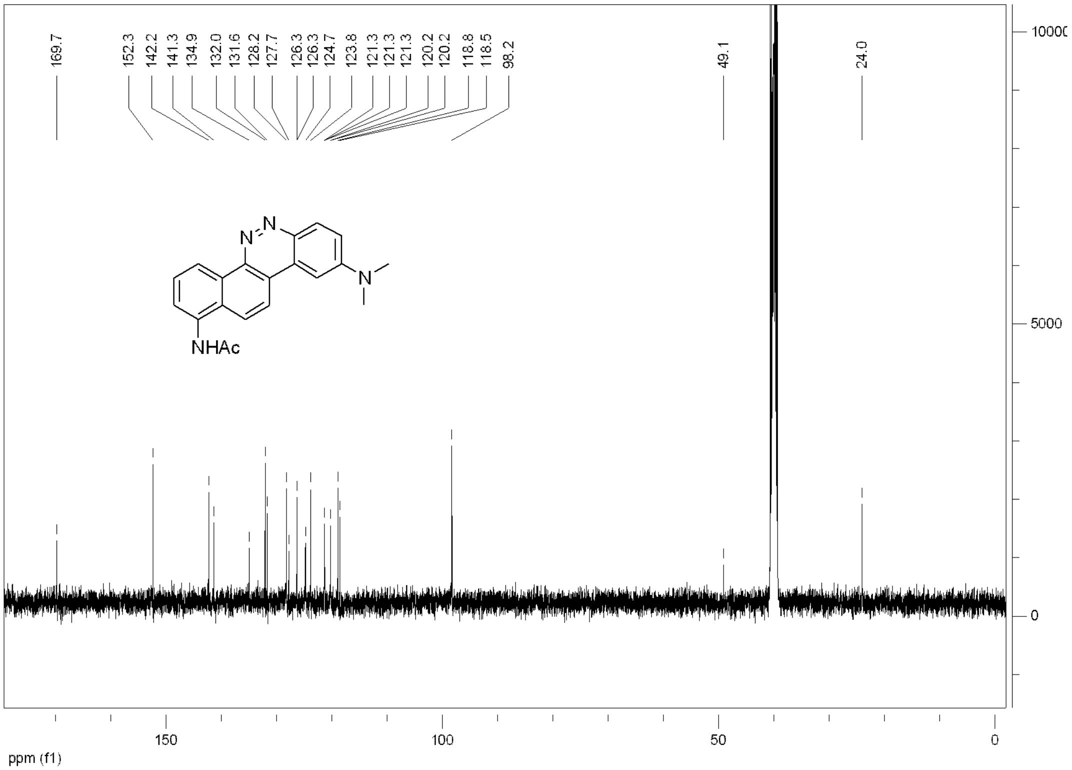

Figure S97: The ^{13}C-NMR spectrum of the cinnoline product from 7

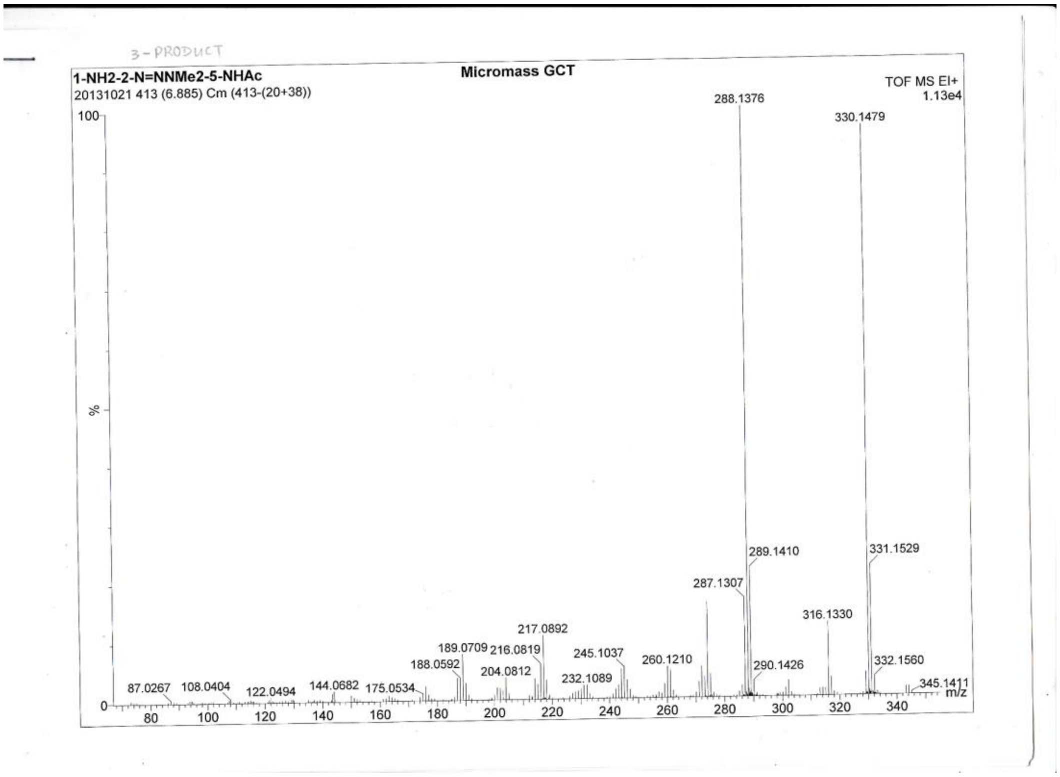

Figure S98: The HRMS spectrum of the cinnoline product from 7

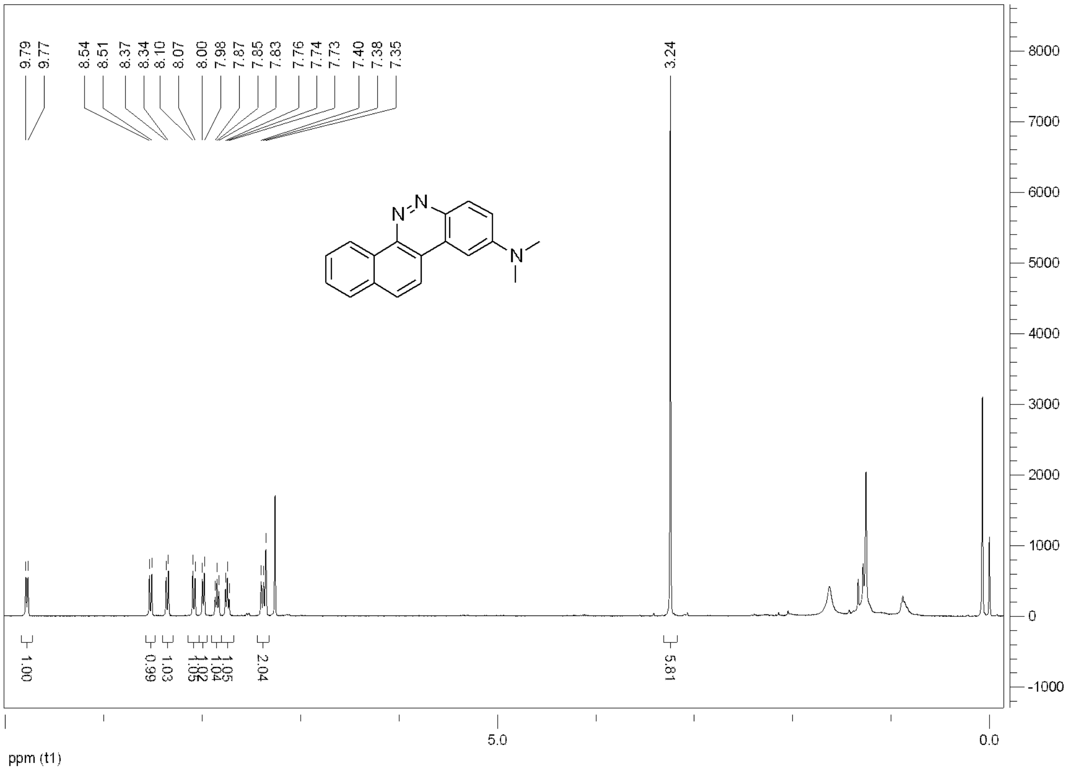

Figure S99: The ¹H-NMR spectrum of the cinnoline product from 8

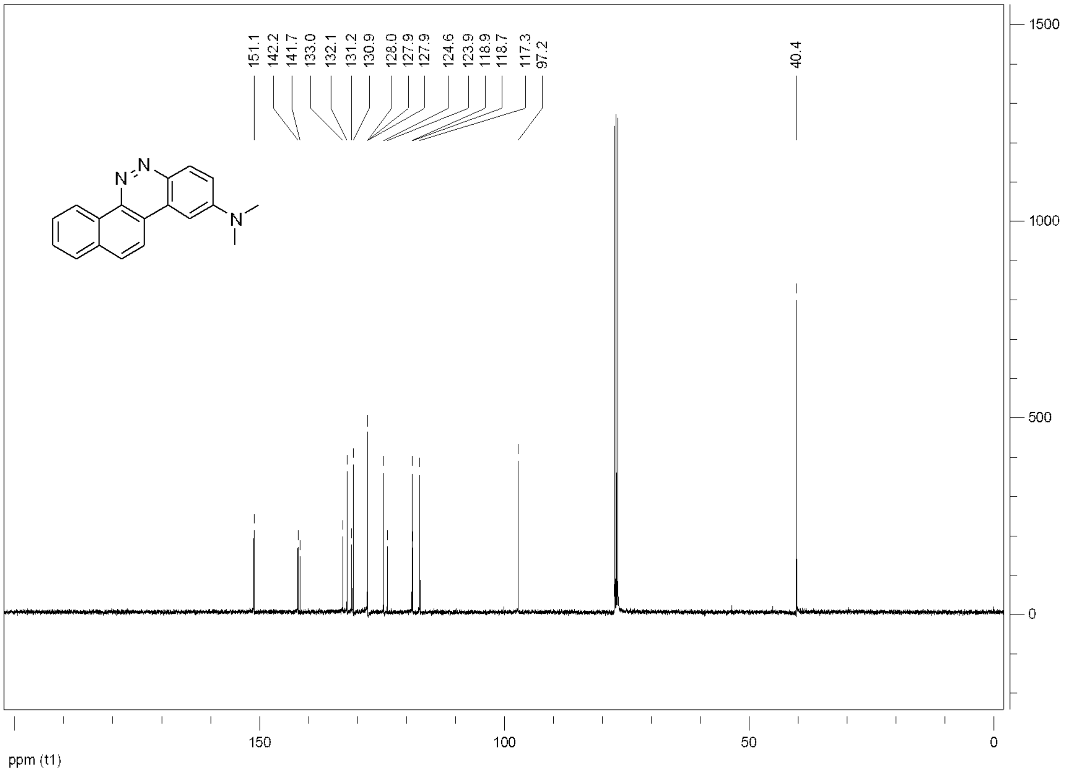

Figure S100: The ¹³C-NMR spectrum of the cinnoline product from 8

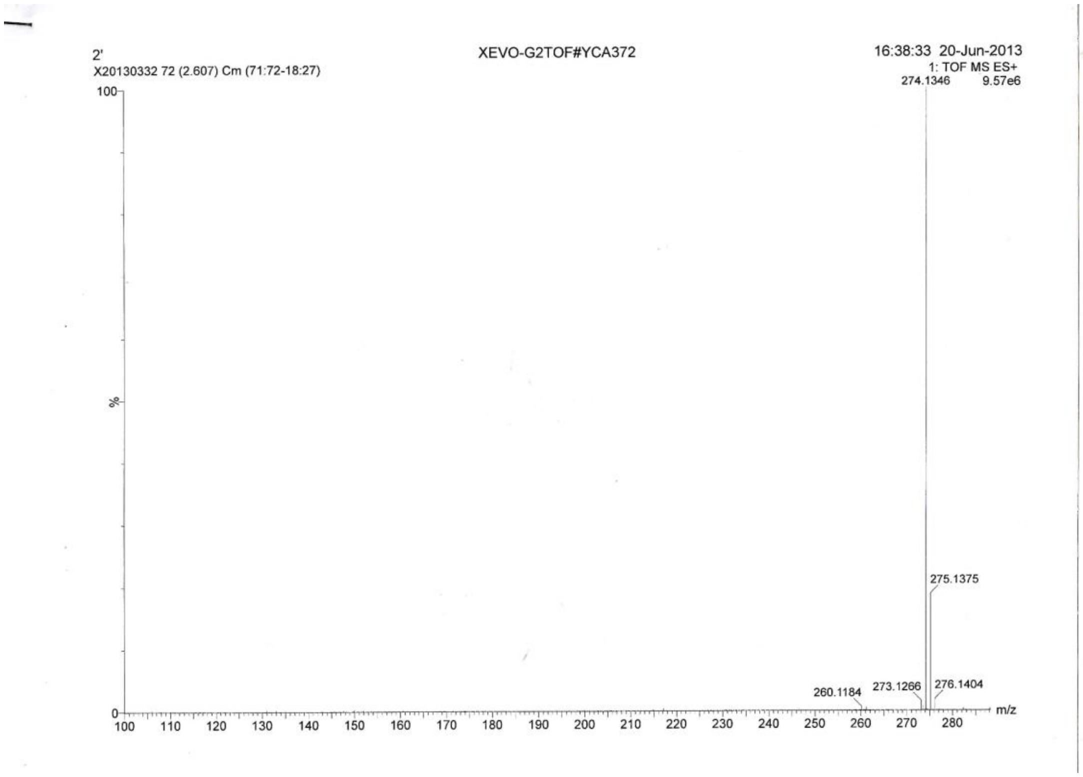

Figure S101: The HRMS spectrum of the cinnoline product from 8

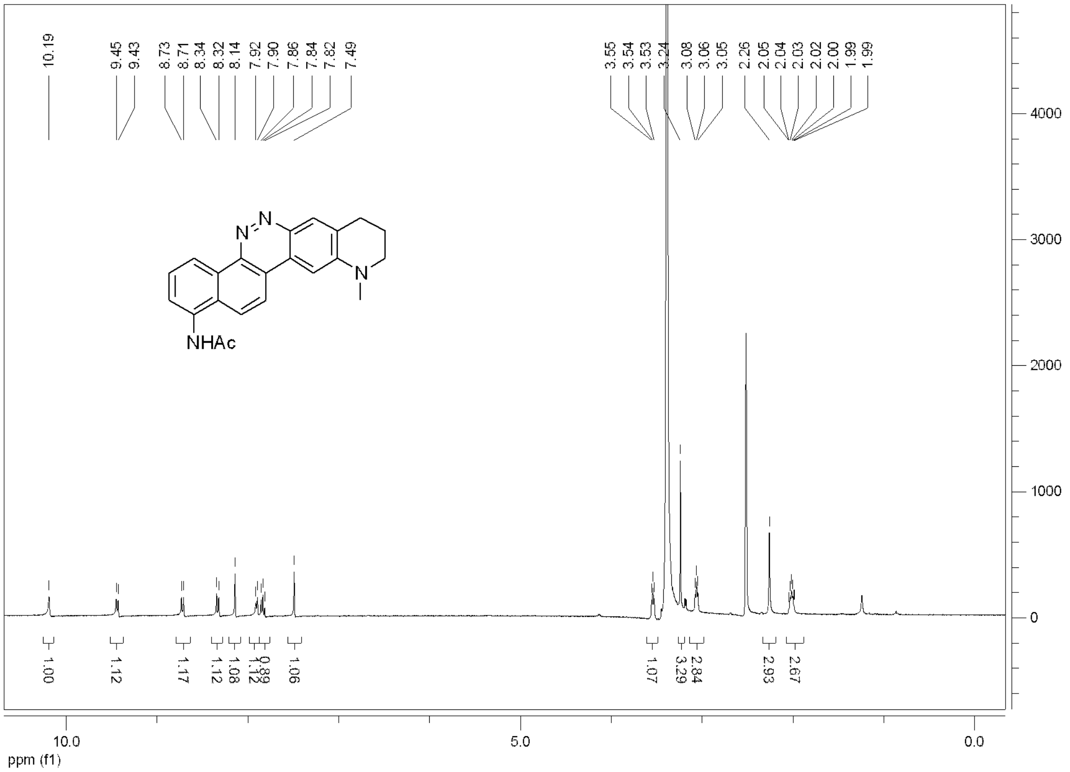

Figure S102: The ^{1}H-NMR spectrum of the cinnoline product from 10

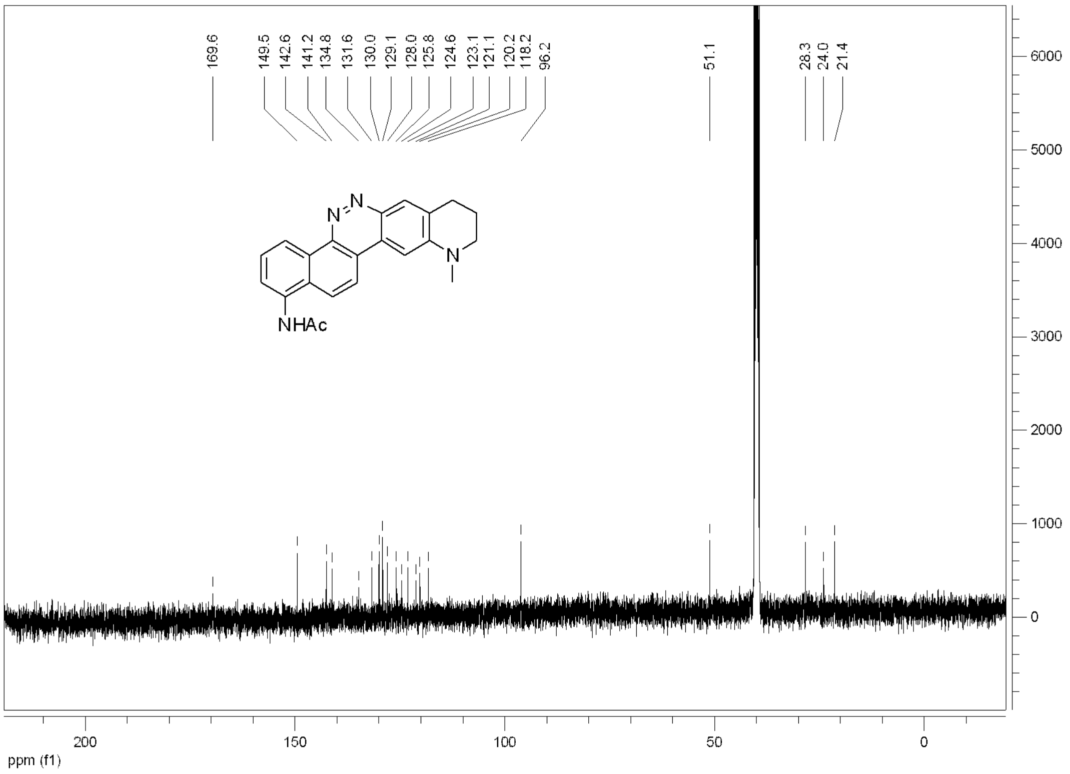

Figure S103: The ^{13}C-NMR spectrum of the cinnoline product from 10

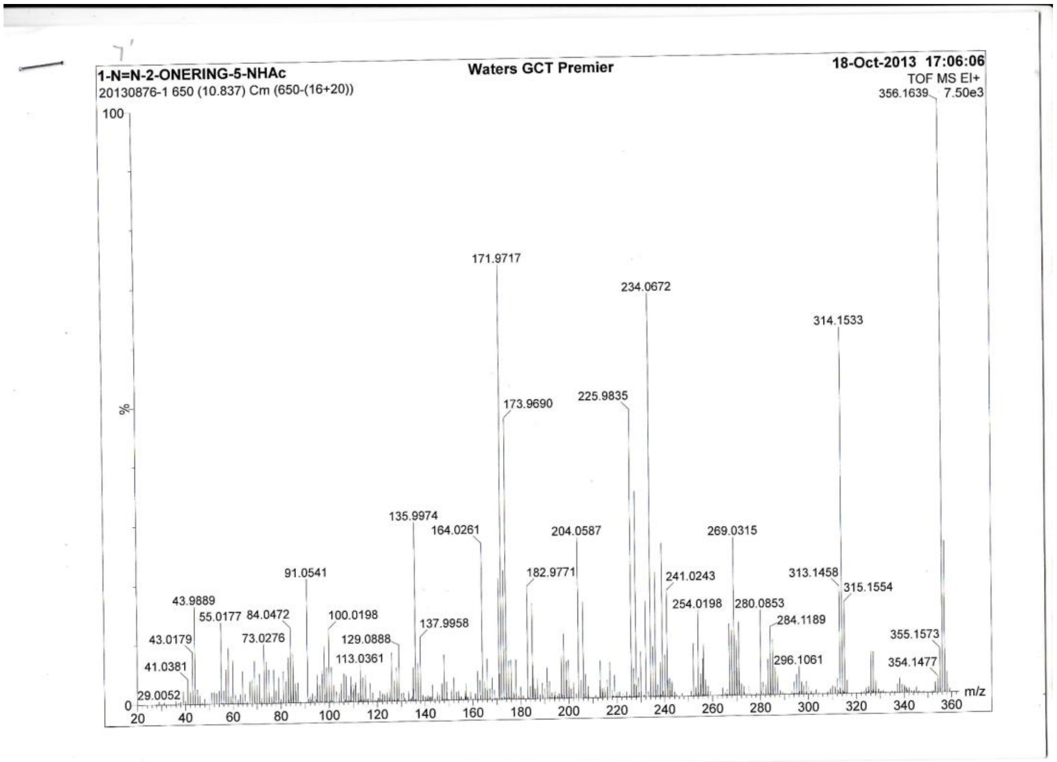

Figure S104: The HRMS spectrum of the cinnoline product from 10

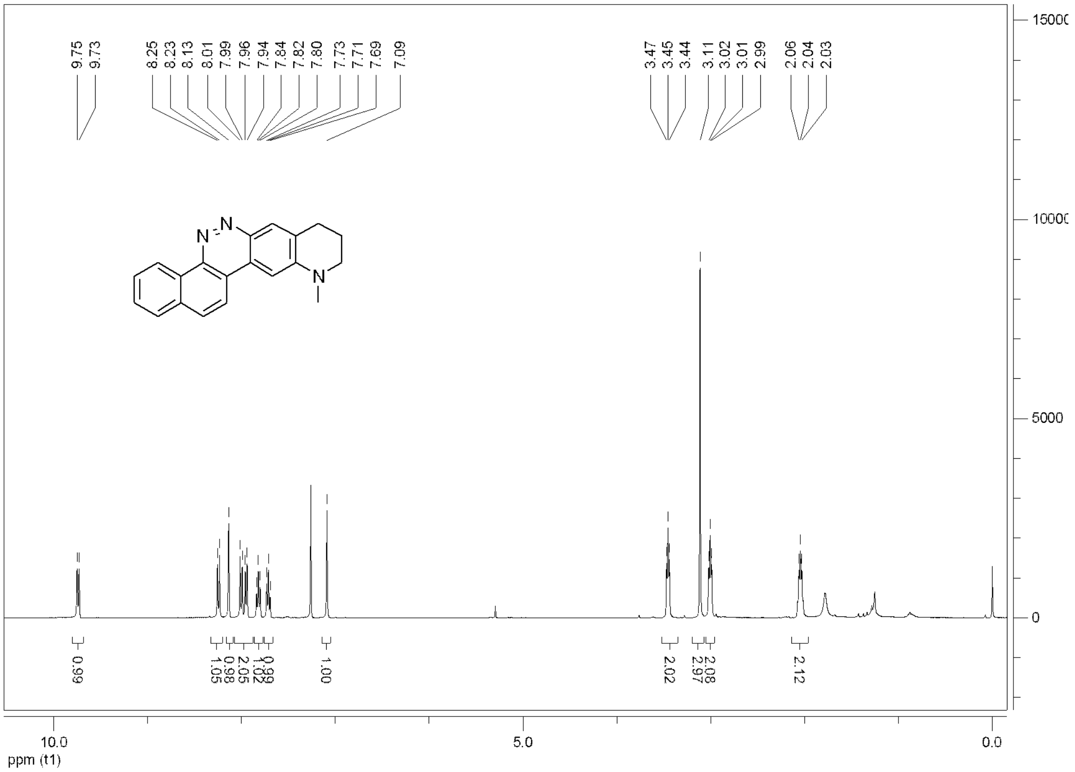

Figure S105: The ¹H-NMR spectrum of the cinnoline product from 11

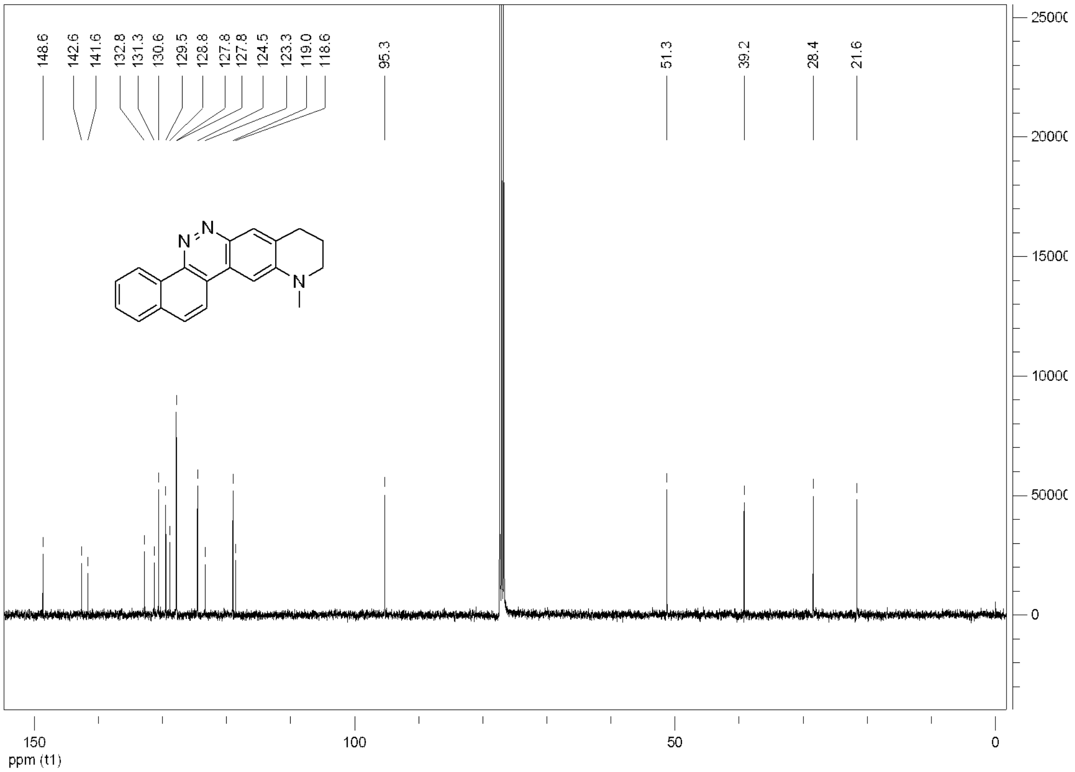

Figure S106: The ¹³C-NMR spectrum of the cinnoline product from 11

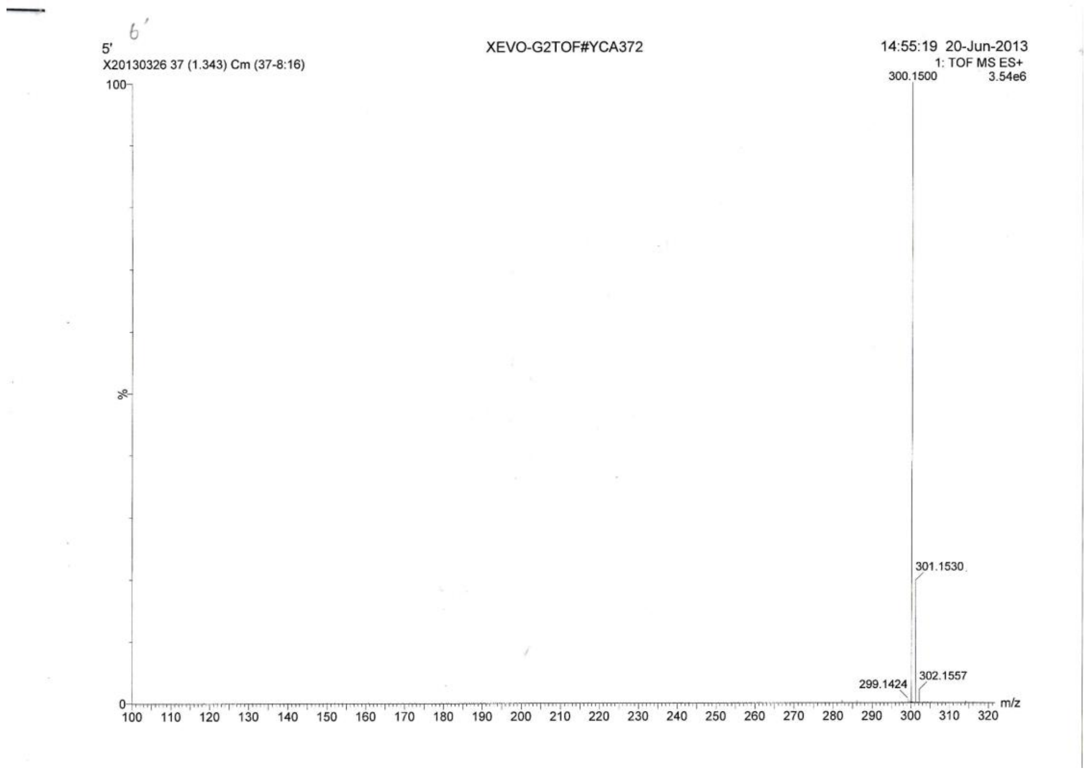

Figure S107: The HRMS spectrum of the cinnoline product from 11

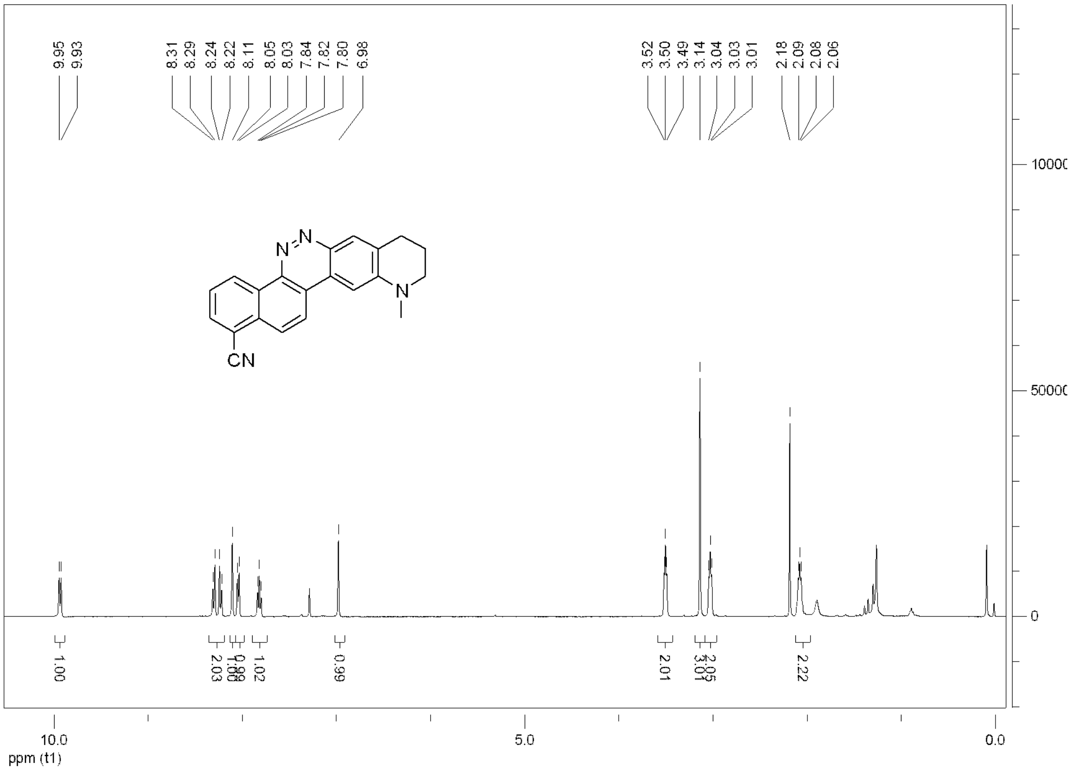

Figure S108: The ^{1}H-NMR spectrum of the cinnoline product from 12

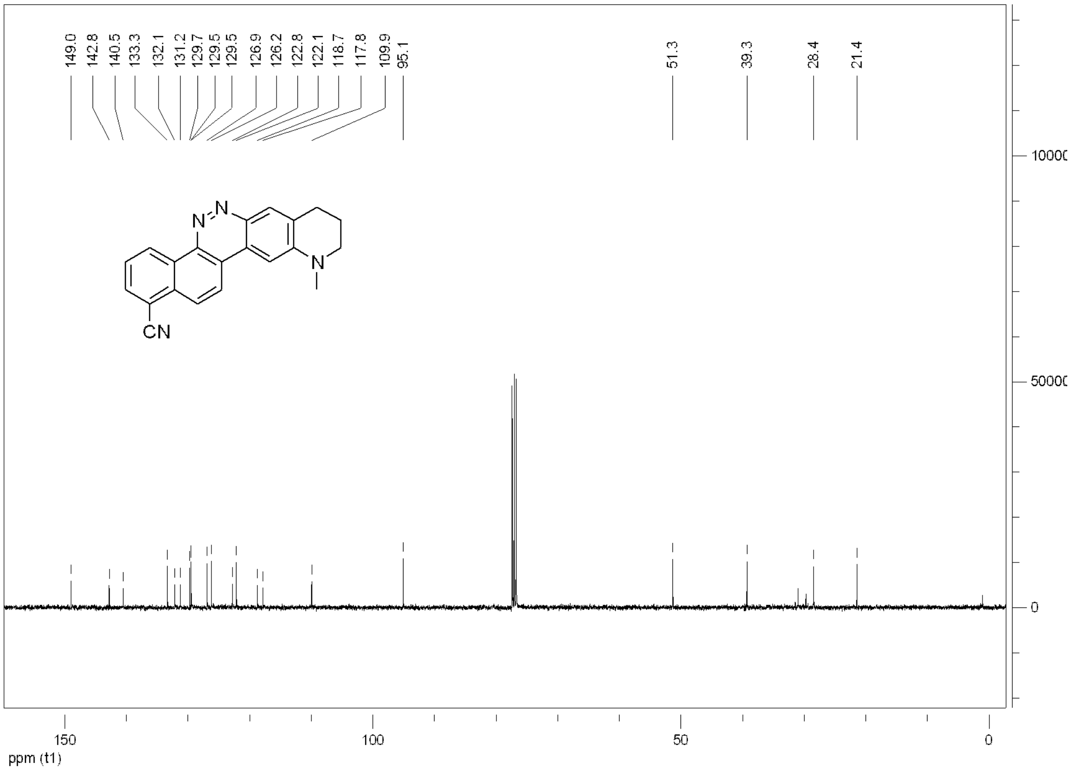

Figure S109: The ^{13}C-NMR spectrum of the cinnoline product from 12

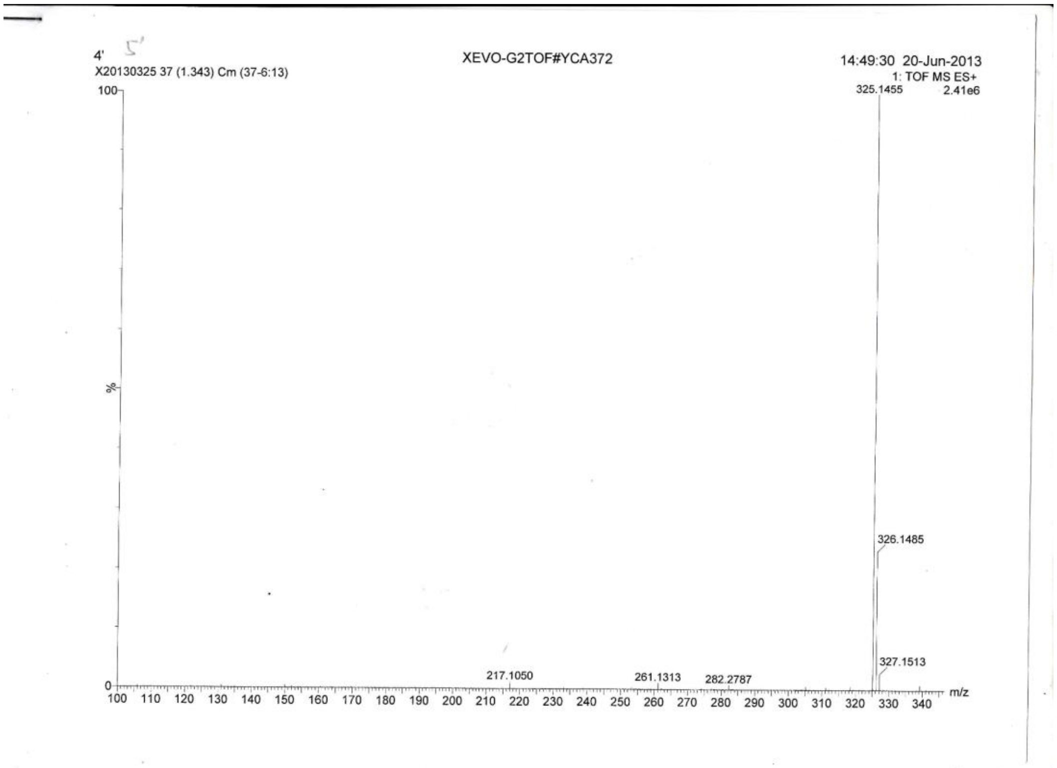

Figure S110: The HRMS spectrum of the cinnoline product from 12

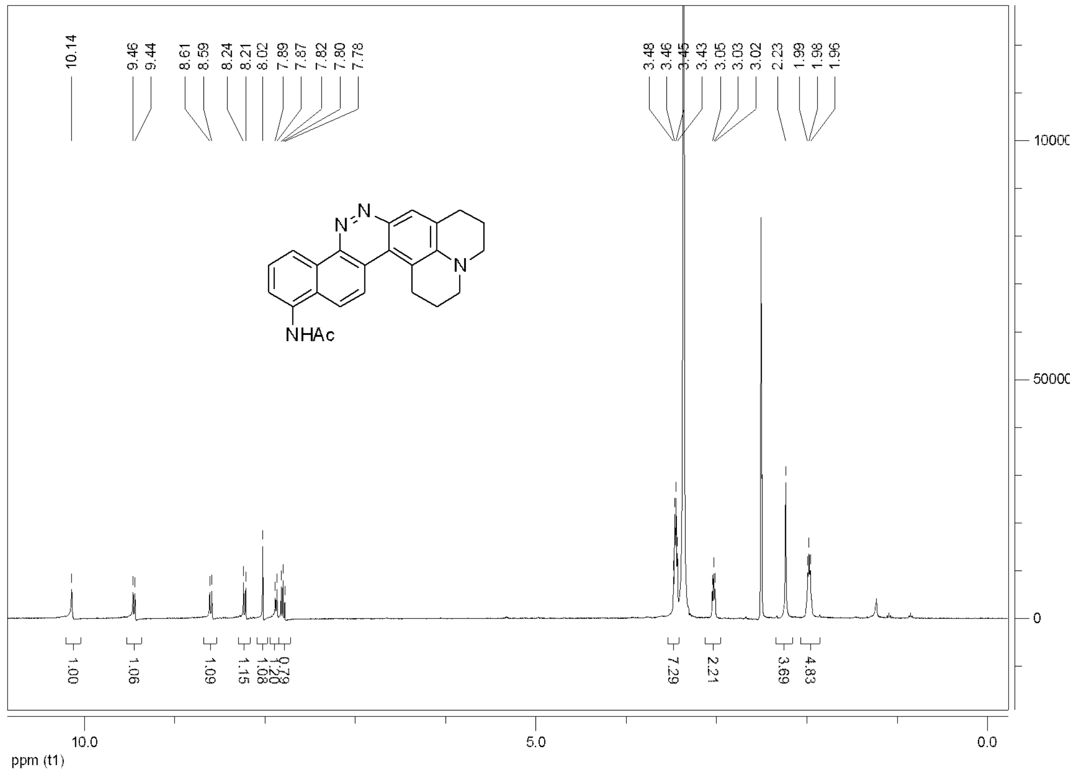

Figure S111: The ^{1}H-NMR spectrum of the cinnoline product from 13

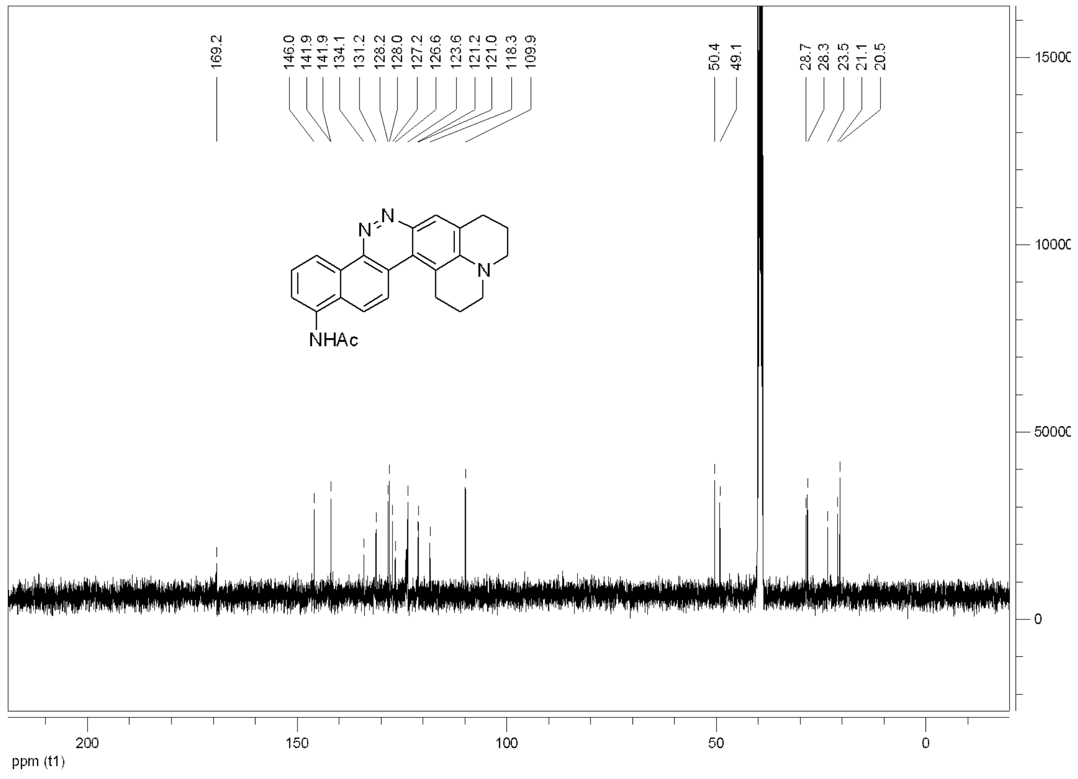

Figure S112: The ^{13}C-NMR spectrum of the cinnoline product from 13

Elemental Composition Report Page 1

Single Mass Analysis
Tolerance = 30.0 mDa / DBE: min = -1.5, max = 100.0
Element prediction: Off
Number of isotope peaks used for i-FIT = 2

Monoisotopic Mass, Even Electron Ions
382 formula(e) evaluated with 24 results within limits (up to 1 closest results for each mass)
Elements Used:
C: 0-30 H: 0-27 N: 0-7 O: 0-20

WB-ZHU ECUST institute of Fine Chem 11-Mar-2014 20:35:20

ZWB-THY-11 35 (1.160) Cm (28:36) 1: TOF MS ES+ 1.74e+003

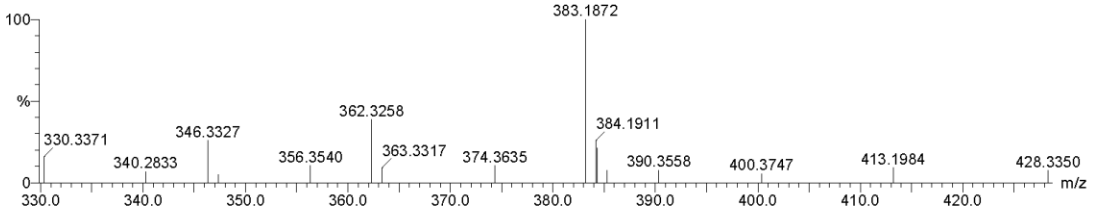

Minimum:				-1.5			
Maximum:		30.0	50.0	100.0			
Mass	Calc. Mass	mDa	PPM	DBE	i-FIT	i-FIT (Norm)	Formula
383.1872	383.1872	0.0	0.0	15.5	19.7	0.0	C24 H23 N4 O

Figure S113: The HRMS spectrum of the cinnoline product from 13

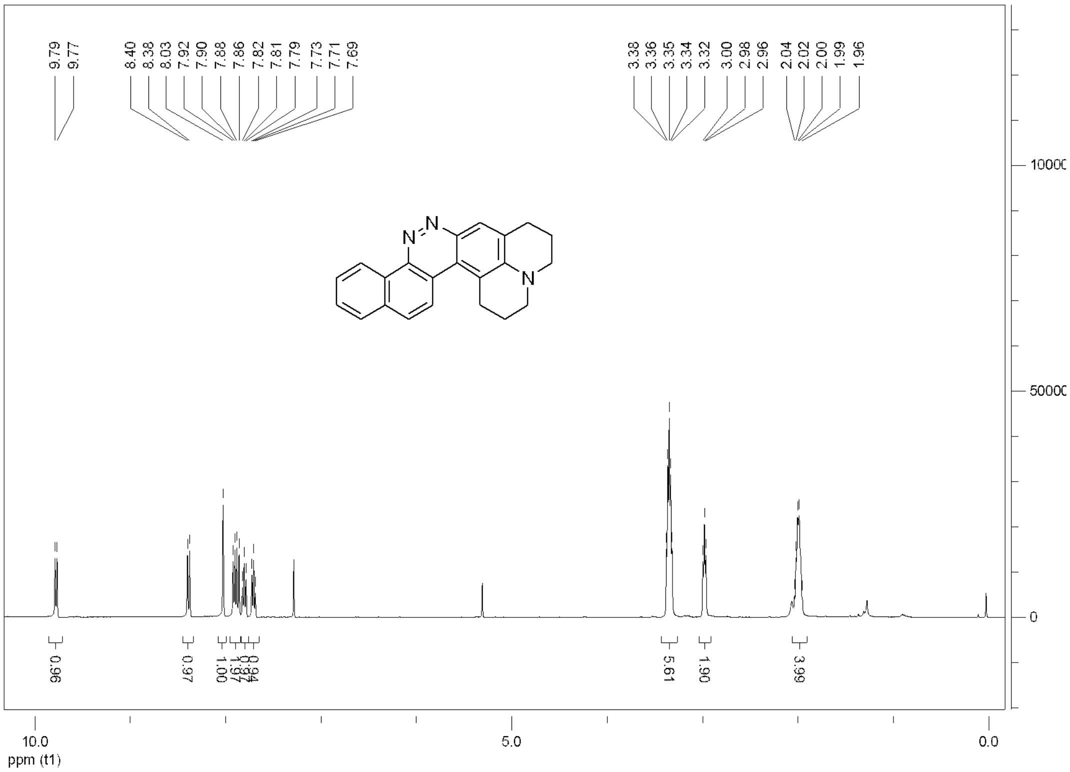

Figure S114: The ^{1}H-NMR spectrum of the cinnoline product from 14

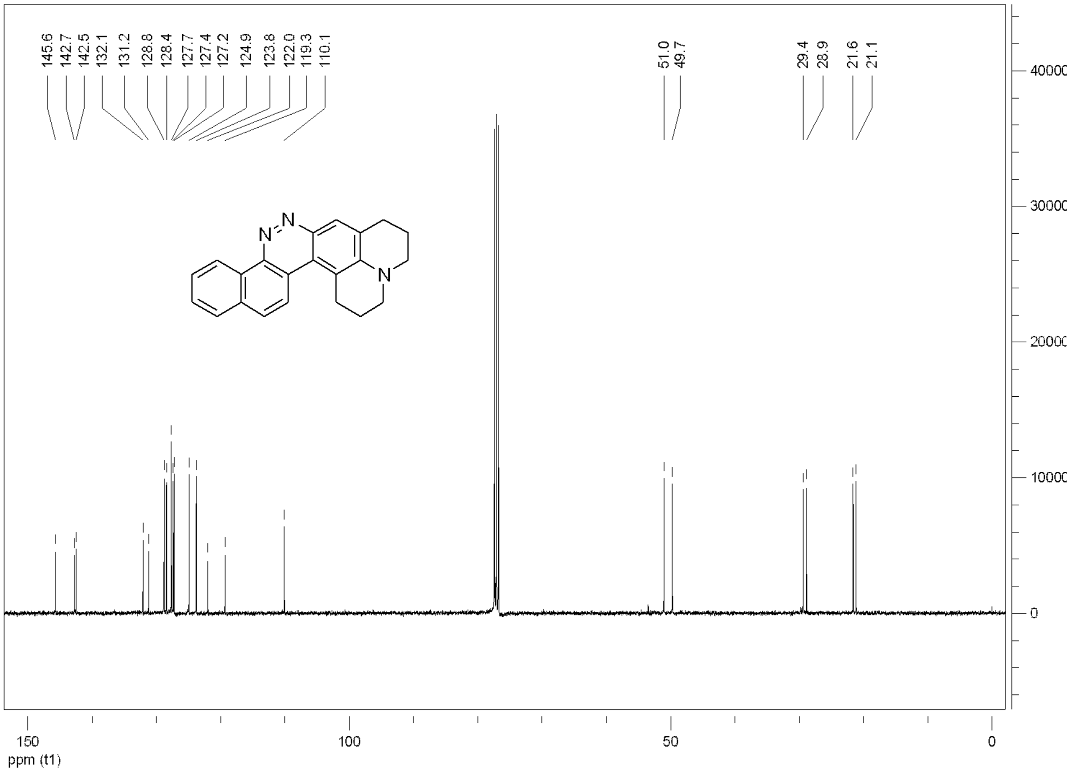

Figure S115: The ^{13}C-NMR spectrum of the cinnoline product from 14

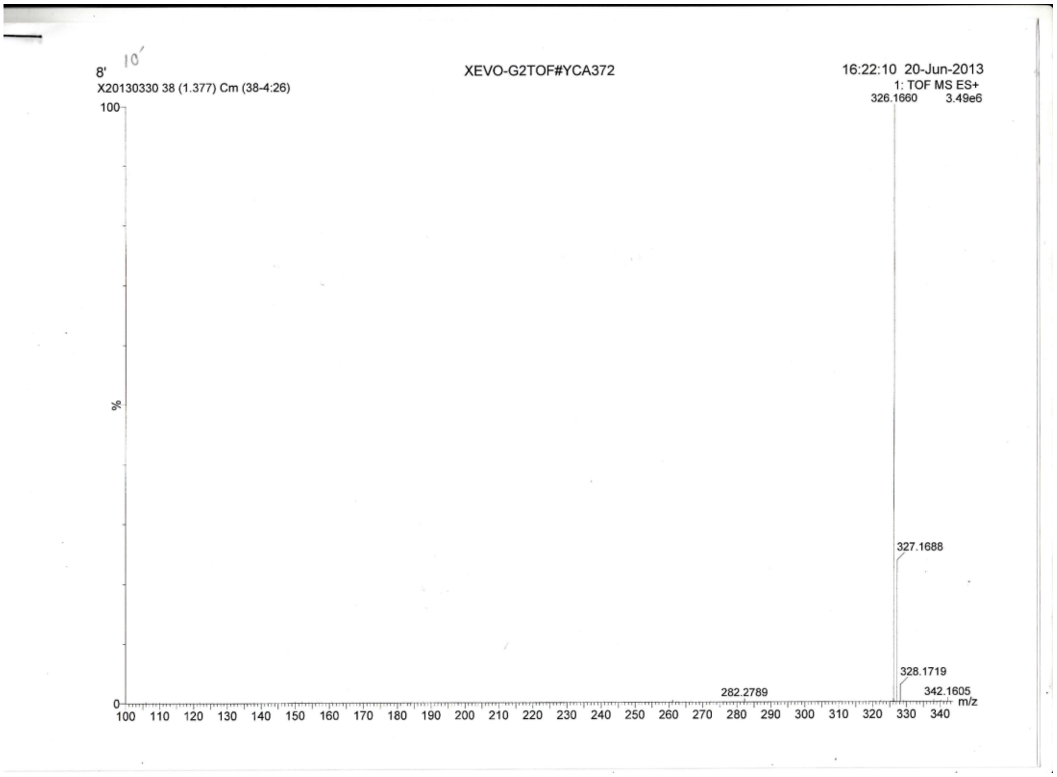

Figure S116: The HRMS spectrum of the cinnoline product from 14

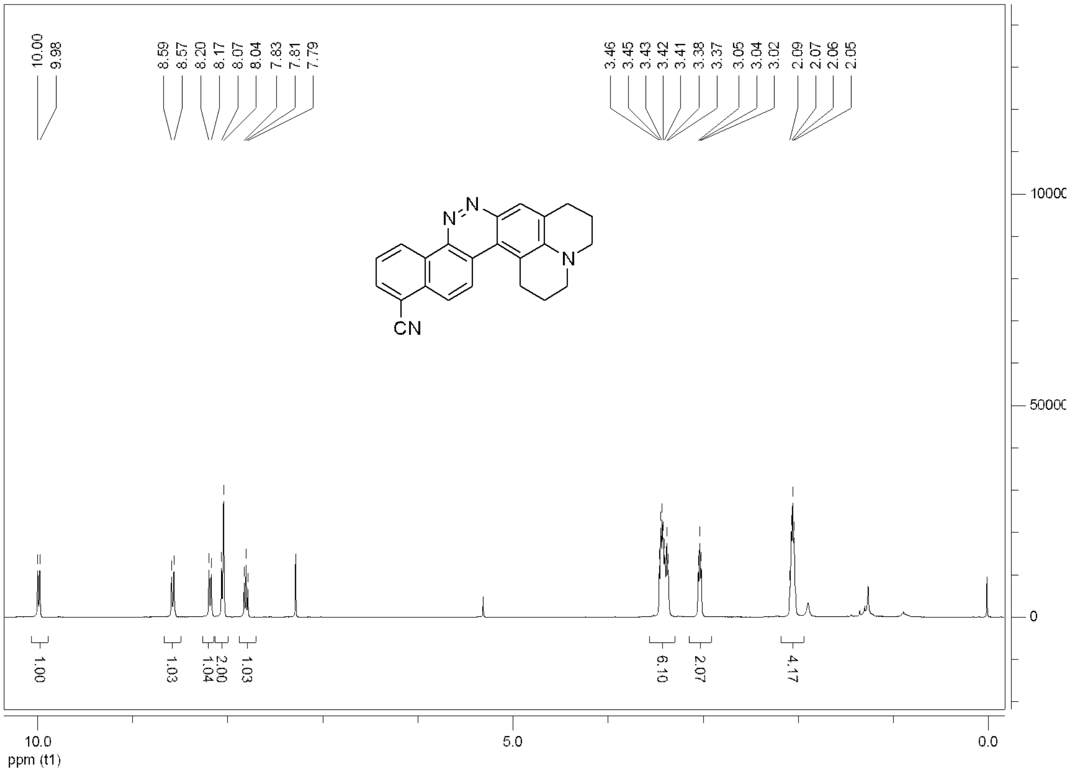

Figure S117: The ^{1}H-NMR spectrum of the cinnoline product from 15

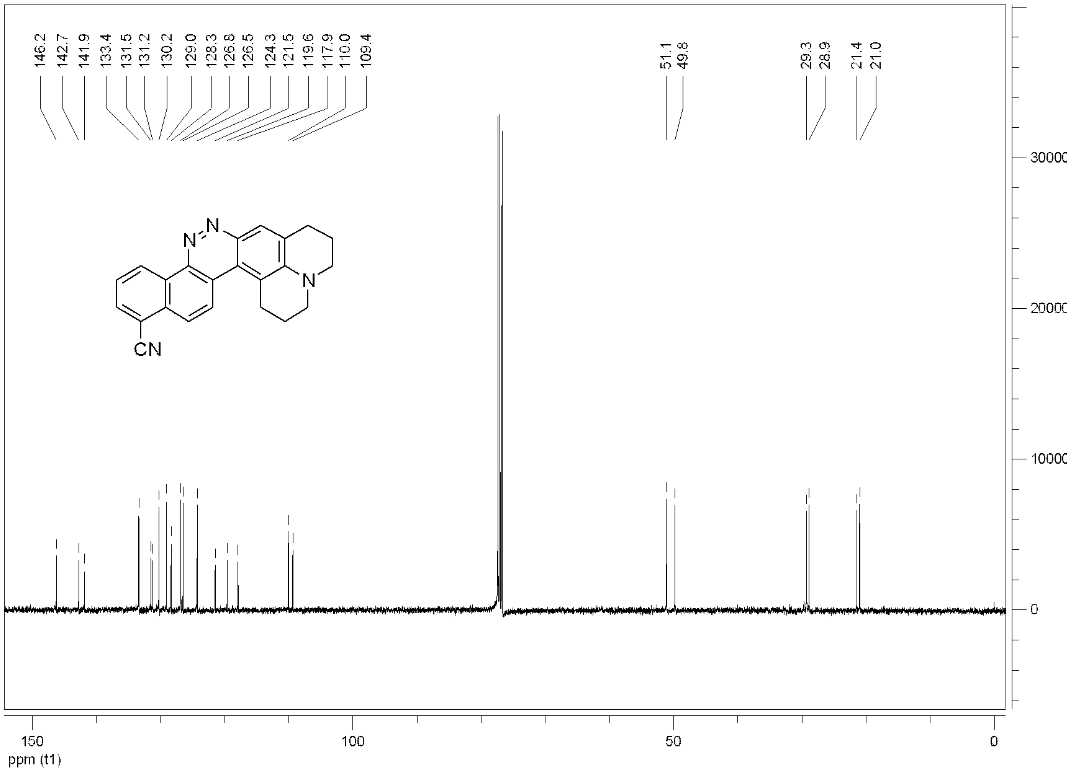

Figure S118: The ^{13}C-NMR spectrum of cinnoline product from 15

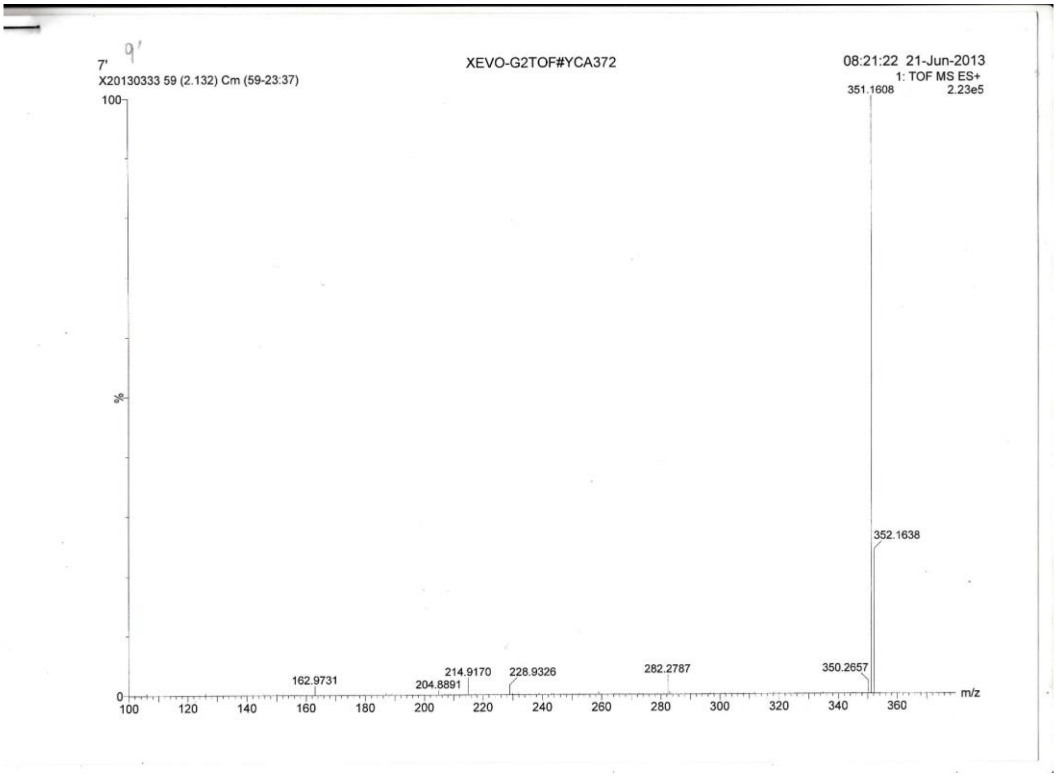

Figure S119: The HRMS spectrum of the cinnoline product from 15
